# Transition metal-free intramolecular regioselective couplings of aliphatic and aromatic C-H bonds

**DOI:** 10.1038/srep19931

**Published:** 2016-01-29

**Authors:** Hua Tian, Haijun Yang, Changjin Zhu, Hua Fu

**Affiliations:** 1 Key Laboratory of Bioorganic Phosphorus Chemistry and Chemical Biology (Ministry of Education), Department of Chemistry, Tsinghua University, Beijing 100084, P. R. China; 2 Department of Applied Chemistry, Beijing Institute of Technology, Beijing 100081, P. R. China

## Abstract

Cross-dehydrogenative couplings of two different C-H bonds have emerged as an attractive goal in organic synthesis. However, achieving regioselective C-H activation is a great challenge because C-H bonds are ubiquitous in organic compounds. Actually, the regioselective couplings promoted by enzymes are a common occurrence in nature. Herein, we have developed simple, efficient and general transition metal-free intramolecular couplings of alphatic and aromatic C-H bonds. The protocol uses readily available aryl triazene as the radical initiator, cheap K_2_S_2_O_8_ as the oxidant, and the couplings were performed well with excellent tolerance of functional groups. Interestingly, α-carbon configuration of some amino acid residues in the substrates was kept after the reactions, and the couplings for substrates with substituted phenylalanine residues exhibited complete β-carbon diastereoselectivity for induction of the chiral α-carbon. Therefore, the present study should provide a novel strategy for regioselective cross-dehydrogenative couplings of two different C-H bonds.

Constructing carbon-carbon and carbon-heteroatom bonds via carbon-hydrogen (C-H) activation has attracted much attention in chemical transformation, and this strategy can reduce the number of steps to the target molecule, and decrease cost and waste^1^,^2^,^3^,^4^,^5^,^6^,^7^. However, achieving regioselective C-H activation is a great challenge because C-H bonds are ubiquitous in organic molecules. The most common protocols are referred to as transition metal-catalyzed directed C-H bond functionalization, in which a directing functional group is able to coordinate to metal of the catalyst and the metal inserts a proximal C-H bond to get a thermodynamically stable five- or six-membered metallacyclic intermediate, and a subsequent reaction occurs at the *ortho* position of the directing group (Fig. 1a)^8^,^9^,^10^,^11^. There has been great progress in the cross-coupling of two different C-H bonds in the absence of directing group^12^,^13^,^14^,^15^,^16^,^17^,^18^, but the reactions usually occur on adjacent C-H bonds to a heteroatom (Fig. 1b), which is away from the ideal chemical transformation^19^. Therefore, the cross-coupling of two different C-H bonds that are not in close proximity to a heteroatom is in undeveloped stage in the absence of *ortho*-site directing group. Actually, the C-H activation is a common occurrence in nature^20^,^21^,^22^, and the regioselective cross-dehydrogenative couplings of two different C-H bonds promoted by enzymes are often found in the biosynthesis of natural products and biologically active molecules^23^,^24^,^25^,^26^,^27^,^28^,^29^,^30^,^31^,^32^,^33^,^34^,^35^,^36^,^37^,^38^. The replication of the enzymatic processes in the laboratory using simple and readily available natural resources is the best procedure^39^. Therefore, it is highly desirable to develop a chemical regioselective cross-coupling of C-H bonds under the inspiration of the enzymatic reactions. Aryl triazenes that are easily prepared from the corresponding anilines are both stable and adaptable to numerous synthetic transformations^40^,^41^,^42^,^43^,^44^. Recently, Baran and co-workers used *o*-tosyl triazene chloride (Tz^*o*^Cl) as the ‘portable desaturase’ to install on aliphatic amines or alcohols, and the desaturation of unactivated aliphatics was performed well when 2,2,6,6-tetramethylpiperidin-1-oxyl (TEMPO) and trifluoroacetic acid (TFA) or trifluoromethanesulfonic acid (TfOH) were added to the system^45^. Inspired by the previous excellent researches, we initiated our project on intramolecular cross-coupling of two different C-H bonds. As shown in Fig. 1c, our strategy is as follows: Conjugation of **A** with Tz^*o*^Cl on the amino gives **B**, **B** provides the corresponding aryl radical **C** under treatment of oxidant, and H-abstraction of aliphatic C-H bond by the aryl radical leads to alkyl radical **D** since aryl radicals were confirmed to be highly reactive, very short-lived intermediates^46^,^47^. Subsequent intramolecular electrophilic reaction of alkyl radical with aryl ring in **D** affords the target product **E**.

## Results and Discussion

### Development of a method

At first, 2-*N*-Tz^*o*^-amino-*N*,3-dimethyl-*N*-phenylbutanamide (**1a**) was chosen as the model substrate to optimize the reaction conditions including catalysts, oxidants, additives, solvents and temperature under nitrogen atmosphere. When TEMPO was used as the oxidant, TFA or TfOH as the additive referencing to Baran’s conditions^45^, the desired product (**2a**) was observed in only 6% and 7% yields using CH_3_NO_2_ as the solvent (Table 1, entries 1 and 2). Interestingly, use of two equiv of K_2_S_2_O_8_ as the oxidant led to a 50% yield in the absence of additive at 100 °C (Table 1, entry 3). Other solvents, CH_3_CN, 1,2-dichloroethane (DCE), toluene, dioxane, DMF and DMSO, were attempted (Table 1, entries 4–9), and CH_3_CN gave the highest yield (53%) (Table 1, entry 4). In order to confirm whether trace amount of transition metals in the system mediate this reaction, the solvent in the resulting solution of entry 4 was removed by a rotary evaporator, and the residue was determined by ICP mass spectrometry. Mn, Pd, Rh, Cu, Fe, Ni, Pt, Au, Ag, Co and Cr almost were not observed (Data determined by ICP mass spectrometry on the residue after the solvent in the resulting solution of entry 4 was removed by a rotary evaporator: Mn (0.1 ppm), Pd (<0.5 ppm), Rh (<0.5 ppm), Cu (44.1 ppm), Fe (72.1 ppm), Ni (4.1 ppm), Pt (<0.5 ppm), Au (8.3 ppm), Ag (11.6 ppm), Co (0.12 ppm) and Cr (2.11 ppm)). Structure of **2a** containing *L*-valine residue was identified by NMR, and the result showed that configuration of the chiral α-carbon in **2a** was retentive and no racemization was observed. Other oxidants were tested (Table 1, entries 10–17), and they were inferior to K_2_S_2_O_8_ (Table 1, entry 4). Yield obviously dropped when temperature was decreased to 60 °C (Table 1, entry 18). Reducing amount of K_2_S_2_O_8_ to one equivalent led to a lower yield (Table 1, entry 19), and increasing its amount to three equivalents gave a similar yield to entry 4 (see Table 1, entry 20). Three common transition-metal catalysts, Pd(OAc)_2_, CuBr_2_ and AgNO_3_ (Table 1, entries 21–23), were added to the reaction system, respectively, and the results showed that addition of transition-metal catalysts did not improve efficiency of the reaction, which exhibits that the present reaction is a transition metal-free process. Reaction in air provided a lower yield (Table 1, entry 24). Therefore, the optimal conditions for the intramolecular coupling of aliphatic and aromatic C-H bonds are as follows: two equiv of K_2_S_2_O_8_ as the oxidant, CH_3_CN as the solvent at 100 °C for 2 h under nitrogen atmosphere.

### Couplings of aliphatic tertiary C-H and aromatic C-H bonds

**Figure 2:**
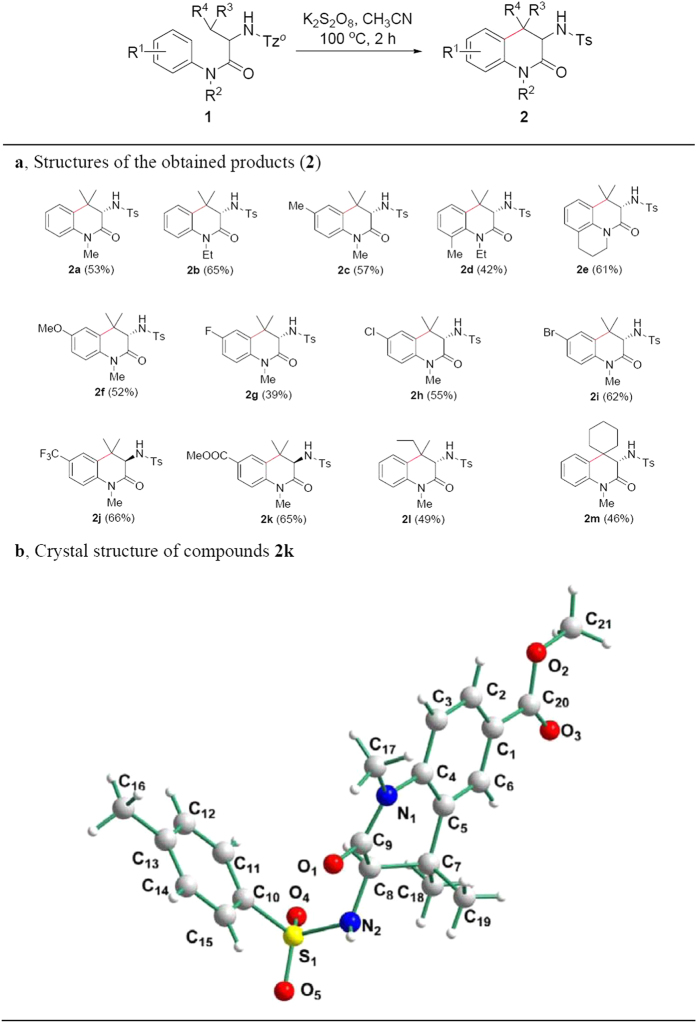
Substrate scope for couplings of aliphatic tertiary C-H and aromatic C-H bonds. (**a)** Structures of the obtained products (**2**). (**b**) Crystal structure of compounds **2k**. Reaction condition: under nitrogen atmosphere, **1** (0.1 mmol), K_2_S_2_O_8_ (0.2 mmol), CH_3_CN (2.0 mL), reaction temperature (100 °C), reaction time (2 h) in a sealed Schlenk tube. ^*^Isolated yield.

We investigated the scope of substrates containing *L*- and *D*-amino acid residues for intramolecular couplings of aliphatic tertiary C-H and aromatic C-H bonds (Fig. 2). When R^2^ in the substrate was ethyl in stead of methyl in **1a**, **2b** was obtained in 65% yield. Various substitutes for R^1^ were attempted. As shown in Fig. 2a, the substrate with *p*-Me relative to NR^2^ on the aromatic ring provided higher yield than that with *o*-Me relative to NR^2^ because of steric hindrance (compare **2c** and **2d** in Fig. 2a). Existence of piperidine in **2e** was favor for the intramolcular coupling of aliphatic tertiary C-H and aromatic C-H bonds because *N*-Tz^*o*^-valine and aromatic C-H bond were on the same side of tetrahydroquinoline. NMR data exhibited that α-carbon configuration in the chiral amino acid residues is kept. In order to further ascertain structures of the newly synthesized products (**2**), **2k** was made from the substrate with *D*-valine residue, its single crystal was prepared (see Supporting Information for details), and X-ray diffraction analysis showed that α-carbon configuration of *D*-valine residue in **2k** was remained (Fig. 2b). The reactions showed good tolerance of functional groups including ether (see **2f** in Fig. 2a), C-F bond (see **2g** in Fig. 2a), C-Cl bond (see **2h** in Fig. 2a), C-Br bond (see **2i** in Fig. 2a), trifluoromethyl (see **2j** in Fig. 2a), ester (see **2k** in Fig. 2a). We also attempted substrates containing different R^3^ and R^4^, and they afforded the reasonable yields (see **2l** and **2m** in Fig. 2a).

### Couplings of benzylic C-H and aromatic C-H bonds

**Figure 3:**
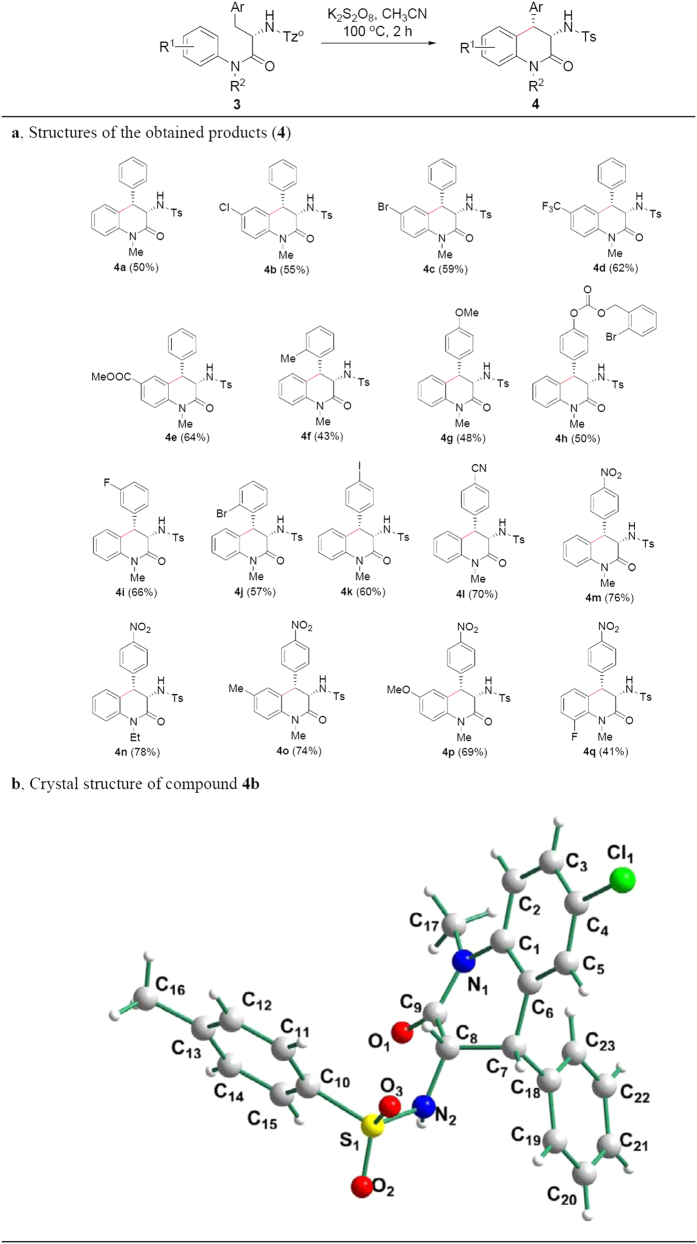
Substrate scope for couplings of benzylic C-H and aromatic C-H bonds. **(a**) Structures of the obtained products (**4**). (**b**) Crystal structure of compounds **4b**. Reaction condition: under nitrogen atmosphere, **3** (0.1 mmol), K_2_S_2_O_8_ (0.2 mmol), CH_3_CN (2.0 mL), reaction temperature (100 °C), reaction time (2 h) in a sealed Schlenk tube. ^*^Isolated yield. Structures of diastereomers were identified by ^1^H NMR analysis and X-ray diffraction analysis.

Inspired by the excellent results above, we extended the substrate scope using substituted phenylalanine residues in **3** in stead of the above amino acid residues containing tertiary C-H bond in **1**. As shown in Fig. 3a, the substrates with phenylalanine residue provided the reasonable yields under the standard conditions (see **4a**–**e** in Fig. 3a), and those with electron-withdrawing groups at *para*-site of NMe on the aromatic ring exhibited higher reactivity (see **4d** and **4e** in Fig. 3a). We attempted other substrates with different substituted phenylalanine residues, and they also gave good results. The substrates containing electron-withdrawing groups on aromatic ring of phenylalanine residue (see **4l**–**q** in Fig. 3a) afforded higher yields because of higher acidity of benzylic C-H than those containing electron-donating groups (see **4g** and **4h** in Fig. 3a). Similarly to **2k**, single crystal of **4b** was prepared, and its structure was identified by X-ray diffraction analysis (Fig. 3b) (see Supporting Information for details). The result showed that the couplings displayed complete *β*-carbon diastereoselectivity because of effect of *ortho*-site α-chiral carbon in the amino acid residues, and they all were *cis*-form configuration. The couplings of benzylic C-H and aromatic C-H bonds exhibited excellent tolerance of functional groups including C-F bond (see **4i** and **4q** in Fig. 3a), C-Cl bond (see **4b** in Fig. 3a), C-Br bond (see **4c** and **4j** in Fig. 3a), C-I bond (see **4k** in Fig. 3a), trifluoromethyl (see **4d** in Fig. 3a), esters (see **4e** and **4h** in Fig. 3a), ether (see **4g** in Fig. 3a), nitrile (see **4l** in Fig. 3a), nitro (see **4m**–**q** in Fig. 3a).

### Couplings of α-C-H bond of carbonyl and aromatic C-H bonds

**Figure 4:**
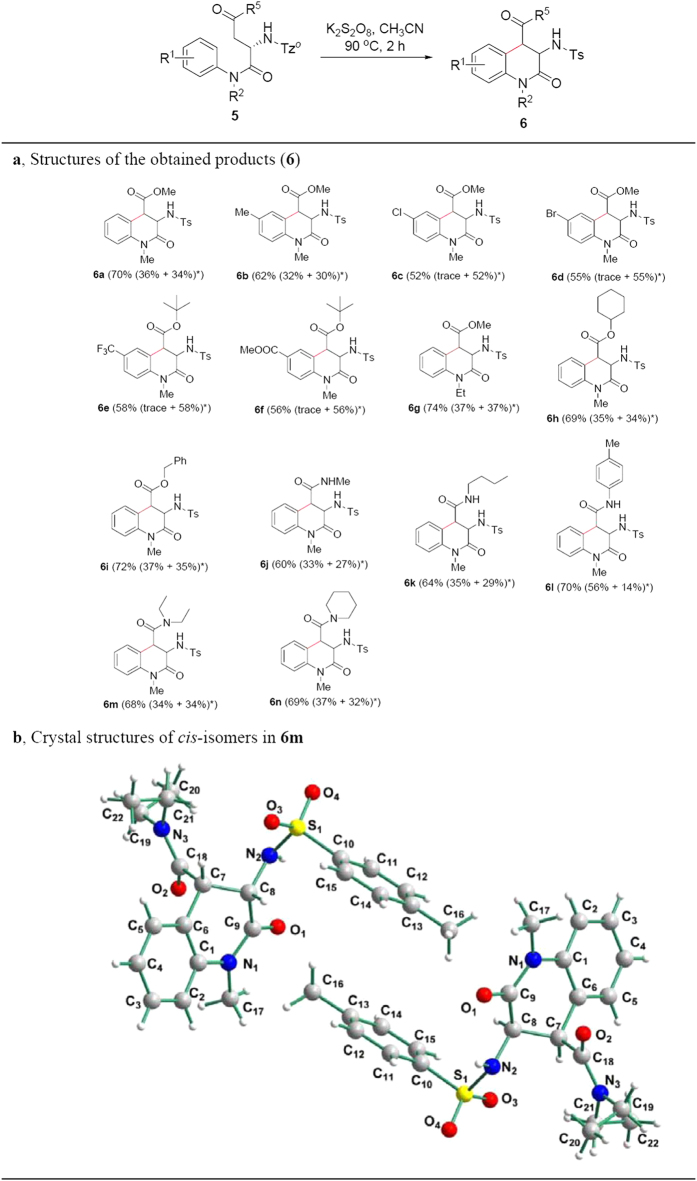
Substrate scope for couplings of α-C-H bond of carbonyl and aromatic C-H bond. **(a**) Structures of the obtained products (**6**). (**b**) Crystal structure of compounds **6m**. Reaction condition: under nitrogen atmosphere, **5** (0.1 mmol), K_2_S_2_O_8_ (0.2 mmol), CH_3_CN (2.0 mL), reaction temperature (100 °C), reaction time (2 h) in a sealed Schlenk tube. ^*^Isolated yield (*cis*- and *trans*-isomers were separated by silica gel column chromatography, and their structures were identified by ^1^H NMR analysis).

We further investigated intramolecular couplings of α-C-H bond of carbonyl and aromatic C-H bond for the substrates (**5**) containing aspartic acid derivatives. As shown in Fig. 4a, the examined substrates provided moderate to good yields. Unfortunately, the reactions led to racemization of α-carbon in aspartic acid residue because of unknown factors, and *cis*- and *trans*-forms were observed through coupling constants between α- and β-C-H. Interestingly, *cis*- and *trans*-isomers were isolated by silicon gel column chromatography, and their structures were identified by ^1^H NMR analysis. Single crystal of *cis*-isomers in **6m** was prepared using similar procedures to **2k**, and X-ray diffraction analysis exhibited that two *cis*-forms were observed (Fig. 4b) (see Supporting Information for details). In addition, for substrates containing electron-withdrawing groups R^1^ such as Cl, Br, CF_3_ and ester, *trans*-form diastereomers were major products (see **6c–f** in Fig. 4a), and that containing the bigger amide afforded major *cis*-form diastereomer (see **6l** in Fig. 4a). The method also displayed good tolerance of functional groups including esters (see **6a**–**i** in Fig. 4a), amides (all the substrates in Fig. 4a), C-Cl bond (see **6c** in Fig. 4a), C-Br bond (see **6d** in Fig. 4a), and CF_3_ (see **6e** in Fig. 4a).

### Mechanistic investigations

**Figure 5:**
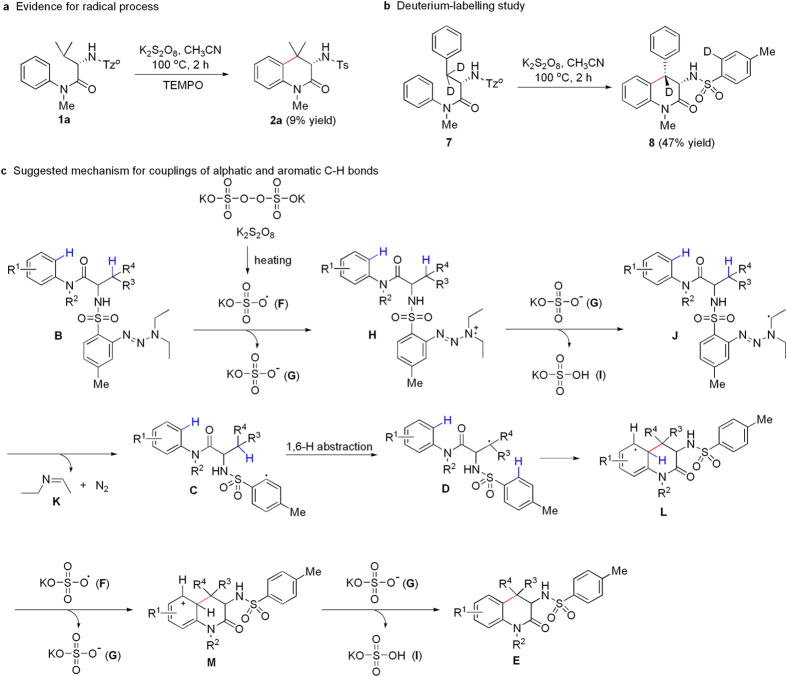
Mechanistic investigations. **(a)** Evidence for the *in situ* formation of an intermediate aryl radical. (**b)** Deuterium-labelling study to support a H-abstraction event during coupling of alphatic and aromatic C-H bonds. (**c**) Proposed reaction mechanism for couplings of alphatic and aromatic C-H bonds.

In order to explore mechanism on the couplings of aliphatic and aromatic C-H bonds, the following control experiments were performed. As shown in Fig. 5a, treatment of 2-*N*-Tz^*o*^-amino-*N*,3-dimethyl-*N*-phenylbutanamide (**1a**) with K_2_S_2_O_8_ was performed in the presence of one equiv of 2,2,6,6-tetramethylpiperidinyl-1-oxyl (TEMPO) under the standard conditions, and only 9%-yielded product (**2a**) was obtained. The result showed that the reaction could undergo a radical intermediate process. Deuterium-labelling phenylalanine was prepared according to the previous procedure^48^, and intramolecular coupling of substrate **7** was carried out under the standard conditions (Fig. 5b). Pleasedly, product **8** was obtained in 47% yield, which implied transfer of deuterium from alphatic *β*-C-D bond to aromatic C-D bond. Therefore, a possible reaction mechanism for couplings of alphatic and aromatic C-H bonds is proposed in Fig. 5c. First, homolysis of K_2_S_2_O_8_ yields radical **F** under heating condition, and treatment of substrate **B** with **F** provides radical cation **H** freeing anion **G**. Deprotonation of **H** by **G** gives radical **J** leaving **I**, and subsequent desorption of *N*-ethylideneethanamine (**K**) and N_2_ leads to highly reactive aryl radical **C**. Intramolecular 1,6-H abstraction from alphatic C-H bond to aromatic C-H bond donates alphatic alkyl radical **D**, and cyclization of **D** affords radical **L**. Treatment of **L** with **F** produces cation **M** leaving **G**, and deprotonation of **M** in the presence of **G** provides the target product (**E**).

### Application of the methods

**Figure 6:**
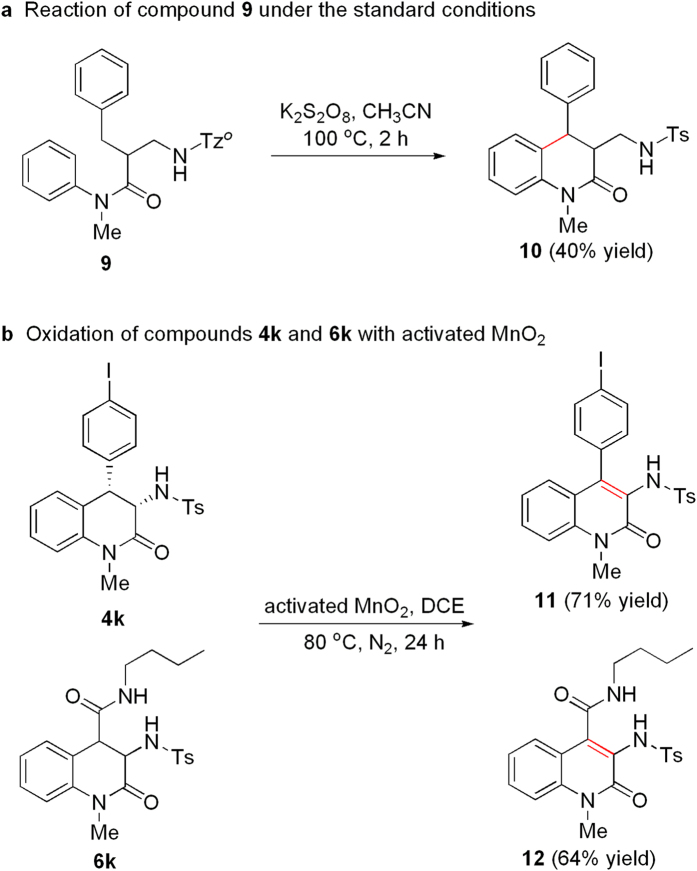
Application of the methods. **(a)** Treatment of compound **9** under the standard conditions. **(b)** Oxidation of compounds **4k** and **6k** with activated MnO_2_.

In order to explore affect of position for hydrogen-transfer from alphatic C-H to aromatic C-H, compounds **9** was prepared and treated under the standard conditions. Pleasedly, product **10** was obtained in 40% yield (Fig. 6a). The result showed that 1,7-H abstraction is also feasible for the coupling of alphatic and aromatic C-H bonds. We attempted oxidation of **4k** and **6k** to lead to quinolinones. As shown in Fig. 6b, treatment of **4k** or **6k** with ten equiv of activated MnO_2_ was performed in 1,2-dichloroethane (DCE) at 80 °C for 24 h, and the corresponding quinolinones **11** or **12** was obtained in 71% and 64% yields, respectively. Therefore, the present study is effective for synthesis of quinolinone derivatives.

## Conclusion

We have developed simple, efficient and general transition metal-free intramolecular regioselective cross-dehydrogenative couplings of alphatic and aromatic C-H bonds. The protocol uses readily available aryl triazene as the radical initiator, cheap K_2_S_2_O_8_ as the oxidant, and the couplings were performed well under mild conditions with excellent tolerance towards various functional groups. Interestingly, *α*-configuration of some amino acid residues in the substrates was kept after the reactions, and the couplings for substrates containing substituted phenylalanine residues exhibited complete *β*-carbon diastereoselectivity because of effect of *ortho*-site *α*-chiral carbon. Although the reactions for substrates containing aspartic acid derivatives gave *cis*- and *trans*-form racemates, the *cis*- and *trans*-isomers could be easily separated by silica gel column chromatography. The reaction mechanism indicated that initiation of reactions began in formation of aryl radicals from treatment of aryl triazenes with K_2_S_2_O_8_, in which aryl triazene seems to act as a radical initiator, and the reactions immediately process once K_2_S_2_O_8_ starts. Therefore, the present method should provide a new strategy for intramolecular regioselective cross-dehydrogenative couplings of two different C-H bonds.

## Methods

To a 25 mL Schlenk tube charged with a magnetic stirrer, **1**, **3** or **5** (0.1 mmol), K_2_S_2_O_8_ (0.2 mmol, 54 mg) and anhydrous MeCN (2.0 mL) were added. The tube was evacuated and back-filled with nitrogen for three cycles and then sealed. It was placed in a preheated oil bath at 100 °C, and the reaction was allowed to proceed for 2 hours. After completion of the reaction, the resulting mixture was filtered, and the filtrate was concentrated by a rotary evaporator. The residue was dissolved with EtOAc (3 mL), and the solution was washed with water (2 × 3 mL) and brine (2 × 3 mL), dried over MgSO_4_, filtered and concentrated by a rotary evaporation. The residue was purified with preparative TLC (silica gel, petroleum ether/EtOAc or dichloromethane/MeOH) to provide the target product (**2**, **4** or **6**).

## Additional Information

**How to cite this article**: Tian, H. *et al.* Transition metal-free intramolecular regioselective couplings of aliphatic and aromatic C-H bonds. *Sci. Rep.* **6**, 19931; doi: 10.1038/srep19931 (2016).

## Supplementary Material

Supplementary Information

## Figures and Tables

**Figure 1:**
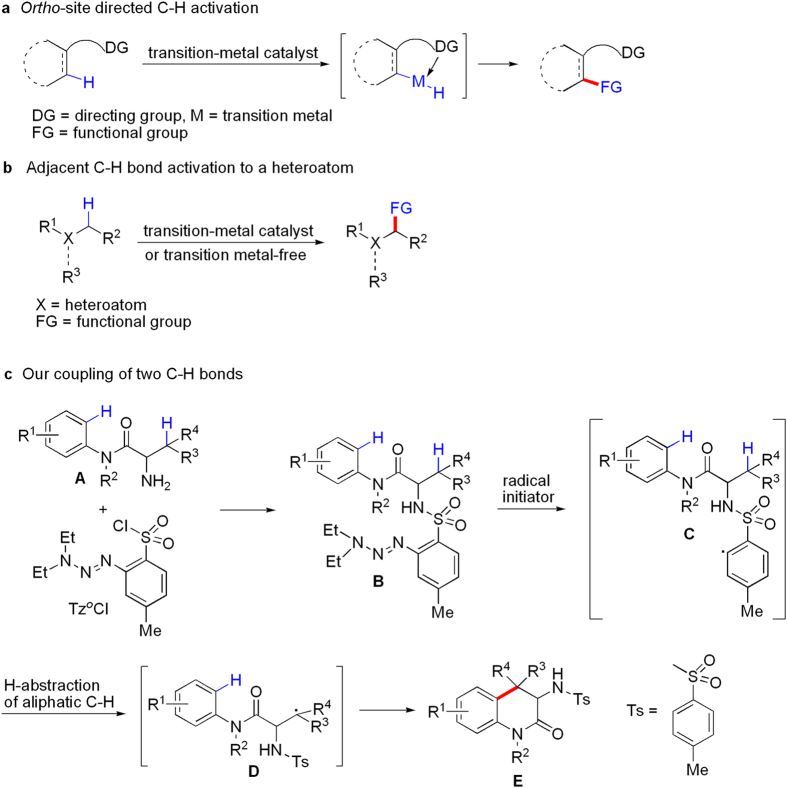
Design on transition metal-free intramolecular coupling of unactivated aliphatic and aromatic C-H bonds. (**a**) *Ortho*-site directed C-H activation. (**b**) Adjacent C-H bond activation to a heteroatom. (**c**) Description of the concept on our coupling of two C-H bonds.

**Table 1:**
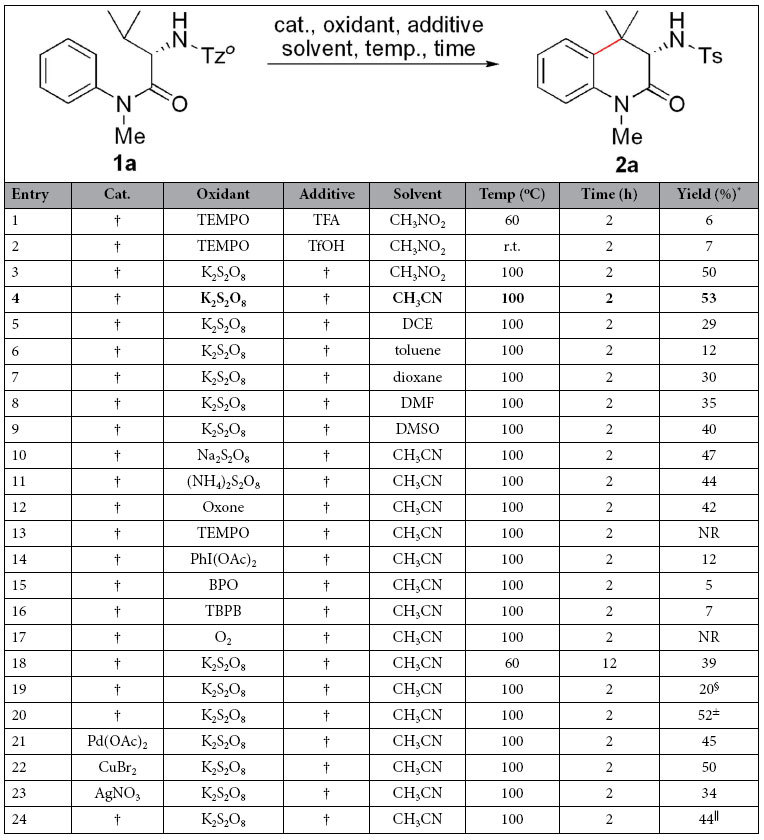
Development of a method for intramolecular coupling of aliphatic and aromatic C-H bonds.

Reaction condition: under nitrogen atmosphere, **1a** (0.1 mmol), catalyst (0.01 mmol), oxidant (0.2 mmol), additive (0.2 mmol for entry 1; 0.3 mmol for entry 2), solvent (2.0 mL), reaction temperature (r.t.−120 °C), reaction time (2 or 12 h) in a sealed Schlenk tube. ^*^Isolated yield. ^†^No addition of reagent. ^§^K_2_S_2_O_8_ (0.1 mmol). ^±^K_2_S_2_O_8_ (0.3 mmol). TEMPO = 2,2,6,6-tetramethylpiperidin-1-oxyl. DCE = 1,2-dichloroethane. DMA = N, N- Dimethylaniline. DMSO = dimethylsulfoxide. ^||^In air.

## References

[b1] Alberico D., Scott M. E. & Lautens M. Aryl-aryl bond formation by transition-metal-catalyzed direct arylation. Chem. Rev. 107, 174–238 (2007). 17212475 10.1021/cr0509760

[b2] Seregin I. V. & Gevorgyan V. Direct transition metal-catalyzed functionalization of heteroaromatic compounds. Chem. Soc. Rev. 36, 1173–1193 (2007). 17576484 10.1039/b606984n PMC3674790

[b3] Daugulis O., Do H.-Q. & Shabashov D. Palladium- and copper-catalyzed arylation of carbon-hydrogen bonds. Acc. Chem. Res. 42, 1074–1086 (2009). 19552413 10.1021/ar9000058 PMC2846291

[b4] Chen X., Engle K. M., Wang D.-H. & Yu J.-Q. Palladium(II)-catalyzed C-H activation/C-C cross-coupling reactions: versatility and practicality. Angew. Chem. Int. Ed. 48, 5094–5115 (2009). 10.1002/anie.200806273 PMC2722958 19557755

[b5] Colby D. A., Bergman R. G. & Ellman J. A. Rhodium-catalyzed C-C bond formation via heteroatom-directed C-H bond activation. Chem. Rev. 110, 624–655 (2010). 19438203 10.1021/cr900005n PMC2820156

[b6] Hartwig J. Borylation and silylation of C–H Bonds: A platform for diverse C–H bond functionalizations. Acc. Chem. Res. 45, 864–873 (2012). 22075137 10.1021/ar200206a

[b7] Neufeldt S. R. & Sanford M. S. Controlling site selectivity in palladium-catalyzed C–H bond functionalization. Acc. Chem. Res. 45, 936–946 (2012). 22554114 10.1021/ar300014f PMC3378812

[b8] Kakiuchi F. & Murai S. Catalytic C−H/olefin coupling. Acc. Chem. Res. 35, 826–834 (2002). 12379135 10.1021/ar960318p

[b9] Lyons T. W. & Sanford M. S. Palladium-catalyzed ligand-directed C-H functionalization reactions. Chem. Rev. 110, 1147–1169 (2010). 20078038 10.1021/cr900184e PMC2836499

[b10] Engle K. M., Mei T.-S., Wasa M. & Yu J.-Q. Weak coordination as a powerful means for developing broadly useful C–H functionalization reactions. Acc. Chem. Res. 45, 788–802 (2012). 22166158 10.1021/ar200185g PMC3334399

[b11] Colby D. A., Tsai A. S., Bergman R. G. & Ellman J. A. Rhodium catalyzed chelation-assisted C–H bond functionalization reactions. Acc. Chem. Res. 45, 814–825 (2012). 22148885 10.1021/ar200190g PMC3307943

[b12] Li C.-J. Cross-dehydrogenative coupling (CDC): Exploring C−C bond formations beyond functional group transformations. Acc. Chem. Res. 42, 335–344 (2009). 19220064 10.1021/ar800164n

[b13] Girard S. A., Knauber T. & Li C.-J. The Cross-Dehydrogenative Coupling of C_sp3_-H Bonds: A Versatile Strategy for C-C Bond Formations. Angew. Chem. Int. Ed. 52, 2–29 (2013). 10.1002/anie.201304268 24214829

[b14] Yeung C. S. & Dong V. M. Catalytic dehydrogenative cross-coupling: forming carbon-carbon bonds by oxidizing two carbon-hydrogen bonds. Chem. Rev. 111, 1215–1292 (2011). 21391561 10.1021/cr100280d

[b15] Sun C.-L., Li B.-J. & Shi Z.-J. Direct C-H transformation via iron catalysis. Chem. Rev. 111, 1293–1314 (2011). 21049955 10.1021/cr100198w

[b16] Liu C., Zhang H., Shi W. & Lei A. Bond formations between two nucleophiles: transition metal catalyzed oxidative cross-coupling reactions. Chem. Rev. 111, 1780–1824 (2011). 21344855 10.1021/cr100379j

[b17] Cho S. H., Kim J. Y., Kwak J. & Chang S. Recent advances in the transition metal-catalyzed twofold oxidative C-H bond activation strategy for C-C and C-N bond formation. Chem. Soc. Rev. 40, 5068–5083 (2011). 21643614 10.1039/c1cs15082k

[b18] Kozhushkov S. I. & Ackermann L. Ruthenium-catalyzed direct oxidative alkenylation of arenes through twofold C-H bond functionalization. Chem. Sci. 4, 886–896 (2013).

[b19] Newhouse T., Baran P. S. & Hoffmann R. W. The economies of synthesis. Chem. Soc. Rev. 38, 3010–3021 (2009). 19847337 10.1039/b821200g PMC2880393

[b20] Holland H. L. C–H activation. Curr. Opin. Chem. Biol. 3, 22–27 (1999). 10021398 10.1016/s1367-5931(99)80005-2

[b21] Krest C. M. *et al.* Reactive Intermediates in Cytochrome P450 Catalysis. J. Biol. Chem. 288, 17074–17081 (2013). 23632017 10.1074/jbc.R113.473108 PMC3682513

[b22] Yosca T. H. *et al.* Iron(IV)hydroxide pK_a_ and the Role of Thiolate Ligation in C–H Bond Activation by Cytochrome P450. Science 342, 825–829 (2013). 24233717 10.1126/science.1244373 PMC4299822

[b23] Hubbard B. K. & Walsh C. T. Vancomycin assembly: Nature’s way. Angew. Chem., Int. Ed. 42, 730–765 (2003). 10.1002/anie.200390202 12596194

[b24] Bugg T. D. H. Introduction to Enzyme and Coenzyme Chemistry. 2nd ed. Blackwell Publishing Ltd.: Oxford, U.K,., Chapter 6 (2004).

[b25] Silverman R. B. The Organic Chemistry of Enzyme-Catalyzed Reactions. 2nd ed.; Academic Press: San Diego, Chapters 3–6 (2002).

[b26] Denisov I. G., Makris T. M., Sligar S. G. & Schlichting I. Structure and chemistry of cytochrome P450. Chem. Rev. 105, 2253–2277 (2005). 15941214 10.1021/cr0307143

[b27] Grobe N. *et al.* Mammalian cytochrome P450 enzymes catalyze the phenol-coupling step in endogenous morphine biosynthesis. J. Biol. Chem. 284, 24425–24431 (2009). 19561069 10.1074/jbc.M109.011320 PMC2782035

[b28] Novo E. & Parola M. Redox mechanisms in hepatic chronic wound healing and fibrogenesis. Fibrogen. Tissue Rep. 1, 5 (2008). 10.1186/1755-1536-1-5 PMC2584013 19014652

[b29] Brady J. D., Sadler I. H. & Fry S. C. Pulcherosine, an oxidatively coupled trimer of tyrosine in plant cell walls: Its role in cross-link formation. Phytochemistry 47, 349–353 (1998). 9433813 10.1016/s0031-9422(97)00592-x

[b30] Fujimoto D., Horiuchi K. & Hirama M. Isotrityrosine, a new crosslinking amino-acid isolated from ascaris cuticle collagen. Biochem. Biophys. Res. Commun. 99, 637–643 (1981). 7236289 10.1016/0006-291x(81)91792-7

[b31] Rouau X. *et al.* Dehydrotrimer of ferulic acid from maize bran. Phytochemistry 63, 899–903 (2003). 12895537 10.1016/s0031-9422(03)00297-8

[b32] Ban F., Lundqvist M. J., Boyd R. J. & Eriksson L. A. Theoretical studies of the cross-linking mechanisms between cytosine and tyrosine. J. Am. Chem. Soc. 124, 2753–2761 (2002). 11890827 10.1021/ja011528m

[b33] Mitrasinovic P. M. Cross-linking between thymine and indolyl radical: Possible mechanisms for cross-linking of DNA and tryptophan-containing peptides. Bioconjugate Chem. 16, 588–597 (2005). 10.1021/bc0500490 15898726

[b34] Williamson N. R., Fineran P. C., Leeper F. J. & Salmond G. P. C. The biosynthesis and regulation of bacterial prodiginines. Nature Rev. Microbiol. 4, 887–899 (2006). 17109029 10.1038/nrmicro1531

[b35] Fürstner A. Chemistry and biology of roseophilin and the prodigiosin alkaloids: A survey of the last 2500 years. Angew. Chem., Int. Ed. 42, 3582–3603 (2003). 10.1002/anie.200300582 12916029

[b36] Withall D. M., Haynes S. W. & Challis G. L. Stereochemistry and Mechanism of Undecylprodigiosin Oxidative Carbocyclization to Streptorubin B by the Rieske Oxygenase RedG. J. Am. Chem. Soc. 137, 7889–7897 (2015). 26023709 10.1021/jacs.5b03994

[b37] Cerdeño A. M., Bibb M. J. & Challis G. L. Analysis of the prodiginine biosynthesis gene cluster of Streptomyces coelicolor A3(2): new mechanisms for chain initiation and termination in modular multienzymes. Chem. Biol. 8, 817–829 (2001). 11514230 10.1016/s1074-5521(01)00054-0

[b38] Haynes S. W., Sydor P. K., Corre C., Song L. & Challis G. L. Stereochemical elucidation of Streptorubin B. J. Am. Chem. Soc. 133, 1793–1798 (2011). 21166415 10.1021/ja109164t

[b39] Justicia J. *et al.* Bioinspired terpene synthesis: a radical approach. Chem. Soc. Rev. 40, 3525–3537 (2011). 21487573 10.1039/c0cs00220h

[b40] Kimball D. B. & Haley M. M. Triazenes: a versatile tool in organic synthesis. Angew. Chem. Int. Ed. 41, 3338–3351 (2002). 10.1002/1521-3773(20020916)41:18<3338::AID-ANIE3338>3.0.CO;2-7 12298030

[b41] Patrick T. B., Willaredt R. P. & DeGonia D. J. Synthesis of biaryls from aryltriazenes. J. Org. Chem. 50, 2232–2235 (1985).

[b42] Gross M. L., Blank D. H. & Welch W. M. The triazene moiety as a protecting group for aromatic amines. J. Org. Chem. 58, 2104–2109 (1993).

[b43] Braese S. The virtue of the multifunctional triazene linkers in the efficient solidphase synthesis of heterocycle libraries. Acc. Chem. Res. 37, 805–816 (2004). 15491127 10.1021/ar0200145

[b44] Nicolaou K. C. *et al.* New synthetic technology for the synthesis of aryl ethers: construction of C-O-D and D-O-E ring model systems of vancomycin. J. Am. Chem. Soc. 119, 3421–3422 (1997).

[b45] Voica A.-F., Mendoza A., Gutekunst W. R., Fraga J. O. & Baran P. S. Guided desaturation of unactivated aliphatics. Nat. Chem. 4, 629–635 (2012). 22824894 10.1038/nchem.1385 PMC3405363

[b46] Han G., McIntosh M. C. & Weinreb S. M. A convenient synthetic method for amide oxidation. Tetrahedron Lett. 35, 5813–5816 (1994).

[b47] Pines S. H., Purick R. M., Reamer R. A. & Gal G. New aspects of intramolecular hydrogen transfer in some *ortho*-substituted aryl radicals. J. Org. Chem. 43, 1337–1342 (1978).

[b48] Maegawa T. *et al.* Efficient and selective deuteration of phenylalanine derivatives catalyzed by Pd/C. Synlett 845–847 (2005).

