# Supplementary material for: Transition metal-free intramolecular regioselective couplings of aliphatic and aromatic C-H bonds
Source: Sci Rep. 2016 Jan 29;6:19931. doi: 10.1038/srep19931 (PMC4731807; doi:10.1038/srep19931)
Supplement: Supplementary Information [file srep19931-s1.pdf]

Supplementary Information

Transition metal-free intramolecular regioselective couplings of aliphatic and aromatic C-H bonds

Hua Tian,[1,2] Haijun Yang,[1] Changjin Zhu,[2] Hua Fu*[1,2]

[1]Key Laboratory of Bioorganic Phosphorus Chemistry and Chemical Biology (Ministry of Education), Department of Chemistry, Tsinghua University, Beijing 100084, P. R. China.

[2]Department of Applied Chemistry, Beijing Institute of Technology, Beijing 100081, P. R. China.

*To whom correspondence should be addressed. E-mail: fuhua@mail.tsinghua.edu.cn

Table of contents

General procedures S2

Experimental procedures and characterization data S2

General procedures for preparation and characterization data of **B** (**1**, **3** and **5**) S2

General procedures for preparation and characterization data of **E** (**2**, **4** and **6**) S27

Evidence for radical process S52

Deuterium-labelling study S53

Application of the methods S54

X-ray crystallographic data for **2k** S58

X-ray crystallographic data for **4b** S71

X-ray crystallographic data for **6m** S85

References S100

The NMR spectra of compounds S101

General procedures

All reactions were carried out under a nitrogen atmosphere with dry solvents using anhydrous conditions. Acetonitrile (MeCN) was purchased from Acros (99.9%, Extra Dry over Molecular Sieve, AcroSeal) and used with a Syringe under a nitrogen atmosphere balloon protection. Reagents were purchased and used without further purification. Reactions were monitored by thin layer chromatography (TLC) and the products were obtained by column chromatography on silica gel or by preparative thin layer chromatography (pTLC). Melting points were recorded on a Beijing Tech X-4 melting point apparatus. High-resolution mass spectra (HRMS) were recorded on Agilent LC-ESI-Q-TOF quadrupole-time of flight mass spectrometer. NMR spectra were recorded on JOEL JNM-ECA 600, JNM-ECS 400 and JNM-ECA 300 and proton and carbon magnetic resonance spectra (^{1}H NMR and ^{13}C NMR) were recorded using tetramethylsilane (TMS) in the solvent of $CDCl_3$ as the internal standard (^{1}H NMR: TMS at 0.00 ppm, $CHCl_3$ at 7.26 ppm; ^{13}C NMR: $CDCl_3$ at 77.16 ppm) The following abbreviations (or combinations thereof) were used to explain the multiplicities: s = singlet, d = doublet, t = triplet, q = quartet, m = multiplet, b = broad.

Experimental procedures and characterization data

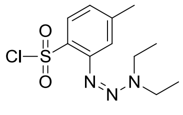

Tz^{o}Cl

Tz^{o}Cl was prepared according to the previous reference[1].

General procedures for preparation and characterization data of B (1, 3 and 5)

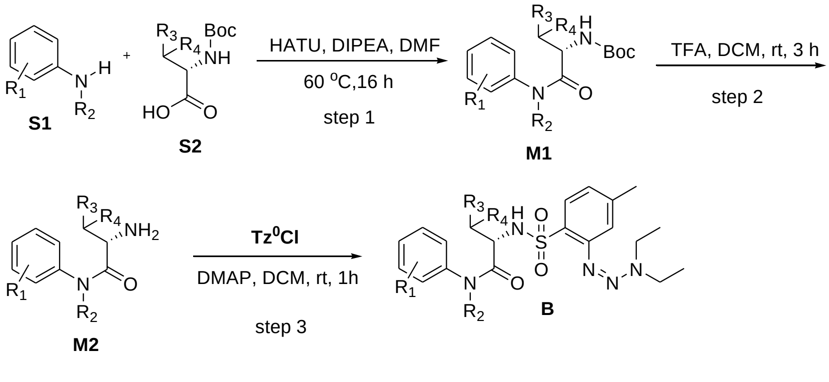

Step 1: To a solution of Boc-L-amino acid (**S2**, 1 mmol) in anhydrous DMF (5 mL) was added

HATU(1.5 mmol, 570 mg) and DIPEA (2 mmol, 250 uL), then the solution was stirred at room temperature under nitrogen atmosphere for 30 minutes. *N*-Substituted arylamines (**S1**, 1.5 mmol, 1.5 equiv) was added and the reaction mixture was allowed to proceed at 60 °C for 16 hours. The reaction mixture was cooled to room temperature, diluted with ethyl acetate (20 mL) and washed with 1N HCl (20 mL). The aqueous phase was extracted with ethyl acetate (20 mL), and the combined organic phase was washed with 1N HCl (2 × 20 mL), saturated aqueous $NaHCO_3$ (2 × 20 mL), brine (20 mL), dried over Na_2SO_4, concentrated and purified by silica gel chromatography to give intermediate **M1**.

Step 2: To a solution of **M1** in DCM was added TFA (TFA/DCM = 1:3) and the mixture was stirred at room temperature for 3 hours. TLC showed that no **M1** remained. The solvent was concentrated and the residue was diluted with DCM (10 mL). Saturated aqueous $NaHCO_3$ was added to alkalize and the two phases was separated. The organic phase was washed with brine (10 mL), dried over Na_2SO_4 and concentrated to give intermediate **M2** which was used for the next step directly.

Step 3: To a solution of **M2** (0.2 mmol) and DMAP (0.6 mmol, 73 mg) in DCM (2 mL) was added at 0 °C a solution of **Tz°Cl** (0.22 mmol, 64 mg) in DCM (1 mL) dropwise, then the reaction mixture was allowed to warm to room temperature and kept stirring for one hour. TLC showed that no **Tz°Cl** left. The resulting mixture was washed with H_2O (3 mL). The layers were then partitioned and the aqueous solution was further extracted with DCM (2 × 3 mL). The combined organic layers were dried over $MgSO_4$, filtrated, concentrated *in vacuo* and was purified by silica gel chromatography to give the desired products (**K**).

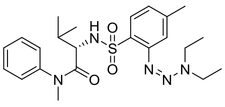

1a

(*S*,*Z*)-2-(2-(3,3-Diethyltriaz-1-enyl)-4-methylphenylsulfonamido)-*N*,3-dimethyl-*N*-Phenylbutanamide (1a): Synthesized from *N*-methylaniline and (S)-2-(*tert*-butoxycarbonylamino)-3-methylbutanoic acid (Boc-L-Valine) following the *general procedure*. Eluent: petroleum ether/ethyl acetate (5:1). Yield: 89 mg (97%). White solid, mp 125 - 127 °C. ESI-MS: $[M+Na]^+$ m/z 482.4.

^1H NMR ($CDCl_3$, 400 MHz) δ 7.74 (d, *J* = 8.2 Hz, 1H), 7.44 (s, 1H), 7.29 - 7.36 (m, 3H), 7.03 (d, *J* = 7.8 Hz, 1H), 6.75 (d, *J* = 6.4 Hz, 2H), 6.56 (d, *J* = 8.7 Hz, 1H), 4.03 - 4.12 (m, 1H), 3.77 - 3.90

(m, 4H), 2.98 (s, 3H), 2.41 (s, 3H), 1.71 - 1.78 (m, 1H), 1.38 - 1.44 (m, 6H), 0.84 (d, J = 6.8 Hz, 3H), 0.77 (d, J = 6.8 Hz, 3H).

^{13}C NMR ($CDCl_3$, 100 MHz) δ 170.8, 148.1, 143.8, 142.6, 130.6, 129.8, 128.3, 128.2, 127.5, 124.7, 118.2, 58.0, 49.8, 42.6, 37.6, 32.1, 21.8, 19.7, 16.9, 14.6, 11.5.

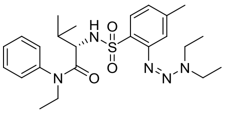

1b

(*S*,*Z*)-2-(2-(3,3-Diethyltriaz-1-enyl)-4-methylphenylsulfonamido)-*N*-ethyl-3-methyl-*N*-phenyl butanamide (1b): Synthesized from *N*-ethylaniline and (S)-2-(*tert*-butoxycarbonylamino)-3-methylbutanoic acid (Boc-L-Valine) following the *general procedure*. Eluent: petroleum ether/ethyl acetate (5:1). Yield: 93 mg (98%). White solid. ESI-MS: $[M+Na]^+$ m/z 496.7.

^{1}H NMR ($CDCl_3$, 400 MHz) δ 7.77 (d, J = 7.8 Hz, 1H), 7.46 (s, 1H), 7.32 - 7.35 (m, 3H), 7.05 (d, J = 7.8 Hz, 1H), 6.67 (br.s., 2H), 6.62 (d, J = 8.7 Hz, 1H), 4.06 - 4.16 (m, 1H), 3.75 - 3.91 (m, 4H), 3.69 - 3.73 (m, 1H), 3.07 - 3.16 (m, 1H), 2.40 (s, 3H), 1.69 - 1.77 (m, 1H), 1.37 - 1.44 (m, 6H), 0.84 (d, J = 6.8 Hz, 3H), 0.74 - 0.78 (m, 6H).

^{13}C NMR ($CDCl_3$, 100 MHz) δ 169.8, 148.0, 143.8, 140.7, 130.3, 129.6, 128.5, 128.3, 128.1, 124.5, 117.9, 58.1, 49.6, 44.6, 42.4, 31.7, 21.6, 19.6, 16.6, 14.4, 12.2, 11.4.

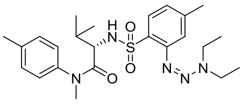

1c

(*S*,*Z*)-2-(2-(3,3-Diethyltriaz-1-enyl)-4-methylphenylsulfonamido)-*N*,3-dimethyl-*N*-p-tolylbuta namide (1c): Synthesized from *N*,4-dimethylaniline and (S)-2-(*tert*-butoxycarbonylamino)-3-methylbutanoic acid (Boc-L-Valine) following the *general procedure*. Eluent: petroleum ether/ethyl acetate (5:1). Yield: 90 mg (95%). White solid. ESI-MS: $[M+Na]^+$ m/z 496.6.

^{1}H NMR ($CDCl_3$, 400 MHz) δ 7.74 (d, J = 8.2 Hz, 1H), 7.44 (s, 1H), 7.11 (d, J = 7.8 Hz, 2H), 7.03 (d, J = 7.8 Hz, 1H), 6.56 - 6.61 (m, 3H), 4.05 - 4.14 (m, 1H), 3.76 - 3.91 (m, 4H), 2.95 (s, 3H), 2.41 (s, 3H), 2.35 (s, 3H), 1.71 - 1.79 (m, 1H), 1.37 - 1.44 (m, 6H), 0.84 (d, J = 6.8 Hz, 3H), 0.79 (d, J = 6.8 Hz, 3H).

^{13}C NMR ($CDCl_3$, 100 MHz) δ 170.8, 148.0, 143.7, 139.8, 138.0, 130.4, 130.3, 128.2, 127.1, 124.5, 118.0, 57.8, 49.7, 42.5, 37.5, 32.0, 21.7, 21.1, 19.6, 16.8, 14.5, 11.4.

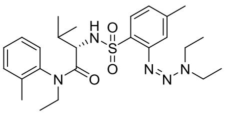

1d

(*S*,*Z*)-2-(2-(3,3-Diethyltriaz-1-enyl)-4-methylphenylsulfonamido)-*N*-ethyl-3-methyl-*N*-o-tolyl butanamide (1d): Synthesized from *N*-ethyl-2-methylaniline and (S)-2-(*tert*-butoxycarbonylamino)-3-methylbutanoic acid (Boc-L-Valine), in Step 1, EDCI (1.2 equiv) and HoBt (1 equiv) were instead of HATU and DIPEA as the condensation reagents, DCM was instead of DMF as the solvent, and the reaction was proceed at room temperature overnight. Then the post-treatment and the following steps following the *general procedure*. Eluent: petroleum ether/ethyl acetate (5:1). Yield: 90 mg (92%), two isomers. Yellow thick oil.. ESI-MS: $[M+Na]^+$ m/z 510.6.

^{1}H NMR ($CDCl_3$, 400 MHz) δ 7.82 (d, *J* = 7.8 Hz, 1H), 7.64 (d, *J* = 7.8 Hz, 0.5H), 7.47 (s, 1H), 7.39 (s, 0.5H), 7.19 - 7.27 (m, 3H), 7.05 - 7.15 (m, 3H), 6.92 (d, *J* = 7.8 Hz, 0.5H), 6.85 (d, *J* = 8.2 Hz, 0.5H), 6.67 (d, *J* = 8.2 Hz, 1H), 6.21 (d, *J* = 7.8 Hz, 1H), 4.04 - 4.15 (m, 3H), 3.69 - 3.88 (m, 6H), 3.17 - 3.22 (m, 0.5H), 2.55 - 2.63 (m, 1H), 2.39 (s, 3H), 2.36 (s, 1.5H), 2.12 (s, 3H), 1.84 (s, 1.5H), 1.36 - 1.47 (m, 10.5H), 0.99 (t, *J* = 6.8 Hz, 1.5H),0.72 - 0.80 (m, 9H), 0.65 (t, *J* = 6.8 Hz, 3H).

^{13}C NMR ($CDCl_3$, 100 MHz) δ 170.4, 170.0, 148.1, 147.5, 143.8, 143.2, 139.3, 138.8, 136.3, 135.1, 131.7, 131.6, 131.4, 130.1, 129.8, 129.2, 128.56, 128.49, 128.4, 127.0, 126.9, 126.7, 124.51, 124.48, 118.0, 117.9, 58.7, 58.1, 49.7, 49.6, 43.9, 43.4, 42.5, 42.4, 31.1, 29.6, 21.6, 19.9, 19.8, 17.4, 17.1, 15.8, 15.7, 14.5, 12.9, 11.43, 11.37.

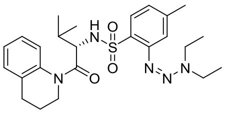

1e

(*S*,*Z*)-2-(3,3-Diethyltriaz-1-enyl)-*N*-(1-(3,4-dihydroquinolin-1(2H)-yl)-3-methyl-1-oxobutan-2-yl)-4-methylbenzenesulfonamide (1e): Synthesized from 1,2,3,4-tetrahydroquinoline and (S)-2-(*tert*-butoxycarbonylamino)-3-methylbutanoic acid (Boc-L-Valine) following the *general procedure*. Eluent: petroleum ether/ethyl acetate (6:1). Yield: 82 mg (85%). White solid.

^{1}H NMR ($CDCl_3$, 400 MHz) δ 7.74 (d, *J* = 7.6 Hz, 1H), 7.35 - 7.39 (m, 1H), 6.76 - 7.16 (m, 5H), 6.70 (d, *J* = 8.2 Hz, 1H), 4.27 - 4.63 (m, 1H), 3.80 - 4.04 (m, 5H), 3.16 - 3.55 (m, 1H), 2.49 - 2.52 (m, 2H), 2.28 - 2.36 (m, 3H), 1.52 - 1.91 (m, 3H), 1.38 - 1.42 (m, 6H), 0.80 - 1.10 (m, 6H).

^{13}C NMR ($CDCl_3$, 100 MHz) δ 170.8, 147.8, 143.7, 138.1, 134.0, 130.5, 128.6, 127.7, 126.6,

126.0, 124.8, 124.3, 118.2, 58.2, 49.8, 43.0, 42.5, 32.2, 26.5, 23.7, 21.7, 19.3, 16.9, 14.6, 11.5.

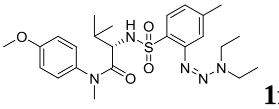

1f

(*S*,*Z*)-2-(2-(3,3-Diethyltriaz-1-enyl)-4-methylphenylsulfonamido)-*N*-(4-methoxyphenyl)-*N*,3-dimethylbutanamide (1f): Synthesized from 4-methoxy-*N*-methylaniline and (S)-2-(*tert*-butoxycarbonylamino)-3-methylbutanoic acid (Boc-L-Valine) following the *general procedure*. Eluent: petroleum ether/ethyl acetate (5:1). Yield: 90 mg (90%). Yellow thick oil.

^{1}H NMR ($CDCl_3$, 400 MHz) δ 7.74 (d, *J* = 7.8 Hz, 1H), 7.44 (s, 1H), 7.03 (d, *J* = 7.8 Hz, 1H), 6.81 - 6.83 (m, 2H), 6.64 - 6.66 (m, 2H), 6.56 (d, *J* = 8.7 Hz, 1H), 4.04 - 4.14 (m, 1H), 3.76 - 3.90 (m, 7H), 2.93 (s, 3H), 2.41 (s, 3H), 1.72 - 1.79 (m, 1H), 1.37 - 1.44 (m, 6H), 0.85 (d, *J* = 6.8 Hz, 3H), 0.80 (d, *J* = 6.8 Hz, 3H).

^{13}C NMR ($CDCl_3$, 100 MHz) δ 171.0, 158.9, 148.0, 143.8, 135.1, 130.4, 128.5, 128.2, 124.5, 118.1, 114.8, 57.8, 55.5, 49.8, 42.5, 37.7, 32.0, 21.7, 19.7, 16.8, 14.5, 11.4.

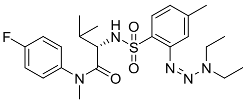

1g

(*S*,*Z*)-2-(2-(3,3-Diethyltriaz-1-enyl)-4-methylphenylsulfonamido)-*N*-(4-fluorophenyl)-*N*,3-dimethylbutanamide (1g): Synthesized from 4-fluoro-*N*-methylaniline and (S)-2-(*tert*-butoxycarbonylamino)-3-methylbutanoic acid (Boc-L-Valine) following the *general procedure*. Eluent: petroleum ether/ethyl acetate (5:1). Yield: 79 mg (83%). Light yellow solid, mp 184 - 186°C.

^{1}H NMR ($CDCl_3$, 400 MHz) δ 7.73 (d, *J* = 8.2 Hz, 1H), 7.44 (s, 1H), 7.00 - 7.05 (m, 3H), 6.71 - 6.76 (m, 2H), 6.55 (d, *J* = 8.7 Hz, 1H), 4.02 - 4.11 (m, 1H), 3.77 - 3.90 (m, 4H), 2.95 (s, 3H), 2.41 (s, 3H), 1.69 - 1.77 (m, 1H), 1.38 - 1.44 (m, 6H), 0.85 (d, *J* = 6.8 Hz, 3H), 0.79 (d, *J* = 6.8 Hz, 3H).

^{13}C NMR ($CDCl_3$, 100 MHz) δ 170.9, 161.8 (d, *J* = 249.2 Hz), 148.1, 143.9, 138.5 (d, *J* = 2.9 Hz), 130.5, 129.3 (d, *J* = 8.6 Hz), 128.1, 124.6, 118.2, 116.8 (d, *J* = 23.0 Hz), 57.9, 49.8, 42.6, 37.7, 32.2, 21.8, 19.7, 17.0, 14.6, 11.5.

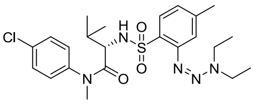

1h

(*S*,*Z*)-*N*-(4-Chlorophenyl)-2-(2-(3,3-diethyltriaz-1-enyl)-4-methylphenylsulfonamido)-*N*,3-di

methylbutanamide (1h): Synthesized from 4-chloro-*N*-methylaniline and (S)-2-(*tert*-butoxycarbonylamino)-3-methylbutanoic acid (Boc-L-Valine) following the *general procedure*. Eluent: petroleum ether/ethyl acetate (5:1). Yield: 94 mg (95%). Light yellow thick oil.

^{1}H NMR ($CDCl_3$, 400 MHz) δ 7.73 (d, *J* = 7.8 Hz, 1H), 7.44 (s, 1H), 7.31 (d, *J* = 8.7 Hz, 2H), 7.04 (d, *J* = 7.8 Hz, 1H), 6.69 (d, *J* = 7.8 Hz, 2H), 6.56 (d, *J* = 8.7 Hz, 1H), 4.04 - 4.11 (m, 1H), 3.78 - 3.90 (m, 4H), 2.95 (s, 3H), 2.41 (s, 3H), 1.71 - 1.75 (m, 1H), 1.38 - 1.43 (m, 6H), 0.84 (d, *J* = 6.8 Hz, 3H), 0.80 (d, *J* = 6.8 Hz, 3H).

^{13}C NMR ($CDCl_3$, 100 MHz) δ 170.6, 148.0, 143.9, 141.0, 133.9, 130.3, 130.0, 128.8, 128.0, 124.6, 118.1, 57.9, 49.8, 42.5, 37.5, 32.2, 21.7, 19.6, 17.0, 14.5, 11.4.

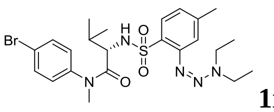

1i

(*S*,*Z*)-*N*-(4-Bromophenyl)-2-(2-(3,3-diethyltriaz-1-enyl)-4-methylphenylsulfonamido)-*N*,3-dimethylbutanamide (1i): Synthesized from 4-bromo-*N*-methylaniline and (S)-2-(*tert*-butoxycarbonylamino)-3-methylbutanoic acid (Boc-L-Valine) following the *general procedure*. Eluent: petroleum ether/ethyl acetate (5:1). Yield: 101 mg (94%). Light yellow thick oil.

^{1}H NMR ($CDCl_3$, 400 MHz) δ 7.73 (d, *J* = 7.8 Hz, 1H), 7.45 - 7.47 (m, 3H), 7.03 (d, *J* = 8.2 Hz, 1H), 6.63 (d, *J* = 8.2 Hz, 2H), 6.56 (d, *J* = 8.7 Hz, 1H), 4.02 - 4.12 (m, 1H), 3.76 - 3.90 (m, 4H), 2.95 (s, 3H), 2.41 (s, 3H), 1.70 - 1.77 (m, 1H), 1.37 - 1.43 (m, 6H), 0.84 (d, *J* = 6.8 Hz, 3H), 0.80 (d, *J* = 6.8 Hz, 3H).

^{13}C NMR ($CDCl_3$, 100 MHz) δ 170.6, 148.0, 143.9, 141.5, 132.9, 130.3, 129.1, 128.0, 124.6,121.9, 118.1, 57.9, 49.8, 42.5, 37.5, 32.2, 21.7, 19.6, 16.9, 14.5, 11.4.

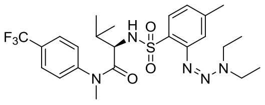

1j

(*R*,*Z*)-2-(2-(3,3-Diethyltriaz-1-enyl)-4-methylphenylsulfonamido)-*N*,3-dimethyl-*N*-(4-(trifluoromethyl)phenyl)butanamide (1j): Synthesized from 4-(trifluoromethyl)aniline and (R)-2-amino-3-methylbutanoic acid (D-Valine) as following steps:

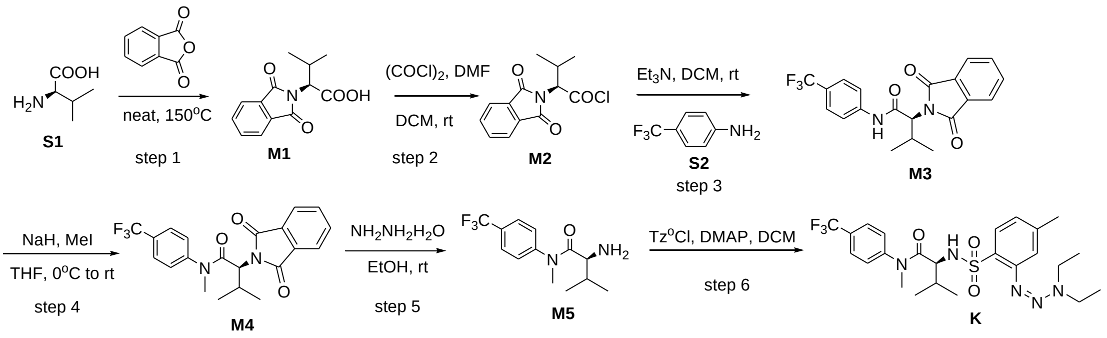

Step 1: (*R*)-2-amino-3-methylbutanoic acid (*D*-Valine, **S1**, 1 mmol) and isobenzofuran-1,3-dione (1 mmol) was mixed and heated to 150 °C in a open flask for one hour. The resulting solution was cooled down and dissolved in hot methaonl (5 mL). Water was added and white solid was precipitated, filtrated and dried to give intermediate **M1**.

Step 2 and **Step 3**: A solution of **M1** (1 mmol) in dry DCM (5 mL) was stirred at 0 °C and oxalyl chloride (1.5 mmol) was dropped into the mixture and stirred at room temperature for 2 hours. After the reaction, the solvent and excess oxalyl chloride was evaporated under reduced pressure. The residue was dissolved in dry DCM (5 mL) and a solution of 4-(trifluoromethyl)aniline (**S2**, 1 mmol) and triethylamine (2 mmol) in dry DCM (2 mL) was added to the mixture at 0 °C and then stirred at room temperature for 2 hours. After the reaction, the solvent was evaporated under reduced pressure, and the crude product was purified by chromatography on silica gel eluted with petroleum ether/ethyl acetate (V/V = 3:1) to offer intermediate **M3**.

Step 4: At 0°C, to a solution of **M3** (1 mmol) in anhydrous THF (5 mL) was added NaH (1.1 mmol) under nitrogen atmosphere, and then the solution was stirred at this temperature for 30 minutes. MeI (1.5 mmol) was added and the reaction mixture was allowed to warm to room temperature and stirred for another one hour. After the reaction completed the solvent was evaporated under reduced pressure, and the crude product was purified by chromatography on silica gel eluted with petroleum ether/ethyl acetate (V/V = 3:1) to offer intermediate **M4**.

Step 5: M4 (1 mmol) and hydrazine hydrate (3 mmol) were added to ethanol (15 mL), and the mixture was stirred at room temperature overnight. After the reaction was completed, the solvent was evaporated under reduced pressure, and the crude product was purified by chromatography on silica gel eluted with DCM/MeOH/Et_3N (V/V/V = 50:1:0.01) to obtain intermediate **M5**.

Step 6: At 0°C, to a solution of **M5** (0.2 mmol, 1 equiv) and DMAP (0.6 mmol, 73 mg) in DCM (2 mL) was added a solution of **Tz°Cl** (0.22 mmol, 64 mg) in DCM (1 mL) dropwise, then the

reaction mixture was allowed to warm to room temperature and kept stirring for one hour. TLC showed no **Tz°Cl** left. The resulting mixture was washed with H_2O (3 mL). The layers were then partitioned and the aqueous solution was further extracted with DCM (2 × 3 mL). The combined organic layers were dried over $MgSO_4$, filtrated, concentrated *in vacuo* and was purified by silica gel chromatography to give the desired products (**1j**). Eluent: petroleum ether/ethyl acetate (4:1). Yield: 99 mg (91%). Light yellow thick oil.

^{1}H NMR ($CDCl_3$, 400 MHz) δ 7.74 (d, *J* = 7.3 Hz, 1H), 7.62 (d, *J* = 7.3 Hz, 2H), 7.45 (s, 1H), 7.03 (d, *J* = 7.3 Hz, 1H), 6.94 (d, *J* = 6.8 Hz, 2H), 6.58 (d, *J* = 8.7 Hz, 1H), 4.02 - 4.05 (m, 1H), 3.80 - 3.90 (m, 4H), 3.01 (s, 3H), 2.41 (s, 3H), 1.71 - 1.77 (m, 1H), 1.37 - 1.43 (m, 6H), 0.84 (d, *J* = 5.5 Hz, 3H), 0.79 (d, *J* = 5.5 Hz, 3H).

^{13}C NMR ($CDCl_3$, 100 MHz) δ 170.7, 148.1, 145.8, 144.1, 130.5, 130.3(m), 128.0, 127.0, 126.3(m), 124.7, 123.7 (q, *J* = 273.2 Hz), 118.3, 58.2, 49.9, 42.6, 37.6, 32.4, 21.8, 19.6, 17.1, 14.5, 11.5.

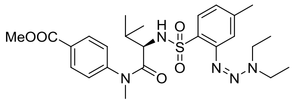

1k

(*R*,*Z*)-Methyl 4-(2-(2-(3,3-diethyltriaz-1-enyl)-4-methylphenylsulfonamido)-*N*,3-dimethyl-butanamido)benzoate (1k): Synthesized from methyl 4-aminobenzoate and (*R*)-2-amino-3-methylbutanoic acid (D-Valine) following the steps as **1j**. Eluent: petroleum ether/ethyl acetate (3:1). Yield: 78 mg (75%). Light yellow thick oil.

^{1}H NMR ($CDCl_3$, 400 MHz) δ 8.01 (d, *J* = 6.8 Hz, 2H), 7.74 (d, *J* = 7.3 Hz, 1H), 7.44 (s, 1H), 7.05 (d, *J* = 7.3 Hz, 1H), 6.84 (d, *J* = 6.8 Hz, 2H), 6.58 (d, *J* = 8.7 Hz, 1H), 4.02 - 4.08 (m, 1H), 3.95 (s, 3H), 3.78 - 3.90 (m, 4H), 3.01 (s, 3H), 2.42 (s, 3H), 1.71 - 1.74 (m, 1H), 1.37 - 1.43 (m, 6H), 0.84 (d, *J* = 5.5 Hz, 3H), 0.78 (d, *J* = 5.5 Hz, 3H).

^{13}C NMR ($CDCl_3$, 100 MHz) δ 170.6, 166.0, 148.0, 146.6, 143.9, 131.1, 130.4, 130.0, 128.0, 127.3, 124.7, 118.1, 58.1, 52.4, 49.8, 42.5, 37.4, 32.3, 21.7, 19.5, 17.0, 14.5, 11.4.

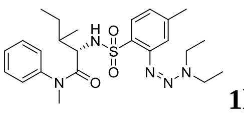

1l

(2*S*)-2-(2-((Z)-3,3-Diethyltriaz-1-enyl)-4-methylphenylsulfonamido)-*N*,3-dimethyl-*N*-phenylpentanamide (1l): Synthesized from *N*-methylaniline and (2S)-2-(*tert*-butoxycarbonylamino)-3-methylpentanoic acid (Boc-L-Isoleucine) following the

general procedure. Eluent: petroleum ether/ethyl acetate (5:1). Yield: 73 mg (77%). White solid.

^{1}H NMR ($CDCl_3$, 400 MHz) δ 7.74 (d, *J* = 8.2 Hz, 1H), 7.44 (s, 1H), 7.28 - 7.35 (m, 3H), 7.03 (d, *J* = 7.8 Hz, 1H), 6.76 (d, *J* = 6.8 Hz, 2H), 6.57 (d, *J* = 8.2 Hz, 1H), 4.02 - 4.11 (m, 1H), 3.78 - 3.91 (m, 4H), 2.99 (s, 3H), 2.40 (s, 3H), 1.49 - 1.57 (m, 2H), 1.30 - 1.43 (m, 6H), 1.00 - 1.09 (m, 1H), 0.73 - 0.77 (m, 6H).

^{13}C NMR ($CDCl_3$, 100 MHz) δ 170.8, 148.0, 143.7, 142.5, 130.6, 129.7, 128.1, 128.0, 127.5, 124.6, 118.1, 57.6, 49.7, 42.5, 39.2, 37.6, 23.6, 21.7, 15.8, 14.5, 11.5.

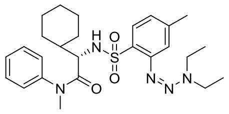

1m

(*S*,*Z*)-2-Cyclohexyl-2-(2-(3,3-diethyltriaz-1-enyl)-4-methylphenylsulfonamido)-*N*-methyl-*N*-phenylacetamide (1m): Synthesized from *N*-methylaniline and (S)-2-(*tert*-butoxycarbonylamino)-2-cyclohexylacetic acid (Boc-L-Cyclohexylglycine) following the *general procedure*. Eluent: petroleum ether/ethyl acetate (5:1). Yield: 84 mg (84%). White solid.

^{1}H NMR ($CDCl_3$, 400 MHz) δ 7.73 (d, *J* = 7.8 Hz, 1H), 7.44 (s, 1H), 7.28 - 7.34 (m, 3H), 7.03 (d, *J* = 8.2 Hz, 1H), 6.70 (d, *J* = 5.0 Hz, 2H), 6.55 (d, *J* = 8.2 Hz, 1H), 4.05 - 4.14 (m, 1H), 3.75 - 3.89 (m, 4H), 2.99 (s, 3H), 2.41 (s, 3H), 1.64 - 1.73 (m, 4H), 1.55 - 1.57 (m, 1H), 1.38 - 1.44 (m, 6H), 1.29 - 1.31 (m, 1H), 1.04 - 1.12 (m, 4H), 0.90 - 1.00 (m, 1H).

^{13}C NMR ($CDCl_3$, 100 MHz) δ 170.7, 148.0, 143.8, 142.5, 130.6, 129.7, 128.3, 128.0, 127.5, 124.7, 118.1, 57.8, 49.8, 42.5, 42.2, 37.6, 29.7, 27.6, 26.3, 26.2, 26.0, 21.7, 14.5, 11.4.

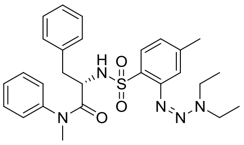

3a

(*S*,*Z*)-2-(2-(3,3-Diethyltriaz-1-enyl)-4-methylphenylsulfonamido)-*N*-methyl-*N*,3-diphenylpropanamide (3a): Synthesized from *N*-methylaniline and (S)-2-(*tert*-butoxycarbonylamino)-3-phenylpropanoic acid (Boc-L-Phenylalanine) following the *general procedure*. Eluent: petroleum ether/ethyl acetate (4:1). Yield: 99 mg (97%). White solid, mp 115 - 117 °C. ESI-MS: $[M+Na]^+$ m/z 530.4.

^{1}H NMR ($CDCl_3$, 400 MHz) δ 7.70 (d, *J* = 8.2 Hz, 1H), 7.41 (s, 1H), 7.16 - 7.23 (m, 4H), 7.12 (t, *J* = 7.3 Hz, 2H), 6.99 (d, *J* = 8.2 Hz, 1H), 6.90 - 6.92 (m, 2H), 6.57 (d, *J* = 8.7 Hz, 1H), 6.16 - 6.23

(m, 2H), 4.17 - 4.23 (m, 1H), 3.81 - 3.98 (m, 4H), 2.94 (s, 3H), 2.86 (dd, *J* =12.8 Hz, 9.2 Hz, 1H), 2.75 (dd, *J* = 13.2 Hz, 5.5 Hz, 1H), 2.39 (s, 3H), 1.38 - 1.43 (m, 6H).

^{13}C NMR ($CDCl_3$, 100 MHz) δ 170.4, 147.8, 143.9, 142.0, 136.3, 130.9, 129.8, 129.5, 128.4, 128.1, 127.9, 127.1, 126.9, 125.0, 118.2, 55.0, 49.9, 42.7, 41.1, 37.4, 21.8, 14.6, 11.4.

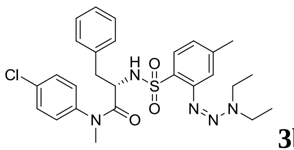

3b

(*S*,*Z*)-*N*-(4-Chlorophenyl)-2-(2-(3,3-diethyltriaz-1-enyl)-4-methylphenylsulfonamido)-*N*-methyl-3-phenylpropanamide (3b): Synthesized from 4-chloro-*N*-methylaniline and (S)-2-(*tert*-butoxycarbonylamino)-3-phenylpropanoic acid (Boc-L-Phenylalanine) following the *general procedure*. Eluent: petroleum ether/ethyl acetate (4:1). Yield: 95 mg (88%). Light yellow thick oil.

^{1}H NMR ($CDCl_3$, 400 MHz) δ 7.71 (d, *J* = 8.2 Hz, 1H), 7.43 (s, 1H), 7.17 - 7.22 (m, 3H), 7.05 (d, *J* = 8.7 Hz, 2H), 7.00 (d, *J* = 8.2 Hz, 1H), 6.92 - 6.94 (m, 2H), 6.58 (d, *J* = 8.7 Hz, 1H), 5.98 - 6.03 (m, 2H), 4.13 - 4.19 (m, 1H), 3.81 - 3.98 (m, 4H), 2.85 - 2.90 (m, 4H), 2.78 (dd, *J* = 12.8 Hz, 5.0 Hz, 1H), 2.39 (s, 3H), 1.38 - 1.43 (m, 6H).

^{13}C NMR ($CDCl_3$, 100 MHz) δ 170.3, 147.7, 144.0, 140.4, 136.1, 133.6, 130.7, 129.7, 129.5, 128.5, 128.4, 127.9, 126.9, 124.9, 118.1, 54.9, 49.9, 42.6, 41.1, 37.3, 21.7, 14.5, 11.3.

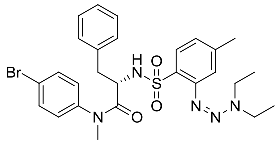

3c

(*S*,*Z*)-*N*-(4-Bromophenyl)-2-(2-(3,3-diethyltriaz-1-enyl)-4-methylphenylsulfonamido)-*N*-methyl-3-phenylpropanamide (3c): Synthesized from 4-bromo-*N*-methylaniline and (S)-2-(*tert*-butoxycarbonylamino)-3-phenylpropanoic acid (Boc-L-Phenylalanine) following the *general procedure*. Eluent: petroleum ether/ethyl acetate (4:1). Yield: 101 mg (86%). Yellow thick oil.

^{1}H NMR ($CDCl_3$, 400 MHz) δ 7.71 (d, *J* = 7.8 Hz, 1H), 7.42 (s, 1H), 7.18 - 7.21 (m, 5H), 7.00 (d, *J* = 7.8 Hz, 1H), 6.92 - 6.94 (m, 2H), 6.58 (d, *J* = 8.7 Hz, 1H), 5.92 - 5.96 (m, 2H), 4.13 - 4.19 (m, 1H), 3.81 - 3.95 (m, 4H), 2.84 - 2.90 (m, 4H), 2.78 (dd, *J* = 12.8 Hz, 5.0 Hz, 1H), 2.39 (s, 3H), 1.37 - 1.43 (m, 6H).

^{13}C NMR ($CDCl_3$, 100 MHz) δ 170.2, 147.7, 143.9, 140.9, 136.1, 132.5, 130.7, 129.6, 128.7, 128.4, 127.8, 126.9, 124.9, 121.6, 118.1, 54.8, 49.9, 42.6, 41.1, 37.2, 21.7, 14.5, 11.3.

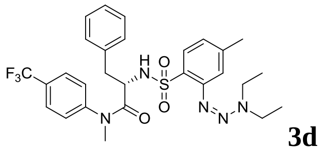

3d

(*S*,*Z*)-2-(2-(3,3-Diethyltriaz-1-enyl)-4-methylphenylsulfonamido)-*N*-methyl-3-phenyl-*N*-(4-(trifluoromethyl)phenyl)propanamide (3d): Synthesized from 4-(trifluoromethyl)aniline and (S)-2-amino-3-phenylpropanoic acid (L-Phenylalanine) following the steps as **1j**. Eluent: petroleum ether/ethyl acetate (3:1). Yield: 104 mg (90%). Yellow thick oil.

^{1}H NMR ($CDCl_3$, 400 MHz) δ 7.71 (d, *J* = 8.2 Hz, 1H), 7.44 (s, 1H), 7.34 (d, *J* = 8.7 Hz, 2H), 7.17 - 7.25 (m, 3H), 7.00 (d, *J* = 8.2 Hz, 1H), 6.90 (d, *J* = 6.8 Hz, 2H), 6.61 (d, *J* = 8.7 Hz, 1H), 6.23 - 6.26 (m, 2H), 4.15 - 4.21 (m, 1H), 3.83 - 3.96 (m, 4H), 2.85 - 2.96 (m, 4H), 2.80 (dd, *J* = 12.8 Hz, 5.0 Hz, 1H), 2.39 (s, 3H), 1.38 - 1.44 (m, 6H).

^{13}C NMR ($CDCl_3$, 100 MHz) δ 170.2, 147.8, 145.0, 144.1, 136.0, 130.6, 129.6, 128.5, 127.8, 127.4, 127.0, 126.42, 126.40, 124.9, 123.6 (q, *J* = 272.2 Hz), 118.2, 55.0, 49.9, 42.6, 41.1, 37.1, 21.6, 14.5, 11.3.

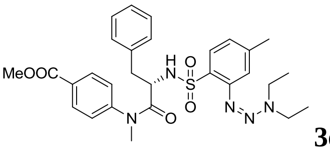

3e

(*S*,*Z*)-Methyl4-(2-(2-(3,3-diethyltriaz-1-enyl)-4-methylphenylsulfonamido)-*N*-methyl-3-phenylpropanamido)benzoate (3e): Synthesized from methyl 4-aminobenzoate and (S)-2-amino-3-phenylpropanoic acid (L-Phenylalanine) following the steps as **1j**. Eluent: petroleum ether/ethyl acetate (3:1). Yield: 88 mg (78%). Yellow thick oil.

^{1}H NMR ($CDCl_3$, 400 MHz) δ 7.76 (d, *J* = 8.4 Hz, 2H), 7.71 (d, *J* = 8.0 Hz, 1H), 7.44 (s, 1H), 7.18 - 7.25 (m, 3H), 7.01 (d, *J* = 8.0 Hz, 1H), 6.91 (d, *J* = 7.0 Hz, 2H), 6.61 (d, *J* = 8.4 Hz, 1H), 6.18 (d, *J* = 7.0 Hz, 2H), 4.16 - 4.22 (m, 1H), 3.80 - 3.98 (m, 7H), 2.86 - 2.97 (m, 4H), 2.79 (dd, *J* = 13.0 Hz, 5.0 Hz, 1H), 2.40 (s, 3H), 1.38 - 1.44 (m, 6H).

^{13}C NMR ($CDCl_3$, 100 MHz) δ 170.1, 166.0, 147.6, 145.6, 143.9, 135.9, 130.62, 130.57, 129.5, 129.2, 128.4, 127.8, 126.9, 126.8, 124.9, 118.1, 54.9, 52.2, 49.8, 42.5, 41.2, 37.0, 21.6, 14.4, 11.2.

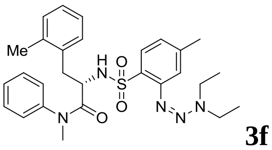

3f

(*S*,*Z*)-2-(2-(3,3-Diethyltriaz-1-enyl)-4-methylphenylsulfonamido)-*N*-methyl-*N*-phenyl-3-o-tolylpropanamide (3f): Synthesized from *N*-methylaniline and (S)-2-(*tert*-butoxycarbonylamino)-3-o-tolylpropanoic acid (Boc-L-Phe(2-Me)-OH) following the *general procedure*. Eluent: petroleum ether/ethyl acetate (4:1). Yield: 96 mg (92%). Yellow thick oil.

^{1}H NMR ($CDCl_3$, 400 MHz) δ 7.72 (d, *J* = 8.2 Hz, 1H), 7.42 (s, 1H), 7.18 (t, *J* = 7.3 Hz, 1H), 7.12 (dt, *J* = 7.3 Hz, 1.4 Hz, 1H), 7.02 - 7.07 (m, 3H), 6.96 - 6.99 (m, 2H), 6.93 (d, *J* = 6.9 Hz, 1H), 6.57 (d, *J* = 8.2 Hz, 1H), 5.81 - 6.18 (m, 2H), 4.23 - 4.29 (m, 1H), 3.84 - 4.00 (m, 4H), 2.93 (s, 3H), 2.77 - 2.87 (m, 2H), 2.38 (s, 3H), 1.75 (s, 3H), 1.45 (t, *J* = 7.3 Hz, 3H), 1.40 (d, *J* = 7.3 Hz, 3H).

^{13}C NMR ($CDCl_3$, 100 MHz) δ 170.6, 147.8, 143.9, 141.8, 137.6, 134.4, 131.1, 130.4, 129.3, 128.0, 127.7, 127.1, 127.0, 125.8, 125.0, 118.2, 52.8, 49.9, 42.7, 38.9, 37.1, 21.7, 18.6, 14.6, 11.4.

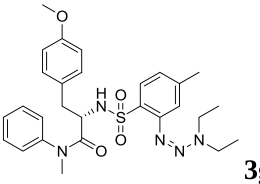

3g

(*S*,*Z*)-2-(2-(3,3-Diethyltriaz-1-enyl)-4-methylphenylsulfonamido)-3-(4-methoxyphenyl)-*N*-methyl-*N*-phenylpropanamide (3g): Synthesized from *N*-methylaniline and (S)-2-(*tert*-butoxycarbonylamino)-3-(4-methoxyphenyl)propanoic acid (Boc-L-Phe(4-OMe)-OH) following the *general procedure*. Eluent: petroleum ether/ethyl acetate (5:1). Yield: 94 mg (88%). Yellow thick oil.

^{1}H NMR ($CDCl_3$, 400 MHz) δ 7.69 (d, *J* = 7.8 Hz, 1H), 7.42 (s, 1H), 7.22 (t, *J* = 7.3 Hz, 1H), 7.15 (t, *J* = 7.8 Hz, 2H), 6.99 (dd, *J* = 7.8 Hz, 0.9 Hz, 1H), 6.83 (d, *J* = 8.2 Hz, 2H), 6.72 (d, *J* = 8.2 Hz, 2H), 6.55 (d, *J* = 8.2 Hz, 1H), 6.24 - 6.28 (m, 2H), 4.10 - 4.20 (m, 1H), 3.79 - 3.98 (m, 4H), 3.77 (s, 3H), 2.95 (s, 3H), 2.82 (dd, *J* = 13.0 Hz, 9.2 Hz, 1H), 2.69 (dd, *J* = 13.0 Hz, 5.5 Hz, 1H), 2.39 (s, 3H), 1.37 - 1.43 (m, 6H).

^{13}C NMR ($CDCl_3$, 100 MHz) δ 170.4, 158.6, 147.7, 143.7, 142.0, 130.7, 130.6, 129.4, 128.2, 127.9, 127.8, 127.0, 124.8, 118.0, 113.7, 55.2, 54.9, 49.8, 42.6, 40.1, 37.3, 21.7, 14.5, 11.3.

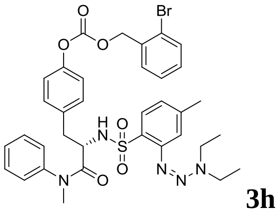

3h

(*S*,*Z*)-2-Bromobenzyl 4-(2-(2-(3,3-diethyltriaz-1-enyl)-4-methylphenylsulfonamido)-3-(methyl(phenyl)amino)-3-oxopropyl)phenyl carbonate (3h): Synthesized from *N*-methylaniline and (S)-3-(4-((2-bromobenzyloxy)carbonyloxy)phenyl)-2-(*tert*-butoxycarbonylamino)propanoic acid (BOC-L-Tyr(2-Br-Z)-OH) following the *general procedure*. Eluent: petroleum ether/ethyl acetate (5:1). Yield: 134 mg (91%). Yellow thick oil.

^{1}H NMR ($CDCl_3$, 400 MHz) δ 7.72 (d, *J* = 8.2 Hz, 1H), 7.58 (d, *J* = 7.9 Hz, 1H), 7.50 (d, *J* = 7.6 Hz, 1H), 7.43 (s, 1H), 7.34 (t, *J* = 7.6 Hz, 1H), 7.11 - 7.24 (m, 4H), 6.98 - 7.03 (m, 3H), 6.92 (d, *J* = 7.9 Hz, 2H), 6.61 (d, *J* = 8.6 Hz, 1H), 6.18 - 6.24 (m, 2H), 5.36 (s, 2H), 4.18 - 4.25 (m, 1H), 3.77 - 4.03 (m, 4H), 2.94 (s, 3H), 2.83 - 2.88 (m, 1H), 2.77 (dd, *J* = 12.9 Hz, 5.5 Hz, 1H), 2.39 (s, 3H), 1.36 - 1.44 (m, 6H).

^{13}C NMR ($CDCl_3$, 100 MHz) δ 170.1, 153.5, 150.1, 147.7, 143.9, 141.7, 134.21, 134.15, 132.9, 130.7, 130.6, 130.2, 130.0, 129.5, 127.9, 127.8, 127.7, 126.9, 124.9, 123.4, 121.0, 118.1, 69.6, 54.7, 49.8, 42.6, 40.5, 37.2, 21.7, 14.5, 11.3.

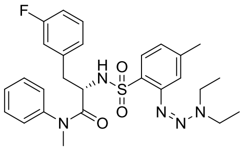

3i

(*S*,*Z*)-2-(2-(3,3-Diethyltriaz-1-enyl)-4-methylphenylsulfonamido)-3-(3-fluorophenyl)-*N*-methyl-*N*-phenylpropanamide (3i): Synthesized from *N*-methylaniline and (S)-2-(*tert*-butoxycarbonylamino)-3-(3-fluorophenyl)propanoic acid (Boc-L-Phe(3-F)-OH) following the *general procedure*. Eluent: petroleum ether/ethyl acetate (4:1). Yield: 89 mg (85%). Yellow thick oil.

^{1}H NMR ($CDCl_3$, 400 MHz) δ 7.70 (d, *J* = 8.2 Hz, 1H), 7.42 (s, 1H), 7.23 - 7.27 (m, 1H), 7.11 - 7.20 (m, 3H), 6.99 (d, *J* = 8.2 Hz, 1H), 6.90 (dt, *J* = 8.2 Hz, 2.3 Hz, 1H), 6.70 (d, *J* = 7.8 Hz, 1H), 6.59 (d, *J* = 8.2 Hz, 2H), 6.31 - 6.33 (m, 2H), 4.19 - 4.24 (m, 1H), 3.78 - 4.00 (m, 4H), 2.96 (s, 3H), 2.85 (dd, *J* = 13.2 Hz, 8.2 Hz, 1H), 2.73 (dd, *J* = 13.0 Hz, 5.5 Hz, 1H), 2.39 (s, 3H), 1.37 - 1.43 (m, 6H).

^{13}C NMR ($CDCl_3$, 100 MHz) δ 170.1, 162.7 (d, *J* = 245.4 Hz), 147.7, 143.9, 141.9, 138.7 (d, *J* = 7.7 Hz), 130.5, 129.7 (d, *J* = 7.7 Hz), 129.6, 128.04, 127.97, 126.9, 125.3 (d, *J* = 1.9 Hz), 124.9, 118.1, 116.5 (d, *J* = 21.1 Hz), 113.7 (d, *J* = 21.1 Hz), 54.6, 49.9, 42.6, 40.6, 37.3, 21.7, 14.5, 11.3.

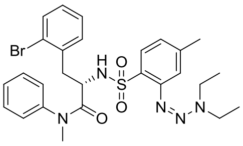

3j

(*S*,*Z*)-3-(2-Bromophenyl)-2-(2-(3,3-diethyltriaz-1-enyl)-4-methylphenylsulfonamido)-*N*-methyl-*N*-phenylpropanamide (3j): Synthesized from *N*-methylaniline and (S)-3-(2-bromophenyl)-2-(*tert*-butoxycarbonylamino)propanoic acid (Boc-L-Phe(2-Br)-OH) following the *general procedure*. Eluent: petroleum ether/ethyl acetate (4:1). Yield: 107 mg (91%). Yellow thick oil.

^{1}H NMR ($CDCl_3$, 400 MHz) δ 7.73 (d, *J* = 7.8 Hz, 1H), 7.40 (s, 1H), 7.36 (d, *J* = 8.2 Hz, 1H), 7.22 (t, *J* = 7.3 Hz, 1H), 7.12 - 7.16 (m, 3H), 7.04 - 7.08 (m, 2H), 6.98 (dd, *J* = 8.2 Hz, 0.9 Hz, 1H), 6.51 (d, *J* = 8.7 Hz, 1H), 6.21 - 6.28 (m, 2H), 4.42 - 4.48 (m, 1H), 3.81 - 4.00 (m, 4H), 2.93 - 2.98 (m, 4H), 2.86 (dd, *J* = 12.8 Hz, 9.2 Hz, 1H), 2.38 (s, 3H), 1.37 - 1.44 (m, 6H).

^{13}C NMR ($CDCl_3$, 100 MHz) δ 170.3, 147.8, 143.9, 141.8, 135.8, 132.7, 132.0, 130.9, 129.6, 128.6, 128.3, 127.9, 127.2, 126.9, 125.6, 125.0, 118.1, 52.4, 49.9, 42.7, 40.8, 37.3, 21.7, 14.6, 11.4.

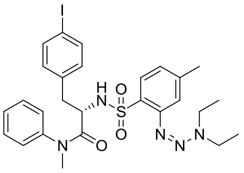

3k

(*S*,*Z*)-2-(2-(3,3-Diethyltriaz-1-enyl)-4-methylphenylsulfonamido)-3-(4-iodophenyl)-*N*-methyl-*N*-phenylpropanamide (3k): Synthesized from *N*-methylaniline and (S)-2-(*tert*-butoxycarbonylamino)-3-(4-iodophenyl)propanoic acid (Boc-L-Phe(4-I)-OH) following the *general procedure*. Eluent: petroleum ether/ethyl acetate (4:1). Yield: 118 mg (93%). White solid.

^{1}H NMR ($CDCl_3$, 400 MHz) δ 7.67 (d, *J* = 8.2 Hz, 1H), 7.47 (d, *J* = 8.2 Hz, 2H), 7.41 (s, 1H), 7.17 - 7.27 (m, 3H), 6.99 (d, *J* = 7.8 Hz, 1H), 6.63 (d, *J* = 8.2 Hz, 2H), 6.55 (d, *J* = 8.2 Hz, 1H), 6.34 - 6.36 (m, 2H), 4.17 - 4.23 (m, 1H), 3.77 - 3.97 (m, 4H), 2.96 (s, 3H), 2.79 (dd, *J* = 13.0 Hz, 8.0 Hz, 1H), 2.67 (dd, *J* = 13.2 Hz, 5.5 Hz, 1H), 2.40 (s, 3H), 1.37 - 1.41 (m, 6H).

^{13}C NMR ($CDCl_3$, 100 MHz) δ 170.1, 147.7, 143.9, 141.9, 137.3, 135.8, 131.6, 130.5, 129.6, 128.0, 127.9, 126.9, 124.9, 118.1, 92.2, 54.6, 49.9, 42.6, 40.4, 37.4, 21.8, 14.5, 11.3.

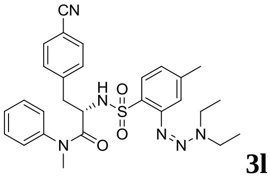

3l

(*S*,*Z*)-3-(4-Cyanophenyl)-2-(2-(3,3-diethyltriaz-1-enyl)-4-methylphenylsulfonamido)-*N*-methyl-*N*-phenylpropanamide (3l): Synthesized from *N*-methylaniline and (S)-2-(*tert*-butoxycarbonylamino)-3-(4-cyanophenyl)propanoic acid (Boc-L-Phe(4-CN)-OH) following the *general procedure*. Eluent: petroleum ether/ethyl acetate (4:1). Yield: 94 mg (88%). Yellow thick oil.

^{1}H NMR ($CDCl_3$, 400 MHz) δ 7.64 (d, *J* = 8.2 Hz, 1H), 7.45 (d, *J* = 8.2 Hz, 2H), 7.42 (s, 1H), 7.28 - 7.32 (m, 1H), 7.25 (t, *J* = 7.8 Hz, 2H), 7.00 - 7.02 (m, 3H), 6.59 (d, *J* = 7.8 Hz, 1H), 6.46 (d, *J* = 7.3 Hz, 2H), 4.19 - 4.25 (m, 1H), 3.93 - 4.00 (m, 1H), 3.87 (q, *J* = 6.8 Hz, 2H), 3.75 - 3.82 (m, 1H), 2.99 (s, 3H), 2.89 (dd, *J* = 13.2 Hz, 6.8 Hz, 1H), 2.75 (dd, *J* = 13.2 Hz, 6.4 Hz, 1H), 2.41 (s, 3H), 1.39 (t, *J* = 6.8 Hz, 6H).

^{13}C NMR ($CDCl_3$, 100 MHz) δ 169.7, 147.7, 144.0, 141.8, 141.7, 131.9, 130.3, 130.0, 129.8, 128.2, 128.0, 126.8, 124.8, 118.8, 118.1, 110.5, 54.3, 49.8, 42.5, 40.6, 37.3, 21.7, 14.4, 11.2.

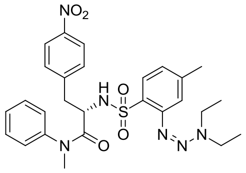

3m

(*S*,*Z*)-2-(2-(3,3-Diethyltriaz-1-enyl)-4-methylphenylsulfonamido)-*N*-methyl-3-(4-nitrophenyl)-*N*-phenylpropanamide (3m): Synthesized from *N*-methylaniline and (S)-2-(tert-butoxycarbonylamino)-3-(4-nitrophenyl)propanoic acid (Boc-L-Phe(4-NO_2)-OH) following the *general procedure*. Eluent: petroleum ether/ethyl acetate (3:1). Yield: 106 mg (96%). Yellow thick oil.

^{1}H NMR ($CDCl_3$, 400 MHz) δ 8.00 (d, *J* = 8.7 Hz, 2H), 7.62 (d, *J* = 7.8 Hz, 1H), 7.41 (s, 1H), 7.32 (t, *J* = 7.3 Hz, 1H), 7.26 (t, *J* = 7.8 Hz, 2H), 7.05 (d, *J* = 8.7 Hz, 2H), 6.99 (d, *J* = 7.8 Hz, 1H), 6.60 (d, *J* = 7.8 Hz, 1H), 6.53 (d, *J* = 7.3 Hz, 2H), 4.22 - 4.28 (m, 1H), 3.94 - 4.01 (m, 1H), 3.87 (q, *J* = 7.3 Hz, 2H), 3.75 - 3.82 (m, 1H), 3.01 (s, 3H), 2.94 (dd, *J* = 13.5 Hz, 6.8 Hz, 1H), 2.78 (dd, *J* =

13.2 Hz, 6.8 Hz, 1H), 2.40 (s, 3H), 1.39 (t, J = 7.3 Hz, 6H).

^{13}C NMR ($CDCl_3$, 100 MHz) δ 169.7, 147.8, 146.8, 144.1, 144.0, 141.8, 130.4, 130.0, 129.9, 128.4, 128.0, 126.9, 124.8, 123.3, 118.1, 54.4, 49.9, 42.5, 40.3, 37.4, 21.7, 14.5, 11.3.

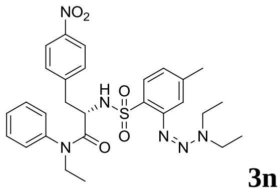

3n

(*S*,*Z*)-2-(2-(3,3-Diethyltriaz-1-enyl)-4-methylphenylsulfonamido)-*N*-ethyl-3-(4-nitrophenyl)-*N*-phenylpropanamide (3n): Synthesized from *N*-ethylaniline and (S)-2-(tert-butoxycarbonylamino)-3-(4-nitrophenyl)propanoic acid (Boc-L-Phe(4-NO_2)-OH) following the *general procedure*. Eluent: petroleum ether/ethyl acetate (3:1). Yield: 105 mg (92%). Yellow thick oil.

^{1}H NMR ($CDCl_3$, 400 MHz) δ 8.02 (d, J = 8.7 Hz, 2H), 7.65 (d, J = 8.2 Hz, 1H), 7.43 (s, 1H), 7.34 (t, J = 7.3 Hz, 1H), 7.27 (t, J = 6.8 Hz, 2H), 7.06 (d, J = 8.2 Hz, 2H), 7.00 (d, J = 8.2 Hz, 1H), 6.64 (d, J = 7.8 Hz, 1H), 6.47 (d, J = 7.3 Hz, 2H), 4.08 - 4.13 (m, 1H), 3.98 - 4.03 (m, 1H), 3.87 (q, J = 7.3 Hz, 2H), 3.70 - 3.78 (m, 2H), 3.16 - 3.24 (m, 1H), 2.93 (dd, J = 13.2 Hz, 6.4 Hz, 1H), 2.78 (dd, J = 13.5 Hz, 6.8 Hz, 1H), 2.40 (s, 3H), 1.39 (t, J = 6.8 Hz, 6H), 0.83 (t, J = 6.8 Hz, 3H).

^{13}C NMR ($CDCl_3$, 100 MHz) δ 169.1, 147.9, 146.9, 144.2, 144.1, 140.2, 130.5, 130.0, 129.8, 128.6, 128.3, 128.1, 124.8, 123.3, 118.0, 54.6, 49.9, 44.8, 42.6, 40.4, 21.7, 14.5, 12.4, 11.3.

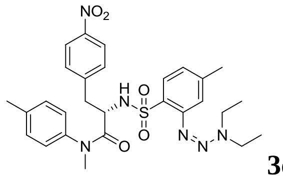

3o

(*S*,*Z*)-2-(2-(3,3-Diethyltriaz-1-enyl)-4-methylphenylsulfonamido)-*N*-methyl-3-(4-nitrophenyl)-*N*-*p*-tolylpropanamide (3o): Synthesized from *N*,4-dimethylaniline and (S)-2-(tert-butoxycarbonylamino)-3-(4-nitrophenyl)propanoic acid (Boc-L-Phe(4-NO_2)-OH) following the *general procedure*. Eluent: petroleum ether/ethyl acetate (3:1). Yield: 102 mg (90%). Yellow thick oil.

^{1}H NMR ($CDCl_3$, 400 MHz) δ 8.01 (d, J = 8.7 Hz, 2H), 7.63 (d, J = 8.2 Hz, 1H), 7.41 (s, 1H), 7.08 (d, J = 8.7 Hz, 2H), 7.05 (d, J = 8.2 Hz, 2H), 6.99 (d, J = 8.2 Hz, 1H), 6.60 (d, J = 7.8 Hz, 1H), 6.40 (d, J = 8.2 Hz, 2H), 4.24 - 4.29 (m, 1H), 3.94 - 4.01 (m, 1H), 3.86 (q, J = 7.3 Hz, 2H),

3.72 - 3.80 (m, 1H), 2.98 (s, 3H), 2.93 (dd, J = 13.5 Hz, 6.4 Hz, 1H), 2.78 (dd, J = 13.2 Hz, 6.4 Hz, 1H), 2.40 (s, 3H), 2.36 (s, 3H), 1.39 (t, J = 7.3 Hz, 6H).

^{13}C NMR ($CDCl_3$, 100 MHz) δ 169.7, 147.8, 146.8, 144.11, 144.05, 139.2, 138.4, 130.44, 130.41, 130.0, 128.0, 126.6, 124.7, 123.2, 118.1, 54.3, 49.8, 42.5, 40.3, 37.4, 21.6, 21.0, 14.4, 11.3.

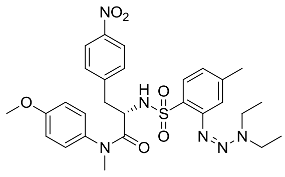

3p

(*S*,*Z*)-2-(2-(3,3-Diethyltriaz-1-enyl)-4-methylphenylsulfonamido)-*N*-(4-methoxyphenyl)-*N*-methyl-3-(4-nitrophenyl)propanamide (3p): Synthesized from 4-methoxy-*N*-methylaniline and (S)-2-(*tert*-butoxycarbonylamino)-3-(4-nitrophenyl)propanoic acid (Boc-L-Phe(4-NO_2)-OH) following the *general procedure*. Eluent: petroleum ether/ethyl acetate (3:1). Yield: 106 mg (91%). Yellow thick oil.

^{1}H NMR ($CDCl_3$, 400 MHz) δ 8.00 (d, J = 8.7 Hz, 2H), 7.61 (d, J = 8.2 Hz, 1H), 7.39 (s, 1H), 7.07 (d, J = 8.2 Hz, 2H), 6.97 (d, J = 7.8 Hz, 1H), 6.73 (d, J = 8.7 Hz, 2H), 6.56 (d, J = 7.8 Hz, 1H), 6.41 (d, J = 8.7 Hz, 2H), 4.20 - 4.25 (m, 1H), 3.91 - 4.00 (m, 1H), 3.85 (q, J = 7.3 Hz, 2H), 3.80 (s, 3H), 3.70 - 3.78 (m, 1H), 2.95 (s, 3H), 2.92 (dd, J = 13.2 Hz, 6.8 Hz, 1H), 2.77 (dd, J = 13.2 Hz, 6.8 Hz, 1H), 2.38 (s, 3H), 1.37 (t, J = 7.3 Hz, 6H).

^{13}C NMR ($CDCl_3$, 100 MHz) δ 169.9, 159.1, 147.8, 146.8, 144.13, 144.07, 134.4, 130.5, 130.0, 128.04, 128.03, 124.7, 123.2, 118.1, 114.9, 55.5, 54.3, 49.8, 42.5, 40.3, 37.6, 21.6, 14.4, 11.2.

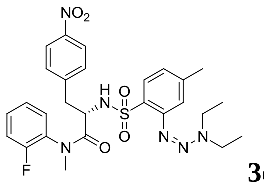

3q

(*S*,*Z*)-2-(2-(3,3-Diethyltriaz-1-enyl)-4-methylphenylsulfonamido)-*N*-(2-fluorophenyl)-*N*-methyl-3-(4-nitrophenyl)propanamide (3q): Synthesized from 2-fluoro-*N*-methylaniline and (S)-2-(*tert*-butoxycarbonylamino)-3-(4-nitrophenyl)propanoic acid (Boc-L-Phe(4-NO_2)-OH) following the *general procedure*. Eluent: petroleum ether/ethyl acetate (3:1). Yield: 95 mg (86%), containing 30% isomer. Yellow thick oil.

^{1}H NMR ($CDCl_3$, 400 MHz) δ 8.00 (d, J = 8.7 Hz, 2H), 7.63 (d, J = 7.8 Hz, 1H), 7.36 - 7.42 (m, 2H), 7.08 - 7.16 (m, 4H), 6.98 (d, J = 8.2 Hz, 1H), 6.65 (t, J = 7.8 Hz, 1H), 6.54 (d, J = 7.8 Hz,

1H), 4.24 - 4.29 (m, 1H), 3.91 - 3.98 (m, 1H), 3.86 (q, *J* = 7.3 Hz, 2H), 3.69 - 3.76 (m, 1H), 3.01 (s, 3H), 2.91 (dd, *J* = 13.5 Hz, 5.5 Hz, 1H), 2.70 (dd, *J* = 13.7 Hz, 6.8 Hz, 1H), 2.40 (s, 3H), 1.34 - 1.40 (m, 6H).

^{13}C NMR ($CDCl_3$, 100 MHz) δ 169.9, 157.8 (d, *J* = 250.2 Hz), 147.9, 147.0, 144.3, 143.8, 130.7 (d, *J* = 7.7 Hz), 130.6, 130.4, 130.0, 128.1, 125.7 (d, *J* = 3.8 Hz), 124.8, 123.41, 123.36, 118.2, 117.1 (d, *J* = 19.2 Hz), 54.4, 49.9, 42.6, 39.5, 36.5, 21.8, 14.5, 11.3.

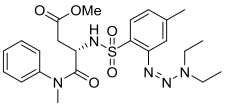

5a

(*S*,*Z*)-Methyl 3-(2-(3,3-diethyltriaz-1-enyl)-4-methylphenylsulfonamido)-4-(methyl(phenyl) amino)-4-oxobutanoate (5a): Synthesized from *N*-methylaniline and (S)-2-(*tert*-butoxycarbonylamino)-4-methoxy-4-oxobutanoic acid (Boc-L-Asp(OMe)-OH) following the *general procedure*. Eluent: petroleum ether/ethyl acetate (2:1). Yield: 92 mg (94%). Yellow thick oil.

^{1}H NMR ($CDCl_3$, 400 MHz) δ 7.66 (d, *J* = 7.8 Hz, 1H), 7.43 (s, 1H), 7.33 - 7.40 (m, 3H), 7.05 (d, *J* = 6.8 Hz, 2H), 6.98 (d, *J* = 7.8 Hz, 1H), 6.57 (d, *J* = 9.2 Hz, 1H), 4.37 - 4.43 (m, 1H), 3.77 - 3.95 (m, 4H), 3.54 (s, 3H), 3.10 (s, 3H), 2.62 (dd, *J* = 15.3 Hz, 7.3 Hz, 1H), 2.39 (s, 3H), 2.35 (dd, *J* = 15.6 Hz, 6.4 Hz, 1H), 1.38 (d, *J* = 7.3 Hz, 6H).

^{13}C NMR ($CDCl_3$, 100 MHz) δ 170.0, 169.4, 147.5, 143.9, 142.1, 130.4, 129.8, 128.2, 127.7, 127.1, 124.9, 118.0, 51.7, 50.5, 49.8, 42.5, 37.8, 37.6, 21.6, 14.4, 11.1.

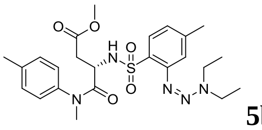

5b

(*S*,*Z*)-Methyl 3-(2-(3,3-diethyltriaz-1-enyl)-4-methylphenylsulfonamido)-4-(methyl (*p*-tolyl)amino)-4-oxobutanoate (5b): Synthesized from *N*,4-dimethylaniline and (S)-2-(*tert*-butoxycarbonylamino)-4-methoxy-4-oxobutanoic acid (Boc-L-Asp(OMe)-OH) following the *general procedure*. Eluent: petroleum ether/ethyl acetate (2:1). Yield: 92 mg (92%). Yellow thick oil.

^{1}H NMR ($CDCl_3$, 400 MHz) δ 7.67 (d, *J* = 7.8 Hz, 1H), 7.42 (s, 1H), 7.16 (d, *J* = 7.8 Hz, 2H), 6.98 (d, *J* = 7.8 Hz, 1H), 6.90 (d, *J* = 7.8 Hz, 2H), 6.57 (d, *J* = 9.2 Hz, 1H), 4.38 - 4.43 (m, 1H), 3.79 - 3.96 (m, 4H), 3.55 (s, 3H), 3.06 (s, 3H), 2.60 (dd, *J* = 15.1 Hz, 6.8 Hz, 1H), 2.40 (s, 3H),

2.32 - 2.38 (m, 4H), 1.39 (d, *J* = 6.8 Hz, 6H).

^{13}C NMR ($CDCl_3$, 100 MHz) δ 170.1, 169.5, 147.7, 143.9, 139.6, 138.2, 130.5, 130.4, 127.8, 126.9, 124.9, 118.1, 51.8, 50.6, 49.9, 42.6, 38.1, 37.7, 21.7, 21.1, 14.5, 11.2.

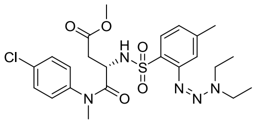

5c

(*S*,*Z*)-Methyl 4-((4-chlorophenyl)(methyl)amino)-3-(2-(3,3-diethyltriaz-1-enyl)-4-methyl phenylsulfonamido)-4-oxobutanoate (5c): Synthesized from 4-chloro-*N*-methylaniline and (S)-2-(*tert*-butoxycarbonylamino)-4-methoxy-4-oxobutanoic acid (Boc-L-Asp(OMe)-OH) following the *general procedure*. Eluent: petroleum ether/ethyl acetate (2:1). Yield: 93 mg (89%). Yellow thick oil.

^{1}H NMR ($CDCl_3$, 400 MHz) δ 7.64 (d, *J* = 7.8 Hz, 1H), 7.42 (s, 1H), 7.34 (d, *J* = 8.2 Hz, 2H), 7.06 (d, *J* = 7.8 Hz, 2H), 6.99 (d, *J* = 8.2 Hz, 1H), 6.47 (d, *J* = 9.2 Hz, 1H), 4.37 - 4.43 (m, 1H), 3.77 - 3.89 (m, 4H), 3.55 (s, 3H), 3.10 (s, 3H), 2.66 (dd, *J* = 15.6 Hz, 7.8 Hz, 1H), 2.40 (s, 3H), 2.35 (dd, *J* = 15.8 Hz, 5.5 Hz, 1H), 1.34 - 1.41 (m, 6H). ^{13}C NMR ($CDCl_3$, 100 MHz) δ 170.1, 169.4, 147.5, 144.1, 140.8, 134.0, 130.6, 129.9, 128.7, 127.6, 125.0, 118.1, 51.8, 50.3, 49.9, 42.6, 37.6, 37.5, 21.7, 14.4, 11.1.

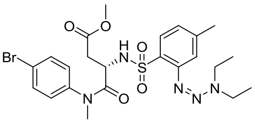

5d

(*S*,*Z*)-Methyl 4-((4-bromophenyl)(methyl)amino)-3-(2-(3,3-diethyltriaz-1-enyl)-4-methylphenylsulfonamido)-4-oxobutanoate (5d): Synthesized from 4-bromo-*N*-methylaniline and (S)-2-(*tert*-butoxycarbonylamino)-4-methoxy-4-oxobutanoic acid (Boc-L-Asp(OMe)-OH) following the *general procedure*. Eluent: petroleum ether/ethyl acetate (2:1). Yield: 98 mg (86%). Yellow thick oil.

^{1}H NMR ($CDCl_3$, 400 MHz) δ 7.64 (d, *J* = 8.2 Hz, 1H), 7.49 (d, *J* = 7.8 Hz, 2H), 7.42 (s, 1H), 6.96 - 7.02 (m, 3H), 6.47 (d, *J* = 9.2 Hz, 1H), 4.37 - 4.43 (m, 1H), 3.78 - 3.89 (m, 4H), 3.55 (s, 3H), 3.10 (s, 3H), 2.66 (dd, *J* = 15.6 Hz, 7.8 Hz, 1H), 2.40 (s, 3H), 2.35 (dd, *J* = 15.8 Hz, 5.5 Hz, 1H), 1.33 - 1.41 (m, 6H).

^{13}C NMR ($CDCl_3$, 100 MHz) δ 170.1, 169.3, 147.5, 144.1, 141.3, 132.9, 130.5, 129.0, 127.5,

125.0, 122.0,118.1, 51.8, 50.3, 49.9, 42.6, 37.6, 37.5, 21.7, 14.4, 11.1.

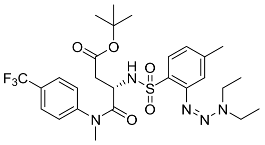

5e

(*S*,*Z*)-*tert*-Butyl 3-(2-(3,3-diethyltriaz-1-enyl)-4-methylphenylsulfonamido)-4-(methyl (4-(trifluoromethyl)phenyl)amino)-4-oxobutanoate (5e): Synthesized from *N*-methyl-4-(trifluoromethyl)aniline (**S2**) and (S)-2-(((9H-fluoren-9-yl)methoxy)carbonylamino)-4-*tert*-butoxy-4-oxobutanoic acid (Fmoc-L-Asp(OtBu)-OH) (**S1**) following the next steps:

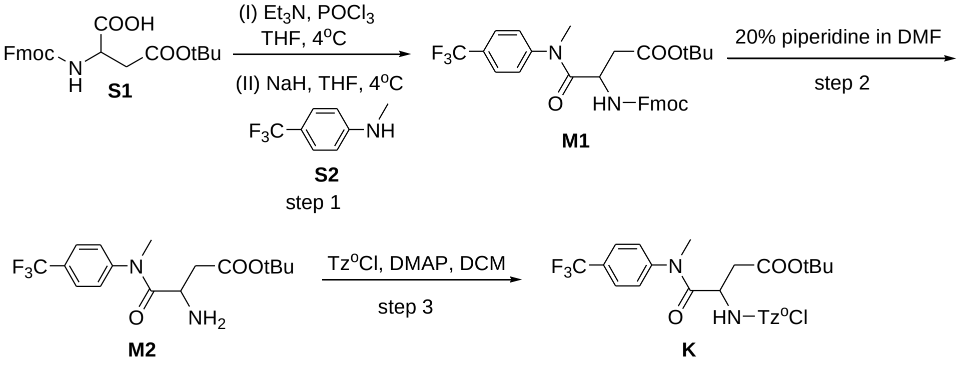

M1 and **M2** were prepared according to the previuos reference[2], and step 3 was following the *general procedure*. Eluent: petroleum ether/ethyl acetate (2:1). Yield: 114 mg (95%). Yellow thick oil.

^{1}H NMR ($CDCl_3$, 600 MHz) δ 7.57 - 7.72 (m, 3H), 7.44 (s, 1H), 7.14 - 7.32 (m, 2H), 6.99 (d, *J* = 8.2 Hz, 1H), 6.51 - 6.59 (m, 1H), 4.33 - 4.48 (m, 1H), 3.78 - 3.89 (m, 4H), 3.05 - 3.32 (m, 3H), 2.51 - 2.64 (m, 1H), 2.40 (s, 3H), 2.25 - 2.36 (m, 1H), 1.28 - 1.41 (m, 15H).

^{13}C NMR ($CDCl_3$, 150 MHz) δ 169.7, 168.8, 147.6, 145.6, 144.1, 130.6, 127.8, 127.5, 126.8, 126.5, 125.0, 123.8 (q, *J* =271.7 Hz), 118.2, 81.3, 50.8, 49.9, 42.6, 39.4, 37.6, 27.9, 21.7, 14.4, 11.2.

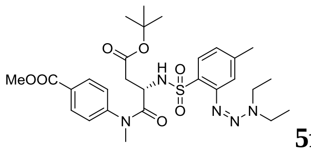

5f

(*S*,*Z*)-Methyl 4-(4-*tert*-butoxy-2-(2-(3,3-diethyltriaz-1-enyl)-4-methylphenylsulfonamido)-*N*-methyl-4-oxobutanamido)benzoate (5f): Synthesized from methyl 4-(methylamino)benzoate (**S2**) and (S)-2-(((9H-fluoren-9-yl)methoxy)carbonylamino)-4-*tert*-butoxy-4-oxobutanoic acid (Fmoc-L-Asp(OtBu)-OH) (**S1**) following the steps as **5e**. Eluent: petroleum ether/ethyl acetate

(2:1). Yield: 94 mg (80%). Yellow thick oil.

^{1}H NMR ($CDCl_3$, 400 MHz) δ 7.97 - 8.06 (m, 2H), 7.64 - 7.72 (m, 1H), 7.43 (s, 1H), 7.04 - 7.14 (m, 2H), 7.00 (d, *J* = 8.2 Hz, 1H), 6.56 (d, *J* = 9.2 Hz, 1H), 4.38 - 4.48 (m, 1H), 3.93 (s, 3H), 3.78 - 3.89 (m, 4H), 3.05 - 3.17 (m, 3H), 2.51 - 2.63 (m, 1H), 2.41 (s, 3H), 2.25 - 2.37 (m, 1H), 1.32 - 1.41 (m, 15H).

^{13}C NMR ($CDCl_3$, 100 MHz) δ 169.8, 168.9, 166.2, 147.6, 146.6, 144.1, 131.0, 130.5, 129.5, 127.6, 126.9, 125.0, 118.1, 81.3, 52.3, 50.9, 49.9, 42.6, 39.5, 37.6, 28.0, 21.7, 14.5, 11.2.

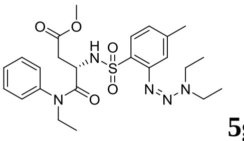

5g

(*S*,*Z*)-Methyl 3-(2-(3,3-diethyltriaz-1-enyl)-4-methylphenylsulfonamido)-4-(ethyl (phenyl)amino)-4-oxobutanoate (5g): Synthesized from *N*-ethylaniline and (S)-2-(*tert*-butoxycarbonylamino)-4-methoxy-4-oxobutanoic acid (Boc-L-Asp(OMe)-OH) following the *general procedure*. Eluent: petroleum ether/ethyl acetate (2:1). Yield: 96 mg (95%). Yellow thick oil.

^{1}H NMR ($CDCl_3$, 400 MHz) δ 7.69 (d, *J* = 8.2 Hz, 1H), 7.43 (s, 1H), 7.35 - 7.41 (m, 3H), 6.96 - 7.01 (m, 3H), 6.63 (d, *J* = 9.2 Hz, 1H), 4.26 - 4.31 (m, 1H), 3.78 - 3.99 (m, 4H), 3.67 - 3.77 (m, 1H), 3.55 (s, 3H), 3.31 - 3.39 (m, 1H), 2.57 (dd, *J* = 15.1 Hz, 6.4 Hz, 1H), 2.39 (s, 3H), 2.35 (dd, *J* = 15.1 Hz, 6.8 Hz, 1H), 1.39 (t, *J* = 7.3 Hz, 6H), 0.91 (t, *J* = 7.3 Hz, 3H).

^{13}C NMR ($CDCl_3$, 100 MHz) δ 170.0, 168.8, 147.7, 144.0, 140.5, 130.5, 129.8, 128.5, 128.3, 127.9, 124.9, 118.0, 51.7, 50.9, 49.9, 44.8, 42.6, 38.2, 21.7, 14.5, 12.4, 11.2.

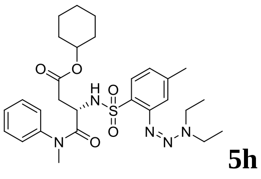

5h

(*S*,*Z*)-Cyclohexyl 3-(2-(3,3-diethyltriaz-1-enyl)-4-methylphenylsulfonamido)-4-(methyl (phenyl)amino)-4-oxobutanoate (5h): Synthesized from *N*-methylaniline and (S)-2-(*tert*-butoxycarbonylamino)-4-(cyclohexyloxy)-4-oxobutanoic acid (Boc-L-Asp(OcHex)-OH) following the *general procedure*. Eluent: petroleum ether/ethyl acetate (2:1). Yield: 101 mg (91%). Yellow thick oil.

^{1}H NMR ($CDCl_3$, 400 MHz) δ 7.67 (d, *J* = 7.8 Hz, 1H), 7.43 (s, 1H), 7.32 - 7.40 (m, 3H), 7.06 (d,

J = 6.8 Hz, 2H), 6.98 (d, J = 7.8 Hz, 1H), 6.58 (d, J = 9.2 Hz, 1H), 4.60 - 4.67 (m, 1H), 4.38 - 4.45 (m, 1H), 3.78 - 3.94 (m, 4H), 3.07 (s, 3H), 2.57 (dd, J = 15.6 Hz, 6.4 Hz, 1H), 2.40 (s, 3H), 2.31 (dd, J = 15.6 Hz, 6.8 Hz, 1H), 1.60 - 1.79 (m, 4H), 1.45 - 1.52 (m, 1H), 1.39 (t, J = 6.8 Hz, 6H), 1.17 - 1.34 (m, 5H).

^{13}C NMR ($CDCl_3$, 100 MHz) δ 169.5, 169.0, 147.6, 143.8, 142.2, 130.5, 129.8, 128.2, 127.7, 127.2, 124.9, 118.1, 73.1, 50.8, 49.9, 42.6, 38.6, 37.6, 31.43, 31.37, 25.3, 23.6, 21.7, 14.5, 11.2.

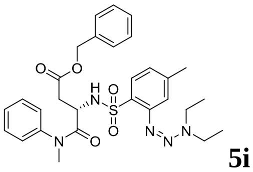

5i

(*S*,*Z*)-Benzyl 3-(2-(3,3-diethyltriaz-1-enyl)-4-methylphenylsulfonamido)-4-(methyl (phenyl)amino)-4-oxobutanoate (5i): Synthesized from *N*-methylaniline and (S)-4-(benzyloxy)-2-(*tert*-butoxycarbonylamino)-4-oxobutanoic acid (Boc-L-Asp(OBz)-OH) following the *general procedure*. Eluent: petroleum ether/ethyl acetate (3:1). Yield: 97 mg (86%). Yellow thick oil.

^{1}H NMR ($CDCl_3$, 400 MHz) δ 7.65 (d, J = 7.8 Hz, 1H), 7.41 (s, 1H), 7.27 - 7.36 (m, 6H), 7.25 (d, J = 7.3 Hz, 2H), 6.95 - 7.00 (m, 3H),, 6.57 (d, J = 9.2 Hz, 1H), 4.95 - 5.03 (m, 2H), 4.42 - 4.47 (m, 1H), 3.75 - 3.94 (m, 4H), 3.08 (s, 3H), 2.67 (dd, J = 15.6 Hz, 6.8 Hz, 1H), 2.36 - 2.41 (m, 4H),1.36 - 1.40 (m, 6H).

^{13}C NMR ($CDCl_3$, 100 MHz) δ 169.4, 147.6, 144.0, 142.1, 135.6, 130.5, 129.8, 128.5, 128.3, 128.2, 127.8, 127.2, 125.0, 118.1, 66.5, 50.7, 49.9, 42.6, 38.1, 37.6, 21.7, 14.5, 11.2.

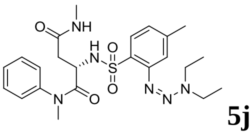

5j

(*S*,*Z*)-2-(2-(3,3-Diethyltriaz-1-enyl)-4-methylphenylsulfonamido)-*N*^{1},*N*^{4}-dimethyl-*N*^{1}-phenylsuccinamide (5j): Synthesized from *N*-methylaniline and (S)-2-(*tert*-butoxycarbonylamino)-4-(methylamino)-4-oxobutanoic acid (which was synthesized from Boc-L-Asp(OMe)-OH and Methylamine solution according to the previous method shown in the reference[3] following the *general procedure*. Eluent: petroleum ether/ethyl acetate (1:1). Yield: 88 mg (87%). Yellow thick oil.

^{1}H NMR ($CDCl_3$, 400 MHz) δ 7.66 (d, J = 7.8 Hz, 1H), 7.44 (s, 1H), 7.30 - 7.34 (m, 3H), 7.02 (d,

J = 8.2 Hz, 1H), 6.79 - 6.86 (m, 2H), 6.69 (d, J = 7.8 Hz, 1H), 6.13 - 6.18 (m, 1H), 4.13 - 4.18 (m, 1H), 4.01 - 4.08 (m, 1H), 3.88 (q, J = 6.8 Hz, 2H), 3.71 - 3.80 (m, 1H), 3.06 (s, 3H), 2.69 (d, J = 5.0 Hz, 3H), 2.40 - 2.48 (m, 4H), 2.30 (dd, J = 15.1 Hz, 6.8 Hz, 1H), 1.38 - 1.41 (m, 6H).

^{13}C NMR ($CDCl_3$, 100 MHz) δ 169.5, 169.3, 148.0, 144.4, 141.9, 129.9, 129.2, 128.6, 128.4, 127.2, 124.9, 118.1, 51.0, 49.9, 42.7, 40.7, 37.7, 26.3, 21.8, 14.5, 11.3.

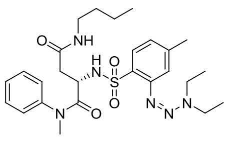

5k

(*S*,*Z*)-*N*[4]-Butyl-2-(2-(3,3-diethyltriaz-1-enyl)-4-methylphenylsulfonamido)-*N*[1]-methyl-*N*[1]-phenylsuccinamide (5k): Synthesized from *N*-methylaniline and (S)-2-(*tert*-butoxycarbonylamino)-4-(butylamino)-4-oxobutanoic acid (which was synthesized from Boc-L-Asp(OMe)-OH and Butylamine according to the previous method shown in the reference (*32*) following the *general procedure*. Eluent: petroleum ether/ethyl acetate (1:1). Yield: 90 mg (85%). Yellow thick oil.

^{1}H NMR ($CDCl_3$, 400 MHz) δ 7.66 (d, J = 7.8 Hz, 1H), 7.45 (s, 1H), 7.30 - 7.34 (m, 3H), 7.02 (d, J = 7.8 Hz, 1H), 6.78 - 6.86 (m, 2H), 6.71 (d, J = 7.3 Hz, 1H), 6.11 - 6.16 (m, 1H), 4.14 - 4.18 (m, 1H), 4.01 - 4.10 (m, 1H), 3.87 (q, J = 6.8 Hz, 2H), 3.71 - 3.80 (m, 1H), 3.09 - 3.21 (m, 2H), 3.05 (s, 3H), 2.39 - 2.47 (m, 4H), 2.30 (dd, J = 15.1 Hz, 6.8 Hz, 1H), 1.36 - 1.45 (m, 8H), 1.24 - 1.33 (m, 2H), 0.89 (t, J = 7.3 Hz, 3H).

^{13}C NMR ($CDCl_3$, 100 MHz) δ 169.4, 168.5, 147.9, 144.3, 141.9, 129.9, 129.2, 128.5, 128.3, 127.2, 124.9, 118.1, 51.0, 49.9, 42.6, 40.8, 39.3, 37.6, 31.3, 21.7, 20.0, 14.5, 13.7, 11.3.

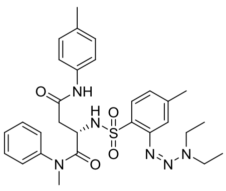

5l

(*S*,*Z*)-2-(2-(3,3-Diethyltriaz-1-enyl)-4-methylphenylsulfonamido)-*N*[1]-methyl-*N*[1]-phenyl-*N*[4]-p-tolylsuccinamide (5l): Synthesized following the *general procedure* except the intermediate **M1** which was synthesized following the next steps:

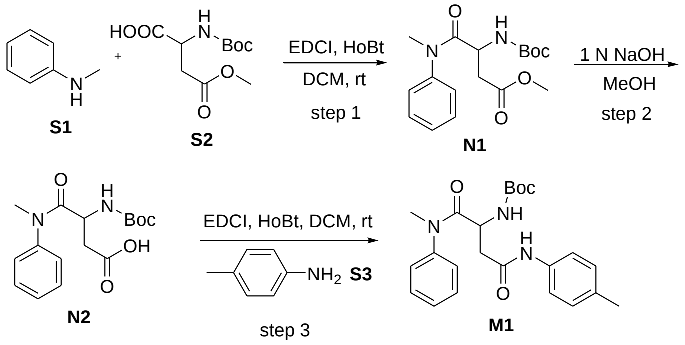

Step 1: To a solution of Boc-L-Asp(OMe)-OH (**S2**, 1mmol, 1 equiv) in anhydrous DCM (5 mL) was added *N*-methylaniline (**S1**, 1mmol) and HoBt (1 mmol), then the solution was cooled in a ice bath under nitrogen atmosphere. EDCI (1.2 mmol) was added portion wise and then the reaction mixture was allowed to warm to room temperature and stirred overnight. After the reaction completed, it was diluted with ethyl acetate (20 mL) and washed with 1N HCl (20 mL). The aqueous phase was extracted with ethyl acetate (20 mL), and the combined organic phase was washed with 1N HCl (2 × 20 mL), saturated aqueous $NaHCO_3$ (2 × 20 mL), brine (20 mL), dried over Na_2SO_4, concentrated and purified by silica gel chromatography to give intermediate **N1**.

Step 2: To a solution of **N1** in MeOH (5 mL) was added 1 N NaOH aqueous solution (5 mL) and the mixture was stirred at room temperature for 3 h. TLC showed that no **N1** left. The solvent was concentrated and the residue was diluted with water (10 mL). 1 N HCl aqueous solution was added to make the pH to 5 and it was extracted with ethyl acetate (3 × 10 mL). The combined organic phase was washed with brine (10 mL), dried over Na_2SO_4 and concentrated to give intermediate **N2** which was used for the next step directly.

Step 3: To a solution of intermediate **N2** (1 equiv) in anhydrous DCM (5 mL) was added *p*-toluidine (**S3**, 1 equiv) and HOBt (1 equiv), then the solution was cooled in a ice bath under nitrogen atmosphere. EDCI (1.2 equiv) was added and then the reaction mixture was allowed to warm to room temperature and stirred overnight. After the reaction completed, it was diluted with ethyl acetate (20 mL) and washed with 1N HCl (20 mL). The aqueous phase was extracted with ethyl acetate (20 mL), and the combined organic phase was washed with 1N HCl (2 × 20 mL), saturated aqueous $NaHCO_3$ (2 × 20 mL), brine (20 mL), dried over Na_2SO_4, concentrated and purified by silica gel chromatography to give intermediate **M1**.

Then the intermediate **M1** was proceed following the *general procedure* to give **5l.**

Eluent: petroleum ether/ethyl acetate (1:1). Yield: 101 mg (90%). Yellow thick oil.

^{1}H NMR ($CDCl_3$, 400 MHz) δ 8.21 (br.s. 1H), 7.68 (d, J = 7.8 Hz, 1H), 7.43 (s, 1H), 7.34 (d, J = 7.8 Hz, 2H), 7.24 - 7.29 (m, 3H), 7.04 (d, J = 8.2 Hz, 2H), 7.00 (d, J = 8.2 Hz, 1H), 6.79 - 6.86 (m, 3H), 4.26 - 4.33 (m, 1H), 3.93 - 4.01 (m, 1H), 3.81 (q, J = 7.3 Hz, 2H), 3.65 - 3.71 (m, 1H), 3.05 (s, 3H), 2.62 (dd, J = 14.9 Hz, 5.5 Hz, 1H), 2.50 (dd, J = 15.1 Hz, 6.4 Hz, 1H), 2.40 (s, 3H), 2.27 (s, 3H), 1.29 - 1.37 (m, 6H).

^{13}C NMR ($CDCl_3$, 100 MHz) δ 169.5, 166.7, 147.9, 144.4, 141.8, 135.4, 133.5, 129.9, 129.2, 128.4, 128.3, 127.1, 124.9, 120.0, 118.1, 51.0, 49.8, 42.6, 41.7, 37.7, 21.7, 20.8, 14. 4, 11.2.

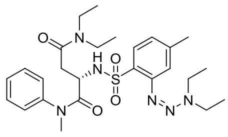

5m

(*S*,*Z*)-2-(2-(3,3-Diethyltriaz-1-enyl)-4-methylphenylsulfonamido)-*N*^{4},*N*^{4}-diethyl-*N*^{1}-methyl-*N*^{1}-phenylsuccinamide (5m): Synthesized from *N*-methylaniline (**S1)**, Boc-L-Asp(OMe)-OH (**S2**) and diethylamine (**S3**) following the method as **5l**. Eluent: petroleum ether/ethyl acetate (1:1). Yield: 98 mg (93%). Yellow thick oil.

^{1}H NMR ($CDCl_3$, 400 MHz) δ 7.64 (d, J = 7.8 Hz, 1H), 7.41 (s, 1H), 7.29 - 7.35 (m, 3H), 7.12 - 7.18 (m, 2H), 6.96 (d, J = 7.8 Hz, 1H), 6.35 (d, J = 8.2 Hz, 1H), 4.36 - 4.41 (m, 1H), 3.78 - 3.92 (m, 4H), 3.27 - 3.36 (m, 2H), 3.18 (s, 3H), 2.94 - 3.07 (m, 2H), 2.69 - 2.74 (m, 1H), 2.39 (s, 3H), 2.28 - 2.34 (m, 1H), 1.30 - 1.40 (m, 6H), 1.02 (t, J = 6.8 Hz, 3H), 0.88 (t, J = 6.8 Hz, 3H).

^{13}C NMR ($CDCl_3$, 100 MHz) δ 169.9, 168.2, 147.5, 143.9, 142.6, 131.0, 129.5, 127.92, 127.87, 127.5, 125.1, 118.0, 51.3, 49.9, 42.7, 42.0, 39.9, 37.7, 36.3, 21.7, 14.5, 13.9, 13.0, 11.2.

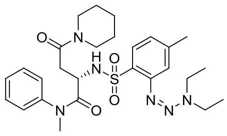

5n

(*S*,*Z*)-2-(2-(3,3-Diethyltriaz-1-enyl)-4-methylphenylsulfonamido)-*N*-methyl-4-oxo-*N*-phenyl-4-(piperidin-1-yl)butanamide (5n): Synthesized from *N*-methylaniline (**S1)**, Boc-L-Asp(OMe)-OH (**S2**) and piperidine (**S3**) following the method as **5l**. Eluent: petroleum ether/ethyl acetate (1:1). Yield: 98 mg (90%). Yellow thick oil.

^{1}H NMR ($CDCl_3$, 400 MHz) δ 7.64 (d, J = 7.8 Hz, 1H), 7.42 (s, 1H), 7.28 - 7.34 (m, 3H), 7.07 - 7.12 (m, 2H), 6.97 (d, J = 7.8 Hz, 1H), 6.39 (d, J = 8.2 Hz, 1H), 4.30 - 4.35 (m, 1H), 3.78 - 3.94 (m, 4H), 3.34 - 3.42 (m, 2H), 3.16 (s, 3H), 3.02 - 3.09 (m, 1H), 2.89 - 2.95 (m, 1H), 2.66 - 2.72 (m,

1H), 2.33 - 2.43 (m, 4H), 1.30 - 1.53 (m, 12H).

^{13}C NMR ($CDCl_3$, 100 MHz) δ 169.8, 167.1, 147.6, 143.8, 142.4, 130.7, 129.5, 127.9, 127.8, 127.4, 124.9, 117.9, 51.1, 49.8, 46.6, 42.6, 42.4, 37.7, 36.8, 26.0, 25.3, 24.3, 21.6, 14.4, 11.1.

General procedures for preparation and characterization data of E (2, 4 and 6)

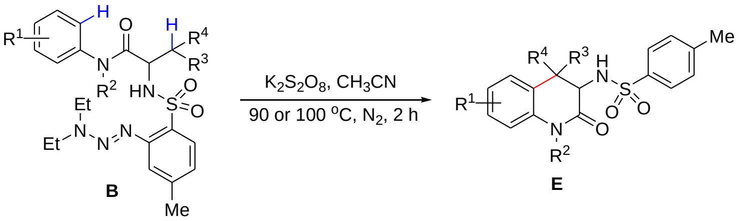

To a 25 mL Schlenk tube charged with a magnetic stirrer, **1**, **3** or **5** (0.1 mmol), $K_2S_2O_8$ (0.2 mmol, 54 mg) and anhydrous MeCN (2.0 mL) were added. The tube was evacuated and back-filled with nitrogen for three cycles and then sealed. It was placed in a preheated oil bath at 100 ℃, and the reaction was allowed to proceed for 2 hours. After completion of the reaction, the resulting mixture was filtered, and the filtrate was concentrated by a rotary evaporator. The residue was dissolved with EtOAc (3 mL), and the solution was washed with water (2 × 3 mL) and brine (2 × 3 mL), dried over $MgSO_4$, filtered and concentrated by a rotary evaporation. The residue was purified with preparative TLC (p-TLC) (silica gel, petroleum ether/EtOAc or dichloromethane/MeOH) to provide the target product (**2**, **4** or **6**).

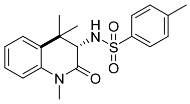

2a

(*S*)-4-Methyl-*N*-(1,4,4-trimethyl-2-oxo-1,2,3,4-tetrahydroquinolin-3-yl)benzenesulfonamide (2a): Starting with **1a** (0.1 mmol, 46 mg) and purified by p-TLC (silica gel, petroleum ether/EtOAc 10:1). Yield: 19.0 mg (53%).

White solid, mp: 134 - 136°C.

TLC (petroleum ether/EtOAc 5:1): R_f = 0.20.

HRMS (*m*/*z*): calcd for $C_{19}H_{22}N_2O_3SH^+$ $[M+H]^+$, 359.1424; found, 359.1428.

^{1}H NMR ($CDCl_3$, 400 MHz) δ 7.74 (d, *J* = 8.2 Hz, 2H), 7.37 (d, *J* = 7.4 Hz, 1H), 7.33 - 7.23 (m, 3H), 7.14 (t, *J* = 7.6 Hz, 1H), 6.97 (d, *J* = 8.0 Hz, 1H), 5.66 (d, *J* = 7.0 Hz, 1H), 3.71 (d, *J* = 7.0 Hz, 1H), 3.29 (s, 3H), 2.39 (s, 3H), 1.54 (s, 3H), 0.96 (s, 3H).

^{13}C NMR ($CDCl_3$, 100 MHz) δ 167.3, 143.7, 138.0, 136.4, 134.3, 129.7, 128.1, 127.7, 125.5, 124.5, 116.0, 60.6, 38.2, 31.1, 23.9, 21.8, 21.7.

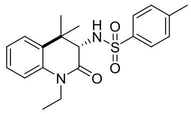

2b

(*S*)-*N*-(1-Ethyl-4,4-dimethyl-2-oxo-1,2,3,4-tetrahydroquinolin-3-yl)-4-methylbenzenesulfonamide (2b): Starting with **1b** (0.1 mmol, 47 mg) and purified by p-TLC (silica gel, petroleum ether/EtOAc 10:1). Yield: 24.2 mg (65%).

Orange solid, mp: 119 - 121°C.

TLC (petroleum ether/EtOAc 5:1): R_f = 0.29.

HRMS (*m/z*): calcd for $C_{20}H_{24}N_2O_3SH^+$ $[M+H]^+$, 373.1580; found, 373.1583.

^{1}H NMR ($CDCl_3$, 400 MHz) δ 7.74 (d, *J* = 8.2 Hz, 2H), 7.37 (d, *J* = 7.6 Hz, 1H), 7.21-7.32 (m, 3H), 7.12 (t, *J* = 7.5 Hz, 1H), 7.00 (d, *J* = 8.1 Hz, 1H), 5.66 (d, *J* = 7.2 Hz, 1H), 3.88 (q, *J* = 7.1 Hz, 2H), 3.70 (d, *J* = 7.2 Hz, 1H), 2.39 (s, 3H), 1.54 (s, 3H), 1.07 (t, *J* = 7.1 Hz, 3H), 0.95 (s, 3H).

^{13}C NMR ($CDCl_3$, 100 MHz) δ 166.7, 143.6, 136.7, 136.4, 134.8, 129.6, 128.2, 127.7, 125.8, 124.4, 115.8, 60.5, 38.8, 38.2, 23.9, 21.7, 21.6, 12.6.

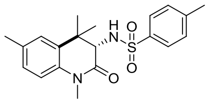

2c

(*S*)-4-Methyl-*N*-(1,4,4,6-tetramethyl-2-oxo-1,2,3,4-tetrahydroquinolin-3-yl)benzenesulfonamide (2c): Starting with **1c** (0.1 mmol, 47 mg) and purified by p-TLC (silica gel, petroleum ether/EtOAc 10:1). Yield: 21.2 mg (57%).

Orange solid, mp: 194 - 196°C.

TLC (petroleum ether/EtOAc 5:1): R_f = 0.22.

HRMS (*m/z*): calcd for $C_{20}H_{24}N_2O_3SH^+$ $[M+H]^+$, 373.1580; found, 373.1586.

^{1}H NMR ($CDCl_3$, 400 MHz) δ 7.73 (d, *J* = 8.2 Hz, 2H), 7.25 (d, *J* = 6.9 Hz, 2H), 7.16 (s, 1H), 7.08 (d, *J* = 8.1 Hz, 1H), 6.85 (d, *J* = 8.2 Hz, 1H), 5.66 (d, *J* = 6.9 Hz, 1H), 3.68 (d, *J* = 6.9 Hz, 1H), 3.27 (s, 3H), 2.39 (s, 3H), 2.34 (s, 3H), 1.53 (s, 3H), 0.94 (s, 3H).

^{13}C NMR ($CDCl_3$, 100 MHz) δ 167.2, 143.6, 136.5, 135.6, 134.2, 134.2, 129.7, 128.5, 127.7, 126.1, 115.9, 60.7, 38.1, 31.2, 23.9, 21.8, 21.7, 21.1.

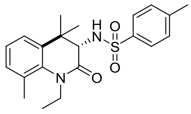

2d

(*S*)-*N*-(1-Ethyl-4,4,8-trimethyl-2-oxo-1,2,3,4-tetrahydroquinolin-3-yl)-4-methylbenzenesulfonamide (2d): Starting with **1d** (0.1 mmol, 49 mg) and purified by p-TLC (silica gel, petroleum

ether/EtOAc 10:1). Yield: 16.2 mg (42%).

Yellow solid, mp: 120 - 122°C.

TLC (petroleum ether/EtOAc 5:1): R_f = 0.33.

HRMS (*m*/*z*): calcd for $C_{21}H_{26}N_2O_3SH^+$ $[M+H]^+$, 387.1737; found, 387.1741.

^{1}H NMR ($CDCl_3$, 400 MHz) δ 7.72 (d, *J* = 8.2 Hz, 2H), 7.26 - 7.24 (m, 2H), 7.23 -7.17 (m, 1H), 7.13 - 7.08 (m, 2H), 5.83 (d, *J* = 7.2 Hz, 1H), 4.13 - 4.01 (m, 1H), 3.69 - 3.51 (m, 3H), 2.40 (s, 3H), 2.29 (s, 3H), 1.46 (s, 3H), 1.04 (t, *J* = 7.1 Hz, 3H), 0.95 (s, 3H).

^{13}C NMR ($CDCl_3$, 100 MHz) δ 169.5, 143.6, 138.6, 137.1, 136.8, 131.4, 129.7, 128.9, 127.50, 125.56, 123.2, 60.6, 42.4, 38.8, 24.6, 21.7, 21.4, 20.9, 12.7.

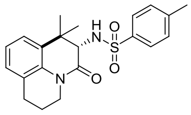

2e

(*S*)-*N*-(1,1-Dimethyl-3-oxo-1,2,3,5,6,7-hexahydropyrido[3,2,1-*ij*]quinolin-2-yl)-4-methylbenzenesulfonamide (2e): Starting with **1e** (0.1 mmol, 49 mg) and purified by p-TLC (silica gel, petroleum ether/EtOAc 10:1). Yield: 23.4 mg (61%).

Yellow oil.

TLC (petroleum ether/EtOAc 5:1): R_f = 0.20.

HRMS (*m*/*z*): calcd for $C_{21}H_{24}N_2O_3SH^+$ $[M+H]^+$, 385.1580; found, 385.1585.

^{1}H NMR ($CDCl_3$, 600 MHz) δ 7.75 (d, *J* = 8.2 Hz, 2H), 7.26 (d, *J* = 8.2 Hz, 2H), 7.20 (dd, *J* = 7.1 Hz, 2.8 Hz, 1H), 7.05 - 7.00 (m, 2H), 5.63 (d, *J* = 6.9 Hz, 1H), 4.09 - 4.02 (m, 1H), 3.76 (d, *J* = 7.0 Hz, 1H), 3.44 - 3.36 (m, 1H), 2.86 - 2.70 (m, 2H), 2.40 (s, 3H), 1.93 - 1.78 (m, 2H), 1.52 (s, 3H), 0.96 (s, 3H).

^{13}C NMR ($CDCl_3$, 150 MHz) δ 166.4, 143.6, 136.6, 133.6, 133.4, 129.6, 128.5, 127.7, 126.0, 124.0, 123.4, 60.6, 41.8, 38.1, 27.2, 24.0, 21.9, 21.7, 21.5.

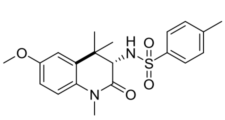

2f

(*S*)-*N*-(6-Methoxy-1,4,4-trimethyl-2-oxo-1,2,3,4-tetrahydroquinolin-3-yl)-4-methylbenzenesulfonamide (2f): Starting with **1f** (0.1 mmol, 49 mg) and purified by p-TLC (silica gel, petroleum ether/EtOAc 5:1). Yield: 20.2 mg (52%).

White solid, mp: 165 - 167°C.

TLC (petroleum ether/EtOAc 5:1): R_f = 0.12.

HRMS (*m*/*z*): calcd for $C_{20}H_{24}N_2O_4SH^+$ $[M+H]^+$, 389.1530; found, 389.1536.

^{1}H NMR ($CDCl_3$, 400 MHz) δ 7.74 (d, *J* = 8.2 Hz, 2H), 7.26 (d, *J* = 8.2 Hz, 2H), 6.92 (d, *J* = 2.8 Hz, 1H), 6.89(d, *J* = 8.7 Hz, 1H), 6.79(dd, *J* = 8.7 Hz, 2.8 Hz, 1H), 5.67 (d, *J* = 6.9 Hz, 1H), 3.81 (s, 3H), 3.67 (d, *J* = 6.9 Hz, 1H), 3.26 (s, 3H), 2.39 (s, 3H), 1.52 (s, 3H), 0.95 (s, 3H).

^{13}C NMR ($CDCl_3$, 100 MHz) δ 166.7, 156.5, 143.6, 136.4, 136.0, 131.5, 129.6, 127.6, 116.9, 112.0, 112.0, 60.5, 55.7, 38.3, 31.3, 23.9, 21.7.

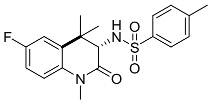

2g

(*S*)-*N*-(6-Fluoro-1,4,4-trimethyl-2-oxo-1,2,3,4-tetrahydroquinolin-3-yl)-4-methylbenzenesulfonamide (2g): Starting with **1g** (0.1 mmol, 48 mg) and purified by p-TLC (silica gel, petroleum ether/EtOAc 15:1). Yield: 14.7 mg (39%).

White solid, mp: 68 - 70°C.

TLC (petroleum ether/EtOAc 5:1): R_f = 0.50.

HRMS (*m*/*z*): calcd for $C_{19}H_{21}FN_2O_3SH^+$ $[M+H]^+$, 377.1330; found, 377.1333.

^{1}H NMR ($CDCl_3$, 400 MHz) δ 7.74 (d, *J* = 8.3 Hz, 2H), 7.27 (d, *J* = 6.4 Hz, 2H), 7.08 (dd, *J* = 9.3 Hz, 3.2Hz, 1H), 7.01 - 6.96 (m, 1H), 6.91 (dd, *J* = 8.9 Hz, 5.0Hz, 1H), 5.64 (d, *J* = 7.0 Hz, 1H), 3.69 (d, *J* = 7.0 Hz, 1H), 3.28 (s, 3H), 2.40 (s, 3H), 1.51 (s, 3H), 0.95 (s, 3H).

^{13}C NMR ($CDCl_3$, 100 MHz) δ 166.9, 159.6 (d, *J* = 244.4 Hz), 143.8, 136.7 (d, *J* = 7.7 Hz), 136.4, 134.2 (d, *J* = 1.9 Hz), 129.7, 127.7, 117.3 (d, *J* = 8.6 Hz), 114.5 (d, *J* = 23.0 Hz), 112.9 (d, *J* = 24.0 Hz), 60.4, 38.3, 31.5, 23.9, 21.7, 21.6.

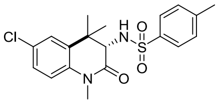

2h

(*S*)-*N*-(6-Chloro-1,4,4-trimethyl-2-oxo-1,2,3,4-tetrahydroquinolin-3-yl)-4-methylbenzenesulfonamide (2h): Starting with **1h** (0.1 mmol, 49 mg) and purified by p-TLC (silica gel, petroleum ether/EtOAc 5:1). Yield: 21.5 mg (55%).

White solid, mp: 205 - 207°C.

TLC (petroleum ether/EtOAc 5:1): R_f = 0.16.

HRMS (*m*/*z*): calcd for $C_{19}H_{21}ClN_2O_3SH^+$ $[M+H]^+$, 393.1034; found, 393.1035.

^{1}H NMR ($CDCl_3$, 400 MHz) δ 7.74 (d, *J* = 8.3 Hz, 2H), 7.32 (d, *J* = 2.4 Hz, 1H), 7.29 - 7.23 (m, 3H), 6.89 (d, *J* = 8.6 Hz, 1H), 5.63 (d, *J* = 7.0 Hz, 1H), 3.69 (d, *J* = 7.1 Hz, 1H), 3.28 (s, 3H), 2.40

(s, 3H), 1.51 (s, 3H), 0.95 (s, 3H).

^{13}C NMR ($CDCl_3$, 100 MHz) δ 167.1, 143.8, 136.6, 136.4, 136.1, 129.9, 129.7, 128.0, 127.6, 125.8, 117.2, 60.4, 38.4, 31.3, 23.8, 21.7, 21.6.

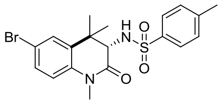

2i

(*S*)-*N*-(6-Bromo-1,4,4-trimethyl-2-oxo-1,2,3,4-tetrahydroquinolin-3-yl)-4-methylbenzenesulfonamide (2i): Starting with **1i** (0.1 mmol, 54 mg) and purified by p-TLC (silica gel, petroleum ether/EtOAc 5:1). Yield: 27.0 mg (62%).

White solid, mp: 232 - 234°C.

TLC (petroleum ether/EtOAc 5:1): R_f = 0.15.

HRMS (*m*/*z*): calcd for $C_{19}H_{21}BrN_2O_3SH^+$ $[M+H]^+$, 437.0529; found, 437.0531.

^{1}H NMR ($CDCl_3$, 400 MHz) δ 7.74 (d, *J* = 8.3 Hz, 2H), 7.46 (d, *J* = 2.2 Hz, 1H), 7.40 (dd, *J* = 8.7 Hz, 2.2 Hz, 1H), 7.27 (d, *J* = 7.8 Hz, 2H), 6.84 (d, *J* = 8.6 Hz, 1H), 5.63 (d, *J* = 7.0 Hz, 1H), 3.69 (d, *J* = 7.1 Hz, 1H), 3.27 (s, 3H), 2.40 (s, 3H), 1.51 (s, 3H), 0.95 (s, 3H).

^{13}C NMR ($CDCl_3$, 100 MHz) δ 167.1, 143.8, 137.1, 136.4, 136.4, 131.0, 129.7, 128.6, 127.6, 117.6, 117.5, 60.4, 38.4, 31.2, 23.8, 21.7, 21.6.

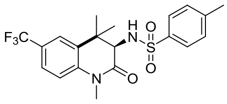

2j

(*R*)-4-Methyl-*N*-(1,4,4-trimethyl-2-oxo-6-(trifluoromethyl)-1,2,3,4-tetrahydroquinolin-3-yl)benzenesulfonamide (2j): Starting with **1j** (0.1 mmol, 53 mg) and purified by p-TLC (silica gel, petroleum ether/EtOAc 5:1). Yield: 28.1 mg (66%).

White solid, mp: 177 - 179°C.

TLC (petroleum ether/EtOAc 5:1): R_f = 0.17;

HRMS (*m*/*z*): calcd for $C_{20}H_{21}F_3N_2O_3SH^+$ $[M+H]^+$, 427.1298; found, 427.1296.

^{1}H NMR ($CDCl_3$, 600 MHz) δ 7.74 (d, *J* = 8.2 Hz, 2H), 7.59 (s, 1H), 7.56 (d, *J* = 8.6 Hz, 1H), 7.29 - 7.25 (m, 2H), 7.05 (d, *J* = 8.4 Hz, 1H), 5.59 (d, *J* = 7.1 Hz, 1H), 3.75 (d, *J* = 7.2 Hz, 1H), 3.33 (s, 3H), 2.40 (s, 3H), 1.56 (s, 3H), 0.98 (s, 3H).

^{13}C NMR ($CDCl_3$, 150 MHz) δ 167.5, 143.8, 140.9, 136.5, 135.0, 129.8, 127.7, 126.5 (q, J = 33.2 Hz), 125.5(m), 124.1 (q, *J* = 271.7 Hz), 122.7(m), 116.0, 60.5, 38.5, 31.2, 23.8, 21.71, 21.66.

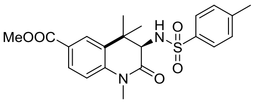

2k

(*R*)-Methyl 1,4,4-trimethyl-3-(4-methylphenylsulfonamido)-2-oxo-1,2,3,4-tetrahydroquinoline-6-carboxylate (2k): Starting with **1k** (0.1 mmol, 52 mg) and purified by p-TLC (silica gel, petroleum ether/EtOAc 5:1). Yield: 27.0 mg (65%).

White solid, mp: 201 - 203°C.

TLC (petroleum ether/EtOAc 5:1): R_f = 0.10;

HRMS (*m*/*z*): calcd for $C_{21}H_{24}N_2O_5SH^+$ $[M+H]^+$, 417.1479; found, 417.1483.

^{1}H NMR ($CDCl_3$, 600 MHz) δ 8.03 (d, *J* = 1.6 Hz, 1H), 7.97 (d, *J* = 8.6 Hz, 1.6 Hz, 1H), 7.74 (d, *J* = 8.2 Hz, 2H), 7.29 - 7.25 (m, 2H), 7.01 (d, *J* = 8.4 Hz, 1H), 5.59 (d, *J* = 7.2 Hz, 1H), 3.92 (s, 3H), 3.76 (d, *J* = 7.3 Hz, 1H), 3.33 (s, 3H), 2.40 (s, 3H), 1.57 (s, 3H), 0.97 (s, 3H).

^{13}C NMR ($CDCl_3$, 150 MHz) δ 167.7, 166.5, 143.8, 141.8, 136.5, 134.2, 130.0, 129.72, 127.7, 127.1, 126.1, 115.7, 60.7, 52.3, 38.4, 31.2, 23.9, 21.72, 21.70.

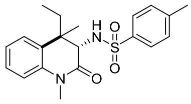

2l

***N*-((*3S,4S*)-4-Ethyl-1,4-dimethyl-2-oxo-1,2,3,4-tetrahydroquinolin-3-yl)-4-methylbenzenesulfonamide (2l)**: Starting with **1l** (0.1 mmol, 47 mg) and purified by p-TLC (silica gel, petroleum ether/EtOAc 10:1). Yield: 18.2 mg (49%).

White solid, mp: 152 - 154°C.

TLC (petroleum ether/EtOAc 5:1): R_f = 0.26.

HRMS (*m*/*z*): calcd for $C_{20}H_{24}N_2O_3SH^+$ $[M+H]^+$, 373.1580; found, 373.1589.

^{1}H NMR ($CDCl_3$, 400 MHz) δ 7.74 (d, *J* = 8.2 Hz, 2H), 7.33 - 7.23 (m, 4H), 7.12 (t, *J* = 7.5 Hz, 1H), 6.96 (d, *J* = 8.0 Hz, 1H), 5.64 (d, *J* = 7.1 Hz, 1H), 3.81 (d, *J* = 7.2 Hz, 1H), 3.27 (s, 3H), 2.39 (s, 3H), 1.47 (s, 3H), 1.37 - 1.44 (m, 1H), 1.17 - 1.26 (m, 1H), 0.63 (t, *J* = 7.4 Hz, 3H).

^{13}C NMR ($CDCl_3$, 100 MHz) δ 167.4, 143.6, 138.2, 136.5, 131.9, 129.7, 128.1, 127.7, 127.4, 124.0, 116.0, 61.4, 41.6, 31.2, 25.2, 21.7, 20.2, 8.3.

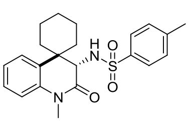

2m

(*S*)-4-Methyl-*N*-(1'-methyl-2'-oxo-2',3'-dihydro-1'*H*-spiro[cyclohexane-1,4'-quinoline]-3'-yl)benzenesulfonamide (2m): Starting with **1m** (0.1 mmol, 50 mg) and purified by p-TLC (silica gel,

petroleum ether/EtOAc 10:1). Yield: 18.3 mg (46%).

White solid, mp: 182 - 184°C.

TLC (petroleum ether/EtOAc 5:1): R_f = 0.17.

HRMS (*m*/*z*): calcd for $C_{22}H_{26}N_2O_3SH^+$ $[M+H]^+$, 399.1737; found, 399.1745.

^{1}H NMR ($CDCl_3$, 400 MHz) δ 7.59 (d, *J* = 8.3 Hz, 2H), 7.38 - 7.30 (m, 4H), 7.29 - 7.20 (m, 2H), 6.74 (d, *J* = 7.8 Hz, 1H), 3.90 (d, *J* = 10.4 Hz, 1H), 2.92 (s, 3H), 2.46 (s, 3H), 1.94 - 1.81 (m, 4H), 1.72 - 1.53 (m, 4H), 1.39 - 1.23 (m, 2H).

^{13}C NMR ($CDCl_3$, 100 MHz) δ 169.6, 143.7, 141.6, 138.2, 136.8, 130.03, 129.5, 128.2, 127.5, 127.0, 125.7, 122.1, 64.5, 56.8, 42.4, 35.8, 33.3, 30.0, 26.1, 25.8, 21.7.

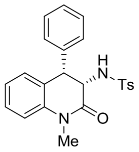

4a

4-Methyl-*N*-((*3S*,*4S*)-1-methyl-2-oxo-4-phenyl-1,2,3,4-tetrahydroquinolin-3-yl)benzenesulfonamide (4a): Starting with **3a** (0.1 mmol, 51 mg) and purified by p-TLC (silica gel, petroleum ether/EtOAc 10:1). Yield: 20.3 mg (50%).

White solid, mp: 162 - 164°C.

TLC (petroleum ether/EtOAc 5:1): R_f = 0.25.

HRMS (*m*/*z*): calcd for $C_{23}H_{22}N_2O_3SH^+$ $[M+H]^+$, 407.1424; found, 407.1421.

^{1}H NMR ($CDCl_3$, 400 MHz) δ 7.75 (d, *J* = 8.2 Hz, 2H), 7.35 (td, J^1 = 8.00 Hz, J^2 = 1.37 Hz, 1H), 7.28-7.21 (m, 6H), 7.13-7.06 (m, 2H), 7.04-7.02 (m, 2H), 5.65 (d, *J* = 4.12 Hz, 1H), 4.57 (d, *J* = 6.87 Hz, 1H), 4.17 (dd, J^1 = 4.58 Hz, J^2 = 6.41 Hz, 1H), 3.37 (s, 3H), 2.39 (s, 3H).

^{13}C NMR ($CDCl_3$, 100 MHz) δ 165.9, 143.8, 139.3, 136.2, 130.0, 129.7, 129.2, 128.9, 128.6, 127.7, 127.6, 127.4, 124.7, 115.9, 56.2, 47.3, 30.9, 21.7.

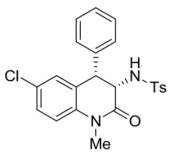

4b

***N*-((*3S*,*4S*)-6-Chloro-1-methyl-2-oxo-4-phenyl-1,2,3,4-tetrahydroquinolin-3-yl)-4-methylbenzenesulfonamide (4b)**: Starting with **3b** (0.1 mmol, 54 mg) and purified by p-TLC (silica gel, petroleum ether/EtOAc 10:1). Yield: 24.2 mg (55%).

Yellow solid, mp: 219 - 221°C.

TLC (petroleum ether/EtOAc 5:1): R_f = 0.20.

HRMS (*m*/*z*): calcd for $C_{23}H_{21}ClN_2O_3SH^+$ $[M+H]^+$, 441.1034; found, 441.1038.

^{1}H NMR ($CDCl_3$, 600 MHz) δ 7.74 (d, *J* = 8.24 Hz, 2H), 7.31 (dd, J^1 = 2.06 Hz, J^2 = 8.24 Hz, 1H), 7.28-7.22 (m, 6H), 7.01-6.99 (m, 3H), 5.61 (d, *J* = 4.81 Hz, 1H), 4.53 (d, *J* = 6.18 Hz, 1H), 4.15 (dd, J^1 = 4.81 Hz, J^2 = 6.87 Hz, 1H), 3.35 (s, 3H), 2.39 (s, 3H).

^{13}C NMR ($CDCl_3$, 150 MHz) δ 165.6, 143.9, 137.9, 136.2, 135.5, 130.0, 129.9, 129.6, 129.2, 129.1, 128.84, 128.79, 127.9, 127.4, 117.2, 55.9, 47.2, 31.0, 21.7.

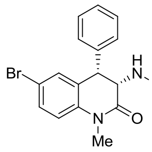

4c

***N*-((3*S*,4*S*)-6-Bromo-1-methyl-2-oxo-4-phenyl-1,2,3,4-tetrahydroquinolin-3-yl)-4-methylbenzenesulfonamide (4c)**: Starting with **3c** (0.1 mmol, 59 mg) and purified by p-TLC (silica gel, petroleum ether/EtOAc 10:1). Yield: 28.5 mg (59%).

Yellow solid, mp: 251 - 253°C.

TLC (petroleum ether/EtOAc 5:1): R_f = 0.21.

HRMS (*m*/*z*): calcd for $C_{23}H_{21}BrN_2O_3SH^+$ $[M+H]^+$, 485.0529; found, 485.0530.

^{1}H NMR ($CDCl_3$, 600 MHz) δ 7.74 (d, *J* = 8.24 Hz, 2H), 7.46 (dd, J^1 = 2.06 Hz, J^2 = 8.93 Hz, 1H), 7.38 (d, *J* = 2.06 Hz, 1H), 7.28-7.23 (m, 5H), 7.00 (dd, J^1 = 2.06 Hz, J^2 = 8.25 Hz, 2H), 6.94 (d, *J* = 8.93 Hz, 1H), 5.61 (d, *J* = 4.81 Hz, 1H), 4.53 (d, *J* = 6.18 Hz, 1H), 4.14 (dd, J^1 = 4.81 Hz, J^2 = 6.18 Hz, 1H), 3.34 (s, 3H), 2.40 (s, 3H).

^{13}C NMR ($CDCl_3$, 150 MHz) δ 165.6, 143.9, 138.5, 136.2, 135.5, 132.5, 131.8, 130.0, 129.6, 129.1, 128.8, 127.9, 127.4, 117.6, 117.4, 55.9, 47.1, 31.0, 21.7.

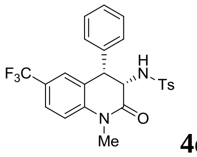

4d

4-Methyl-*N*-((3*S*,4*S*)-1-methyl-2-oxo-4-phenyl-6-(trifluoromethyl)-1,2,3,4-tetrahydroquinolin-3-yl)benzenesulfonamide (4d): Starting with **3d** (0.1 mmol, 58 mg) and purified by p-TLC (silica gel, petroleum ether/EtOAc 10:1). Yield: 29.4 mg (62%).

White solid, mp: 207 - 209°C.

TLC (petroleum ether/EtOAc 5:1): R_f = 0.19.

HRMS (m/z): calcd for $C_{24}H_{21}F_3N_2O_3SH^+$ $[M+H]^+$, 475.1298; found, 475.1295.

^1H NMR ($CDCl_3$, 600 MHz) δ 7.75 (d, J = 8.24 Hz, 2H), 7.61 (d, J = 8.93 Hz, 1H), 7.51-7.50 (m, 1H), 7.28 (d, J = 8.24 Hz, 2H), 7.26-7.24 (m, 3H), 7.16 (d, J = 8.24 Hz, 1H), 7.00 (dd, J^1 = 2.06 Hz, J^2 = 7.56 Hz, 2H), 5.57 (d, J = 4.12 Hz, 1H), 4.62 (d, J = 6.87 Hz, 1H), 4.20 (dd, J^1 = 4.13 Hz, J^2 = 6.18 Hz, 1H), 3.40 (s, 3H), 2.40 (s, 3H).

^{13}C NMR ($CDCl_3$, 150 MHz) δ 166.0, 144.0, 142.2, 136.2, 135.3, 130.0, 129.0, 128.9, 128.6, 128.14, 128.07, 127.3, 126.9-126.8 (m), 126.2-126.1 (m), 125.7 (q, J = 285.8 Hz), 116.1, 56.0, 47.3, 31.1, 21.7.

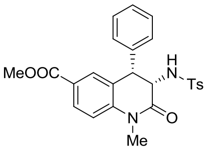

4e

(*3S,4S*)-Methyl 1-methyl-3-(4-methylphenylsulfonamido)-2-oxo-4-phenyl-1,2,3,4-tetrahydroquinoline-6-carboxylate (4e): Starting with **3e** (0.1 mmol, 57 mg) and purified by p-TLC (silica gel, petroleum ether/EtOAc 5:1). Yield: 29.7 mg (64%).

White solid, mp: 193 - 195°C.

TLC (petroleum ether/EtOAc 5:1): R_f = 0.14.

HRMS (m/z): calcd for $C_{25}H_{24}N_2O_3SH^+$ $[M+H]^+$, 465.1479; found, 465.1481.

^1H NMR ($CDCl_3$, 600 MHz) δ 8.03 (dd, J^1 = 2.10 Hz, J^2 = 8.94 Hz, 1H), 7.92 (d, J = 2.04 Hz, 1H), 7.75 (d, J = 8.28 Hz, 2H), 7.28 (d, J = 8.28 Hz, 2H), 7.24-7.22 (m, 3H), 7.12 (d, J = 8.94 Hz, 1H), 7.00-6.99 (m, 2H), 5.59 (d, J = 4.80 Hz, 1H), 4.63 (d, J = 6.18 Hz, 1H), 4.20 (dd, J^1 = 4.80 Hz, J^2 = 6.18 Hz, 1H), 3.88 (s, 3H), 3.40 (s, 3H), 2.40 (s, 3H).

^{13}C NMR ($CDCl_3$, 150 MHz) δ 166.2, 166.1, 143.9, 143.0, 136.2, 135.7, 131.1, 130.6, 130.0, 129.0, 128.8, 127.9, 127.44, 127.40, 126.2, 115.7, 56.1, 52.3, 47.2, 31.0, 21.7.

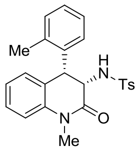

4f

4-Methyl-*N*-((*3S,4S*)-1-methyl-2-oxo-4-o-tolyl-1,2,3,4-tetrahydroquinolin-3-yl)benzenesulfon amide (4f): Starting with **3f** (0.1 mmol, 52 mg) and purified by p-TLC (silica gel, petroleum ether/EtOAc 10:1). Yield: 18.1 mg (43%).

Yellow solid, mp: 66 - 68°C.

TLC (petroleum ether/EtOAc 5:1): R_f = 0.29.

HRMS (*m/z*): calcd for $C_{24}H_{24}N_2O_3SH^+$ $[M+H]^+$, 421.1580; found, 421.1587.

^{1}H NMR ($CDCl_3$, 400 MHz) δ 7.73 (d, *J* = 8.24 Hz, 2H), 7.32 (td, J^1 = 7.80 Hz, J^2 = 1.36 Hz, 1H), 7.26 (d, *J* = 8.24 Hz, 2H), 7.23-7.17 (m, 2H), 7.12-7.05 (m, 3H), 6.96 (t, *J* = 7.56 Hz, 1H), 6.54 (d, *J* = 7.76 Hz, 1H), 5.51 (d, *J* = 5.48 Hz, 1H), 4.90 (d, *J* = 6.88 Hz, 1H), 4.30 (t, *J* = 5.96 Hz, 1H), 3.40 (s, 3H), 2.70 (s, 3H), 2.39 (s, 3H).

^{13}C NMR ($CDCl_3$, 75 MHz) δ 166.2, 143.7, 139.4, 138.0, 136.3, 135.8, 131.3, 129.9, 129.8, 128.8, 128.7, 127.6, 127.4, 127.0, 126.6, 124.6, 116.1, 56.6, 43.4, 31.0, 21.7, 21.0.

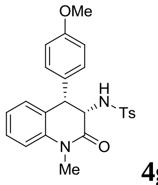

4g

***N*-((*3S,4S*)-4-(4-Methoxyphenyl)-1-methyl-2-oxo-1,2,3,4-tetrahydroquinolin-3-yl)-4-methylbenzenesulfonamide (4g)**: Starting with **3g** (0.1 mmol, 54 mg) and purified by p-TLC (silica gel, petroleum ether/EtOAc 10:1). Yield: 20.9 mg (48%).

White solid, mp: 143 - 145°C.

TLC (petroleum ether/EtOAc 5:1): R_f = 0.18.

HRMS (*m/z*): calcd for $C_{24}H_{24}N_2O_4SH^+$ $[M+H]^+$, 437.1530; found, 437.1535.

^{1}H NMR ($CDCl_3$, 400 MHz) δ 7.74 (d, *J* = 8.72 Hz, 2H), 7.34 (td, J^1 = 7.80 Hz, J^2 = 1.40 Hz, 1H), 7.26 (d, *J* = 8.12 Hz, 2H), 7.23 (d, *J* = 7.80 Hz, 1H), 7.10 (t, *J* = 7.76 Hz, 1H), 7.06 (d, *J* = 8.24 Hz, 1H), 6.94 (d, *J* = 8.68 Hz, 2H), 6.77 (d, *J* = 8.72 Hz, 2H), 5.66 (d, *J* = 4.12 Hz, 1H), 4.52 (d, *J* = 6.40 Hz, 1H), 4.12 (dd, J^1 = 4.12 Hz, J^2 = 6.44 Hz, 1H), 3.74 (s, 3H), 3.35 (s, 3H), 2.38 (s, 3H).

^{13}C NMR ($CDCl_3$, 100 MHz) δ 165.9, 159.0, 143.8, 139.2, 136.1, 130.2, 130.0, 129.6, 128.8, 128.0, 127.8, 127.4, 124.7, 115.9, 114.0, 56.3, 55.3, 46.5, 30.9, 21.7.

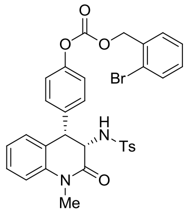

4h

2-Bromobenzyl 4-((3S,4S)-1-methyl-3-(4-methylphenylsulfonamido)-2-oxo-1,2,3,4-tetrahydroquinolin-4-yl)phenyl carbonate (4h): Starting with **3h** (0.1 mmol, 74 mg) and

purified by p-TLC (silica gel, petroleum ether/EtOAc 5:1). Yield: 35.5 mg (56%).

White solid, mp: 72 - 74°C.

TLC (petroleum ether/EtOAc 5:1): R_f = 0.12.

HRMS (*m*/*z*): calcd for $C_{31}H_{27}BrN_2O_6SH^+$ $[M+H]^+$, 635.0846; found,635.0845.

^{1}H NMR ($CDCl_3$, 300 MHz) δ 7.74 (d, *J* = 8.22 Hz, 2H), 7.59 (dd, J^1 = 7.89 Hz, J^2 = 1.38 Hz, 1H), 7.48 (dd, J^1 = 7.53 Hz, J^2 = 1.35 Hz, 1H), 7.39-7.31 (m, 2H), 7.28-7.19 (m, 4H), 7.13-7.01 (m, 6H), 5.71 (d, *J* = 4.11 Hz, 1H), 5.34 (s, 2H), 4.59 (d, *J* = 6.51 Hz, 1H), 4.14 (dd, J^1 = 4.14 Hz, J^2 = 6.54 Hz, 1H), 3.35 (s, 3H), 2.38 (s, 3H).

^{13}C NMR ($CDCl_3$, 75 MHz) δ 165.6, 153.4, 150.5, 143.9, 139.2, 135.9, 134.3, 134.0, 133.1, 130.4, 130.31, 130.27, 130.0, 129.8, 129.1, 127.8, 127.4, 127.1, 124.8, 123.6, 121.1, 116.0, 69.7, 56.0, 46.5, 30.9, 21.7.

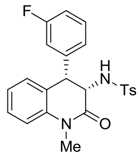

4i

***N*-((*3S,4S*)-4-(3-Fluorophenyl)-1-methyl-2-oxo-1,2,3,4-tetrahydroquinolin-3-yl)-4-methylbenzenesulfonamide (4i)**: Starting with **3i** (0.1 mmol, 53 mg) and purified by p-TLC (silica gel, petroleum ether/EtOAc 10:1). Yield: 28.0 mg (66%).

Yellow solid, mp: 68 - 70°C.

TLC (petroleum ether/EtOAc 5:1): R_f = 0.23.

HRMS (*m*/*z*): calcd for $C_{23}H_{21}FN_2O_3SH^+$ $[M+H]^+$, 425.1330; found, 425.1341.

^{1}H NMR ($CDCl_3$, 400 MHz) δ 7.75 (d, *J* = 8.28 Hz, 2H), 7.38 (td, J^1 = 8.24 Hz, J^2 = 1.36 Hz, 1H), 7.29-7.19 (m, 4H), 7.13 (t, *J* = 7.32 Hz, 1H), 7.08 (d, *J* = 8.24 Hz, 1H), 6.93-6.89 (m, 2H), 6.63 (d, *J* = 10.08 Hz, 1H), 5.71 (d, *J* = 3.64 Hz, 1H), 4.58 (d, *J* = 6.44 Hz, 1H), 4.14 (dd, J^1 = 4.12 Hz, J^2 = 6.40 Hz, 1H), 3.37 (s, 3H), 2.39 (s, 3H).

^{13}C NMR ($CDCl_3$, 100 MHz) δ 165.6, 162.8 (d, *J* = 245.0 Hz), 143.9, 139.2, 138.7 (d, *J* = 7.62 Hz), 135.9, 130.1, 130.0, 129.7, 129.2, 127.4, 126.9, 125.3 (d, *J* = 2.86 Hz), 124.8, 116.1, 115.9 (d, *J* = 21.93 Hz), 114.6 (d, *J* = 20.97 Hz), 55.9, 46.8, 30.9, 21.7.

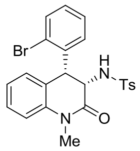

4j

***N*-((*3S,4R*)-4-(2-Bromophenyl)-1-methyl-2-oxo-1,2,3,4-tetrahydroquinolin-3-yl)-4-methylbenzenesulfonamide (4j)**: Starting with **3j** (0.1 mmol, 59 mg) and purified by p-TLC (silica gel, petroleum ether/EtOAc 5:1). Yield: 32.4 mg (57%).

Yellow solid, mp: 91 - 93°C.

TLC (petroleum ether/EtOAc 5:1): R_f = 0.14.

HRMS (*m*/*z*): calcd for $C_{23}H_{21}BrN_2O_3SH^+$ $[M+H]^+$, 485.0529; found, 485.0542.

^{1}H NMR ($CDCl_3$, 400 MHz) δ 7.74 (d, *J* = 8.24 Hz, 2H), 7.62 (dd, J^1 = 7.80 Hz, J^2 = 1.84 Hz, 1H), 7.40 (dd, J^1 = 7.32 Hz, J^2 = 1.36 Hz, 1H), 7.32 (td, J^1= 7.80 Hz, J^2 = 1.36 Hz, 1H), 7.26 (d, *J* = 8.24 Hz, 2H), 7.12-7.04 (m, 4H), 6.72 (dd, J^1= 7.32 Hz, J^2 = 1.84 Hz, 1H), 5.50 (d, *J* = 5.48 Hz, 1H), 5.30 (d, *J* = 7.36 Hz, 1H), 4.37 (t, *J* = 6.44 Hz, 1H), 3.41 (s, 3H), 2.39 (s, 3H).

^{13}C NMR ($CDCl_3$, 100 MHz) δ 166.3, 143.6, 138.8, 137.4, 136.6, 134.0, 129.82, 129.75, 129.2, 129.0, 128.2, 128.1, 127.5, 127.4, 126.0, 124.6, 116.1, 55.9, 45.6, 31.0, 21.7.

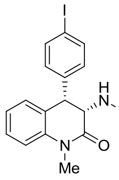

4k

***N*-((*3S,4S*)-4-(4-Iodophenyl)-1-methyl-2-oxo-1,2,3,4-tetrahydroquinolin-3-yl)-4-methylbenzenesulfonamide (4k)**: Starting with **3k** (0.1 mmol, 63 mg) and purified by p-TLC (silica gel, petroleum ether/EtOAc 10:1). Yield: 31.9 mg (60%).

Yellow solid, mp: 164 - 166°C.

TLC (petroleum ether/EtOAc 5:1): R_f = 0.26.

HRMS (*m*/*z*): calcd for $C_{23}H_{21}IN_2O_3SH^+$ $[M+H]^+$, 533.0390; found, 533.0404.

^{1}H NMR ($CDCl_3$, 400 MHz) δ 7.74 (d, *J* = 8.72 Hz, 2H), 7.56 (d, *J* = 8.24 Hz, 2H), 7.37 (td, J^1 = 7.80 Hz, J^2 = 1.40 Hz, 1H), 7.27 (d, *J* = 8.24 Hz, 2H), 7.22 (dd, J^1 = 7.32 Hz, J^2 = 1.36 Hz, 1H), 7.12 (t, *J* = 7.32 Hz, 1H), 7.07 (d, *J* = 8.24 Hz, 1H), 6.78 (d, *J* = 8.68 Hz, 2H), 5.70 (d, *J* = 3.68 Hz, 1H), 4.55 (d, *J* = 6.44 Hz, 1H), 4.12 (dd, J^1 = 3.68 Hz, J^2 = 6.88 Hz, 1H), 3.35 (s, 3H), 2.39 (s, 3H).

^{13}C NMR ($CDCl_3$, 100 MHz) δ 165.5, 143.9, 139.2, 137.7, 135.9, 131.2, 130.0, 129.7, 129.2, 127.4, 126.8, 124.8, 116.0, 93.5, 55.9, 46.6, 30.9, 21.7.

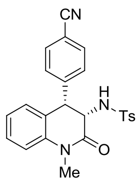

4l

***N*-((*3S,4S*)-4-(4-Cyanophenyl)-1-methyl-2-oxo-1,2,3,4-tetrahydroquinolin-3-yl)-4-methylbenzenesulfonamide (4l)**: Starting with **3l** (0.1 mmol, 53 mg) and purified by p-TLC (silica gel, petroleum ether/EtOAc 5:1). Yield: 30.2 mg (70%).

Yellow solid, mp: 126 - 128°C.

TLC (petroleum ether/EtOAc 5:1): R_f = 0.10.

HRMS (*m/z*): calcd for $C_{24}H_{21}N_3O_3SH^+$ $[M+H]^+$, 432.1376; found, 432.1378.

^{1}H NMR ($CDCl_3$, 400 MHz) δ 7.74 (d, *J* = 8.24 Hz, 2H), 7.54 (d, *J* = 8.28 Hz, 2H), 7.41 (td, J^1 = 8.24 Hz, J^2 = 1.36 Hz, 1H), 7.29 (d, *J* = 8.24 Hz, 2H), 7.24 (dd, J^1 = 7.80 Hz, J^2 = 1.84 Hz, 1H), 7.18-7.15 (m, 3H), 7.11 (d, *J* = 8.24 Hz, 1H), 5.74 (d, *J* = 3.20 Hz, 1H), 4.69 (d, *J* = 6.88 Hz, 1H), 4.13 (dd, J^1 = 3.64 Hz, J^2 = 6.88 Hz, 1H), 3.38 (s, 3H), 2.40 (s, 3H).

^{13}C NMR ($CDCl_3$, 100 MHz) δ 165.2, 144.2, 141.7, 139.1, 135.5, 132.3, 130.2, 130.1, 129.8, 129.5, 127.4, 125.9, 125.0, 118.7, 116.2, 111.6, 55.8, 46.9, 31.0, 21.7.

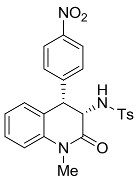

4m

4-Methyl-*N*-((*3S,4S*)-1-methyl-4-(4-nitrophenyl)-2-oxo-1,2,3,4-tetrahydroquinolin-3-yl)benzenesulfonamide (4m): Starting with **3m** (0.1 mmol, 55 mg) and purified by p-TLC (silica gel, petroleum ether/EtOAc 10:1). Yield: 34.3 mg (76%).

Orange solid, mp: 119 - 121°C.

TLC (petroleum ether/EtOAc 5:1): R_f = 0.16.

HRMS (*m/z*): calcd for $C_{23}H_{21}N_3O_5SH^+$ $[M+H]^+$, 452.1275; found, 452.1280.

^{1}H NMR ($CDCl_3$, 400 MHz) δ 8.10 (d, *J* = 8.72 Hz, 2H), 7.75 (d, *J* = 8.24 Hz, 2H), 7.42 (td, J^1 = 8.28 Hz, J^2 = 1.40 Hz, 1H), 7.29 (d, *J* = 8.24 Hz, 2H), 7.26-7.23 (m, 3H), 7.16 (td, J^1 = 7.32 Hz, J^2 = 0.92 Hz, 1H), 7.12 (d, *J* = 8.24 Hz, 1H), 5.77 (d, *J* = 3.20 Hz, 1H), 4.75 (d, *J* = 6.88 Hz, 1H), 4.14 (dd, J^1 = 2.40 Hz, J^2 = 6.84 Hz, 1H), 3.39 (s, 3H), 2.40 (s, 3H).

^{13}C NMR ($CDCl_3$, 100 MHz) δ 165.1, 147.4, 144.2, 143.7, 139.1, 135.4, 130.3, 130.1, 129.8,

129.6, 127.4, 125.8, 125.1, 123.7, 116.2, 55.7, 46.7, 31.0, 21.7.

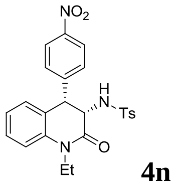

4n

***N*-((3*S*,4*S*)-1-Ethyl-4-(4-nitrophenyl)-2-oxo-1,2,3,4-tetrahydroquinolin-3-yl)-4-methylbenzenesulfonamide (4n)**: Starting with **3n** (0.1 mmol, 57 mg) and purified by p-TLC (silica gel, petroleum ether/EtOAc 10:1). Yield: 36.3 mg (78%).

Orange solid, mp: 89 - 91°C.

TLC (petroleum ether/EtOAc 5:1): R_f = 0.20.

HRMS (*m*/*z*): calcd for $C_{24}H_{23}N_3O_5SH^+$ $[M+H]^+$, 466.1431; found, 466.1433.

^{1}H NMR ($CDCl_3$, 600 MHz) δ 8.09 (d, *J* = 8.94 Hz, 2H), 7.75 (d, *J* = 8.28 Hz, 2H), 7.41 (td, J^1 = 7.56 Hz, J^2 = 1.38 Hz, 1H), 7.29 (d, *J* = 8.22 Hz, 2H), 7.26-7.24 (m, 3H), 7.16-7.14 (m, 2H), 5.79 (d, *J* = 3.42 Hz, 1H), 4.73 (d, *J* = 6.90 Hz, 1H), 4.16 (dd, J^1 = 3.42 Hz, J^2 = 6.84 Hz, 1H), 4.06-4.03 (m, 1H), 3.94-3.90 (m, 1H), 2.40 (s, 3H), 1.23 (t, *J* = 7.56 Hz, 3H).

^{13}C NMR ($CDCl_3$, 150 MHz) δ 164.7, 147.5, 144.1, 143.7, 137.9, 135.6, 130.4, 130.2, 130.1, 129.6, 127.4, 126.1, 124.9, 123.5, 116.0, 55.6, 46.7, 38.7, 21.7, 12.6.

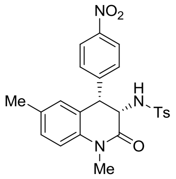

4o

***N*-((3*S*,4*S*)-1,6-Dimethyl-4-(4-nitrophenyl)-2-oxo-1,2,3,4-tetrahydroquinolin-3-yl)-4-methylbenzenesulfonamide (4o)**: Starting with **3o** (0.1 mmol, 57 mg) and purified by p-TLC (silica gel, petroleum ether/EtOAc 10:1). Yield: 34.4 mg (74%).

Orange solid, mp: 209 - 211°C.

TLC (petroleum ether/EtOAc 5:1): R_f = 0.19.

HRMS (*m*/*z*): calcd for $C_{24}H_{23}N_3O_5SH^+$ $[M+H]^+$, 466.1431; found, 466.1429.

^{1}H NMR ($CDCl_3$, 600 MHz) δ 8.10 (d, *J* = 8.94 Hz, 2H), 7.74 (d, *J* = 8.22 Hz, 2H), 7.28 (d, *J* = 8.28 Hz, 2H), 7.23 (d, *J* = 8.94 Hz, 2H), 7.20 (d, *J* = 7.56 Hz, 1H), 7.05 (s, 1H), 7.00 (d, *J* = 8.28 Hz, 1H), 5.77 (d, *J* = 3.42 Hz, 1H), 4.69 (d, *J* = 6.84 Hz, 1H), 4.11 (dd, J^1 = 3.48 Hz, J^2 = 6.90 Hz, 1H), 3.36 (s, 3H), 2.40 (s, 3H), 2.31 (s, 3H);

^{13}C NMR ($CDCl_3$, 150 MHz) δ 165.0, 147.4, 144.1, 143.9, 136.7, 135.6, 135.0, 130.3, 130.1, 127.4, 125.7, 123.7, 116.1, 55.8, 46.8, 31.0, 21.7, 20.7.

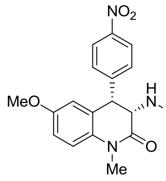

4p

***N*-((*3S,4S*)-6-Methoxy-1-methyl-4-(4-nitrophenyl)-2-oxo-1,2,3,4-tetrahydroquinolin-3-yl)-4-methylbenzenesulfonamide (4p)**: Starting with **3p** (0.1 mmol, 58 mg) and purified by p-TLC (silica gel, petroleum ether/EtOAc 5:1). Yield: 34.4 mg (69%).

Orange solid, mp: 109 - 111°C.

TLC (petroleum ether/EtOAc 5:1): R_f = 0.10.

HRMS (*m/z*): calcd for $C_{24}H_{23}N_3O_6SH^+$ $[M+H]^+$, 482.1380; found, 482.1381.

^1H NMR ($CDCl_3$, 600 MHz) δ 8.11 (d, J = 8.22 Hz, 2H), 7.74 (d, J = 8.28 Hz, 2H), 7.29 (d, J = 8.22 Hz, 2H), 7.24 (d, J = 8.94 Hz, 2H), 7.04 (d, J = 8.94 Hz, 1H), 6.93 (dd, J^1 = 2.76 Hz, J^2 = 8.94 Hz, 1H), 6.78 (d, J = 2.70 Hz, 1H), 5.78 (d, J = 3.48 Hz, 1H), 4.69 (d, J = 6.18 Hz, 1H), 4.10 (dd, J^1 = 3.48 Hz, J^2 = 6.90 Hz, 1H), 3.78 (s, 3H), 3.36 (s, 3H), 2.40 (s, 3H).

^{13}C NMR ($CDCl_3$, 150 MHz) δ 164.6, 156.8, 147.5, 144.2, 143.6, 135.5, 132.5, 130.4, 130.1, 127.4, 127.2, 123.7, 117.5, 114.89, 114.88, 55.77, 55.75, 47.0, 31.1, 21.7.

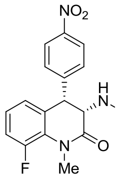

4q

***N*-((*3S,4S*)-8-Fluoro-1-methyl-4-(4-nitrophenyl)-2-oxo-1,2,3,4-tetrahydroquinolin-3-yl)-4-methylbenzenesulfonamide (4q)**: Starting with **3q** (0.1 mmol, 57 mg) and purified by p-TLC (silica gel, petroleum ether/EtOAc 10:1). Yield: 19.2 mg (41%).

Orange solid, mp: 163 - 165°C.

TLC (petroleum ether/EtOAc 5:1): R_f = 0.30.

HRMS (*m/z*): calcd for $C_{24}H_{23}N_3O_6SH^+$ $[M+H]^+$, 470.1180; found, 470.1174.

^1H NMR ($CDCl_3$, 400 MHz) δ 8.15 (d, J = 8.72 Hz, 2H), 7.73 (d, J = 8.24 Hz, 2H), 7.30 (d, J = 7.80 Hz, 2H), 7.27-7.25 (m, 2H), 7.19-7.15 (m, 2H), 7.07-7.05 (m, 1H), 5.73 (d, J = 3.64 Hz, 1H), 4.72 (d, J = 6.40 Hz, 1H), 4.11 (dd, J^1 = 3.68 Hz, J^2 = 5.96 Hz, 1H), 3.43-3.42 (d, J = 5.52 Hz, 3H),

2.41 (s, 3H).

^{13}C NMR ($CDCl_3$, 100 MHz) δ 165.7, 152.7 (d, J = 247.89 Hz), 147.7, 144.3, 142.7, 135.5, 130.22, 130.19, 129.9, 128.0 (d, J = 7.63 Hz), 127.3, 126.7 (d, J = 7.62 Hz), 125.0 (d, J = 2.86 Hz), 123.8, 117.8 (d, J = 21.0 Hz), 55.7, 47.0, 34.4 (d, J = 10.5 Hz), 21.7.

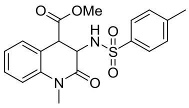

6a

Methyl 1-methyl-3-(4-methylphenylsulfonamido)-2-oxo-1,2,3,4-tetrahydroquinoline-4-carboxylate (6a): Starting with **5a** (0.1 mmol, 49 mg) and purified by p-TLC (silica gel, petroleum ether/EtOAc 5:1 to give pure *cis*-**6a** and crude *trans*-**6a** which was future purified by p-TLC (dichloromethane)). Yield: 70%.

cis-**6a**: 14.0 mg (36%), white solid, mp: 203 - 205°C.

TLC (petroleum ether/EtOAc 1:1): R_f = 0.65.

HRMS (*m*/*z*): calcd for $C_{19}H_{20}N_2O_5SH^+$ $[M+H]^+$, 389.1166; found, 389.1164.

^{1}H NMR ($CDCl_3$, 400 MHz) δ 7.77 (d, J = 8.2 Hz, 2H), 7.32 - 7.35 (m, 2H), 7.28 (d, J = 8.2 Hz, 2H), 7.10 (t, J = 7.3 Hz, 1H), 6.96 (d, J = 8.2 Hz, 1H), 6.11 (d, J = 4.1 Hz, 1H), 4.26 (d, J = 6.0 Hz, 1H), 3.95 (dd, J = 6.0 Hz, 4.6 Hz, 1H), 3.65 (s, 3H), 3.31 (s, 3H), 2.40 (s, 3H).

^{13}C NMR ($CDCl_3$, 100 MHz) δ 170.0, 165.8, 143.9, 139.4, 135.8, 130.0, 129.84, 129.77, 127.5, 124.1, 121.2, 115.9, 52.9, 52.7, 48.8, 30.9, 21.7.

trans-**6a**: 13.2 mg (34%), white solid, mp: 125 - 127°C.

TLC (petroleum ether/EtOAc 1:1): R_f = 0.46.

HRMS (*m*/*z*): calcd for $C_{19}H_{20}N_2O_5SH^+$ $[M+H]^+$, 389.1166; found, 389.1177.

^{1}H NMR ($CDCl_3$, 400 MHz) δ 7.77 (d, J = 8.2 Hz, 2H), 7.32 - 7.36 (m, 1H), 7.28 (d, J = 8.2 Hz, 2H), 7.08 - 7.10 (m, 2H), 6.98 (d, J = 8.2 Hz, 1H), 5.56 (d, J = 6.9 Hz, 1H), 4.34 (dd, J = 10.8 Hz, 6.9 Hz, 1H), 4.01 (d, J = 10.8 Hz, 1H), 3.74 (s, 3H), 3.31 (s, 3H), 2.41 (s, 3H).

^{13}C NMR ($CDCl_3$, 100 MHz) δ 170.6, 166.0, 143.9, 138.8, 136.4, 129.7, 129.4, 128.4, 127.8, 124.3, 121.3, 115.7, 54.3, 52.8, 50.0, 31.0, 21.7.

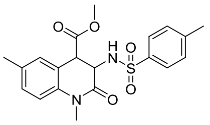

6b

Methyl 1,6-dimethyl-3-(4-methylphenylsulfonamido)-2-oxo-1,2,3,4-tetrahydroquino

line-4-carboxylate (6b): Starting with **5b** (0.1 mmol, 50 mg) and purified by p-TLC (silica gel, petroleum ether/EtOAc 5:1 for the first time then dichloromethane for the future purification). Yield: 62%.

cis-**6b**: 12.9 mg (32%), yellow solid, mp: 233 - 235°C.

TLC (petroleum ether/EtOAc 1:1): R_f = 0.70.

HRMS (*m/z*): calcd for $C_{20}H_{22}N_2O_5SH^+$ $[M+H]^+$, 403.1322; found, 403.1327.

^{1}H NMR ($CDCl_3$, 600 MHz) δ 7.76 (d, *J* = 8.2 Hz, 2H), 7.28 (d, *J* = 8.2 Hz, 2H), 7.11 - 7.14 (m, 2H), 6.84 (d, *J* = 7.6 Hz, 1H), 6.10 (d, *J* = 4.1 Hz, 1H), 4.20 (d, *J* = 5.5 Hz, 1H), 3.91 (dd, *J* = 5.5 Hz, 4.8 Hz, 1H), 3.65 (s, 3H), 3.29 (s, 3H), 2.39 (s, 3H),2.32 (s, 3H).

^{13}C NMR ($CDCl_3$, 150 MHz) δ 170.1, 165.6, 143.9, 137.1, 136.0, 134.0, 130.4, 130.2, 130.0, 127.5, 121.1, 115.9, 53.0, 52.7, 48.9, 30.9, 21.7, 20.7.

trans-**6b**: 12.1 mg (30%), yellow solid, mp: 206 – 208 °C.

TLC (petroleum ether/EtOAc 1:1): R_f = 0.52.

HRMS (*m/z*): calcd for $C_{19}H_{20}N_2O_5SH^+$ $[M+H]^+$, 403.1322; found, 403.1324.

^{1}H NMR ($CDCl_3$, 600 MHz) δ 7.77 (d, *J* = 8.2 Hz, 2H), 7.29 (d, *J* = 8.2 Hz, 2H), 7.13 (d, *J* = 8.2 Hz, 1H), 6.86 - 6.87 (m, 2H), 5.45 (d, *J* = 7.6 Hz, 1H), 4.30 (dd, *J* = 10.7 Hz, 6.9 Hz, 1H), 4.00 (d, *J* = 10.3 Hz, 1H), 3.76 (s, 3H), 3.28 (s, 3H), 2.41 (s, 3H), 2.30 (s, 3H).

^{13}C NMR ($CDCl_3$, 150 MHz) δ 170.7, 165.7, 143.9, 136.5, 136.4, 134.1, 129.9, 129.7, 129.1, 127.8, 121.1, 115.6, 54.4, 52.8, 50.1, 31.0, 21.7, 20.8.

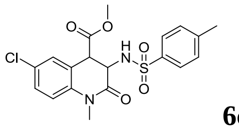

6c

Methyl 6-chloro-1-methyl-3-(4-methylphenylsulfonamido)-2-oxo-1,2,3,4-tetrahydroquinoline-4-carboxylate (6c): Starting with **5c** (0.1 mmol, 52 mg) and purified by p-TLC (silica gel, petroleum ether/EtOAc 5:1 for the first time then dichloromethane for the future purification). Just *trans*-**6c** was isolated. Yield: 52%.

trans-**6c**: 21.9 mg (52%), yellow solid, mp: 186 – 188 °C.

TLC (petroleum ether/EtOAc 1:1): R_f = 0.51.

HRMS (*m/z*): calcd for $C_{19}H_{19}ClN_2O_5SH^+$ $[M+H]^+$, 423.0776; found, 423.0777.

^{1}H NMR ($CDCl_3$, 600 MHz) δ 7.76 (d, *J* = 8.2 Hz, 2H), 7.28 - 7.31 (m, 3H), 7.05 (d, *J* = 2.3 Hz,

1H), 6.91 (d, *J* = 8.7 Hz, 1H), 5.49 (d, *J* = 6.9 Hz, 1H), 4.33 (dd, *J* = 10.3 Hz, 6.9 Hz, 1H), 4.00 (d, *J* = 10.0 Hz, 1H), 3.75 (s, 3H), 3.29 (s, 3H), 2.42 (s, 3H).

^{13}C NMR ($CDCl_3$, 150 MHz) δ 170.0, 165.7, 144.4, 137.6, 136.4, 129.8, 129.6, 129.3, 128.6, 127.7, 122.9, 116.8, 54.2, 53.0, 49.6, 31.1, 21.7.

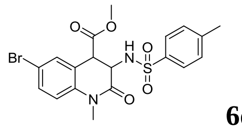

6d

Methyl 6-bromo-1-methyl-3-(4-methylphenylsulfonamido)-2-oxo-1,2,3,4-tetrahydroquinoline-4-carboxylate (6d): Starting with **5d** (0.1 mmol, 57 mg) and purified by p-TLC (silica gel, petroleum ether/EtOAc 5:1 for the first time then dichloromethane for the future purification). Just *trans*-**6d** was isolated. Yield: 55%.

trans-**6d**: 25.6 mg (55%), yellow solid, mp: 202 – 204 °C.

TLC (petroleum ether/EtOAc 1:1): R_f = 0.52.

HRMS (*m*/*z*): calcd for $C_{19}H_{19}BrN_2O_5SH^+$ $[M+H]^+$, 467.0271; found, 467.0268.

^{1}H NMR ($CDCl_3$, 600 MHz) δ 7.75 (d, *J* = 8.3 Hz, 2H), 7.44 (dd, *J* = 8.3 Hz, 2.1 Hz, 1H),7.29 (d, *J* = 8.3 Hz, 2H), 7.19 (s, 1H), 6.85 (d, *J* = 8.9 Hz, 1H), 5.49 (d, *J* = 6.9 Hz, 1H), 4.33 (dd, *J* = 10.3 Hz, 6.9 Hz, 1H), 4.00 (d, *J* = 10.3 Hz, 1H), 3.74 (s, 3H), 3.28 (s, 3H), 2.42 (s, 3H).

^{13}C NMR ($CDCl_3$, 150 MHz) δ 169.9, 165.6, 144.0, 138.1, 136.5, 132.3, 131.4, 129.8, 127.7, 123.1, 117.2, 117.0, 54.2, 53.0, 49.5, 31.0, 21.7.

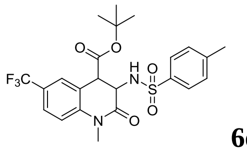

6e

***tert*-Butyl 1-methyl-3-(4-methylphenylsulfonamido)-2-oxo-6-(trifluoromethyl)-1,2,3,4-tetrahydroquinoline-4-carboxylate (6e)**: Starting with **5e** (0.1 mmol, 60 mg) and purified by p-TLC (silica gel, petroleum ether/EtOAc 5:1 for the first time then dichloromethane for the future purification). Just *trans*-**6e** was isolated. Yield: 58%.

trans-**6e**: 28.9 mg (58%), yellow solid, mp: 214 – 216 °C.

TLC (petroleum ether/EtOAc 1:1): R_f = 0.68.

HRMS (*m*/*z*): calcd for $C_{19}H_{17}F_3N_2O_5SH^+$ $[M-C_4H_8+H]^+$, 443.0883; found, 443.0885.

^{1}H NMR ($CDCl_3$, 400 MHz) δ 7.75 (d, *J* = 8.2 Hz, 2H), 7.58 (d, *J* = 7.8 Hz, 1H), 7.41 (s, 1H),

7.29 (d, J = 8.2 Hz, 2H), 7.06 (d, J = 8.7 Hz, 1H), 5.26 (d, J = 8.2 Hz, 1H), 4.49 (dd, J = 9.4 Hz, 8.2 Hz, 1H), 3.90 (d, J = 9.6 Hz, 1H), 3.29 (s, 3H), 2.42 (s, 3H), 1.45 (s, 9H).

^{13}C NMR ($CDCl_3$, 100 MHz) δ 168.3, 166.3, 143.9, 141.9, 137.1, 129.7, 127.6, 126.4 (m), 126.0 (q, J = 33.6 Hz), 125.5 (m), 123.8 (q, J = 271.3 Hz), 122.4, 115.6, 83.7, 54.3, 50.2, 30.8, 27.9, 21.7.

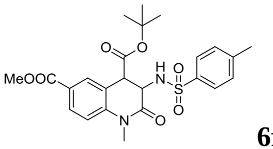

6f

***4-tert*-Butyl 6-methyl 1-methyl-3-(4-methylphenylsulfonamido)-2-oxo-1,2,3,4-tetrahydroquinoline-4,6-dicarboxylate (6f)**: Starting with **5f** (0.1 mmol, 59 mg) and purified by p-TLC (silica gel, petroleum ether/EtOAc 5:1 for the first time then dichloromethane for the future purification). Just *trans*-**6f** was isolated. Yield: 56%.

trans-**6f**: 27.3 mg (56%), yellow solid, mp: 217 – 219 °C.

TLC (petroleum ether/EtOAc 1:1): R_f = 0.56.

HRMS (*m*/*z*): calcd for $C_{20}H_{20}N_2O_7SH^+$ $[M-C_4H_8+H]^+$, 433.1064; found, 433.1067.

^{1}H NMR ($CDCl_3$, 400 MHz) δ 8.01 (dd, J = 8.5 Hz, 1.8 Hz, 1H), 7.88 (s, 1H), 7.77 (d, J = 8.2 Hz, 2H), 7.29 (d, J = 8.2 Hz, 2H), 7.02 (d, J = 8.2 Hz, 1H), 5.18 (d, J = 8.2 Hz, 1H), 4.49 (dd, J = 9.2 Hz, 8.7 Hz, 1H), 3.91 (s, 3H), 3.88 (d, J = 10.1 Hz, 1H), 3.30 (s, 3H), 2.42 (s, 3H), 1.47 (s, 9H).

^{13}C NMR ($CDCl_3$, 100 MHz) δ 168.5, 166.6, 166.1, 143.8, 142.7, 137.1, 130.9, 129.8, 129.7, 127.6, 125.6, 121.8, 115.3, 83.5, 54.4, 52.4, 50.2, 30.8, 28.0, 21.7.

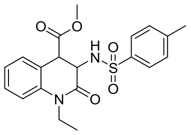

6g

Methyl 1-ethyl-3-(4-methylphenylsulfonamido)-2-oxo-1,2,3,4-tetrahydroquinoline-4-carboxylate (6g): Starting with **5g** (0.1 mmol, 50 mg) and purified by p-TLC (silica gel, petroleum ether/EtOAc 5:1 to give pure *cis*-**6g** and crude *trans*-**6g** which was future purified by p-TLC (dichloromethane)). Yield: 74%.

cis-**6g**: 14.8 mg (37%), yellow solid, mp: 72 – 74 °C.

TLC (petroleum ether/EtOAc 1:1): R_f = 0.71.

HRMS (*m*/*z*): calcd for $C_{20}H_{22}N_2O_5SH^+$ $[M+H]^+$, 403.1322; found, 403.1326.

^1H NMR ($CDCl_3$, 600 MHz) δ 7.77 (d, *J* = 8.2 Hz, 2H), 7.31 - 7.33 (m, 2H), 7.28 (d, *J* = 8.2 Hz, 2H), 7.08 (t, *J* = 7.6 Hz, 1H), 7.00 (d, *J* = 8.9 Hz, 1H), 6.11 (d, *J* = 4.1 Hz, 1H), 4.22 (d, *J* = 5.5 Hz, 1H), 3.95 - 4.02 (m, 2H), 3.83 - 3.89 (m, 1H), 3.64 (s, 3H), 2.40 (s, 3H), 1.13 (t, *J* = 6.9 Hz, 3H).

^{13}C NMR ($CDCl_3$, 150 MHz) δ 169.9, 165.3, 143.9, 138.2, 136.1, 130.2, 129.9, 129.8, 127.5, 124.1, 121.7, 116.0, 52.9, 52.6, 49.0, 38.6, 21.7, 12.4.

trans-**6g**: 14.8 mg (37%), yellow solid, mp: 157 – 159 ^oC.

TLC (petroleum ether/EtOAc 1:1): R_f = 0.60.

HRMS (*m*/*z*): calcd for $C_{20}H_{22}N_2O_5SH^+$ $[M+H]^+$, 403.1322; found, 403.1324.

^1H NMR ($CDCl_3$, 600 MHz) δ 7.77 (d, *J* = 8.2 Hz, 2H), 7.31 - 7.34 (m, 1H), 7.29 (d, *J* = 8.2 Hz, 2H), 7.07 - 7.08 (m, 2H), 7.01 (d, *J* = 8.3 Hz, 1H), 5.48 (d, *J* = 7.6 Hz, 1H), 4.34 (dd, *J* = 10.3 Hz, 7.6 Hz, 1H), 3.91 - 3.99 (m, 2H), 3.81 - 3.87 (m, 1H), 3.73 (s, 3H), 2.41 (s, 3H), 1.13 (d, *J* = 6.9 Hz, 3H).

^{13}C NMR ($CDCl_3$, 150 MHz) δ 170.5, 165.4, 143.8, 137.8, 136.6, 129.7, 129.4, 128.7, 127.8, 124.2, 121.6, 115.6, 54.3, 52.8, 50.2, 38.9, 21.7, 12.6.

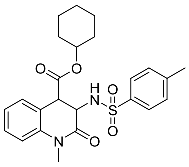

6h

Cyclohexyl 1-methyl-3-(4-methylphenylsulfonamido)-2-oxo-1,2,3,4-tetrahydroquinoline-4-carboxylate (6h): Starting with **5h** (0.1 mmol, 56 mg) and purified by p-TLC (silica gel, petroleum ether/EtOAc 10:1 to give pure *cis*-**6h** and crude *trans*-**6h** which was future purified by p-TLC (dichloromethane)). Yield: 69%.

cis-**6h**: 16.0 mg (35%), yellow solid, mp: 172 – 174 ^oC.

TLC (petroleum ether/EtOAc 1:1): R_f = 0.80.

HRMS (*m*/*z*): calcd for $C_{24}H_{28}N_2O_5SH^+$ $[M+H]^+$, 457.1792; found, 457.1796.

^1H NMR ($CDCl_3$, 400 MHz) δ 7.77 (d, *J* = 8.2 Hz, 2H), 7.26 - 7.35 (m, 4H), 7.09 (dt, *J* = 7.3 Hz, 0.9 Hz, 1H), 6.95 (d, *J* = 8.2 Hz, 1H), 6.09 (d, *J* = 4.1 Hz, 1H), 4.65 - 4.75 (m, 1H), 4.21 (d, *J* = 6.0 Hz, 1H), 3.95 (dd, *J* = 5.7 Hz, 4.1 Hz, 1H), 3.31 (s, 3H), 2.40 (s, 3H), 1.72 - 1.78 (m, 1H), 1.56 - 1.69 (m, 4H), 1.23 - 1.47 (m, 5H).

^{13}C NMR ($CDCl_3$, 100 MHz) δ 168.9, 165.9, 143.9, 139.3, 135.9, 1230.0, 129.8, 129.6, 127.5,

124.1, 121.6, 115.9, 74.1, 52.9, 49.3, 31.3, 31.2, 30.8, 25.3, 23.4, 21.7.

trans-**6h**: 15.5 mg (34%), white solid, mp: 139 – 141 °C.

TLC (petroleum ether/EtOAc 1:1): R_f = 0.60;

HRMS (*m*/*z*): calcd for $C_{24}H_{28}N_2O_5SH^+$ $[M+H]^+$, 457.1792; found, 457.1796.

^{1}H NMR ($CDCl_3$, 600 MHz) δ 7.77 (d, *J* = 8.2 Hz, 2H), 7.34 (t, *J* = 7.6 Hz, 1H), 7.28 (d, *J* = 8.2 Hz, 2H), 7.13 (d, *J* = 7.6 Hz, 1H), 7.09 (t, *J* = 7.6 Hz, 1H), 6.98 (d, *J* = 8.2 Hz, 1H), 5.31 (d, *J* = 7.6 Hz, 1H), 4.68 - 4.84 (m, 1H), 4.42 (dd, *J* = 9.6 Hz, 7.6 Hz, 1H), 3.94 (d, *J* = 10.3 Hz, 1H), 3.29 (s, 3H), 2.41 (s, 3H), 1.82 - 1.88 (m, 2H), 1.66 - 1.82 (m, 2H), 1.24 - 1.64 (m, 6H).

^{13}C NMR ($CDCl_3$, 150 MHz) δ 169.6, 166.1, 143.8, 139.0, 136.9, 129.7, 129.3, 128.6, 127.7, 124.2, 121.7, 115.6, 74.7, 54.5, 50.0, 31.6, 31.5, 30.8, 25.4, 23.8, 21.7.

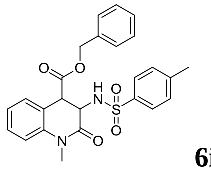

6i

Benzyl 1-methyl-3-(4-methylphenylsulfonamido)-2-oxo-1,2,3,4-tetrahydroquinoline-4-carboxylate (6i): Starting with **5i** (0.1 mmol, 57 mg) and purified by p-TLC (silica gel, petroleum ether/EtOAc 10:1 to give pure *cis*-**6i** and crude *trans*-**6i** which was future purified by p-TLC (dichloromethane)). Yield: 72%.

cis-**6i**: 17.1 mg (37%), yellow solid, mp: 143 – 145 °C.

TLC (petroleum ether/EtOAc 1:1): R_f = 0.75.

HRMS (*m*/*z*): calcd for $C_{25}H_{24}N_2O_5SH^+$ $[M+H]^+$, 465.1479; found, 465.1485.

^{1}H NMR ($CDCl_3$, 400 MHz) δ 7.76 (d, *J* = 8.2 Hz, 2H), 7.24 - 7.36 (m, 7H), 7.16 - 7.22 (m, 2H), 7.08 (d, *J* = 6.9 Hz, 1H), 6.94 (d, *J* = 7.8 Hz, 1H), 6.11 (d, *J* = 4.1 Hz, 1H), 5.12 (d, *J* = 12.4 Hz, 1H), 5.02 (d, *J* = 12.4 Hz, 1H), 4.30 (d, *J* = 5.9 Hz, 1H), 3.98 (dd, *J* = 5.5 Hz, 4.6 Hz, 1H), 3.25 (s, 3H), 2.38 (s, 3H).

^{13}C NMR ($CDCl_3$, 100 MHz) δ 169.3, 165.7, 143.9, 139.4, 135.9, 135.3, 130.0, 129.9, 129.8, 128.6, 128.4, 127.9, 127.5, 124.2, 121.1, 115.9, 67.2, 52.9, 49.0, 30.8, 21.7.

trans-**6i**: 16.2 mg (35%), white solid, mp: 205 – 207 °C.

TLC (petroleum ether/EtOAc 1:1): R_f = 0.55.

HRMS (*m*/*z*): calcd for $C_{25}H_{24}N_2O_5SH^+$ $[M+H]^+$, 465.1479; found, 465.1481.

^{1}H NMR ($CDCl_3$, 600 MHz) δ 7.75 (d, *J* = 8.2 Hz, 2H), 7.29 - 7.37 (m, 6H), 7.26 (d, *J* = 8.2 Hz, 2H), 7.01 - 7.04 (m, 2H), 6.96 (d, *J* = 8.2 Hz, 1H), 5.50 (d, *J* = 6.9 Hz, 1H), 5.23 (d, *J* = 12.4 Hz, 1H), 5.13 (d, *J* = 12.4 Hz, 1H), 4.35 (dd, *J* = 10.7, 6.9 Hz, 1H), 4.03 (d, *J* = 10.5 Hz, 1H), 3.28 (s, 3H), 2.40 (s, 3H).

^{13}C NMR ($CDCl_3$, 150 MHz) δ 170.0, 165.9, 143.9, 138.8, 136.4, 135.4, 129.7, 129.4, 128.72, 128.70, 128.6, 128.5, 127.8, 124.3, 121.2, 115.6, 67.7, 54.3, 50.1, 31.0, 21.7.

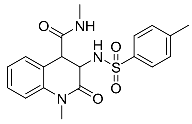

6j

***N*,1-Dimethyl-3-(4-methylphenylsulfonamido)-2-oxo-1,2,3,4-tetrahydroquinoline-4-carboxamide (6j)**: Starting with **5j** (0.1 mmol, 49 mg) and purified by p-TLC (silica gel, petroleum ether/EtOAc 1:1 to give pure *cis*-**6j** and crude *trans*-**6j** which was future purified by p-TLC (dichloromethane/MeOH 200:1)). Yield: 60%.

cis-**6j**: 12.7 mg (33%), yellow solid, mp: 215 – 216 °C.

TLC (petroleum ether/EtOAc 1:1): R_f = 0.21.

HRMS (*m/z*): calcd for $C_{19}H_{21}N_3O_4SH^+$ $[M+H]^+$, 388.1326; found, 388.1329.

^{1}H NMR ($CDCl_3$, 400 MHz) δ 7.77 (d, *J* = 7.3 Hz, 2H), 7.27 - 7.35 (m, 3H), 7.20 (d, *J* = 7.4 Hz, 1H), 7.06 (t, *J* = 7.8 Hz, 1H), 6.97 (d, *J* = 8.2 Hz, 1H), 6.28 (d, *J* = 2.3 Hz, 1H), 6.05 (q, *J* = 3.6 Hz, 1H), 4.03 (d, *J* = 5.8 Hz, 1H), 3.86 (qq, *J* = 5.7 Hz, 3.6 Hz, 1H), 3.34 (s, 3H), 2.79 (d, *J* = 4.1 Hz, 3H), 2.40 (s, 3H).

^{13}C NMR ($CDCl_3$, 100 MHz) δ 169.9, 165.5, 144.2, 139.9, 135.3, 130.1, 129.5, 128.9, 127.5, 123.9, 122.7, 116.2, 53.4, 50.0, 31.0, 26.6, 21.7.

trans-**6j**: 10.4 mg (27%), yellow solid, mp: 236 – 238 °C.

TLC (petroleum ether/EtOAc 1:1): R_f = 0.08.

HRMS (*m/z*): calcd for $C_{19}H_{21}N_3O_4SH^+$ $[M+H]^+$, 388.1326; found, 388.1325.

^{1}H NMR ($CDCl_3$, 600 MHz) δ 7.76 (d, *J* = 8.2 Hz, 2H), 7.32 (t, *J* = 7.6 Hz, 1H), 7.27 (d, *J* = 8.2 Hz, 2H), 7.17 (t, *J* = 7.6 Hz, 1H), 7.09 (t, *J* = 7.5 Hz, 1H), 6.97 (d, *J* = 8.2 Hz, 1H), 6.13 - 6.18 (m, 1H), 5.65 (d, *J* = 7.6 Hz, 1H), 4.38 (dd, *J* = 11.0 Hz, 7.6 Hz, 1H), 3.83 (d, *J* = 11.0 Hz, 1H), 3.29 (s, 3H), 2.82 (d, *J* = 4.8 Hz, 3H), 2.40 (s, 3H).

^{13}C NMR ($CDCl_3$, 150 MHz) δ 169.7, 166.6, 143.9, 139.1, 136.4, 129.7, 129.3, 128.6, 127.8,

124.4, 122.5, 115.6, 54.7, 50.9, 31.0, 26.9, 21.7.

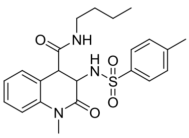

6k

***N*-Butyl-1-methyl-3-(4-methylphenylsulfonamido)-2-oxo-1,2,3,4-tetrahydroquinoline-4-carboxamide (6k)**: Starting with **5k** (0.1 mmol, 53 mg) and purified by p-TLC (silica gel, petroleum ether/EtOAc 1:1 to give pure *cis*-**6k** and crude *trans*-**6k** which was future purified by p-TLC (dichloromethane/MeOH 200:1)). Yield: 64%.

cis-**6k**: 15.0 mg (35%), yellow solid, mp: 164 – 166 ^{o}C.

TLC (petroleum ether/EtOAc 1:1): R_f = 0.49.

HRMS (*m/z*): calcd for $C_{22}H_{27}N_3O_4SH^+$ $[M+H]^+$, 430.1795; found, 430.1797.

^{1}H NMR ($CDCl_3$, 400 MHz) δ 7.76 (d, *J* = 8.2 Hz, 2H), 7.25 - 7.36 (m, 3H), 7.19 (d, *J* = 7.8 Hz, 1H), 7.06 (t, *J* = 7.4 Hz, 1H), 6.97 (d, *J* = 8.2 Hz, 1H), 6.26 - 6.29 (m, 1H), 6.03 - 6.06 (m, 1H), 4.01 (d, *J* = 6.4 Hz, 1H), 3.85 (dd, *J* = 6.0 Hz, 2.8 Hz, 1H), 3.33 (s, 3H), 3.22 - 3.30 (m, 1H), 3.09 - 3.18 (m, 1H), 2.39 (s, 3H), 1.44 - 1.51 (m, 2H), 1.25 - 1.36 (m, 2H), 0.90 (t, *J* = 7.3 Hz, 3H).

^{13}C NMR ($CDCl_3$, 100 MHz) δ 169.2, 165.5, 144.1, 140.0, 135.3, 130.1, 129.4, 128.8, 127.4, 123.9, 122.8, 116.1, 53.4, 50.1, 39.6, 31.6, 31.0, 21.7, 20.1, 13.8.

trans-**6k**: 12.4 mg (29%), yellow solid, mp: 209 - 211°C.

TLC (petroleum ether/EtOAc 1:1): R_f = 0.20;

HRMS (*m/z*): calcd for $C_{22}H_{27}N_3O_4SH^+$ $[M+H]^+$, 430.1795; found, 430.1794.

^{1}H NMR ($CDCl_3$, 600 MHz) δ 7.78 (d, *J* = 8.2 Hz, 2H), 7.33 (t, *J* = 7.6 Hz, 1H), 7.28 (d, *J* = 8.2 Hz, 2H), 7.20 (d, *J* = 8.2 Hz, 1H), 7.11 (t, *J* = 7.6 Hz, 1H), 6.98 (d, *J* = 8.2 Hz, 1H), 5.99 (t, *J* = 5.5 Hz, 1H), 5.46 (d, *J* = 6.9 Hz, 1H), 4.38 (dd, *J* = 10.3 Hz, 7.6 Hz, 1H), 3.79 (d, *J* = 11.0 Hz, 1H), 3.30 - 3.35 (m, 1H), 3.29 (s, 3H), 3.20 - 3.26 (m, 1H), 2.41 (s, 3H), 1.48 - 1.53 (m, 2H), 1.31 - 1.37 (m, 2H), 0.92 (t, *J* = 7.6 Hz, 3H).

^{13}C NMR ($CDCl_3$, 150 MHz) δ 168.7, 166.5, 143.9, 139.1, 136.5, 129.7, 129.3, 128.5, 127.8, 124.4, 122.7, 115.6, 54.6, 51.0, 40.0, 31.5, 31.0, 21.7, 20.2, 13.9.

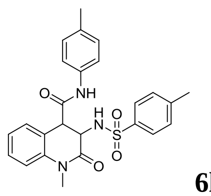

6l

1-Methyl-3-(4-methylphenylsulfonamido)-2-oxo-*N*-*p*-tolyl-1,2,3,4-tetrahydroquinoline-4-carboxamide (6l): Starting with **5l** (0.1 mmol, 56 mg) and purified by p-TLC (silica gel, petroleum ether/EtOAc 5:1 to give pure *cis*-**6l** and crude *trans*-**6l** which was future purified by p-TLC (dichloromethane)). Yield: 70%.

cis-**6l**: 25.9 mg (56%), yellow solid, mp: 264 - 266°C.

TLC (petroleum ether/EtOAc 1:1): R_f = 0.69.

HRMS (*m*/*z*): calcd for $C_{25}H_{25}N_3O_4SH^+$ $[M+H]^+$, 464.1639; found, 464.1639.

^{1}H NMR ($CDCl_3$, 600 MHz) δ 7.87 (br.s., 1H), 7.79 (d, *J* = 8.3 Hz, 2H), 7.34 (d, *J* = 8.3 Hz, 2H), 7.27 - 7.32 (m, 4H), 7.05 - 7.09 (m, 3H), 6.99 (d, *J* = 8.3 Hz, 1H), 6.33 - 6.34 (m, 1H), 4.23 (d, *J* = 6.9 Hz, 1H), 3.94 (dd, *J* = 6.2 Hz, 3.4 Hz, 1H), 3.35 (s, 3H), 2.38 (s, 3H), 2.27 (s, 3H).

^{13}C NMR ($CDCl_3$, 150 MHz) δ 167.2, 165.2, 144.3, 139.9, 135.2, 134.9, 134.5, 130.1, 129.6, 129.5, 129.1, 127.5, 124.1, 122.4, 120.4, 116.2, 53.5, 50.7, 31.0, 21.7, 21.0.

trans-**6l**: 6.5 mg (14%), yellow solid, mp: 230 - 232°C.

TLC (petroleum ether/EtOAc 1:1): R_f = 0.19.

HRMS (*m*/*z*): calcd for $C_{25}H_{25}N_3O_4SH^+$ $[M+H]^+$, 464.1639; found, 464.1635.

^{1}H NMR ($CDCl_3$, 600 MHz) δ 8.25 (br.s., 1H), 7.67 (d, *J* = 8.3 Hz, 2H), 7.33 (d, *J* = 8.3 Hz, 2H), 7.27 - 7.30 (m, 1H), 7.21 (d, *J* = 7.3 Hz, 1H),7.02 - 7.09 (m, 5H), 6.96 (t, *J* = 7.6 Hz, 1H), 6.21 - 6.24 (m, 1H), 4.60 (dd, *J* = 11.7 Hz, 8.4 Hz, 1H), 4.15 (d, *J* = 11.7 Hz, 1H),3.27 (s, 3H), 2.32 (s, 3H), 2.30 (s, 3H).

^{13}C NMR ($CDCl_3$, 150 MHz) δ 170.7, 167.5, 143.5, 139.2, 137.1, 135.2, 134.2, 129.6, 129.4, 129.1, 128.1, 127.5, 124.3, 120.4, 120.3, 115.6, 55.0, 51.0, 31.1, 21.7, 21.1.

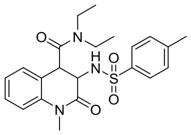

6m

***N*,*N*-Diethyl-1-methyl-3-(4-methylphenylsulfonamido)-2-oxo-1,2,3,4-tetrahydroquinoline-4-carboxamide (6m)**: Starting with **5m** (0.1 mmol, 53 mg) and purified by p-TLC (silica gel, petroleum ether/EtOAc 2:1 to give pure *cis*-**6m** and crude *trans*-**6m** which was future purified by

p-TLC (dichloromethane/MeOH 200:1)). Yield: 68%.

cis-**6m**: 14.6 mg (34%), yellow solid, mp: 202 - 204°C.

TLC (petroleum ether/EtOAc 1:1): R_f = 0.39.

HRMS (*m*/*z*): calcd for $C_{22}H_{27}N_3O_4SH^+$ $[M+H]^+$, 430.1795; found, 430.1800.

^{1}H NMR ($CDCl_3$, 600 MHz) δ 7.76 (d, *J* = 8.2 Hz, 2H), 7.26 - 7.30 (m, 3H), 7.19 (dd, *J* = 7.7, 1.1 Hz, 1H), 7.04 (t, *J* = 7.7 Hz, 1H), 6.96 (d, *J* = 7.7 Hz, 1H), 6.18 (d, *J* = 3.3 Hz, 1H), 4.57 (d, *J* = 6.0 Hz, 1H), 3.89 (dd, *J* = 6.0, 3.9 Hz, 1H), 3.72 - 3.79 (m, 1H), 3.61 - 3.67 (m, 1H), 3.39 - 3.45 (m, 1H), 3.33 (s, 3H), 3.25 - 3.32 (m, 1H), 2.38 (s, 3H), 1.39 (t, *J* = 7.1 Hz, 3H), 1.09 (t, *J* = 7.1 Hz, 3H).

^{13}C NMR ($CDCl_3$, 150 MHz) δ 169.2, 166.3, 143.9, 140.6, 135.6, 130.0, 129.2, 128.8, 127.4, 123.5, 123.2, 116.4, 54.1, 45.3, 42.6, 40.8, 31.0, 21.7, 15.1, 13.1.

trans-**6m**: 14.6 mg (34%), yellow solid, mp: 212 - 214°C.

TLC (petroleum ether/EtOAc 1:1): R_f = 0.23;

HRMS (*m*/*z*): calcd for $C_{22}H_{27}N_3O_4SH^+$ $[M+H]^+$, 430.1795; found, 430.1800.

^{1}H NMR ($CDCl_3$, 600 MHz) δ 7.82 (d, *J* = 8.3 Hz, 2H), 7.22 - 7.30 (m, 3H), 7.04 (t, *J* = 7.6 Hz, 1H), 6.93 - 6.98 (m, 2H), 5.77 (d, *J* = 7.8 Hz, 1H), 4.48 (dd, *J* = 11.7, 6.2 Hz, 1H), 4.31 (d, *J* = 12.0 Hz, 1H), 3.52 - 3.58 (m, 1H), 3.45 - 3.51 (m, 1H), 3.38 - 3.44 (m, 1H), 3.35 (s, 3H), 3.20 - 3.26 (m, 1H), 2.75 (s, 3H), 2.39 (s, 3H), 1.26 (t, *J* = 7.6 Hz, 3H), 1.19 (t, *J* = 7.6 Hz, 3H).

^{13}C NMR ($CDCl_3$, 150 MHz) δ 168.9, 167.7, 143.4, 139.4, 136.9, 129.4, 128.8, 128.0, 127.6, 124.2, 123.4, 115.4, 55.5, 45.4, 42.7, 41.3, 31.1, 21.7, 15.2, 13.3.

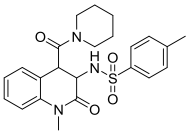

6n

4-Methyl-*N*-(1-methyl-2-oxo-4-(piperidine-1-carbonyl)-1,2,3,4-tetrahydroquinolin-3-yl)benzenesulfonamide (6n): Starting with **5n** (0.1 mmol, 54 mg) and purified by p-TLC (silica gel, petroleum ether/EtOAc 2:1 to give pure *cis*-**6n** and crude *trans*-**6n** which was future purified by p-TLC (dichloromethane/MeOH 200:1)). Yield: 69%.

cis-**6n**: 16.3 mg (37%), yellow solid, mp: 235 - 237°C.

TLC (petroleum ether/EtOAc 1:1): R_f = 0.37.

HRMS (*m*/*z*): calcd for $C_{23}H_{27}N_3O_4SH^+$ $[M+H]^+$, 442.1795; found, 442.1800.

^{1}H NMR ($CDCl_3$, 600 MHz) δ 7.76 (d, *J* = 8.2 Hz, 2H), 7.25 - 7.29 (m, 3H), 7.15 (d, *J* = 7.6 Hz, 1H), 7.03 (t, *J* = 7.6 Hz, 1H), 6.96 (d, *J* = 8.2 Hz, 1H), 6.20 (d, *J* = 3.4 Hz, 1H), 4.71 (d, *J* = 6.2 Hz, 1H), 3.82 - 3.89 (m, 2H), 3.70 - 3.75 (m, 1H), 3.62 - 3.67 (m, 1H), 3.38 - 3.43 (m, 1H), 3.33 (s, 3H), 2.39 (s, 3H), 1.81 - 1.86 (m, 1H), 1.70 - 1.74 (m, 1H), 1.55 - 1.66 (m, 3H), 1.45 - 1.51 (m, 1H).

^{13}C NMR ($CDCl_3$, 150 MHz) δ 168.1, 166.3, 143.9, 140.5, 135.7, 130.0, 129.2, 128.7, 127.4, 123.5, 123.1, 116.3, 53.8, 47.9, 44.4, 43.6, 30.9, 26.9, 25.8, 24.6, 21.6.

trans-**6n**: 14.1 mg (32%), yellow solid, mp: 230 - 232°C.

TLC (petroleum ether/EtOAc 1:1): R_f = 0.21.

HRMS (*m/z*): calcd for $C_{23}H_{27}N_3O_4SH^+$ $[M+H]^+$, 442.1795; found, 442.1797.

^{1}H NMR ($CDCl_3$, 600 MHz) δ 7.82 (d, *J* = 8.2 Hz, 2H), 7.24 - 7.30 (m, 3H), 7.01 - 7.07 (m, 2H), 6.97 (d, *J* = 8.2 Hz, 1H), 5.59 (d, *J* = 6.2 Hz, 1H), 4.50 (d, *J* = 11.0 Hz, 1H), 4.45 (dd, *J* = 11.0 Hz, 6.2 Hz, 1H), 3.53 - 3.65 (m, 4H), 3.33 (s, 3H), 2.40 (s, 3H), 1.52 - 1.76 (m, 6H).

^{13}C NMR ($CDCl_3$, 150 MHz) δ 168.0, 167.2, 143.5, 139.5, 137.0, 129.5, 128.9, 128.0, 127.9, 124.1, 123.0, 115.5, 55.6, 47.6, 45.1, 43.7, 30.9, 26.9, 25.7, 24.7, 21.7.

Evidence for radical process

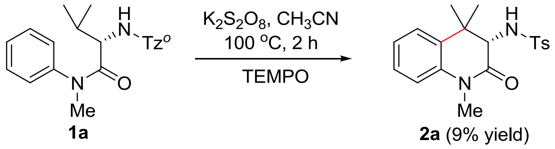

To a 25 mL Schlenk tube charged with a magnetic stirrer, **1a** (0.1 mmol, 46 mg), TEMPO (0.1 mmol, 15.6 mg), $K_2S_2O_8$ (0.2 mmol, 54 mg) and anhydrous MeCN (2.0 mL) were added. The tube was evacuated and back-filled with nitrogen for three cycles and then sealed. It was then placed in a preheated oil bath at 100 ℃ and the reaction was allowed to proceed for 2 hours. After completion of the reaction, the resulting mixture was filtered, and the filtrate was concentrated by a rotary evaporator. The residue was dissolved with EtOAc (3 mL), and the solution was washed with water (2 × 3 mL) and brine (2 × 3 mL), dried over $MgSO_4$, filtered and concentrated by a rotary evaporation. The residue was purified with preparative TLC (silica gel, petroleum ether/EtOAc or dichloromethane/MeOH). Yield: 9%.

Deuterium-labelling study

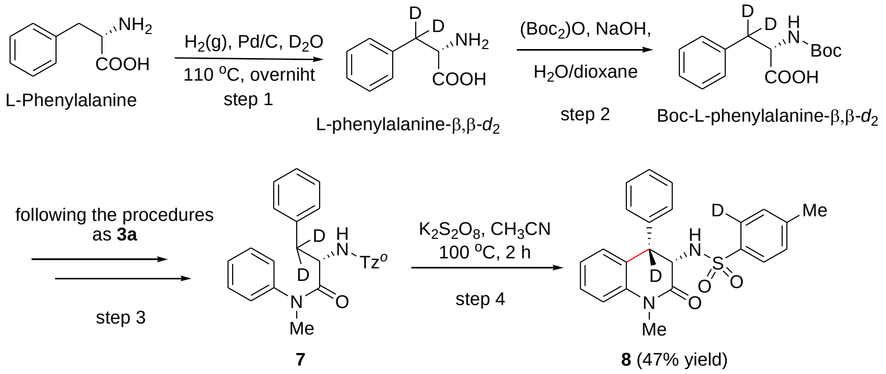

Step 1: L-phenylalanine-β,β-d_2 was synthesized through the H-D exchange reaction from L-phenylalanine according to the method described in the reference[4]. Deuterium efficiency (96%, same as shown in the reference[4]) was determined by ^{1}H NMR: (D_2O, 600 MHz) δ 7.45 (t, J = 7.6 Hz, 2H), 7.40 (t, J = 6.9 Hz, 1H), 7.35 (d, J = 6.9 Hz, 2H H), 4.00 (s, 1H), 3.29 (d, J = 4.8 Hz, 0.044H), 3.13 (d, J = 8.3 Hz, 0.039H).

Step 2: To L-phenylalanine-β,β-d_2 (167 mg, 1 mmol, 1 equiv) and NaOH (60 mg, 1.5 mmol, 1.5 equiv) in a mixed solvent (H_2O/dioxane 5 mL : 5 mL) was added Boc_2O (327 mg, 1.5 mmol, 1.5 equiv), then the reaction mixture was stirred at room temperature overnight. Dioxane was evaporated and the aqueous residue was washed with Et_2O (2 × 5 mL) and the organic phase was extracted with saturated $NaHCO_3$ (2 × 5 mL). The combined aqueous phase was acidified with 1 N $KHSO_4$, then extracted with EtOAc (3 × 5 mL). The combined organic phase was washed with brine, dried over Na_2SO_4 and concentrated to give Boc-L-phenylalanine-β,β-d_2 that was used for the next step directly.

Step 3: Following the procedures as **3a** to obtain **7**: Eluent: petroleum ether/ethyl acetate (4:1). Yield: 94 mg (92%). Yellow thick oil.

^{1}H NMR ($CDCl_3$, 400 MHz) δ 7.70 (d, J = 8.2 Hz, 1H), 7.42 (s, 1H), 7.16 - 7.23 (m, 4H), 7.11 (t, J = 7.3 Hz, 2H), 6.99 (d, J = 7.8 Hz, 1H), 6.90 - 6.93 (m, 2H), 6.59 (d, J = 8.7 Hz, 1H), 6.12 - 6.22 (m, 2H), 4.18 (d, J = 8.2 Hz, 1H), 3.79 - 3.99 (m, 4H), 2.93 (s, 3H), 2.39 (s, 3H), 1.37 - 1.45 (m, 6H).

^{13}C NMR ($CDCl_3$, 100 MHz) δ 170.3, 147.7, 143.8, 141.9, 136.1, 130.6, 129.6, 129.4, 128.3, 127.9, 127.8, 127.0, 126.8, 124.9, 118.1, 54.7, 49.8, 42.6, 40.3 (m), 37.3, 21.7, 14.5, 11.3.

^{2}H NMR ($CHCl_3$, 92.126 MHz) δ 2.84, 2.75.

Step 4: Following the procedures as **4a** to obtain **8**: Starting with **7** (0.1 mmol, 51 mg) and purified

by p-TLC (silica gel, petroleum ether/EtOAc 10:1). Yield: 19.2 mg (47%).

Yellow solid, mp: 117 - 119°C.

TLC (petroleum ether/EtOAc 5:1): R_f = 0.23.

HRMS (*m*/*z*): calcd for $C_{23}H_{20}D_2N_2O_3SH^+$ $[M+H]^+$, 409.1549; found, 409.1553.

^{1}H NMR ($CDCl_3$, 400 MHz) δ 7.75 (d, *J* = 8.7 Hz, 1H), 7.35 (td, *J* = 7.8 Hz, 1.4 Hz, 1H), 7.20 - 7.28 (m, 6H), 7.06 - 7.13 (m, 2H), 7.02 - 7.04 (m, 2H), 5.65 (d, *J* = 4.6 Hz, 1H), 4.17 (d, *J* = 4.1 Hz, 1H), 3.37 (s, 3H), 2.39 (s, 3H).

^{13}C NMR ($CDCl_3$, 100 MHz) δ 165.9, 143.8, 139.3, 136.2, 130.0, 129.8, 129.7, 129.2, 128.9, 128.7, 127.7, 127.6, 127.4, 124.7, 115.9, 56.1, 46.9(m), 30.9, 21.7.

^{2}H NMR ($CHCl_3$, 92.126 MHz) δ 7.81, 4.58.

The comparison of ^{1}H NMR of compounds **8** and **4a**, and ^{2}H NMR of compounds **7** and **8** was showed as follows:

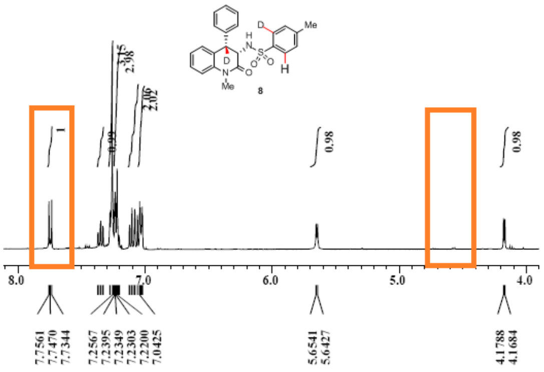

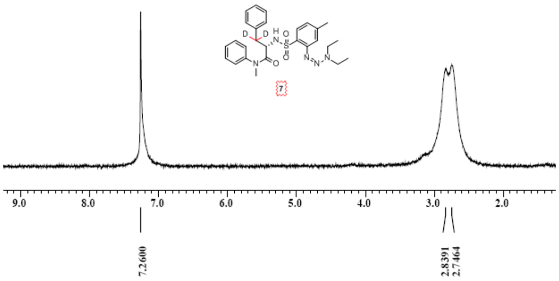

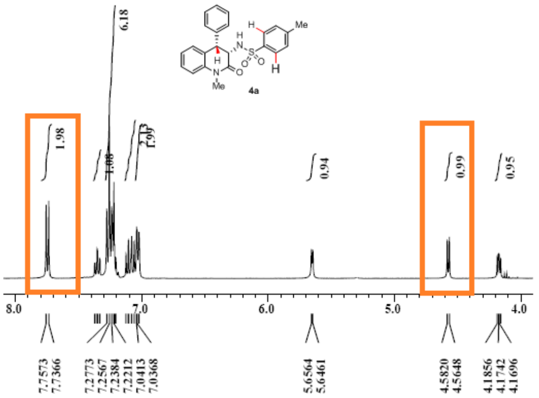

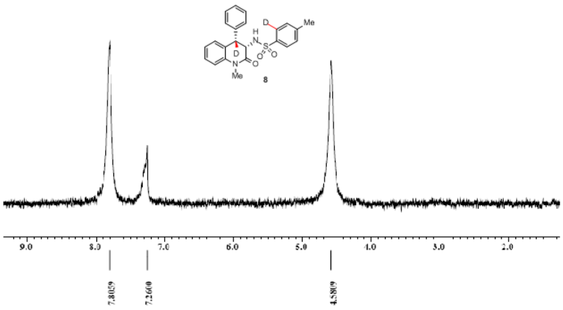

Application of the methods

a. Treatment of compound **9** under the standard conditions:

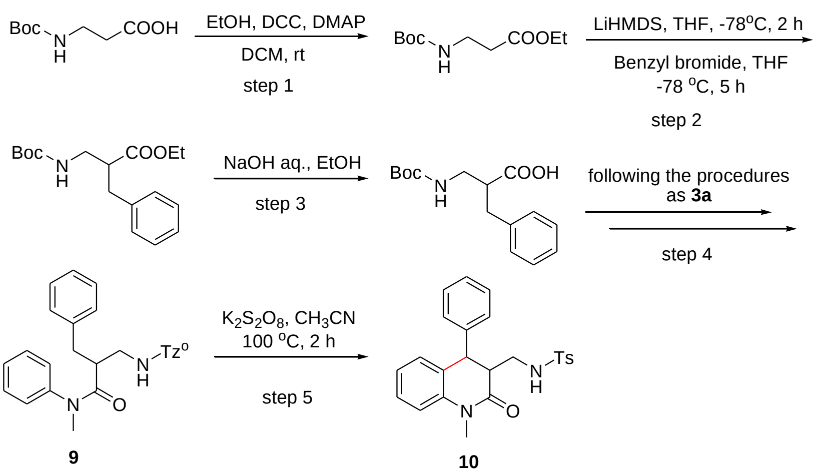

Step 1: To a stirred solution of 3-(*tert*-butoxycarbonylamino)propanoic acid (1.89 g, 10 mmol) in DCM (10 mL) was added DMAP (122 mg, 1mmol) and EtOH (1.75 mL, 30 mmol). DCC (2.27 g, 11 mmol) was added to the mixture at 0°C, which was then stirred for 5 min at 0°C and 3 hours at room temperature. Precipitated urea was then filtered off, and the filtrate evaporated down in vacuo. The residue was taken up in DCM and filtered free of any further precipitated urea. The DCM solution was washed twice with 0.5 N HCl and with saturated $NaHCO_3$ solution, then dried over $MgSO_4$, and concentrated. The residue was purified by silica gel chromatography (petroleum ether/EtOAc 10:1) to give ethyl 3-(*tert*-butoxycarbonylamino)propanoate (2.14 g, 99%) as a colourless oil.

Step 2: Following the method described in the reference[5]: To a stirred solution of ethyl 3-(*tert*-butoxycarbonylamino)propanoate (1.085 g, 5 mmol) in THF (10 mL) at -78°C under argon was added LiHMDS (1M, 6 mL, 6 mmol) over a 30 minute period. Then the reaction mixture was allowed to stir for 2 hours at the same temperature. After addition of benzyl bromide (0.71 mL, 6 mmol), the reaction mixture was stirred at -78°C for an additional 5 hours. The reaction mixture was quenched by addition of saturated NH_4Cl solution and then extracted with EtOAc (2 × 20 mL). The combined organic layer was dried over $MgSO_4$ and evaporated under reduced pressure to give an oily residue.

Step 3: To a solution of the residue in EtOH (20 mL) was added 1 N NaOH aqueous solution (20 mL) and the mixture was stirred at room temperature for 3 hours. After completion of the reaction, the solvent was concentrated and the residue was diluted with water (10 mL) and washed with EtOAc (3 × 10 mL). 1 N HCl aqueous solution was added to the aqueous phase to adjust pH to 5, and the solution was extracted with EtOAc (3 × 10 mL). The combined organic phase was washed

with brine (10 mL), dried over Na_2SO_4 and concentrated to give 2-benzyl-3-(*tert*-butoxycarbonylamino)propanoic acid (colourless solid, 1.33 g, 96% over two steps) which was used for the next step directly.

Step 4: Following the procedures as **3a** to obtain **9**: Eluent: petroleum ether/ethyl acetate (3:1). Yield: 100 mg (96%). Yellow thick oil.

^{1}H NMR ($CDCl_3$, 400 MHz) δ 7.68 (d, *J* = 7.8 Hz, 1H), 7.38 (s, 1H), 7.21 - 7.25 (m, 3H), 7.14 - 7.19 (m, 3H), 6.96 (d, *J* = 8.2 Hz, 1H), 6.77 - 6.83 (m, 2H), 6.52 - 6.63 (m, 2H), 6.07 (d, *J* = 7.3 Hz, 1H), 3.73 - 3.92 (m, 4H), 3.11 (s, 3H), 3.03 - 3.08 (m, 1H), 2.88 - 2.98 (m, 2H), 2.72 (dd, *J* = 13.1 Hz, 8.7 Hz, 1H), 2.49 (dd, *J* = 13.1 Hz, 5.5 Hz, 1H), 2.38 (s, 3H), 1.31 - 1.40 (m, 6H).

^{13}C NMR ($CDCl_3$, 100 MHz) δ 173.1, 147.5, 143.9, 143.0, 138.5, 129.5, 129.13, 129.07, 128.8, 128.3, 127.71, 127.66, 126.4, 125.3, 118.1, 49.9, 45.3, 44.7, 42.4, 37.2, 36.7, 21.7, 14.5, 11.2.

Step 5: Following the procedures as **4a** to obtain **10**: Starting with **9** (0.1 mmol, 52 mg) and purified by p-TLC (silica gel, petroleum ether/EtOAc 5:1). Yield: 16.8 mg (40%).

White solid, mp: 62 - 64°C.

TLC (petroleum ether/EtOAc 5:1): R_f = 0.11;

HRMS (*m/z*): calcd for $C_{24}H_{24}N_2O_3SH^+$ $[M+H]^+$, 421.1580; found, 421.1586;

^{1}H NMR ($CDCl_3$, 600 MHz) δ 7.59 (d, *J* = 8.2 Hz, 2H), 7.39 (t, *J* = 7.6 Hz, 2H), 7.35 (t, *J* = 7.6 Hz, 1H), 7.26 (t, *J* = 7.6 Hz, 1H), 7.24 (d, *J* = 8.2 Hz, 2H), 7.14 (d, *J* = 6.8 Hz, 2H), 7.02 (d, *J* = 8.2 Hz, 1H), 6.93 (t, *J* = 7.6 Hz, 1H), 6.59 (d, *J* = 7.6 Hz, 1H), 5.61 (t, *J* = 6.8 Hz, 1H), 4.08 (d, *J* = 13.1 Hz, 1H), 3.40 (s, 3H), 3.02 - 3.06 (m, 1H), 2.90 - 3.00 (m, 2H), 2.40 (s, 3H);

^{13}C NMR ($CDCl_3$, 150 MHz) δ 170.8, 143.3, 139.3, 139.0, 137.0, 129.8, 129.29, 129.28, 128.6, 128.1, 127.9, 127.2, 123.6, 114.9, 46.5, 44.5, 42.3, 30.1, 21.6.

Oxidation of compounds 4k with activated MnO_2

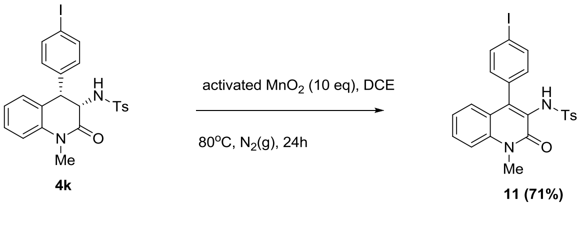

Following the method described in the reference[6]: **4k** (21.3 mg, 0.04 mmol) and activated MnO_2 (34.8 mg, 0.4 mmol), were added to an oven-dried side-arm tube equipped with a stirrer bar. The

tube was evacuated and refilled with argon five times. Then, anhydrous DCE (2 mL) was added via syringe. The reaction mixture was stirred at 80 °C for 24 hours then TLC showed the reaction completed. The resulting mixture was passed through a pad of Celite and eluted with DCM. The filtrate was concentrated under reduced pressure. The resulting residue was purified by p-TLC (petroleum ether/EtOAc 5:1) to give the product **11**.Yield: 12.7mg (71%).

White solid, mp: 234 - 236°C.

TLC (petroleum ether/EtOAc 1:1): R_f = 0.48;

HRMS (*m*/*z*): calcd for $C_{23}H_{19}IN_2O_3SH^+$ $[M+H]^+$, 531.0234; found, 531.0220;

^{1}H NMR ($CDCl_3$, 400 MHz) δ 7.53 - 7.58 (m, 3H), 7.36 - 7.41 (m, 3H), 7.22 - 7.24 (m, 1H), 7.13 - 7.17 (m, 3H), 6.98 (d, *J* = 8.2 Hz, 2H), 3.79 (s, 3H), 2.46 (s, 3H);

^{13}C NMR ($CDCl_3$, 100 MHz) δ 159.8, 143.3, 140.5, 138.0, 137.9, 137.4, 133.3, 132.1, 130.5, 129.3, 127.9, 126.6, 125.4, 123.0, 120.9, 114.5, 95.2, 30.8, 22.0.

Oxidation of *cis*-6k with activated MnO_2

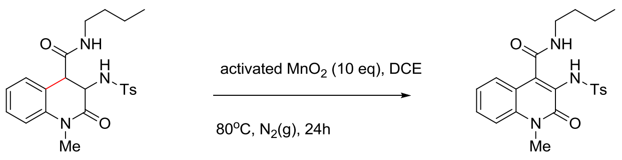

***cis*-6k** **12 (64%)**

Following the method described in the reference[6]: ***cis*-6k** (12.9 mg, 0.03 mmol) and activated MnO_2 (26.1 mg, 0.3 mmol), were added to an oven-dried side-arm tube equipped with a stirrer bar. The tube was evacuated and refilled with argon five times. Then, anhydrous DCE (2 mL) was added via syringe. The reaction mixture was stirred at 80 °C for 24 hours then TLC showed the reaction completed. The resulting mixture was passed through a pad of Celite and eluted with DCM. The filtrate was concentrated under reduced pressure. The resulting residue was purified by p-TLC (petroleum ether/EtOAc 2:1) to give the product **12**. Yield: 8.2 mg (64%).

Yellow solid, mp: 226 - 228°C.

TLC (petroleum ether/EtOAc 1:1): R_f = 0.22;

HRMS (*m*/*z*): calcd for $C_{22}H_{25}N_3O_3SH^+$ $[M+H]^+$, 428.1639; found, 428.1631;

^{1}H NMR ($CDCl_3$, 600 MHz) δ 7.89 (dd, *J* = 8.3 Hz, 1.4 Hz, 1H), 7.69 (d, *J* = 8.3 Hz, 2H), 7.59 (dt, *J* = 8.3 Hz, 1.4 Hz, 1H), 7.29 - 7.33 (m, 2H), 7.23 (d, *J* = 7.7 Hz, 2H), 7.12 (br.s., 1H), 6.94 (t, *J* = 5.5 Hz, 1H), 3.52 - 3.56 (m, 5H), 2.39 (s, 3H), 1.66 - 1.72 (m, 2H), 1.44 - 1.52 (m, 2H), 0.99 (t, *J*

= 7.6 Hz, 3H);

^{13}C NMR ($CDCl_3$, 150 MHz) δ 164.0, 159.2, 144.4, 139.8, 138.4, 136.4, 131.2, 129.6, 128.2, 127.8, 123.6, 122.1, 118.3, 114.4, 40.1, 31.4, 30.6, 21.8, 20.4, 13.9.

X-ray crystallographic data for 2k

Metrical parameters for the crystal structures of compound **2k** are available free from the Cambridge Crystallographic Data Centre under reference number CCDC 1415446.

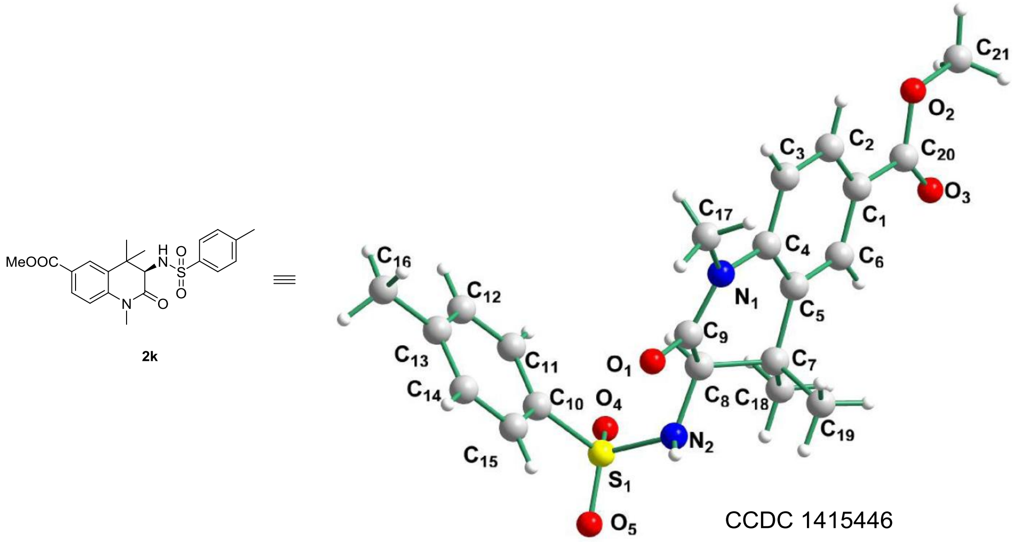

Table S1. Crystal data and structure refinement for CCDC 1415446.

Identification code	CCDC 1415446
Empirical formula	C21 H24 N2 O5 S1
Formula weight	416.4907
Temperature	153(2) K
Wavelength	0.71073 A
Crystal system, space group	?, ?
Unit cell dimensions	a = 10.8044(11) A alpha = 90 deg.
	b = 10.8044(11) A beta = 90 deg.
	c = 36.526(5) A gamma = 90 deg.
Volume	4263.9(9) A^3
Z, Calculated density	55, 1.298 Mg/m^3
Absorption coefficient	0.186 mm^-1

F(000)	1760
Crystal size	0.35 x 0.33 x 0.22 mm
Theta range for data collection	2.23 to 29.11 deg.
Limiting indices	-14<=h<=14, -14<=k<=13, -49<=l<=49
Reflections collected / unique	38631 / 5714 [R(int) = 0.0428]
Completeness to theta = 29.11	99.8 %
Max. and min. transmission	0.9611 and 0.9378
Refinement method	Full-matrix least-squares on F^2
Data / restraints / parameters	5714 / 0 / 271
Goodness-of-fit on F^2	1.000
Final R indices [I>2sigma(I)]	R1 = 0.0535, wR2 = 0.1317
R indices (all data)	R1 = 0.0552, wR2 = 0.1331
Absolute structure parameter	0.04(9)
Largest diff. peak and hole	0.150 and -0.291 e.A^-3

Table S2. Atomic coordinates (x 10^4) and equivalent isotropic displacement parameters (A^2 x 10^3) for CCDC 1415446. U(eq) is defined as one third of the trace of the orthogonalized Uij tensor.

	x	y	z	U(eq)
S(1)	460(1)	5198(1)	1024(1)	43(1)
O(1)	954(2)	6593(2)	140(1)	52(1)
O(2)	-6141(2)	4600(2)	-664(1)	67(1)
O(3)	-5611(2)	2932(2)	-330(1)	56(1)
O(4)	-715(2)	4924(2)	1186(1)	54(1)
O(5)	1569(2)	4690(2)	1177(1)	61(1)

N(1)	-815(2)	6418(2)	-189(1)	45(1)
N(2)	420(2)	4710(2)	603(1)	40(1)
C(1)	-4172(2)	4601(2)	-399(1)	41(1)
C(2)	-3927(2)	5737(2)	-557(1)	43(1)
C(3)	-2817(2)	6328(2)	-489(1)	44(1)
C(4)	-1945(2)	5793(2)	-255(1)	38(1)
C(5)	-2170(2)	4636(2)	-93(1)	39(1)
C(6)	-3287(2)	4054(2)	-169(1)	40(1)
C(7)	-1198(2)	4053(2)	156(1)	41(1)
C(8)	-563(2)	5139(2)	358(1)	36(1)
C(9)	-53(2)	6108(2)	96(1)	41(1)
C(10)	610(2)	6822(2)	1015(1)	39(1)
C(11)	-412(2)	7572(2)	1077(1)	41(1)
C(12)	-284(2)	8854(2)	1063(1)	46(1)
C(13)	855(3)	9394(2)	984(1)	48(1)
C(14)	1865(2)	8621(3)	922(1)	48(1)
C(15)	1757(2)	7351(2)	934(1)	46(1)
C(16)	1001(3)	10781(3)	968(1)	68(1)
C(17)	-452(3)	7451(3)	-428(1)	65(1)
C(18)	-1770(3)	3176(2)	439(1)	56(1)
C(19)	-247(3)	3348(3)	-78(1)	60(1)
C(20)	-5359(2)	3942(3)	-458(1)	47(1)
C(21)	-7325(3)	4017(4)	-731(1)	96(1)

Table S3. Bond lengths [A] and angles [deg] for CCDC 1415446.

S(1)-O(5)	1.4307(18)
S(1)-O(4)	1.4312(19)
S(1)-N(2)	1.6257(19)
S(1)-C(10)	1.762(2)
O(1)-C(9)	1.219(3)
O(2)-C(20)	1.338(3)
O(2)-C(21)	1.447(4)
O(3)-C(20)	1.218(3)
N(1)-C(9)	1.370(3)
N(1)-C(4)	1.416(3)
N(1)-C(17)	1.469(3)
N(2)-C(8)	1.463(3)
N(2)-H(2N)	0.75(3)
C(1)-C(2)	1.382(4)
C(1)-C(6)	1.402(3)
C(1)-C(20)	1.482(3)
C(2)-C(3)	1.382(3)
C(2)-H(2)	0.9500
C(3)-C(4)	1.396(3)
C(3)-H(3)	0.9500
C(4)-C(5)	1.405(3)
C(5)-C(6)	1.390(3)
C(5)-C(7)	1.524(3)
C(6)-H(6)	0.9500
C(7)-C(18)	1.534(4)
C(7)-C(19)	1.537(3)
C(7)-C(8)	1.548(3)
C(8)-C(9)	1.523(3)

C(8)-H(8)	1.0000
C(10)-C(11)	1.389(3)
C(10)-C(15)	1.396(3)
C(11)-C(12)	1.392(3)
C(11)-H(11)	0.9500
C(12)-C(13)	1.392(4)
C(12)-H(12)	0.9500
C(13)-C(14)	1.393(4)
C(13)-C(16)	1.508(4)
C(14)-C(15)	1.378(4)
C(14)-H(14)	0.9500
C(15)-H(15)	0.9500
C(16)-H(16A)	0.9800
C(16)-H(16B)	0.9800
C(16)-H(16C)	0.9800
C(17)-H(17A)	0.9800
C(17)-H(17B)	0.9800
C(17)-H(17C)	0.9800
C(18)-H(18A)	0.9800
C(18)-H(18B)	0.9800
C(18)-H(18C)	0.9800
C(19)-H(19A)	0.9800
C(19)-H(19B)	0.9800
C(19)-H(19C)	0.9800
C(21)-H(21A)	0.9800
C(21)-H(21B)	0.9800
C(21)-H(21C)	0.9800
O(5)-S(1)-O(4)	120.19(12)
O(5)-S(1)-N(2)	105.44(11)

O(4)-S(1)-N(2)	107.36(11)
O(5)-S(1)-C(10)	108.24(12)
O(4)-S(1)-C(10)	107.22(11)
N(2)-S(1)-C(10)	107.87(10)
C(20)-O(2)-C(21)	115.0(3)
C(9)-N(1)-C(4)	122.07(19)
C(9)-N(1)-C(17)	118.4(2)
C(4)-N(1)-C(17)	119.47(19)
C(8)-N(2)-S(1)	119.64(15)
C(8)-N(2)-H(2N)	115(2)
S(1)-N(2)-H(2N)	108(2)
C(2)-C(1)-C(6)	119.6(2)
C(2)-C(1)-C(20)	122.1(2)
C(6)-C(1)-C(20)	118.3(2)
C(3)-C(2)-C(1)	120.1(2)
C(3)-C(2)-H(2)	119.9
C(1)-C(2)-H(2)	119.9
C(2)-C(3)-C(4)	120.3(2)
C(2)-C(3)-H(3)	119.9
C(4)-C(3)-H(3)	119.9
C(3)-C(4)-C(5)	120.7(2)
C(3)-C(4)-N(1)	119.2(2)
C(5)-C(4)-N(1)	120.13(19)
C(6)-C(5)-C(4)	117.9(2)
C(6)-C(5)-C(7)	122.1(2)
C(4)-C(5)-C(7)	120.0(2)
C(5)-C(6)-C(1)	121.5(2)
C(5)-C(6)-H(6)	119.3
C(1)-C(6)-H(6)	119.3
C(5)-C(7)-C(18)	112.3(2)

C(5)-C(7)-C(19)	109.55(19)
C(18)-C(7)-C(19)	109.7(2)
C(5)-C(7)-C(8)	106.11(18)
C(18)-C(7)-C(8)	108.92(18)
C(19)-C(7)-C(8)	110.1(2)
N(2)-C(8)-C(9)	109.89(18)
N(2)-C(8)-C(7)	112.01(17)
C(9)-C(8)-C(7)	112.35(17)
N(2)-C(8)-H(8)	107.4
C(9)-C(8)-H(8)	107.4
C(7)-C(8)-H(8)	107.4
O(1)-C(9)-N(1)	122.2(2)
O(1)-C(9)-C(8)	122.3(2)
N(1)-C(9)-C(8)	115.47(19)
C(11)-C(10)-C(15)	120.1(2)
C(11)-C(10)-S(1)	120.31(18)
C(15)-C(10)-S(1)	119.57(18)
C(10)-C(11)-C(12)	119.7(2)
C(10)-C(11)-H(11)	120.1
C(12)-C(11)-H(11)	120.1
C(13)-C(12)-C(11)	120.8(2)
C(13)-C(12)-H(12)	119.6
C(11)-C(12)-H(12)	119.6
C(12)-C(13)-C(14)	118.4(2)
C(12)-C(13)-C(16)	121.2(3)
C(14)-C(13)-C(16)	120.4(3)
C(15)-C(14)-C(13)	121.7(2)
C(15)-C(14)-H(14)	119.2
C(13)-C(14)-H(14)	119.2
C(14)-C(15)-C(10)	119.3(2)

C(14)-C(15)-H(15)	120.3
C(10)-C(15)-H(15)	120.3
C(13)-C(16)-H(16A)	109.5
C(13)-C(16)-H(16B)	109.5
H(16A)-C(16)-H(16B)	109.5
C(13)-C(16)-H(16C)	109.5
H(16A)-C(16)-H(16C)	109.5
H(16B)-C(16)-H(16C)	109.5
N(1)-C(17)-H(17A)	109.5
N(1)-C(17)-H(17B)	109.5
H(17A)-C(17)-H(17B)	109.5
N(1)-C(17)-H(17C)	109.5
H(17A)-C(17)-H(17C)	109.5
H(17B)-C(17)-H(17C)	109.5
C(7)-C(18)-H(18A)	109.5
C(7)-C(18)-H(18B)	109.5
H(18A)-C(18)-H(18B)	109.5
C(7)-C(18)-H(18C)	109.5
H(18A)-C(18)-H(18C)	109.5
H(18B)-C(18)-H(18C)	109.5
C(7)-C(19)-H(19A)	109.5
C(7)-C(19)-H(19B)	109.5
H(19A)-C(19)-H(19B)	109.5
C(7)-C(19)-H(19C)	109.5
H(19A)-C(19)-H(19C)	109.5
H(19B)-C(19)-H(19C)	109.5
O(3)-C(20)-O(2)	123.5(2)
O(3)-C(20)-C(1)	124.6(2)
O(2)-C(20)-C(1)	111.9(2)
O(2)-C(21)-H(21A)	109.5

O(2)-C(21)-H(21B)	109.5
H(21A)-C(21)-H(21B)	109.5
O(2)-C(21)-H(21C)	109.5
H(21A)-C(21)-H(21C)	109.5
H(21B)-C(21)-H(21C)	109.5

Symmetry transformations used to generate equivalent atoms:

Table S4. Anisotropic displacement parameters (A^2 x 10^3) for CCDC 1415446

The anisotropic displacement factor exponent takes the form:

-2 pi^2 [h^2 a*^2 U11 + ... + 2 h k a* b* U12]

	U11	U22	U33	U23	U13	U12
S(1)	49(1)	48(1)	33(1)	3(1)	-7(1)	1(1)
O(1)	49(1)	63(1)	45(1)	8(1)	-6(1)	-15(1)
O(2)	35(1)	97(2)	69(1)	15(1)	-14(1)	-1(1)
O(3)	40(1)	57(1)	70(1)	-10(1)	-2(1)	2(1)
O(4)	63(1)	56(1)	42(1)	6(1)	9(1)	-7(1)
O(5)	69(1)	65(1)	49(1)	1(1)	-24(1)	16(1)
N(1)	47(1)	51(1)	35(1)	8(1)	-6(1)	-7(1)
N(2)	37(1)	46(1)	36(1)	-2(1)	-7(1)	1(1)
C(1)	34(1)	49(1)	39(1)	-13(1)	-2(1)	10(1)
C(2)	42(1)	54(1)	34(1)	-8(1)	-6(1)	13(1)
C(3)	52(1)	48(1)	33(1)	-1(1)	-5(1)	5(1)
C(4)	40(1)	45(1)	29(1)	-4(1)	-2(1)	2(1)
C(5)	39(1)	41(1)	36(1)	-7(1)	-3(1)	5(1)
C(6)	40(1)	40(1)	40(1)	-9(1)	-1(1)	5(1)
C(7)	41(1)	38(1)	45(1)	-6(1)	-9(1)	4(1)
C(8)	37(1)	41(1)	31(1)	-1(1)	-3(1)	1(1)

C(9)	43(1)	47(1)	33(1)	-2(1)	-1(1)	-4(1)
C(10)	40(1)	48(1)	29(1)	-1(1)	-6(1)	1(1)
C(11)	37(1)	53(1)	34(1)	-3(1)	-4(1)	-3(1)
C(12)	47(1)	52(1)	40(1)	-4(1)	-6(1)	6(1)
C(13)	59(1)	54(1)	31(1)	3(1)	-8(1)	-9(1)
C(14)	46(1)	61(2)	38(1)	-1(1)	-2(1)	-14(1)
C(15)	37(1)	61(2)	39(1)	-5(1)	-1(1)	-4(1)
C(16)	90(2)	51(2)	63(2)	5(1)	-7(2)	-11(2)
C(17)	62(2)	81(2)	50(1)	26(1)	-12(1)	-18(2)
C(18)	61(2)	40(1)	67(2)	10(1)	-24(1)	-7(1)
C(19)	58(2)	60(2)	63(2)	-26(1)	-19(1)	23(1)
C(20)	34(1)	61(2)	46(1)	-8(1)	-1(1)	8(1)
C(21)	36(2)	141(4)	111(3)	37(3)	-22(2)	-18(2)

Table S5. Hydrogen coordinates (x 10^4) and isotropic displacement parameters (A^2 x 10^3) for CCDC 1415446.

	x	y	z	U(eq)
H(2)	-4524	6113	-712	52
H(3)	-2646	7102	-602	53
H(6)	-3455	3268	-64	48
H(8)	-1205	5551	513	44
H(11)	-1194	7213	1130	49
H(12)	-983	9366	1108	55
H(14)	2648	8979	869	58
H(15)	2455	6841	887	55
H(16A)	216	11177	1033	82
H(16B)	1237	11026	719	82
H(16C)	1647	11040	1140	82
H(17A)	403	7691	-374	77
H(17B)	-1002	8157	-384	77
H(17C)	-516	7194	-684	77
H(18A)	-2379	3628	587	67
H(18B)	-1118	2851	599	67
H(18C)	-2180	2488	313	67
H(19A)	-644	2626	-189	72
H(19B)	439	3076	78	72
H(19C)	69	3894	-270	72
H(21A)	-7198	3239	-864	115
H(21B)	-7844	4570	-878	115
H(21C)	-7734	3847	-497	115
H(2N)	1070(30)	4740(30)	526(8)	63(10)

Table S6. Torsion angles [deg] for CCDC 1415446.

O(5)-S(1)-N(2)-C(8)	-175.62(18)
O(4)-S(1)-N(2)-C(8)	55.1(2)
C(10)-S(1)-N(2)-C(8)	-60.1(2)
C(6)-C(1)-C(2)-C(3)	0.0(3)
C(20)-C(1)-C(2)-C(3)	178.8(2)
C(1)-C(2)-C(3)-C(4)	-1.2(3)
C(2)-C(3)-C(4)-C(5)	1.5(3)
C(2)-C(3)-C(4)-N(1)	-179.8(2)
C(9)-N(1)-C(4)-C(3)	164.5(2)
C(17)-N(1)-C(4)-C(3)	-14.2(3)
C(9)-N(1)-C(4)-C(5)	-16.9(3)
C(17)-N(1)-C(4)-C(5)	164.5(2)
C(3)-C(4)-C(5)-C(6)	-0.7(3)
N(1)-C(4)-C(5)-C(6)	-179.3(2)
C(3)-C(4)-C(5)-C(7)	178.8(2)
N(1)-C(4)-C(5)-C(7)	0.2(3)
C(4)-C(5)-C(6)-C(1)	-0.5(3)
C(7)-C(5)-C(6)-C(1)	-179.99(19)
C(2)-C(1)-C(6)-C(5)	0.8(3)
C(20)-C(1)-C(6)-C(5)	-178.0(2)
C(6)-C(5)-C(7)-C(18)	-27.4(3)
C(4)-C(5)-C(7)-C(18)	153.1(2)
C(6)-C(5)-C(7)-C(19)	94.8(3)
C(4)-C(5)-C(7)-C(19)	-84.6(3)
C(6)-C(5)-C(7)-C(8)	-146.3(2)
C(4)-C(5)-C(7)-C(8)	34.2(3)
S(1)-N(2)-C(8)-C(9)	103.76(19)
S(1)-N(2)-C(8)-C(7)	-130.62(17)

C(5)-C(7)-C(8)-N(2)	-178.75(18)
C(18)-C(7)-C(8)-N(2)	60.1(2)
C(19)-C(7)-C(8)-N(2)	-60.3(2)
C(5)-C(7)-C(8)-C(9)	-54.5(2)
C(18)-C(7)-C(8)-C(9)	-175.64(19)
C(19)-C(7)-C(8)-C(9)	64.0(2)
C(4)-N(1)-C(9)-O(1)	175.4(2)
C(17)-N(1)-C(9)-O(1)	-5.9(4)
C(4)-N(1)-C(9)-C(8)	-5.9(3)
C(17)-N(1)-C(9)-C(8)	172.8(2)
N(2)-C(8)-C(9)-O(1)	-12.7(3)
C(7)-C(8)-C(9)-O(1)	-138.2(2)
N(2)-C(8)-C(9)-N(1)	168.61(19)
C(7)-C(8)-C(9)-N(1)	43.2(3)
O(5)-S(1)-C(10)-C(11)	-144.50(18)
O(4)-S(1)-C(10)-C(11)	-13.5(2)
N(2)-S(1)-C(10)-C(11)	101.88(18)
O(5)-S(1)-C(10)-C(15)	37.3(2)
O(4)-S(1)-C(10)-C(15)	168.31(17)
N(2)-S(1)-C(10)-C(15)	-76.34(19)
C(15)-C(10)-C(11)-C(12)	-0.8(3)
S(1)-C(10)-C(11)-C(12)	-178.98(16)
C(10)-C(11)-C(12)-C(13)	0.5(3)
C(11)-C(12)-C(13)-C(14)	-0.3(3)
C(11)-C(12)-C(13)-C(16)	179.8(2)
C(12)-C(13)-C(14)-C(15)	0.5(3)
C(16)-C(13)-C(14)-C(15)	-179.7(2)
C(13)-C(14)-C(15)-C(10)	-0.7(3)
C(11)-C(10)-C(15)-C(14)	0.9(3)
S(1)-C(10)-C(15)-C(14)	179.11(17)

C(21)-O(2)-C(20)-O(3)	-0.6(4)
C(21)-O(2)-C(20)-C(1)	-179.6(3)
C(2)-C(1)-C(20)-O(3)	178.7(2)
C(6)-C(1)-C(20)-O(3)	-2.5(3)
C(2)-C(1)-C(20)-O(2)	-2.4(3)
C(6)-C(1)-C(20)-O(2)	176.4(2)

Symmetry transformations used to generate equivalent atoms:

Table S7. Hydrogen bonds for CCDC 1415446 [A and deg.].

D-H...A	d(D-H)	d(H...A)	d(D...A)	<(DHA)

X-ray crystallographic data for 4b

Metrical parameters for the crystal structures of compound **4b** are available free from the Cambridge Crystallographic Data Centre under reference number CCDC 1415447.

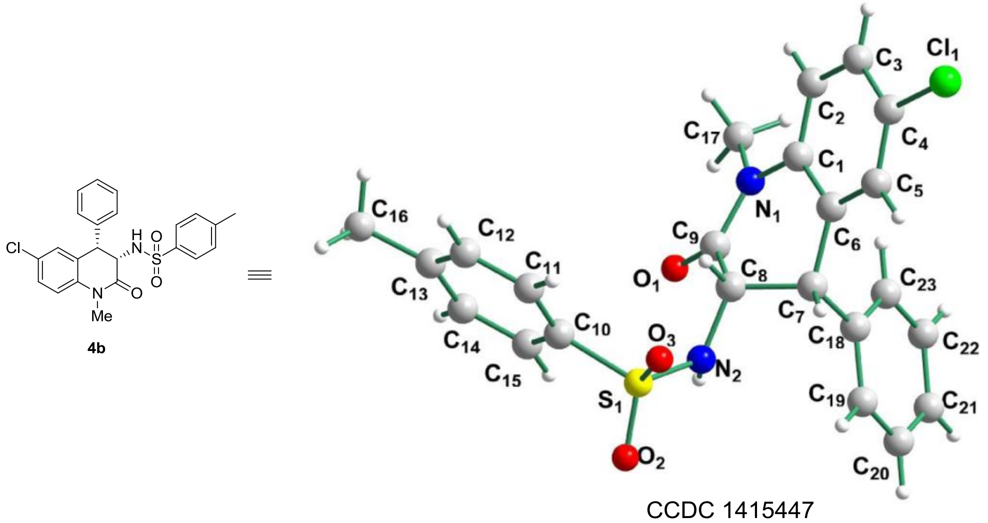

CCDC 1415447

Table S8. Crystal data and structure refinement for CCDC 1415447.

Identification code	CCDC 1415447

Empirical formula	C23 H21 Cl N2 O3 S
Formula weight	440.93
Temperature	153(2) K
Wavelength	0.71073 A
Crystal system, space group	Trigonal, P3(1)
Unit cell dimensions	a = 13.1110(4) A alpha = 90 deg.
	b = 13.1110(4) A beta = 90 deg.
	c = 10.7857(5) A gamma = 120 deg.
Volume	1605.65(10) A^3
Z, Calculated density	3, 1.368 Mg/m^3
Absorption coefficient	0.303 mm^-1
F(000)	690
Crystal size	0.45 x 0.16 x 0.16 mm
Theta range for data collection	2.60 to 31.47 deg.
Limiting indices	-19<=h<=18, -18<=k<=17, -15<=l<=15
Reflections collected / unique	16159 / 6924 [R(int) = 0.0338]
Completeness to theta = 31.47	99.9 %
Absorption correction	Semi-empirical from equivalents
Max. and min. transmission	0.9530 and 0.8755
Refinement method	Full-matrix least-squares on F^2
Data / restraints / parameters	6924 / 1 / 277
Goodness-of-fit on F^2	1.002
Final R indices [I>2sigma(I)]	R1 = 0.0444, wR2 = 0.0853
R indices (all data)	R1 = 0.0519, wR2 = 0.0894
Absolute structure parameter	0.03(4)
Largest diff. peak and hole	0.217 and -0.268 e.A^-3

Table S9. Atomic coordinates (x 10^4) and equivalent isotropic displacement parameters (A^2 x 10^3) for CCDC 1415447.
U(eq) is defined as one third of the trace of the orthogonalized Uij tensor.

	x	y	z	U(eq)
Cl(1)	1942(1)	9403(1)	-5542(1)	38(1)
S(1)	4432(1)	8081(1)	894(1)	26(1)
O(1)	2435(2)	9303(2)	1786(1)	48(1)
O(2)	4373(1)	7182(1)	1692(1)	35(1)
O(3)	4899(1)	8212(1)	-339(1)	36(1)
N(1)	1980(2)	9805(2)	-63(1)	31(1)
N(2)	3100(1)	7829(1)	774(2)	28(1)
C(1)	1943(2)	9710(2)	-1373(2)	25(1)
C(2)	1938(2)	10582(2)	-2119(2)	29(1)
C(3)	1930(2)	10491(2)	-3401(2)	32(1)
C(4)	1926(2)	9526(2)	-3932(2)	27(1)
C(5)	1911(2)	8642(2)	-3201(2)	25(1)
C(6)	1906(2)	8723(2)	-1918(2)	22(1)
C(7)	1973(2)	7820(2)	-1093(2)	23(1)
C(8)	2853(2)	8549(2)	-64(2)	25(1)
C(9)	2406(2)	9245(2)	662(2)	31(1)
C(10)	5285(2)	9460(2)	1635(2)	26(1)
C(11)	6230(2)	10359(2)	1016(2)	35(1)
C(12)	6930(2)	11417(2)	1621(2)	41(1)
C(13)	6696(2)	11585(2)	2846(2)	40(1)
C(14)	5731(2)	10675(2)	3430(2)	41(1)
C(15)	5010(2)	9608(2)	2839(2)	36(1)
C(16)	7490(2)	12728(2)	3506(3)	60(1)

C(17)	1543(3)	10513(3)	541(2)	58(1)
C(18)	797(2)	6872(2)	-549(2)	25(1)
C(19)	683(2)	5795(2)	-199(2)	31(1)
C(20)	-352(2)	4924(2)	334(2)	38(1)
C(21)	-1287(2)	5109(2)	517(2)	45(1)
C(22)	-1195(2)	6162(2)	168(2)	42(1)
C(23)	-156(2)	7049(2)	-355(2)	32(1)

Table S10. Bond lengths [A] and angles [deg] for BO4108.

Cl(1)-C(4)	1.7448(19)
S(1)-O(2)	1.4296(14)
S(1)-O(3)	1.4372(14)
S(1)-N(2)	1.6118(16)
S(1)-C(10)	1.7716(19)
O(1)-C(9)	1.214(2)
N(1)-C(9)	1.369(2)
N(1)-C(1)	1.417(2)
N(1)-C(17)	1.464(3)
N(2)-C(8)	1.456(2)
N(2)-H(2N)	0.89(2)
C(1)-C(6)	1.400(2)
C(1)-C(2)	1.401(2)
C(2)-C(3)	1.388(3)
C(2)-H(2)	0.9500
C(3)-C(4)	1.386(3)
C(3)-H(3)	0.9500
C(4)-C(5)	1.393(3)
C(5)-C(6)	1.389(2)
C(5)-H(5)	0.9500
C(6)-C(7)	1.518(2)
C(7)-C(18)	1.532(2)
C(7)-C(8)	1.541(2)
C(7)-H(7)	1.0000
C(8)-C(9)	1.525(3)
C(8)-H(8)	1.0000
C(10)-C(11)	1.382(3)
C(10)-C(15)	1.387(3)

C(11)-C(12)	1.386(3)
C(11)-H(11)	0.9500
C(12)-C(13)	1.399(3)
C(12)-H(12)	0.9500
C(13)-C(14)	1.383(3)
C(13)-C(16)	1.508(3)
C(14)-C(15)	1.391(3)
C(14)-H(14)	0.9500
C(15)-H(15)	0.9500
C(16)-H(16A)	0.9800
C(16)-H(16B)	0.9800
C(16)-H(16C)	0.9800
C(17)-H(17A)	0.9800
C(17)-H(17B)	0.9800
C(17)-H(17C)	0.9800
C(18)-C(23)	1.395(3)
C(18)-C(19)	1.396(2)
C(19)-C(20)	1.387(3)
C(19)-H(19)	0.9500
C(20)-C(21)	1.378(3)
C(20)-H(20)	0.9500
C(21)-C(22)	1.377(3)
C(21)-H(21)	0.9500
C(22)-C(23)	1.394(3)
C(22)-H(22)	0.9500
C(23)-H(23)	0.9500
O(2)-S(1)-O(3)	120.00(9)
O(2)-S(1)-N(2)	105.79(8)
O(3)-S(1)-N(2)	107.67(9)

O(2)-S(1)-C(10)	108.45(8)
O(3)-S(1)-C(10)	106.35(9)
N(2)-S(1)-C(10)	108.12(8)
C(9)-N(1)-C(1)	121.80(15)
C(9)-N(1)-C(17)	118.65(17)
C(1)-N(1)-C(17)	119.55(17)
C(8)-N(2)-S(1)	119.11(13)
C(8)-N(2)-H(2N)	116.5(14)
S(1)-N(2)-H(2N)	115.9(14)
C(6)-C(1)-C(2)	120.12(16)
C(6)-C(1)-N(1)	118.97(16)
C(2)-C(1)-N(1)	120.91(16)
C(3)-C(2)-C(1)	120.27(17)
C(3)-C(2)-H(2)	119.9
C(1)-C(2)-H(2)	119.9
C(4)-C(3)-C(2)	119.15(17)
C(4)-C(3)-H(3)	120.4
C(2)-C(3)-H(3)	120.4
C(3)-C(4)-C(5)	121.15(18)
C(3)-C(4)-Cl(1)	120.01(14)
C(5)-C(4)-Cl(1)	118.83(15)
C(6)-C(5)-C(4)	119.94(17)
C(6)-C(5)-H(5)	120.0
C(4)-C(5)-H(5)	120.0
C(5)-C(6)-C(1)	119.32(16)
C(5)-C(6)-C(7)	121.37(16)
C(1)-C(6)-C(7)	119.08(15)
C(6)-C(7)-C(18)	115.47(14)
C(6)-C(7)-C(8)	104.82(14)
C(18)-C(7)-C(8)	111.39(14)

C(6)-C(7)-H(7)	108.3
C(18)-C(7)-H(7)	108.3
C(8)-C(7)-H(7)	108.3
N(2)-C(8)-C(9)	110.29(15)
N(2)-C(8)-C(7)	112.68(14)
C(9)-C(8)-C(7)	110.39(14)
N(2)-C(8)-H(8)	107.8
C(9)-C(8)-H(8)	107.8
C(7)-C(8)-H(8)	107.8
O(1)-C(9)-N(1)	123.05(18)
O(1)-C(9)-C(8)	122.72(18)
N(1)-C(9)-C(8)	114.23(16)
C(11)-C(10)-C(15)	121.20(18)
C(11)-C(10)-S(1)	119.45(15)
C(15)-C(10)-S(1)	119.33(15)
C(10)-C(11)-C(12)	119.3(2)
C(10)-C(11)-H(11)	120.4
C(12)-C(11)-H(11)	120.4
C(11)-C(12)-C(13)	121.0(2)
C(11)-C(12)-H(12)	119.5
C(13)-C(12)-H(12)	119.5
C(14)-C(13)-C(12)	118.23(19)
C(14)-C(13)-C(16)	121.4(2)
C(12)-C(13)-C(16)	120.4(2)
C(13)-C(14)-C(15)	121.8(2)
C(13)-C(14)-H(14)	119.1
C(15)-C(14)-H(14)	119.1
C(10)-C(15)-C(14)	118.5(2)
C(10)-C(15)-H(15)	120.8
C(14)-C(15)-H(15)	120.8

C(13)-C(16)-H(16A)	109.5
C(13)-C(16)-H(16B)	109.5
H(16A)-C(16)-H(16B)	109.5
C(13)-C(16)-H(16C)	109.5
H(16A)-C(16)-H(16C)	109.5
H(16B)-C(16)-H(16C)	109.5
N(1)-C(17)-H(17A)	109.5
N(1)-C(17)-H(17B)	109.5
H(17A)-C(17)-H(17B)	109.5
N(1)-C(17)-H(17C)	109.5
H(17A)-C(17)-H(17C)	109.5
H(17B)-C(17)-H(17C)	109.5
C(23)-C(18)-C(19)	118.35(17)
C(23)-C(18)-C(7)	122.95(17)
C(19)-C(18)-C(7)	118.67(16)
C(20)-C(19)-C(18)	120.75(18)
C(20)-C(19)-H(19)	119.6
C(18)-C(19)-H(19)	119.6
C(21)-C(20)-C(19)	120.2(2)
C(21)-C(20)-H(20)	119.9
C(19)-C(20)-H(20)	119.9
C(22)-C(21)-C(20)	119.9(2)
C(22)-C(21)-H(21)	120.0
C(20)-C(21)-H(21)	120.0
C(21)-C(22)-C(23)	120.3(2)
C(21)-C(22)-H(22)	119.8
C(23)-C(22)-H(22)	119.8
C(22)-C(23)-C(18)	120.39(19)
C(22)-C(23)-H(23)	119.8
C(18)-C(23)-H(23)	119.8

Symmetry transformations used to generate equivalent atoms:

Table S11. Anisotropic displacement parameters (A^2 x 10^3) for CCDC 1415447. The anisotropic displacement factor exponent takes the form: -2 pi^2 [h^2 a*^2 U11 + ... + 2 h k a* b* U12]

	U11	U22	U33	U23	U13	U12
Cl(1)	45(1)	43(1)	24(1)	6(1)	3(1)	20(1)
S(1)	27(1)	26(1)	29(1)	-2(1)	-4(1)	15(1)
O(1)	79(1)	59(1)	25(1)	-7(1)	-8(1)	49(1)
O(2)	35(1)	28(1)	45(1)	3(1)	-9(1)	19(1)
O(3)	36(1)	41(1)	33(1)	-6(1)	2(1)	21(1)
N(1)	43(1)	34(1)	26(1)	-6(1)	-4(1)	27(1)
N(2)	28(1)	31(1)	26(1)	4(1)	-2(1)	16(1)
C(1)	26(1)	24(1)	25(1)	-2(1)	-3(1)	14(1)
C(2)	33(1)	25(1)	33(1)	-3(1)	-4(1)	16(1)
C(3)	32(1)	27(1)	35(1)	6(1)	-1(1)	14(1)
C(4)	23(1)	31(1)	24(1)	3(1)	2(1)	11(1)
C(5)	23(1)	26(1)	26(1)	-1(1)	0(1)	12(1)
C(6)	21(1)	23(1)	23(1)	0(1)	-1(1)	11(1)
C(7)	25(1)	23(1)	21(1)	-2(1)	-1(1)	14(1)
C(8)	24(1)	25(1)	27(1)	0(1)	-4(1)	13(1)
C(9)	39(1)	31(1)	26(1)	-4(1)	-4(1)	20(1)
C(10)	26(1)	26(1)	30(1)	-1(1)	-5(1)	15(1)
C(11)	30(1)	33(1)	41(1)	3(1)	2(1)	16(1)
C(12)	29(1)	29(1)	60(1)	1(1)	-1(1)	12(1)
C(13)	38(1)	30(1)	55(1)	-7(1)	-16(1)	18(1)
C(14)	56(1)	33(1)	34(1)	-6(1)	-9(1)	23(1)

C(15)	43(1)	30(1)	32(1)	1(1)	-1(1)	16(1)
C(16)	53(2)	36(1)	85(2)	-19(1)	-21(1)	17(1)
C(17)	104(2)	70(2)	38(1)	-11(1)	-2(1)	70(2)
C(18)	26(1)	27(1)	24(1)	-1(1)	-1(1)	14(1)
C(19)	28(1)	30(1)	38(1)	3(1)	2(1)	16(1)
C(20)	32(1)	32(1)	50(1)	9(1)	3(1)	15(1)
C(21)	29(1)	41(1)	57(1)	13(1)	10(1)	12(1)
C(22)	27(1)	45(1)	56(1)	6(1)	8(1)	20(1)
C(23)	30(1)	35(1)	38(1)	2(1)	0(1)	20(1)

Table S12. Hydrogen coordinates (x 10^4) and isotropic displacement parameters (A^2 x 10^3) for CCDC 1415447.

	x	y	z	U(eq)
H(2)	1940	11239	-1745	35
H(3)	1927	11081	-3908	38
H(5)	1904	7984	-3581	30
H(7)	2318	7420	-1587	27
H(8)	3610	9126	-471	30
H(11)	6398	10252	184	41
H(12)	7579	12038	1197	49
H(14)	5555	10782	4258	49
H(15)	4346	8995	3251	43
H(16A)	7601	13390	2988	72
H(16B)	8256	12789	3662	72
H(16C)	7131	12746	4296	72
H(17A)	1456	10345	1433	70
H(17B)	777	10316	188	70
H(17C)	2104	11351	408	70
H(19)	1320	5656	-327	37
H(20)	-416	4197	574	46
H(21)	-1993	4512	883	54
H(22)	-1844	6285	284	50
H(23)	-95	7777	-581	39
H(2N)	2667(19)	7580(20)	1460(20)	33(6)

Table S13. Torsion angles [deg] for CCDC 1415447.

O(2)-S(1)-N(2)-C(8)	-175.31(14)
O(3)-S(1)-N(2)-C(8)	-45.85(16)
C(10)-S(1)-N(2)-C(8)	68.68(15)
C(9)-N(1)-C(1)-C(6)	24.5(3)
C(17)-N(1)-C(1)-C(6)	-155.0(2)
C(9)-N(1)-C(1)-C(2)	-155.83(19)
C(17)-N(1)-C(1)-C(2)	24.7(3)
C(6)-C(1)-C(2)-C(3)	-1.8(3)
N(1)-C(1)-C(2)-C(3)	178.48(18)
C(1)-C(2)-C(3)-C(4)	0.1(3)
C(2)-C(3)-C(4)-C(5)	1.0(3)
C(2)-C(3)-C(4)-Cl(1)	-178.87(15)
C(3)-C(4)-C(5)-C(6)	-0.4(3)
Cl(1)-C(4)-C(5)-C(6)	179.55(13)
C(4)-C(5)-C(6)-C(1)	-1.4(3)
C(4)-C(5)-C(6)-C(7)	-175.85(16)
C(2)-C(1)-C(6)-C(5)	2.5(3)
N(1)-C(1)-C(6)-C(5)	-177.81(16)
C(2)-C(1)-C(6)-C(7)	177.07(16)
N(1)-C(1)-C(6)-C(7)	-3.2(2)
C(5)-C(6)-C(7)-C(18)	-100.24(19)
C(1)-C(6)-C(7)-C(18)	85.3(2)
C(5)-C(6)-C(7)-C(8)	136.82(16)
C(1)-C(6)-C(7)-C(8)	-37.6(2)
S(1)-N(2)-C(8)-C(9)	-116.77(15)
S(1)-N(2)-C(8)-C(7)	119.38(15)
C(6)-C(7)-C(8)-N(2)	-175.59(14)
C(18)-C(7)-C(8)-N(2)	58.87(19)
C(6)-C(7)-C(8)-C(9)	60.62(18)
C(18)-C(7)-C(8)-C(9)	-64.92(19)

C(1)-N(1)-C(9)-O(1)	-179.2(2)
C(17)-N(1)-C(9)-O(1)	0.3(3)
C(1)-N(1)-C(9)-C(8)	1.3(3)
C(17)-N(1)-C(9)-C(8)	-179.2(2)
N(2)-C(8)-C(9)-O(1)	10.1(3)
C(7)-C(8)-C(9)-O(1)	135.2(2)
N(2)-C(8)-C(9)-N(1)	-170.42(16)
C(7)-C(8)-C(9)-N(1)	-45.2(2)
O(2)-S(1)-C(10)-C(11)	126.74(15)
O(3)-S(1)-C(10)-C(11)	-3.60(17)
N(2)-S(1)-C(10)-C(11)	-119.00(15)
O(2)-S(1)-C(10)-C(15)	-51.48(17)
O(3)-S(1)-C(10)-C(15)	178.18(15)
N(2)-S(1)-C(10)-C(15)	62.78(17)
C(15)-C(10)-C(11)-C(12)	1.4(3)
S(1)-C(10)-C(11)-C(12)	-176.79(15)
C(10)-C(11)-C(12)-C(13)	0.2(3)
C(11)-C(12)-C(13)-C(14)	-1.4(3)
C(11)-C(12)-C(13)-C(16)	177.9(2)
C(12)-C(13)-C(14)-C(15)	0.9(3)
C(16)-C(13)-C(14)-C(15)	-178.4(2)
C(11)-C(10)-C(15)-C(14)	-1.8(3)
S(1)-C(10)-C(15)-C(14)	176.37(16)
C(13)-C(14)-C(15)-C(10)	0.6(3)
C(6)-C(7)-C(18)-C(23)	-26.0(2)
C(8)-C(7)-C(18)-C(23)	93.36(19)
C(6)-C(7)-C(18)-C(19)	155.81(16)
C(8)-C(7)-C(18)-C(19)	-84.8(2)
C(23)-C(18)-C(19)-C(20)	-0.3(3)
C(7)-C(18)-C(19)-C(20)	177.95(18)

C(18)-C(19)-C(20)-C(21)	0.5(3)
C(19)-C(20)-C(21)-C(22)	0.1(4)
C(20)-C(21)-C(22)-C(23)	-0.9(4)
C(21)-C(22)-C(23)-C(18)	1.0(3)
C(19)-C(18)-C(23)-C(22)	-0.5(3)
C(7)-C(18)-C(23)-C(22)	-178.62(18)

Symmetry transformations used to generate equivalent atoms:

Table S14. Hydrogen bonds for CCDC 1415447 [A and deg.].

D-H...A	d(D-H)	d(H...A)	d(D...A)	<(DHA)

X-ray crystallographic data for 6m

Metrical parameters for the crystal structures of compound **6m** are available free from the Cambridge Crystallographic Data Centre under reference number CCDC 1415448.

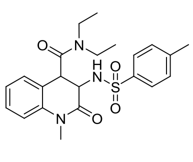

cis-6m

≡

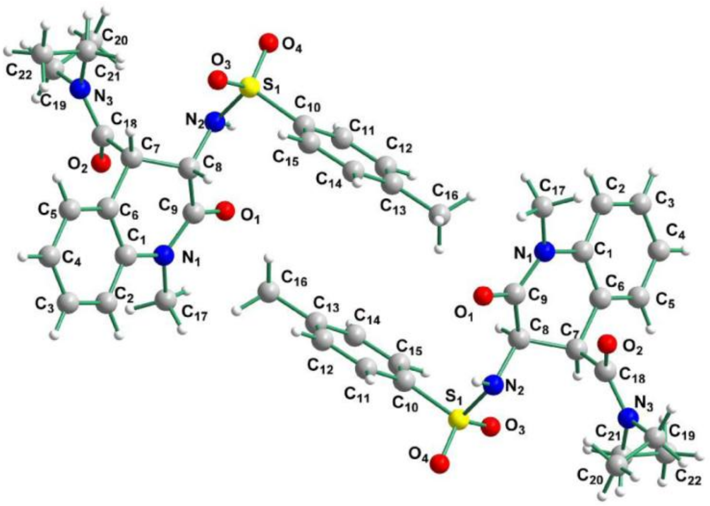

CCDC 1415448

Table S15. Crystal data and structure refinement for CCDC 1415448.

Identification code	CCDC 1415448

Empirical formula	C22 H27 N3 O4 S
Formula weight	429.53
Temperature	153(2) K
Wavelength	0.71073 A
Crystal system, space group	?, ?
Unit cell dimensions	a = 9.956(3) A alpha = 106.896(4) deg.
	b = 11.095(3) A beta = 107.810(2) deg.
	c = 11.180(3) A gamma = 96.9220(10) deg.
Volume	1094.9(6) A^3
Z, Calculated density	2, 1.303 Mg/m^3
Absorption coefficient	0.181 mm^-1
F(000)	456
Crystal size	0.36 x 0.21 x 0.12 mm
Theta range for data collection	2.60 to 31.51 deg.
Limiting indices	-14<=h<=14, -16<=k<=16, -16<=l<=16
Reflections collected / unique	18568 / 7162 [R(int) = 0.0340]
Completeness to theta = 31.51	97.9 %
Max. and min. transmission	0.9786 and 0.9377
Refinement method	Full-matrix least-squares on F^2
Data / restraints / parameters	7162 / 0 / 279
Goodness-of-fit on F^2	0.999
Final R indices [I>2sigma(I)]	R1 = 0.0580, wR2 = 0.1317
R indices (all data)	R1 = 0.0691, wR2 = 0.1399
Largest diff. peak and hole	0.343 and -0.442 e.A^-3

Table S16. Atomic coordinates (x 10^4) and equivalent isotropic displacement parameters (A^2 x 10^3) for bo4103.

U(eq) is defined as one third of the trace of the orthogonalized Uij tensor.

	x	y	z	U(eq)
S(1)	5685(1)	2973(1)	7296(1)	21(1)
O(1)	3505(1)	4200(1)	9768(1)	30(1)
O(2)	1590(1)	4992(1)	7149(1)	30(1)
O(3)	4917(1)	2091(1)	5948(1)	27(1)
O(4)	6950(1)	3934(1)	7591(1)	28(1)
N(1)	1237(1)	2963(1)	8475(1)	23(1)
N(2)	4528(1)	3738(1)	7753(1)	22(1)
N(3)	2176(2)	4709(1)	5301(1)	27(1)
C(1)	240(2)	2158(1)	7187(2)	22(1)
C(2)	-1049(2)	1387(2)	7069(2)	29(1)
C(3)	-2047(2)	631(2)	5806(2)	32(1)
C(4)	-1766(2)	632(2)	4671(2)	29(1)
C(5)	-474(2)	1368(2)	4790(2)	25(1)
C(6)	537(2)	2138(1)	6039(2)	22(1)
C(7)	1976(2)	2906(1)	6180(1)	20(1)
C(8)	3111(2)	2955(1)	7489(2)	19(1)
C(9)	2640(2)	3457(1)	8684(2)	21(1)
C(10)	6154(2)	2021(2)	8321(2)	21(1)
C(11)	6935(2)	2640(2)	9690(2)	27(1)
C(12)	7351(2)	1897(2)	10483(2)	33(1)
C(13)	6994(2)	545(2)	9935(2)	34(1)
C(14)	6206(2)	-47(2)	8573(2)	32(1)
C(15)	5775(2)	681(2)	7755(2)	26(1)
C(16)	7444(3)	-265(3)	10803(2)	53(1)
C(17)	697(2)	3398(2)	9571(2)	33(1)
C(18)	1906(2)	4298(2)	6241(2)	22(1)
C(19)	2113(2)	6048(2)	5396(2)	32(1)

C(20)	3554(2)	6976(2)	6315(2)	39(1)
C(21)	2764(2)	4023(2)	4310(2)	36(1)
C(22)	1588(3)	3248(3)	2968(2)	55(1)

Table S17. Bond lengths [A] and angles [deg] for CCDC 1415448.

S(1)-O(4)	1.4321(12)
S(1)-O(3)	1.4396(12)
S(1)-N(2)	1.6277(14)
S(1)-C(10)	1.7732(16)
O(1)-C(9)	1.2245(18)
O(2)-C(18)	1.234(2)
N(1)-C(9)	1.3590(19)
N(1)-C(1)	1.4216(19)
N(1)-C(17)	1.464(2)
N(2)-C(8)	1.4602(19)
N(2)-H(2N)	0.84(2)
N(3)-C(18)	1.345(2)
N(3)-C(19)	1.469(2)
N(3)-C(21)	1.471(2)
C(1)-C(6)	1.397(2)
C(1)-C(2)	1.401(2)
C(2)-C(3)	1.392(2)
C(2)-H(2)	0.9500
C(3)-C(4)	1.381(3)
C(3)-H(3)	0.9500
C(4)-C(5)	1.384(2)
C(4)-H(4)	0.9500
C(5)-C(6)	1.390(2)
C(5)-H(5)	0.9500
C(6)-C(7)	1.514(2)
C(7)-C(8)	1.533(2)
C(7)-C(18)	1.536(2)
C(7)-H(7)	1.0000

C(8)-C(9)	1.527(2)
C(8)-H(8)	1.0000
C(10)-C(15)	1.387(2)
C(10)-C(11)	1.396(2)
C(11)-C(12)	1.386(2)
C(11)-H(11)	0.9500
C(12)-C(13)	1.398(3)
C(12)-H(12)	0.9500
C(13)-C(14)	1.389(3)
C(13)-C(16)	1.513(3)
C(14)-C(15)	1.395(2)
C(14)-H(14)	0.9500
C(15)-H(15)	0.9500
C(16)-H(16A)	0.9800
C(16)-H(16B)	0.9800
C(16)-H(16C)	0.9800
C(17)-H(17A)	0.9800
C(17)-H(17B)	0.9800
C(17)-H(17C)	0.9800
C(19)-C(20)	1.517(3)
C(19)-H(19A)	0.9900
C(19)-H(19B)	0.9900
C(20)-H(20A)	0.9800
C(20)-H(20B)	0.9800
C(20)-H(20C)	0.9800
C(21)-C(22)	1.514(3)
C(21)-H(21A)	0.9900
C(21)-H(21B)	0.9900
C(22)-H(22A)	0.9800
C(22)-H(22B)	0.9800

C(22)-H(22C)	0.9800
O(4)-S(1)-O(3)	120.24(8)
O(4)-S(1)-N(2)	106.94(8)
O(3)-S(1)-N(2)	106.67(7)
O(4)-S(1)-C(10)	108.45(7)
O(3)-S(1)-C(10)	106.98(8)
N(2)-S(1)-C(10)	106.88(7)
C(9)-N(1)-C(1)	121.87(13)
C(9)-N(1)-C(17)	118.78(13)
C(1)-N(1)-C(17)	118.94(13)
C(8)-N(2)-S(1)	117.02(11)
C(8)-N(2)-H(2N)	117.0(15)
S(1)-N(2)-H(2N)	109.7(15)
C(18)-N(3)-C(19)	117.59(15)
C(18)-N(3)-C(21)	125.94(14)
C(19)-N(3)-C(21)	115.67(15)
C(6)-C(1)-C(2)	119.90(14)
C(6)-C(1)-N(1)	120.31(14)
C(2)-C(1)-N(1)	119.80(15)
C(3)-C(2)-C(1)	119.83(17)
C(3)-C(2)-H(2)	120.1
C(1)-C(2)-H(2)	120.1
C(4)-C(3)-C(2)	120.22(16)
C(4)-C(3)-H(3)	119.9
C(2)-C(3)-H(3)	119.9
C(3)-C(4)-C(5)	119.89(15)
C(3)-C(4)-H(4)	120.1
C(5)-C(4)-H(4)	120.1
C(4)-C(5)-C(6)	121.05(17)

C(4)-C(5)-H(5)	119.5
C(6)-C(5)-H(5)	119.5
C(5)-C(6)-C(1)	119.08(15)
C(5)-C(6)-C(7)	121.33(15)
C(1)-C(6)-C(7)	119.53(13)
C(6)-C(7)-C(8)	107.82(12)
C(6)-C(7)-C(18)	111.53(12)
C(8)-C(7)-C(18)	108.70(12)
C(6)-C(7)-H(7)	109.6
C(8)-C(7)-H(7)	109.6
C(18)-C(7)-H(7)	109.6
N(2)-C(8)-C(9)	109.67(12)
N(2)-C(8)-C(7)	112.01(12)
C(9)-C(8)-C(7)	111.87(12)
N(2)-C(8)-H(8)	107.7
C(9)-C(8)-H(8)	107.7
C(7)-C(8)-H(8)	107.7
O(1)-C(9)-N(1)	123.37(15)
O(1)-C(9)-C(8)	121.15(14)
N(1)-C(9)-C(8)	115.37(12)
C(15)-C(10)-C(11)	121.01(14)
C(15)-C(10)-S(1)	119.97(12)
C(11)-C(10)-S(1)	119.00(12)
C(12)-C(11)-C(10)	119.05(16)
C(12)-C(11)-H(11)	120.5
C(10)-C(11)-H(11)	120.5
C(11)-C(12)-C(13)	121.08(16)
C(11)-C(12)-H(12)	119.5
C(13)-C(12)-H(12)	119.5
C(14)-C(13)-C(12)	118.73(16)

C(14)-C(13)-C(16)	120.24(19)
C(12)-C(13)-C(16)	121.02(18)
C(13)-C(14)-C(15)	121.22(17)
C(13)-C(14)-H(14)	119.4
C(15)-C(14)-H(14)	119.4
C(10)-C(15)-C(14)	118.91(16)
C(10)-C(15)-H(15)	120.5
C(14)-C(15)-H(15)	120.5
C(13)-C(16)-H(16A)	109.5
C(13)-C(16)-H(16B)	109.5
H(16A)-C(16)-H(16B)	109.5
C(13)-C(16)-H(16C)	109.5
H(16A)-C(16)-H(16C)	109.5
H(16B)-C(16)-H(16C)	109.5
N(1)-C(17)-H(17A)	109.5
N(1)-C(17)-H(17B)	109.5
H(17A)-C(17)-H(17B)	109.5
N(1)-C(17)-H(17C)	109.5
H(17A)-C(17)-H(17C)	109.5
H(17B)-C(17)-H(17C)	109.5
O(2)-C(18)-N(3)	122.51(15)
O(2)-C(18)-C(7)	118.46(14)
N(3)-C(18)-C(7)	119.03(14)
N(3)-C(19)-C(20)	110.94(14)
N(3)-C(19)-H(19A)	109.5
C(20)-C(19)-H(19A)	109.5
N(3)-C(19)-H(19B)	109.5
C(20)-C(19)-H(19B)	109.5
H(19A)-C(19)-H(19B)	108.0
C(19)-C(20)-H(20A)	109.5

C(19)-C(20)-H(20B)	109.5
H(20A)-C(20)-H(20B)	109.5
C(19)-C(20)-H(20C)	109.5
H(20A)-C(20)-H(20C)	109.5
H(20B)-C(20)-H(20C)	109.5
N(3)-C(21)-C(22)	112.37(18)
N(3)-C(21)-H(21A)	109.1
C(22)-C(21)-H(21A)	109.1
N(3)-C(21)-H(21B)	109.1
C(22)-C(21)-H(21B)	109.1
H(21A)-C(21)-H(21B)	107.9
C(21)-C(22)-H(22A)	109.5
C(21)-C(22)-H(22B)	109.5
H(22A)-C(22)-H(22B)	109.5
C(21)-C(22)-H(22C)	109.5
H(22A)-C(22)-H(22C)	109.5
H(22B)-C(22)-H(22C)	109.5

Symmetry transformations used to generate equivalent atoms:

Table S18. Anisotropic displacement parameters (A^2 x 10^3) for CCDC 1415448.

The anisotropic displacement factor exponent takes the form:

-2 pi^2 [h^2 a*^2 U11 + ... + 2 h k a* b* U12]

	U11	U22	U33	U23	U13	U12
S(1)	17(1)	23(1)	22(1)	9(1)	6(1)	4(1)
O(1)	24(1)	32(1)	23(1)	3(1)	4(1)	-3(1)

O(2)	38(1)	23(1)	31(1)	9(1)	14(1)	11(1)
O(3)	26(1)	30(1)	22(1)	8(1)	6(1)	8(1)
O(4)	22(1)	31(1)	35(1)	15(1)	12(1)	3(1)
N(1)	18(1)	23(1)	22(1)	5(1)	5(1)	1(1)
N(2)	18(1)	20(1)	24(1)	6(1)	4(1)	3(1)
N(3)	29(1)	26(1)	27(1)	14(1)	8(1)	9(1)
C(1)	17(1)	19(1)	26(1)	5(1)	4(1)	4(1)
C(2)	20(1)	26(1)	34(1)	6(1)	8(1)	2(1)
C(3)	18(1)	24(1)	42(1)	3(1)	6(1)	0(1)
C(4)	19(1)	21(1)	33(1)	0(1)	-1(1)	4(1)
C(5)	23(1)	20(1)	25(1)	3(1)	1(1)	5(1)
C(6)	18(1)	17(1)	25(1)	6(1)	2(1)	5(1)
C(7)	19(1)	19(1)	19(1)	6(1)	3(1)	3(1)
C(8)	15(1)	19(1)	24(1)	8(1)	4(1)	3(1)
C(9)	18(1)	19(1)	22(1)	7(1)	4(1)	2(1)
C(10)	17(1)	25(1)	24(1)	11(1)	6(1)	7(1)
C(11)	27(1)	27(1)	26(1)	9(1)	6(1)	8(1)
C(12)	34(1)	38(1)	24(1)	12(1)	6(1)	12(1)
C(13)	33(1)	39(1)	35(1)	22(1)	11(1)	13(1)
C(14)	30(1)	25(1)	39(1)	14(1)	7(1)	5(1)
C(15)	20(1)	26(1)	29(1)	9(1)	6(1)	3(1)
C(16)	65(2)	55(1)	50(1)	36(1)	15(1)	23(1)
C(17)	26(1)	38(1)	29(1)	3(1)	12(1)	0(1)
C(18)	18(1)	20(1)	23(1)	7(1)	3(1)	4(1)
C(19)	32(1)	29(1)	39(1)	20(1)	9(1)	10(1)
C(20)	35(1)	30(1)	53(1)	20(1)	12(1)	4(1)
C(21)	41(1)	40(1)	36(1)	20(1)	18(1)	14(1)
C(22)	73(2)	56(1)	30(1)	11(1)	15(1)	10(1)

Table S19. Hydrogen coordinates (x 10^4) and isotropic displacement parameters (A^2 x 10^3) for CCDC 1415448.

	x	y	z	U(eq)
H(2)	-1241	1380	7849	34
H(3)	-2924	112	5724	38
H(4)	-2458	129	3810	35
H(5)	-276	1345	4006	30
H(7)	2258	2467	5408	24
H(8)	3218	2051	7396	23
H(11)	7178	3558	10071	33
H(12)	7887	2312	11413	39
H(14)	5956	-965	8192	38
H(15)	5231	266	6826	31
H(16A)	7174	-1181	10238	64
H(16B)	6955	-138	11454	64
H(16C)	8496	-2	11279	64
H(17A)	492	2680	9880	40
H(17B)	-193	3690	9253	40
H(17C)	1432	4115	10312	40
H(19A)	1344	6290	5744	38
H(19B)	1863	6114	4493	38
H(20A)	3820	6886	7200	47
H(20B)	3470	7866	6403	47
H(20C)	4302	6775	5938	47
H(21A)	3312	3433	4655	43
H(21B)	3448	4660	4187	43
H(22A)	998	2529	3057	66
H(22B)	2033	2906	2309	66

H(22C)	972	3808	2670	66
H(2N)	4950(20)	4300(20)	8520(20)	34(6)

Table S20. Torsion angles [deg] for CCDC 1415448.

O(4)-S(1)-N(2)-C(8)	177.15(11)
O(3)-S(1)-N(2)-C(8)	47.30(13)
C(10)-S(1)-N(2)-C(8)	-66.87(12)
C(9)-N(1)-C(1)-C(6)	-18.0(2)
C(17)-N(1)-C(1)-C(6)	154.46(16)
C(9)-N(1)-C(1)-C(2)	162.23(15)
C(17)-N(1)-C(1)-C(2)	-25.3(2)
C(6)-C(1)-C(2)-C(3)	-1.8(2)
N(1)-C(1)-C(2)-C(3)	177.90(15)
C(1)-C(2)-C(3)-C(4)	0.5(3)
C(2)-C(3)-C(4)-C(5)	1.3(3)
C(3)-C(4)-C(5)-C(6)	-1.8(2)
C(4)-C(5)-C(6)-C(1)	0.4(2)
C(4)-C(5)-C(6)-C(7)	177.71(14)
C(2)-C(1)-C(6)-C(5)	1.4(2)
N(1)-C(1)-C(6)-C(5)	-178.35(14)
C(2)-C(1)-C(6)-C(7)	-175.97(14)
N(1)-C(1)-C(6)-C(7)	4.3(2)
C(5)-C(6)-C(7)-C(8)	-146.58(14)
C(1)-C(6)-C(7)-C(8)	30.70(18)
C(5)-C(6)-C(7)-C(18)	94.15(17)
C(1)-C(6)-C(7)-C(18)	-88.57(16)
S(1)-N(2)-C(8)-C(9)	138.96(11)
S(1)-N(2)-C(8)-C(7)	-96.20(13)
C(6)-C(7)-C(8)-N(2)	-176.97(12)
C(18)-C(7)-C(8)-N(2)	-55.92(16)
C(6)-C(7)-C(8)-C(9)	-53.37(16)
C(18)-C(7)-C(8)-C(9)	67.68(15)

C(1)-N(1)-C(9)-O(1)	176.49(15)
C(17)-N(1)-C(9)-O(1)	4.0(2)
C(1)-N(1)-C(9)-C(8)	-7.3(2)
C(17)-N(1)-C(9)-C(8)	-179.78(14)
N(2)-C(8)-C(9)-O(1)	-14.80(19)
C(7)-C(8)-C(9)-O(1)	-139.71(15)
N(2)-C(8)-C(9)-N(1)	168.86(12)
C(7)-C(8)-C(9)-N(1)	43.95(17)
O(4)-S(1)-C(10)-C(15)	-130.49(13)
O(3)-S(1)-C(10)-C(15)	0.57(15)
N(2)-S(1)-C(10)-C(15)	114.53(14)
O(4)-S(1)-C(10)-C(11)	47.93(15)
O(3)-S(1)-C(10)-C(11)	178.99(12)
N(2)-S(1)-C(10)-C(11)	-67.04(14)
C(15)-C(10)-C(11)-C(12)	0.9(3)
S(1)-C(10)-C(11)-C(12)	-177.50(13)
C(10)-C(11)-C(12)-C(13)	-0.3(3)
C(11)-C(12)-C(13)-C(14)	-0.2(3)
C(11)-C(12)-C(13)-C(16)	-179.8(2)
C(12)-C(13)-C(14)-C(15)	0.1(3)
C(16)-C(13)-C(14)-C(15)	179.78(19)
C(11)-C(10)-C(15)-C(14)	-0.9(2)
S(1)-C(10)-C(15)-C(14)	177.46(13)
C(13)-C(14)-C(15)-C(10)	0.4(3)
C(19)-N(3)-C(18)-O(2)	1.2(2)
C(21)-N(3)-C(18)-O(2)	170.44(16)
C(19)-N(3)-C(18)-C(7)	-179.47(13)
C(21)-N(3)-C(18)-C(7)	-10.2(2)
C(6)-C(7)-C(18)-O(2)	59.41(18)
C(8)-C(7)-C(18)-O(2)	-59.34(18)

C(6)-C(7)-C(18)-N(3)	-119.96(15)
C(8)-C(7)-C(18)-N(3)	121.29(15)
C(18)-N(3)-C(19)-C(20)	85.9(2)
C(21)-N(3)-C(19)-C(20)	-84.4(2)
C(18)-N(3)-C(21)-C(22)	97.2(2)
C(19)-N(3)-C(21)-C(22)	-93.4(2)

Symmetry transformations used to generate equivalent atoms:

Table S21. Hydrogen bonds for CCDC 1415448 [A and deg.].

D-H...A	d(D-H)	d(H...A)	d(D...A)	<(DHA)

References

1. Voica, A.-F., Mendoza, A., Gutekunst, W. R., Fraga, J. O. & Baran, P. S. Guided desaturation of unactivated aliphatics. *Nat. Chem.* **4**, 629-635 (2012).
2. Sakamoto, K., Sato, K., Shigenaga, A., Tsuji, K., Tsuda, S., Hibino, H. & Nishiuchi, Y. Synthetic procedure for *N*‑Fmoc amino acyl‑*N*‑sulfanylethylaniline linker as crypto-peptide thioester precursor with application to native chemical ligation. *J. Org. Chem.* **77**, 6948-6958 (2012).
3. Lima, E. C., Souza, C. C., Soares, R. O., Vaz, B. G., Eberlin, M. N., Dias, A. G. & Costa, P. R. R. DBU as a catalyst for the synthesis of amides via aminolysis of methyl esters. *J. Braz. Chem. Soc.* **22**, 2186-2190 (2011).
4. Maegawa, T., Akashi, A., Esaki, H., Aoki, F., Sajiki, H. & Hirota, K. Efficient and selective deuteration of phenylalanine derivatives catalyzed by Pd/C. *Synlett* **5**, 845-847 (2005).
5. Park, J., Tian, G. R. & Kim, D. H. Synthesis of optically active 2-alkyl-3,4-iminobutanoic acids. *β*-amino acids containing an aziridine heterocycle. *J. Org. Chem.* **66**, 3696-3703 (2001).
6. Miura, T., Funakoshi, Y. & Murakami, M. Intramolecular dearomatizing [3 + 2] annulation of α‑imino carbenoids with aryl rings furnishing 3,4-fused indole skeletons. *J. Am. Chem. Soc.* **136**, 2272-2275 (2014).

The ^{1}H and ^{13}C NMR spectra of compounds

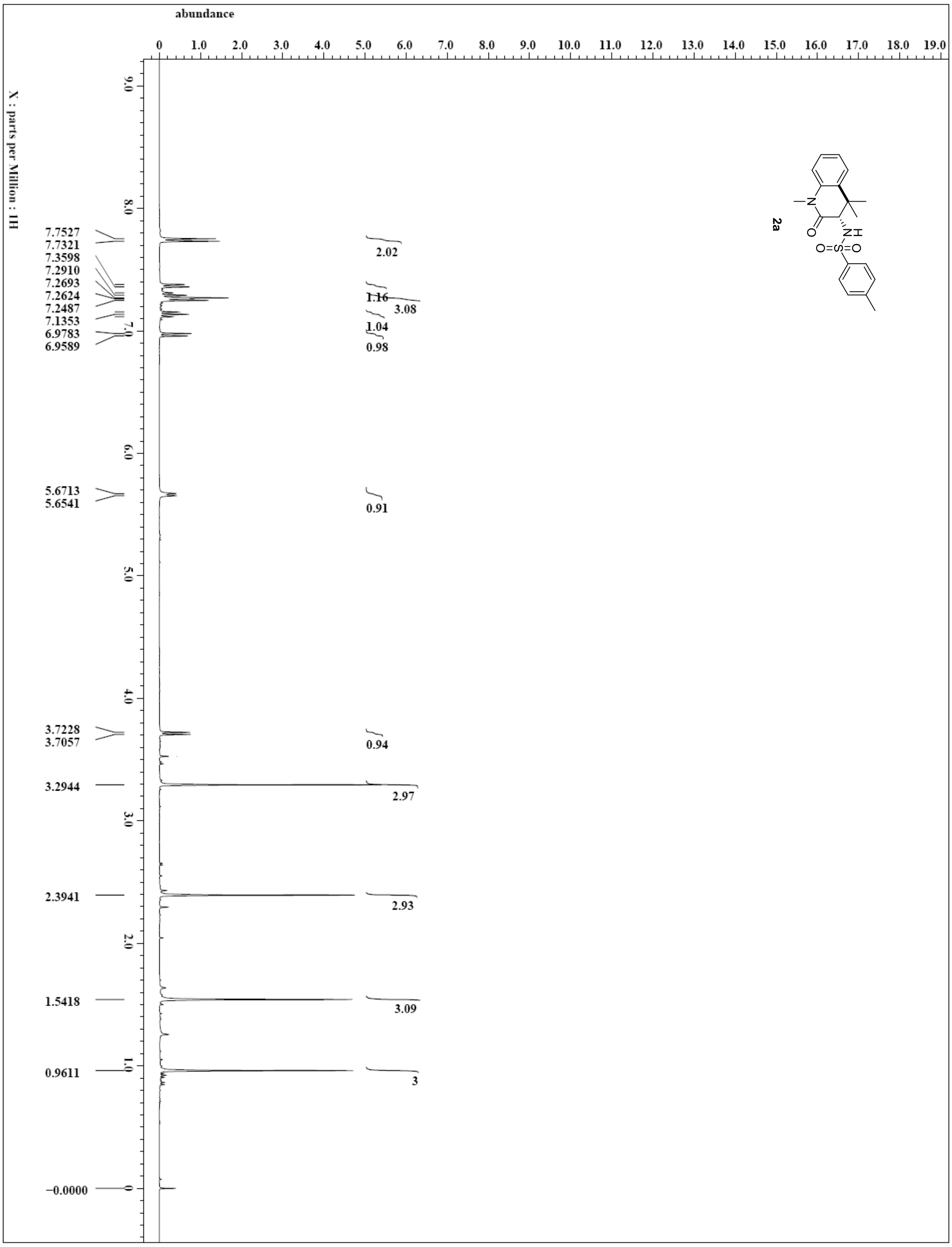

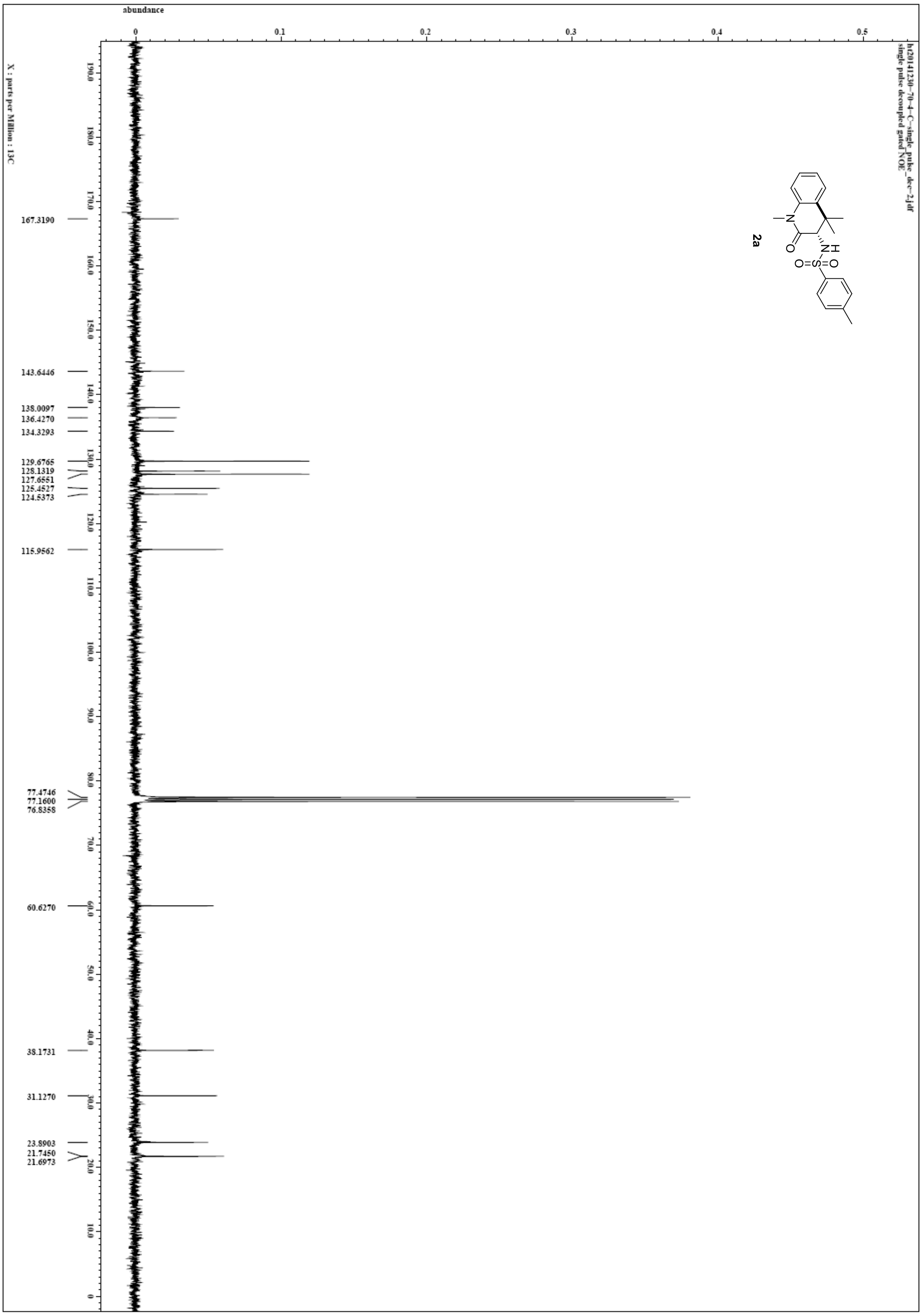

ht20141230--70--4--C--single_pulse_dec--2.jdf
single pulse decoupled gated NOE
abundance
X : parts per Million : 13C
2a
167.3190
143.6446
138.0097
136.4270
134.3293
129.6765
128.1319
127.6551
125.4527
124.5373
115.9562
77.4746
77.1600
76.8358
60.6270
38.1731
31.1270
23.8903
21.7450
21.6973

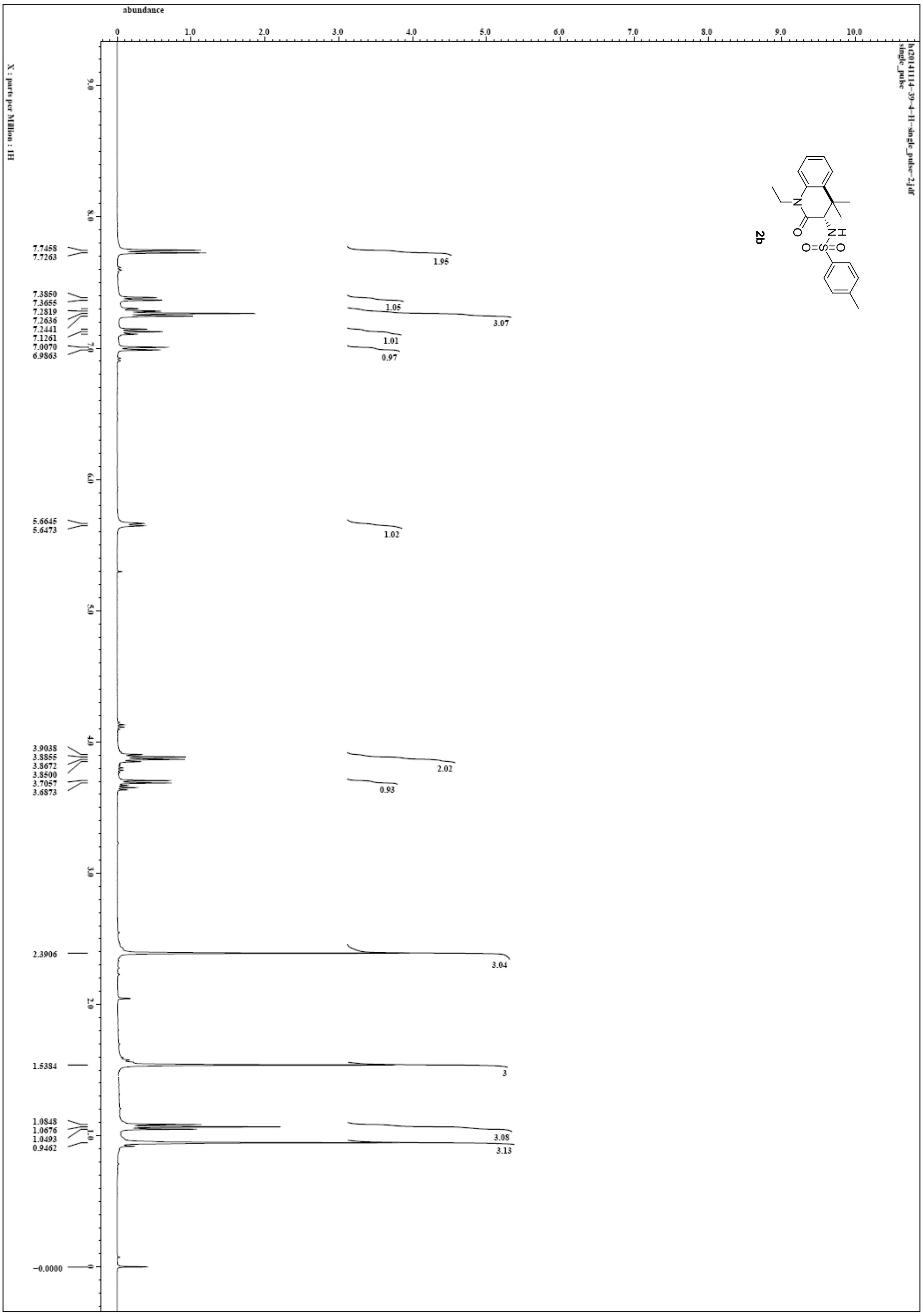
abundance
X : parts per Million : 1H
2b
7.7458
7.7263
7.3850
7.3655
7.2819
7.2636
7.2441
7.1261
7.0070
6.9863
5.6645
5.6473
3.9038
3.8855
3.8672
3.8500
3.7057
3.6873
2.3906
1.5384
1.0848
1.0676
1.0493
0.9462
-0.0000
1.95
1.05
3.07
1.01
0.97
1.02
2.02
0.93
3.04
3
3.08
3.13

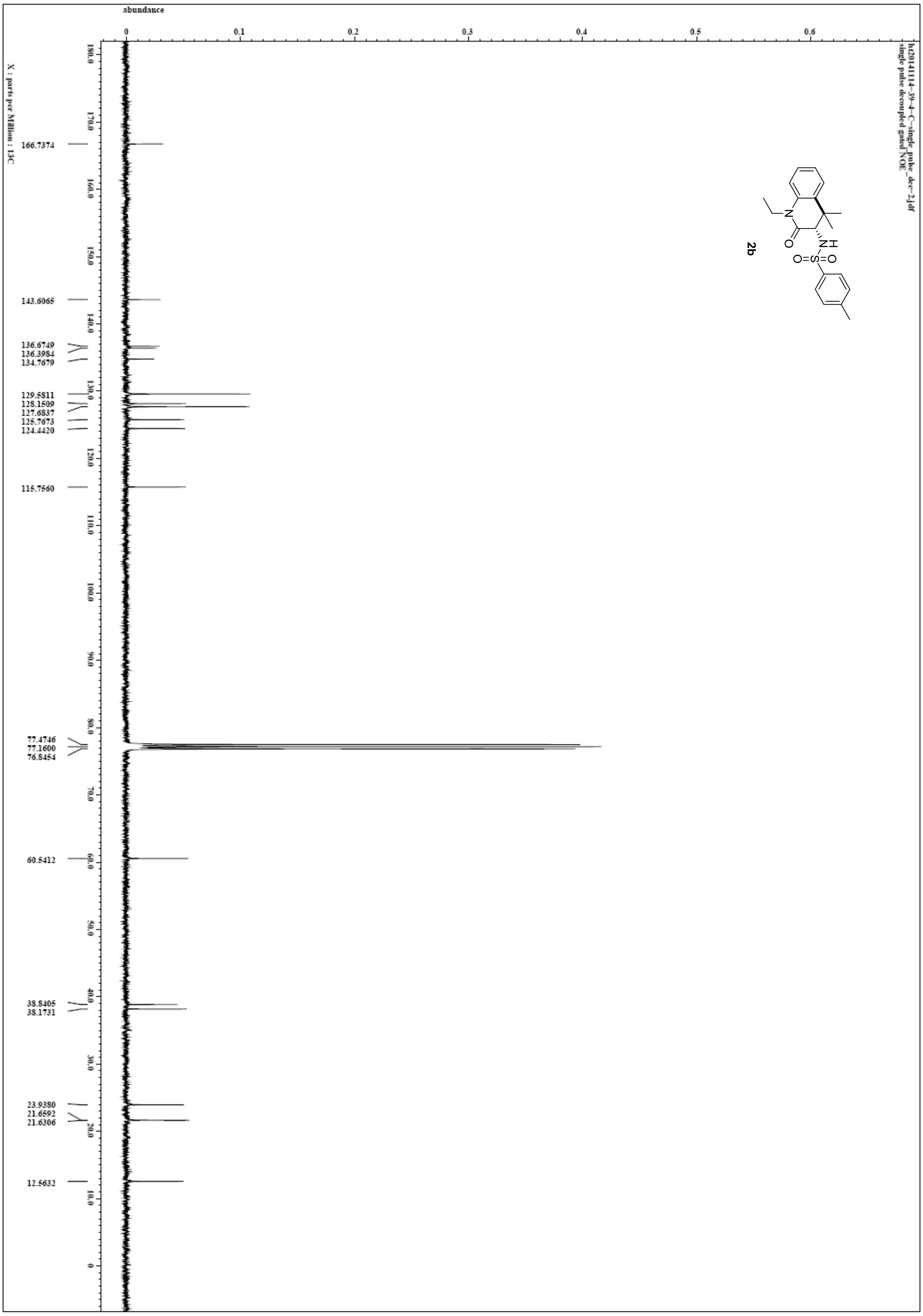
abundance
X : parts per Million : 13C
2b
166.7374
143.6065
136.6749
136.3984
134.7679
129.5811
128.1509
127.6837
125.7673
124.4420
115.7560
77.4746
77.1600
76.8454
60.5412
38.8405
38.1731
23.9380
21.6592
21.6306
12.5632

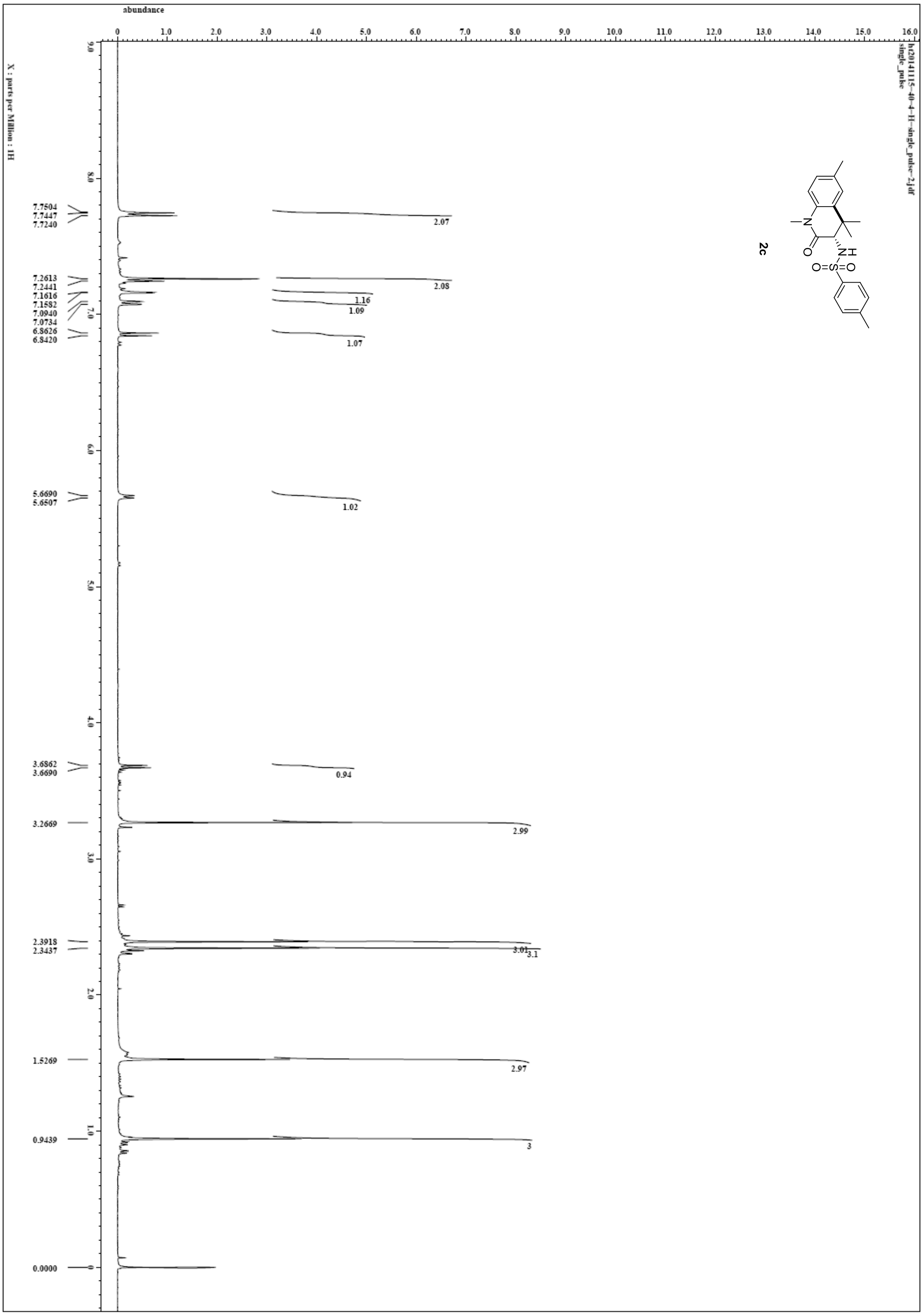
abundance
X : parts per Million : 1H
7.7504
7.7447
7.7240
7.2613
7.2441
7.1616
7.1582
7.0940
7.0734
6.8626
6.8420
5.6690
5.6507
3.6862
3.6690
3.2669
2.3918
2.3437
1.5269
0.9439
0.0000
2.07
2.08
1.16
1.09
1.07
1.02
0.94
2.99
3.01
3.1
2.97
3
2c

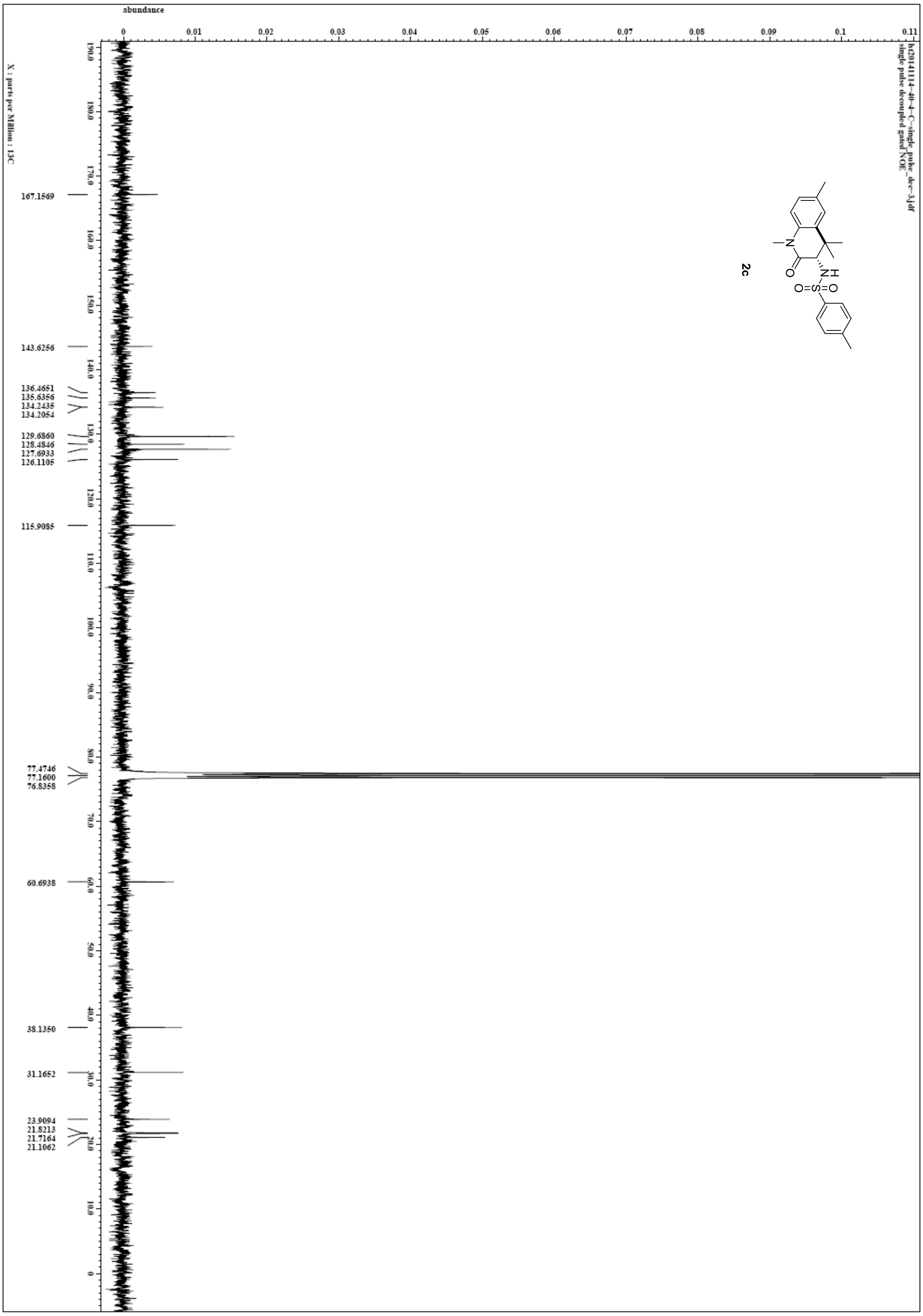
abundance
X : parts per Million : 13C
167.1569
143.6256
136.4651
135.6356
134.2435
134.2054
129.6860
128.4846
127.6933
126.1105
115.9085
77.4746
77.1600
76.8358
60.6938
38.1350
31.1652
23.9094
21.8213
21.7164
21.1062
2c

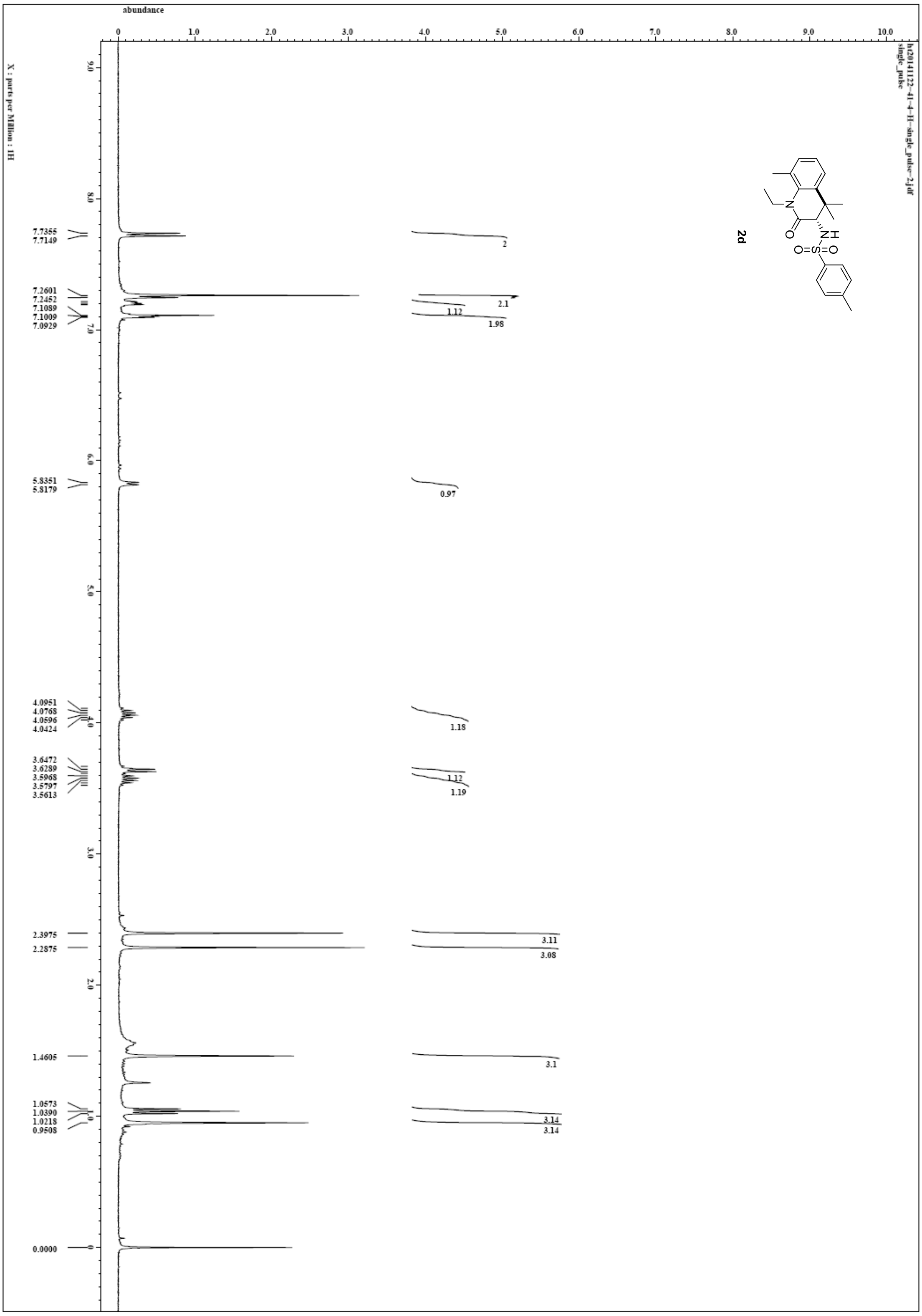
abundance
X : parts per Million : 1H
2d
7.7355
7.7149
7.2601
7.2452
7.1089
7.1009
7.0929
5.8351
5.8179
4.0951
4.0768
4.0596
4.0424
3.6472
3.6289
3.5968
3.5797
3.5613
2.3975
2.2875
1.4605
1.0573
1.0390
1.0218
0.9508
0.0000

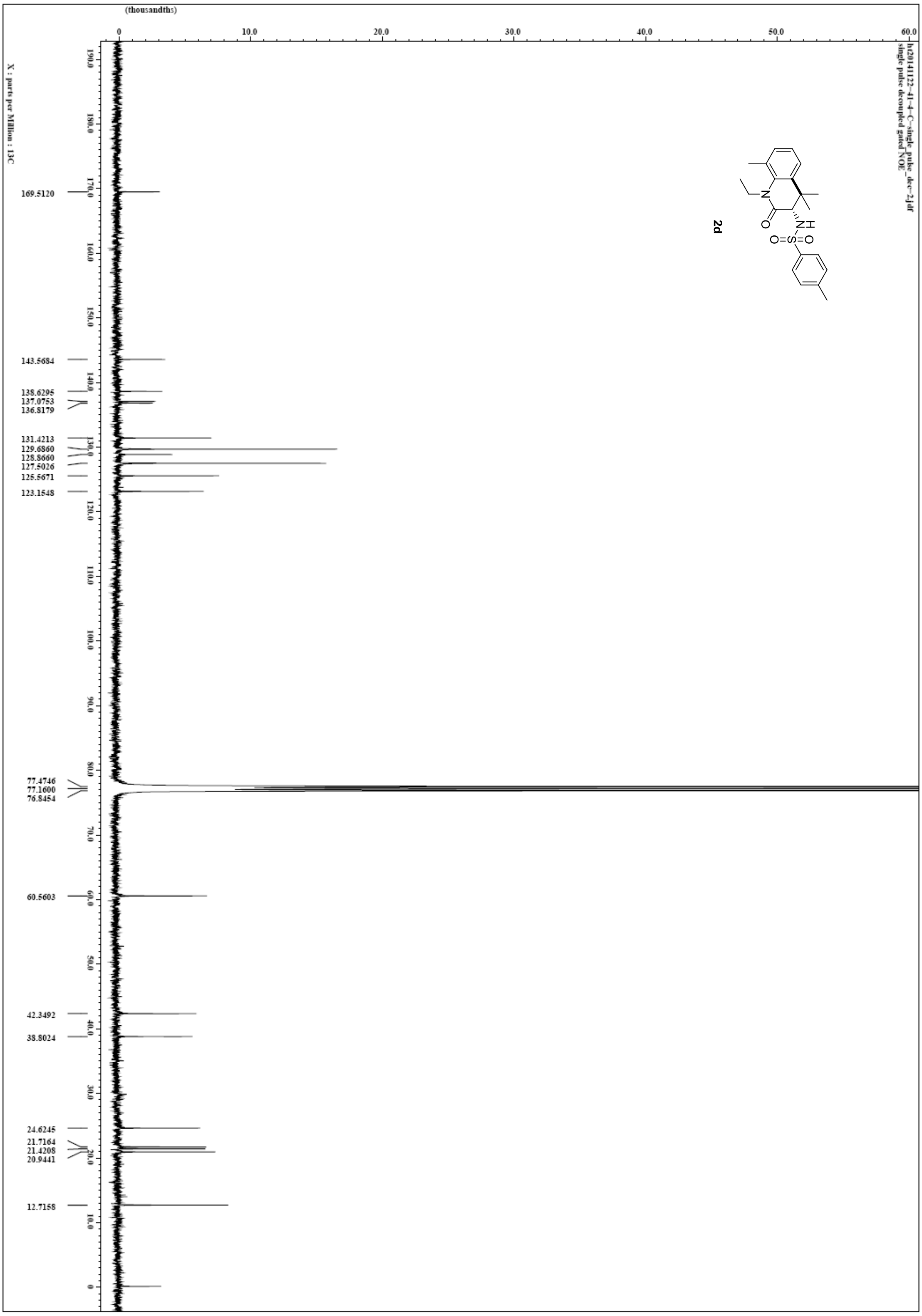
(thousandths)
X : parts per Million : 13C
2d
169.5120
143.5684
138.6295
137.0753
136.8179
131.4213
129.6860
128.8660
127.5026
125.5671
123.1548
77.4746
77.1600
76.8454
60.5603
42.3492
38.8024
24.6245
21.7164
21.4208
20.9441
12.7158

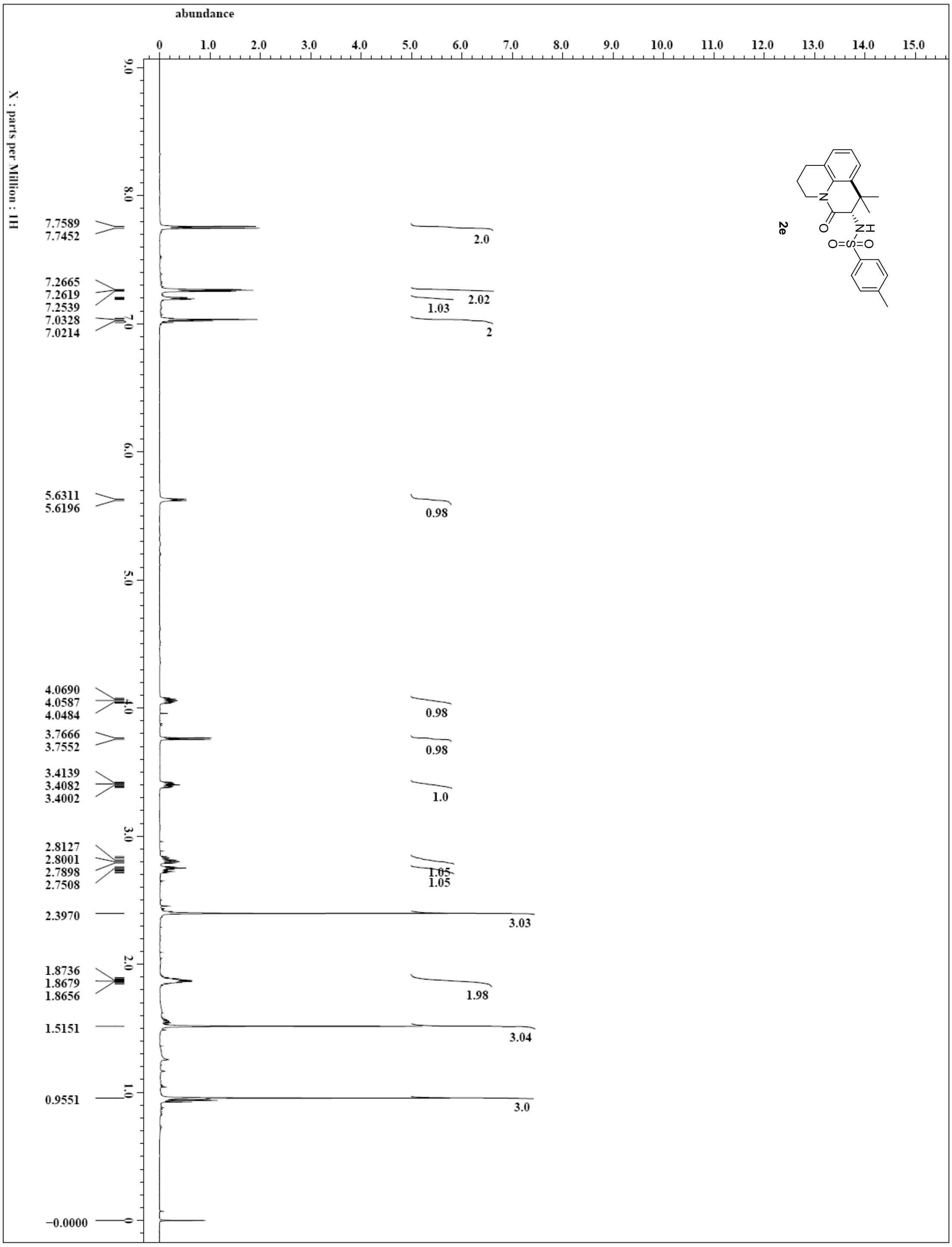
abundance
X : parts per Million : 1H
7.7589
7.7452
7.2665
7.2619
7.2539
7.0328
7.0214
5.6311
5.6196
4.0690
4.0587
4.0484
3.7666
3.7552
3.4139
3.4082
3.4002
2.8127
2.8001
2.7898
2.7508
2.3970
1.8736
1.8679
1.8656
1.5151
0.9551
−0.0000
2.0
2.02
1.03
2
0.98
0.98
0.98
1.0
1.05
1.05
3.03
1.98
3.04
3.0
2e

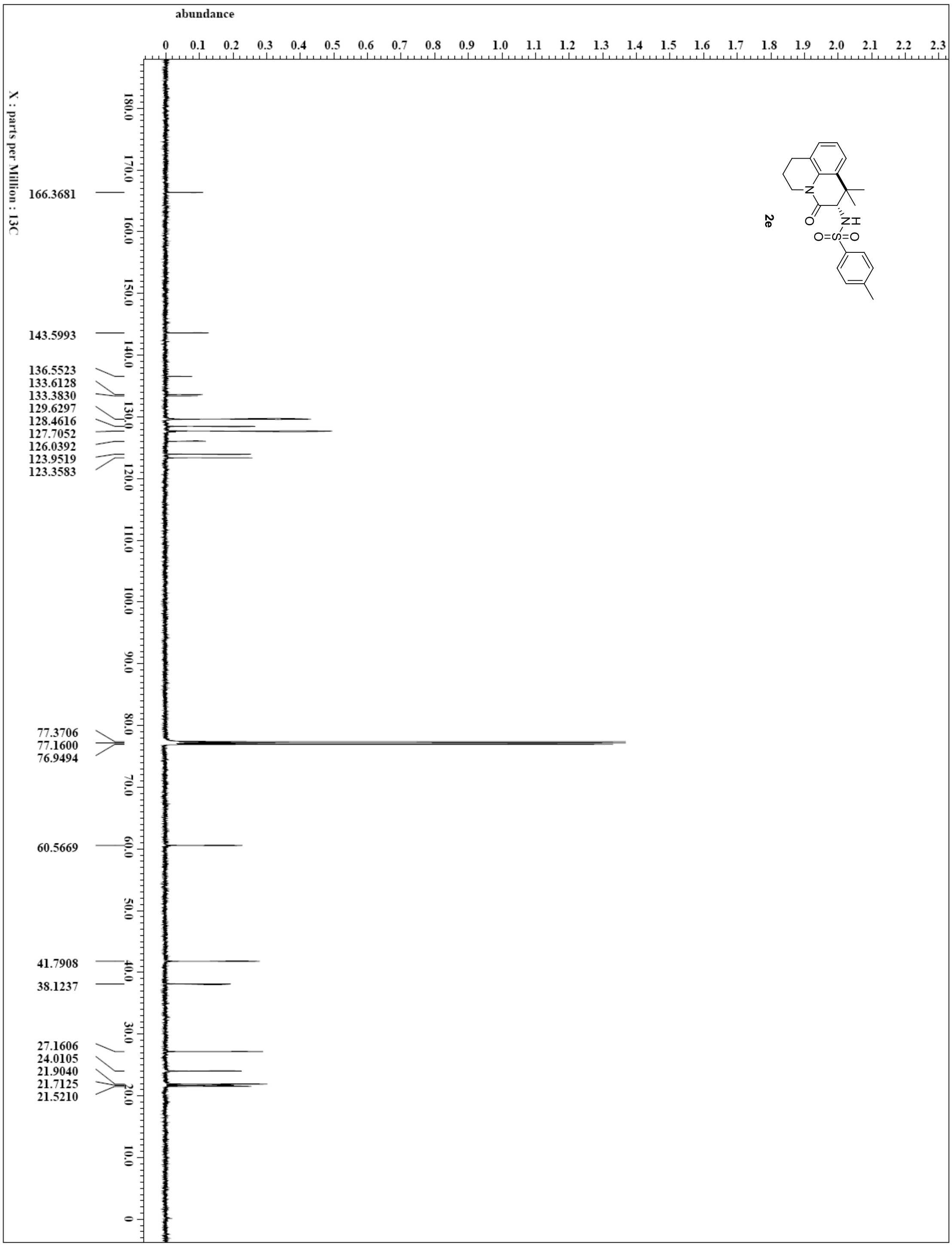
abundance
X : parts per Million : 13C
166.3681
143.5993
136.5523
133.6128
133.3830
129.6297
128.4616
127.7052
126.0392
123.9519
123.3583
77.3706
77.1600
76.9494
60.5669
41.7908
38.1237
27.1606
24.0105
21.9040
21.7125
21.5210
2e

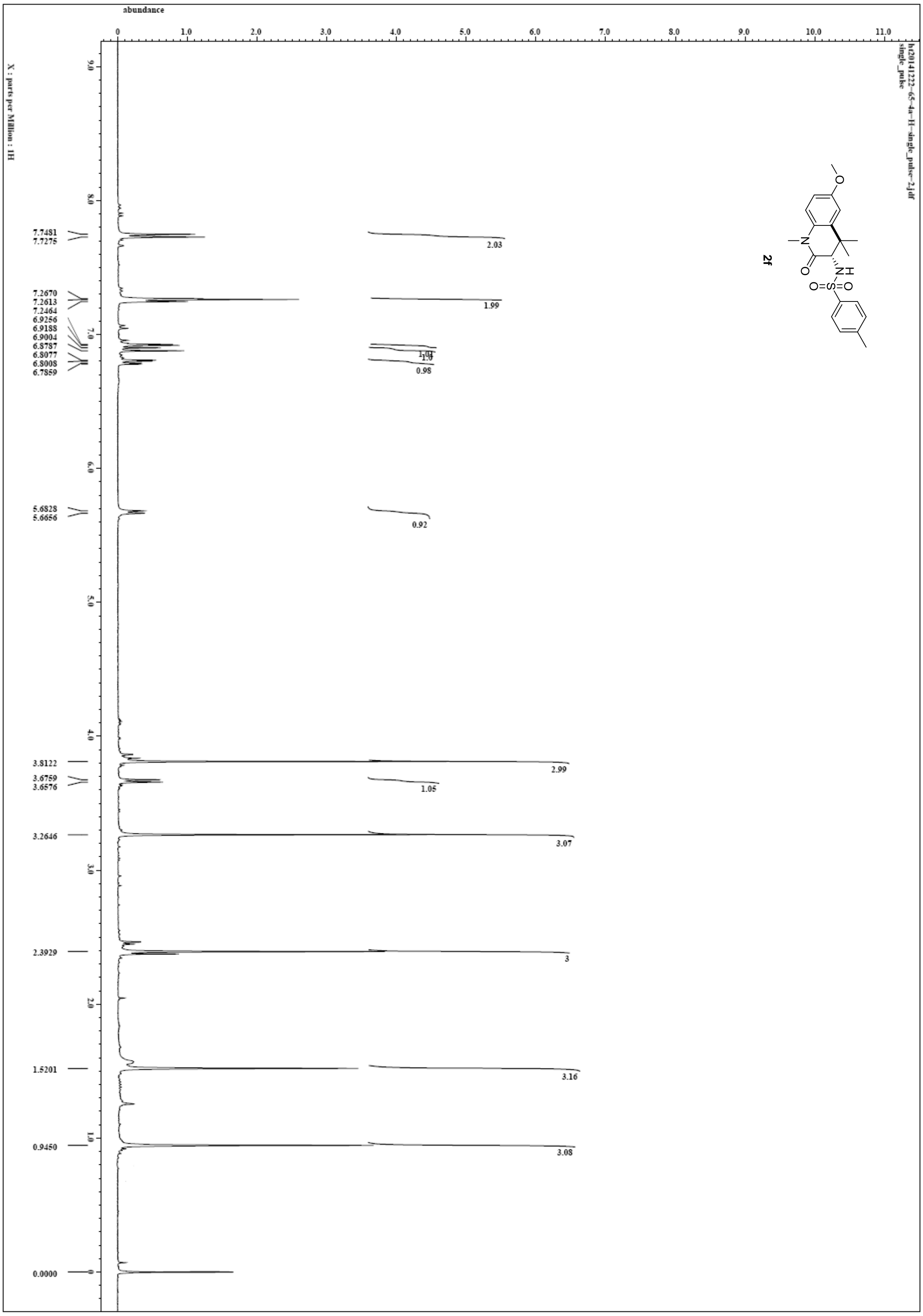
abundance
X : parts per Million : 1H
single_pulse
2f
7.7481
7.7275
7.2670
7.2613
7.2464
6.9256
6.9188
6.9004
6.8787
6.8077
6.8008
6.7859
5.6828
5.6656
3.8122
3.6759
3.6576
3.2646
2.3929
1.5201
0.9450
0.0000
2.03
1.99
0.98
0.92
2.99
1.05
3.07
3
3.16
3.08

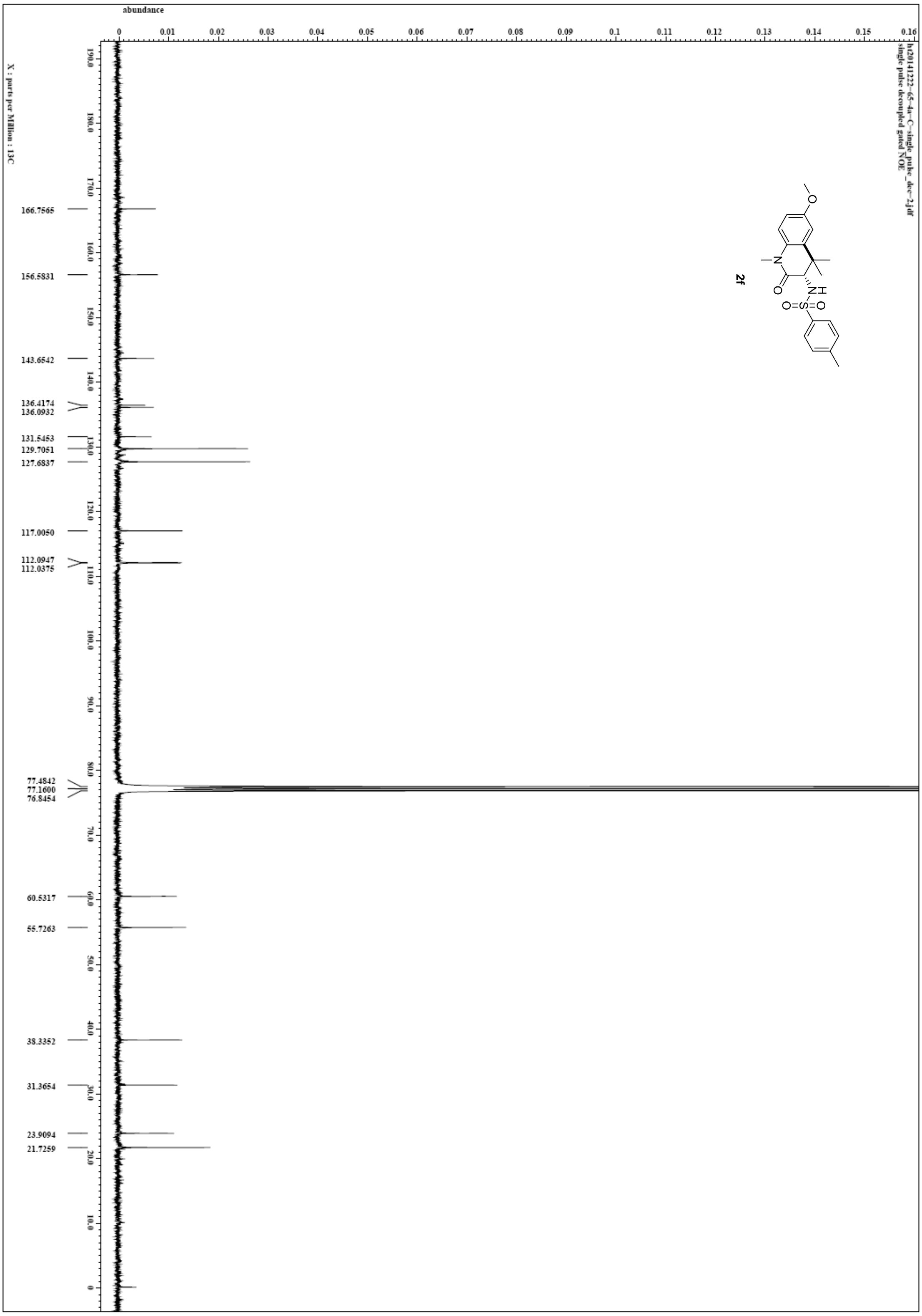
abundance
X : parts per Million : 13C
hi20141222-65-4a-C--single_pulse_dec-2.jdf
single pulse decoupled gated NOE
2f
166.7565
156.5831
143.6542
136.4174
136.0932
131.5453
129.7051
127.6837
117.0050
112.0947
112.0375
77.4842
77.1600
76.8454
60.5317
55.7263
38.3352
31.3654
23.9094
21.7259

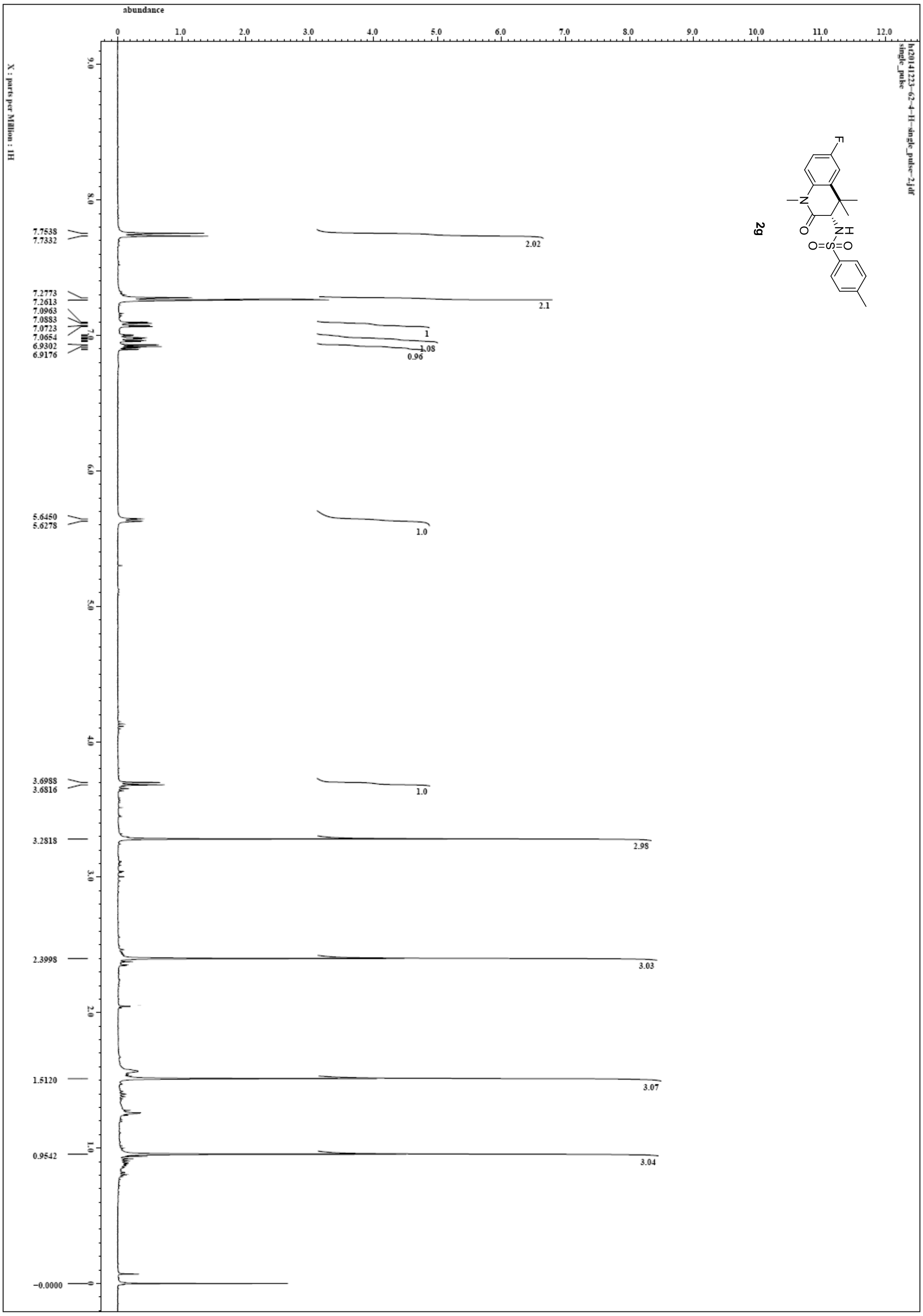

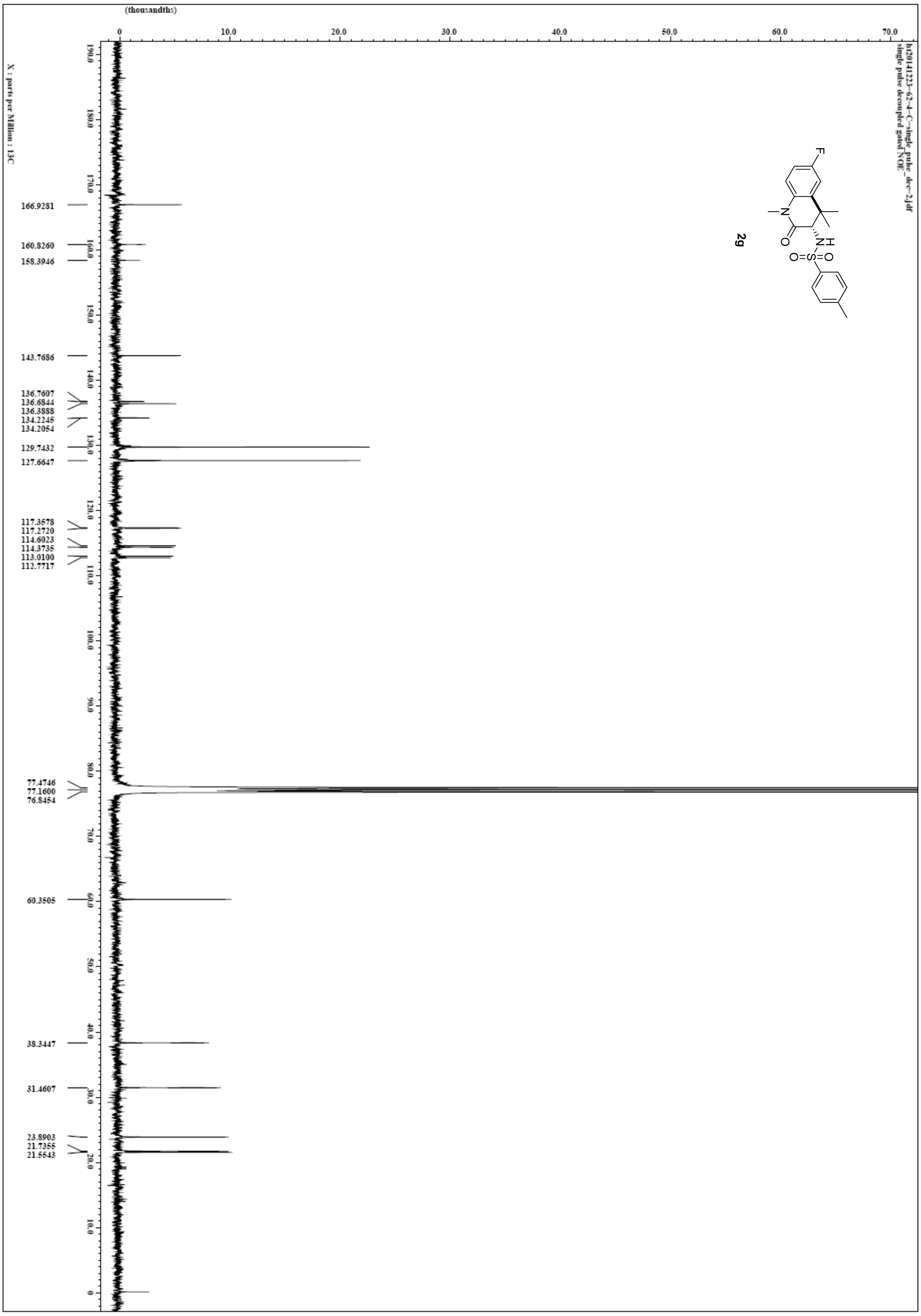
(thousandths)
X : parts per Million : 13C
h20141223--62-4--C--single_pulse_dec--2.jdf
single pulse decoupled gated NOE
2g
166.9281
160.8260
158.3946
143.7686
136.7607
136.6844
136.3888
134.2245
134.2054
129.7432
127.6647
117.3578
117.2720
114.6023
114.3735
113.0100
112.7717
77.4746
77.1600
76.8454
60.3505
38.3447
31.4607
23.8903
21.7355
21.5543

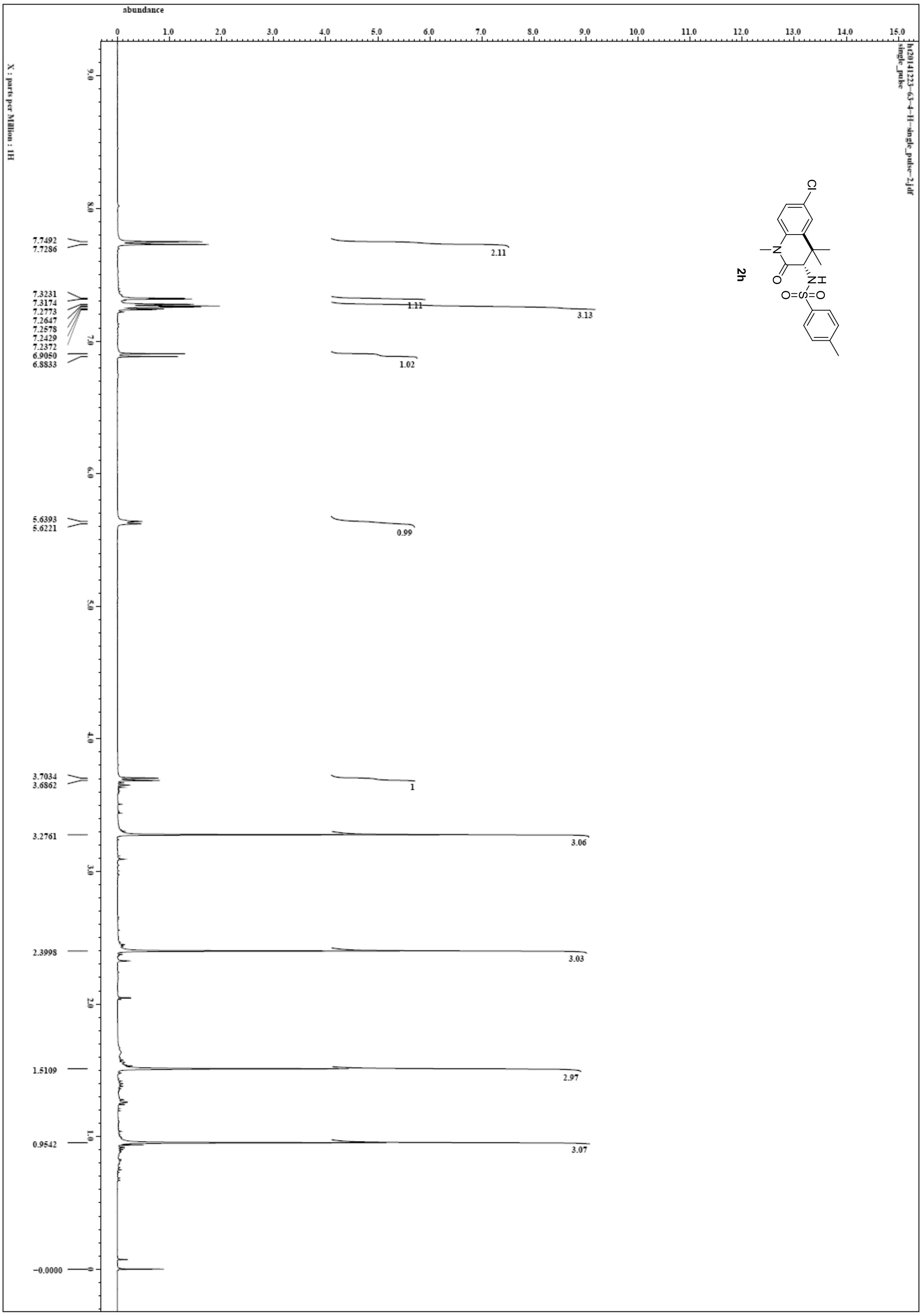
abundance
X : parts per Million : 1H
2h
7.7492
7.7286
7.3231
7.3174
7.2773
7.2647
7.2578
7.2429
7.2372
6.9050
6.8833
5.6393
5.6221
3.7034
3.6862
3.2761
2.3998
1.5109
0.9542
−0.0000
2.11
1.11
3.13
1.02
0.99
1
3.06
3.03
2.97
3.07

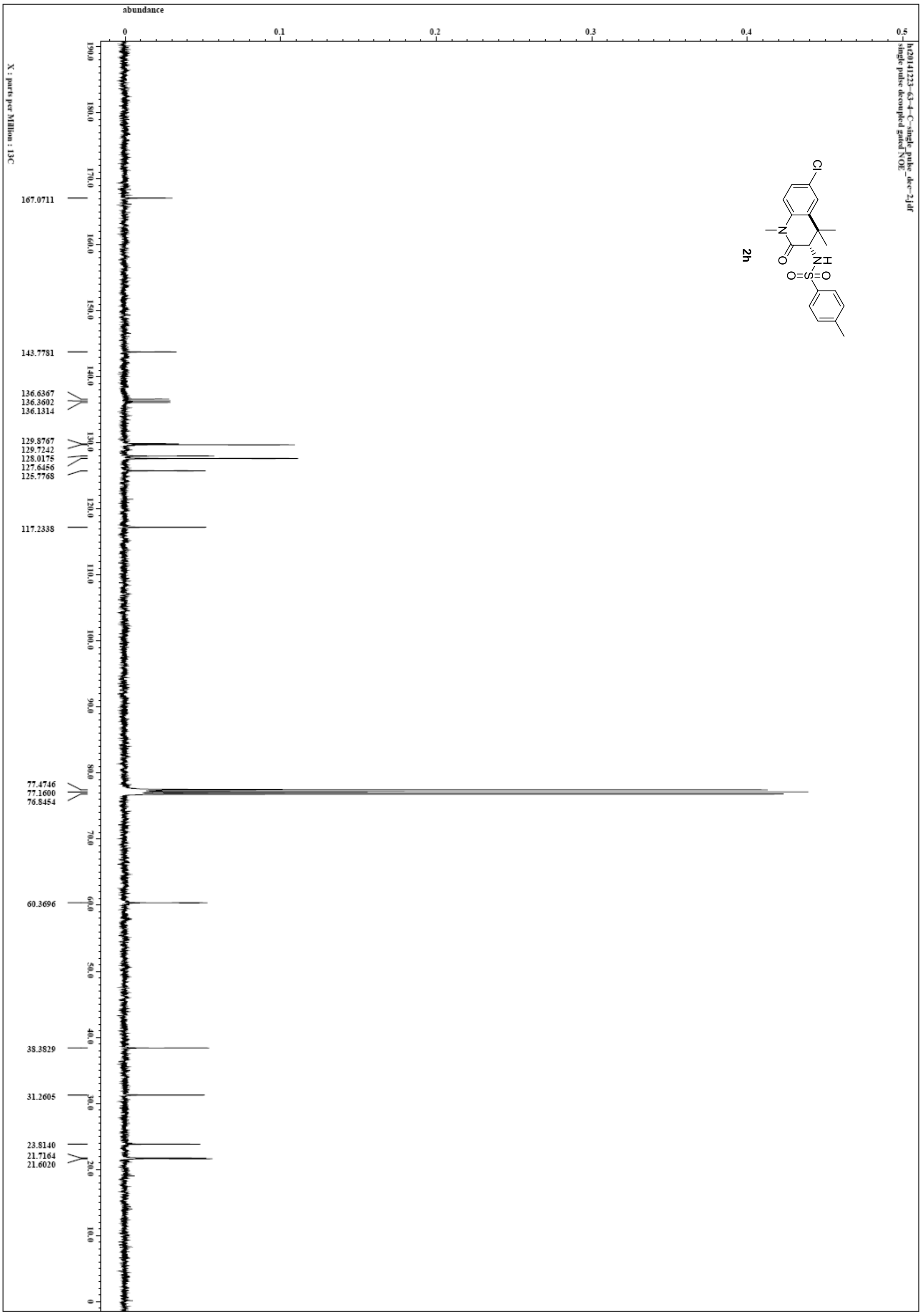
abundance
X : parts per Million : 13C
167.0711
143.7781
136.6367
136.3602
136.1314
129.8767
129.7242
128.0175
127.6456
125.7768
117.2338
77.4746
77.1600
76.8454
60.3696
38.3829
31.2605
23.8140
21.7164
21.6020
2h

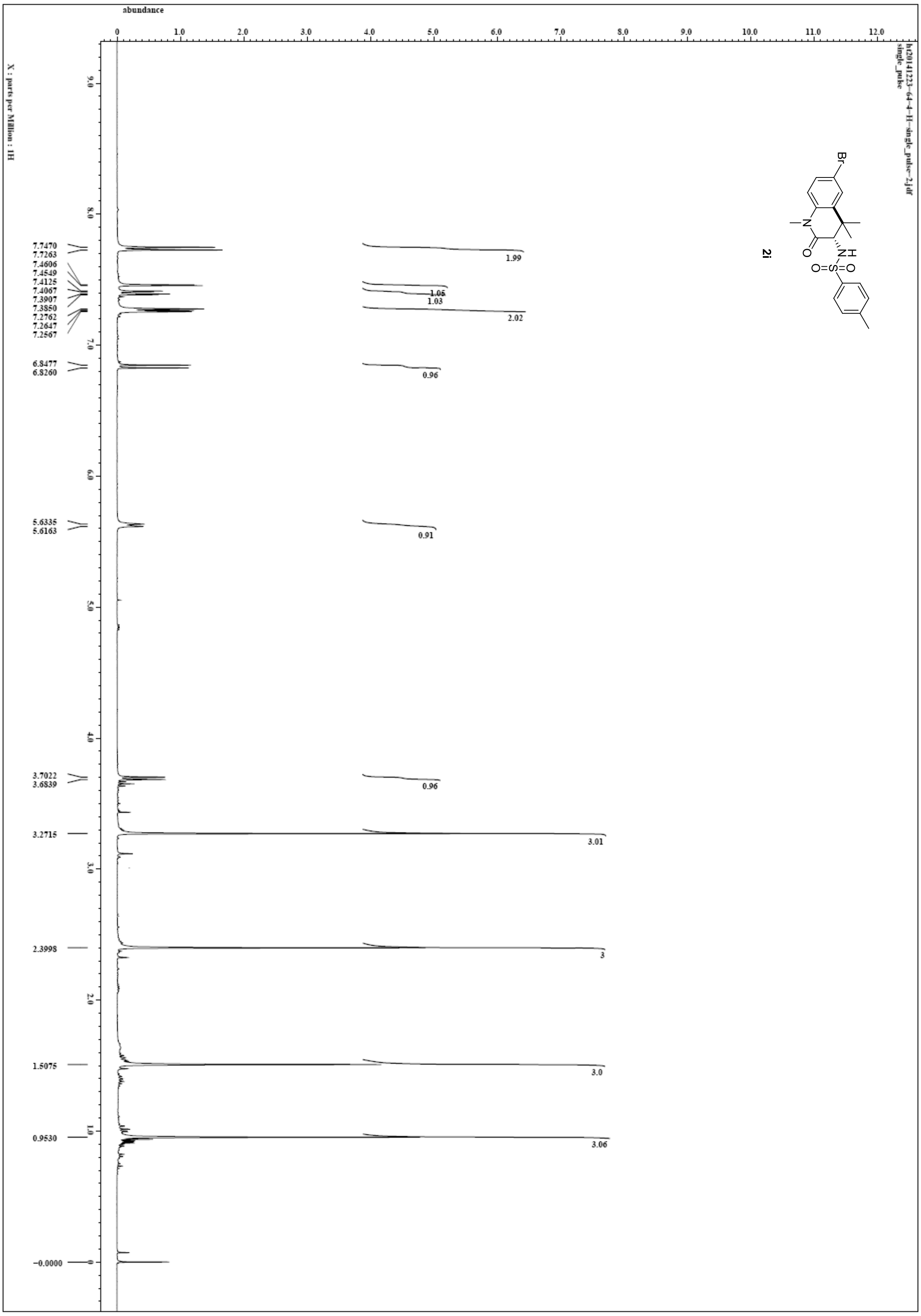
abundance
X : parts per Million : 1H
7.7470
7.7263
7.4606
7.4549
7.4125
7.4067
7.3907
7.3850
7.2762
7.2647
7.2567
6.8477
6.8260
5.6335
5.6163
3.7022
3.6839
3.2715
2.3998
1.5075
0.9530
-0.0000
1.99
1.05
1.03
2.02
0.96
0.91
0.96
3.01
3
3.0
3.06
2i

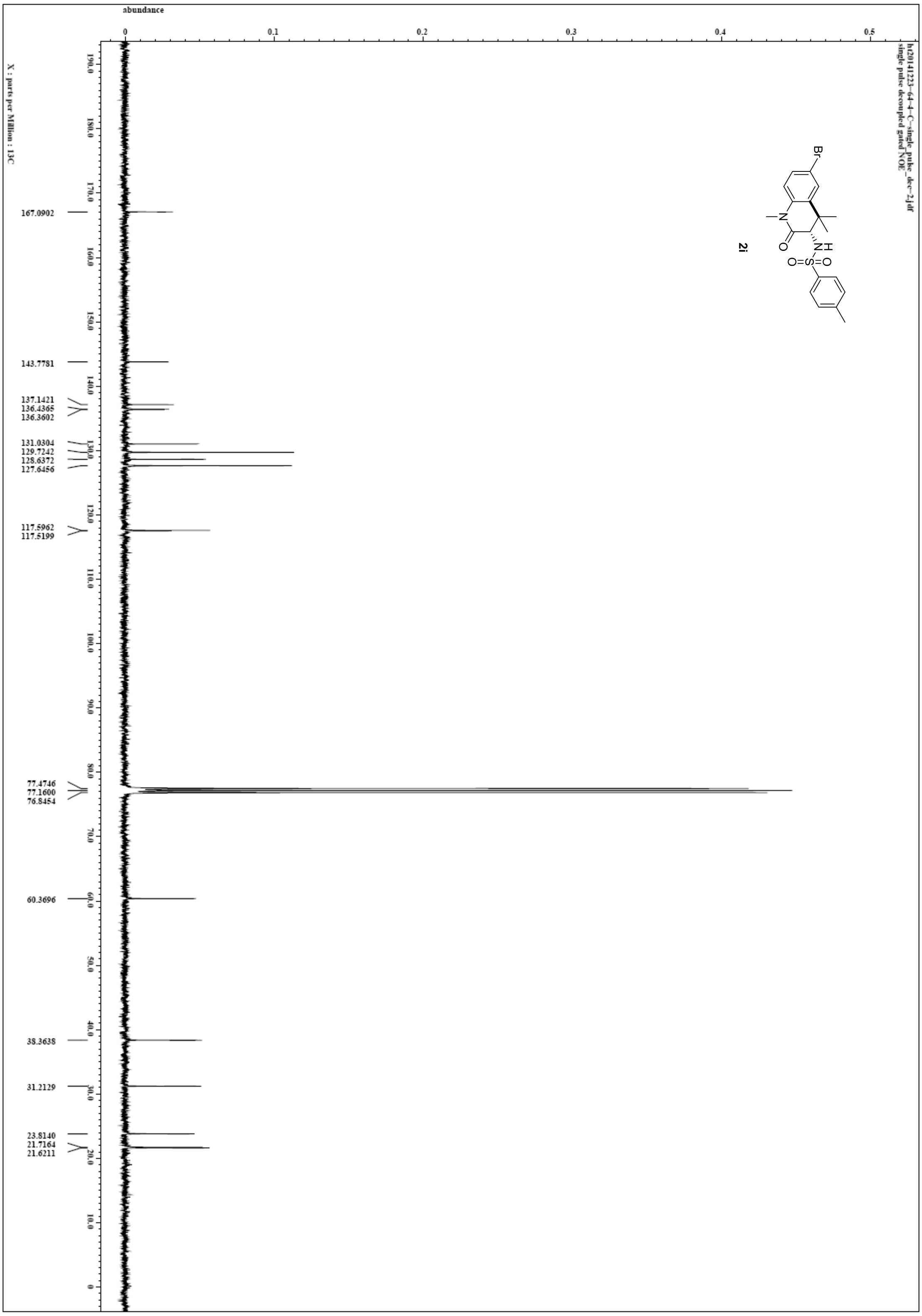
abundance
X : parts per Million : 13C
167.0902
143.7781
137.1421
136.4365
136.3602
131.0304
129.7242
128.6372
127.6456
117.5962
117.5199
77.4746
77.1600
76.8454
60.3696
38.3638
31.2129
23.8140
21.7164
21.6211
2i

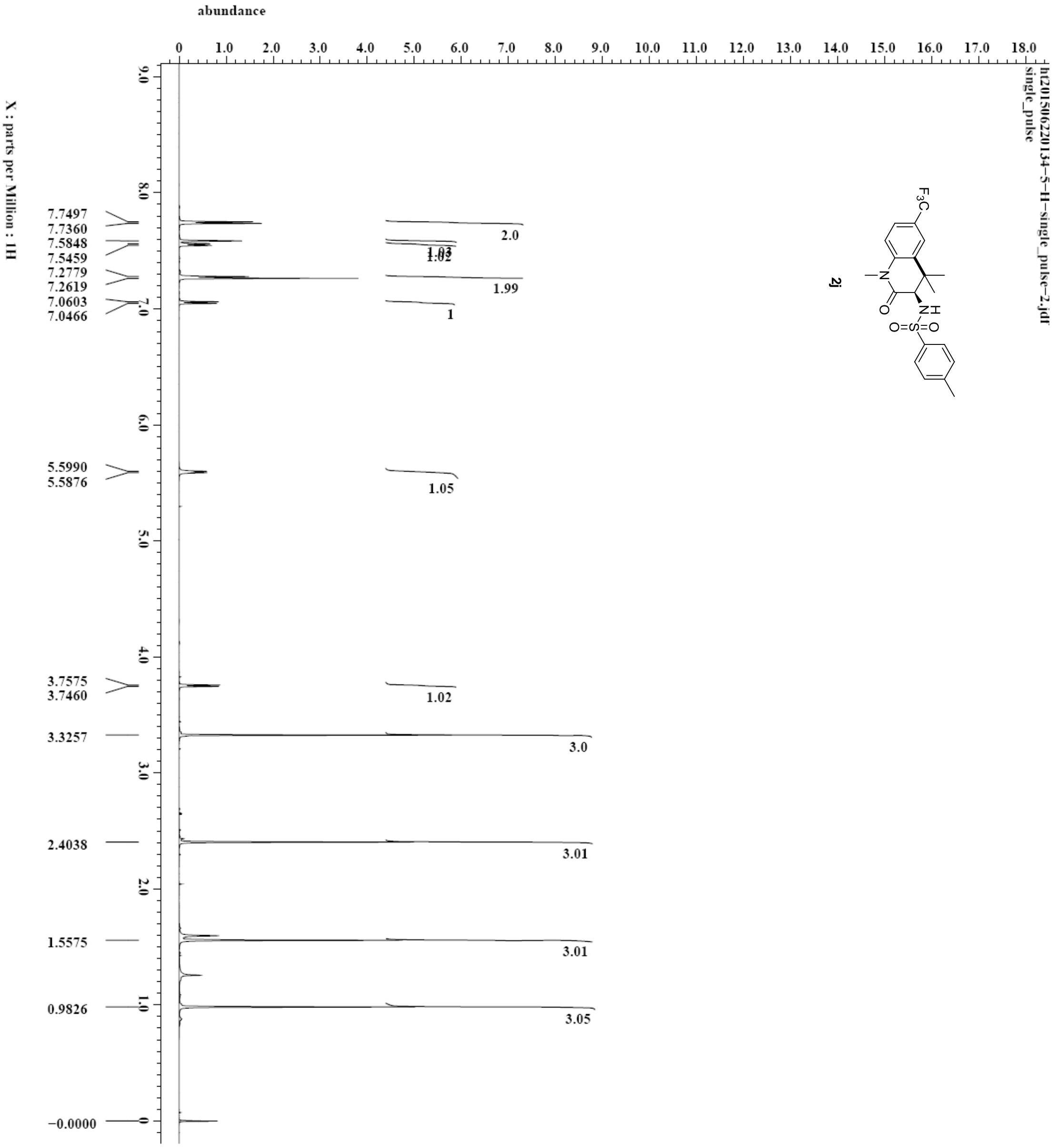
abundance
X : parts per Million : 1H
ht20150622O134-5-H-single_pulse-2.jdf
single_pulse
7.7497
7.7360
7.5848
7.5459
7.2779
7.2619
7.0603
7.0466
5.5990
5.5876
3.7575
3.7460
3.3257
2.4038
1.5575
0.9826
-0.0000
2.0
1.02
1.99
1
1.05
1.02
3.0
3.01
3.01
3.05
2j

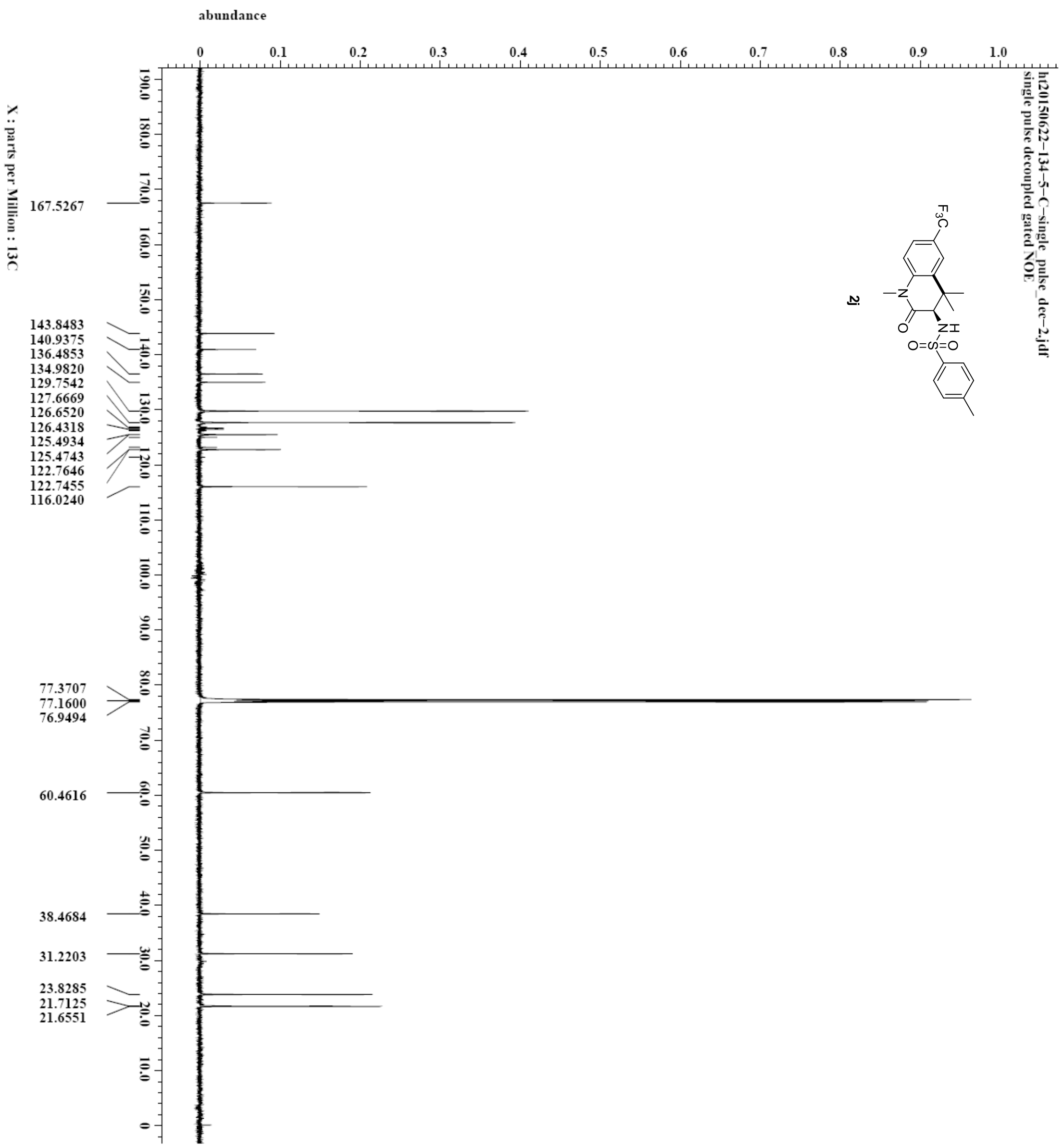
abundance
ht20150622-134-5-C-single_pulse_dec-2.jdf
single pulse decoupled gated NOE
2j
X : parts per Million : 13C
167.5267
143.8483
140.9375
136.4853
134.9820
129.7542
127.6669
126.6520
126.4318
125.4934
125.4743
122.7646
122.7455
116.0240
77.3707
77.1600
76.9494
60.4616
38.4684
31.2203
23.8285
21.7125
21.6551

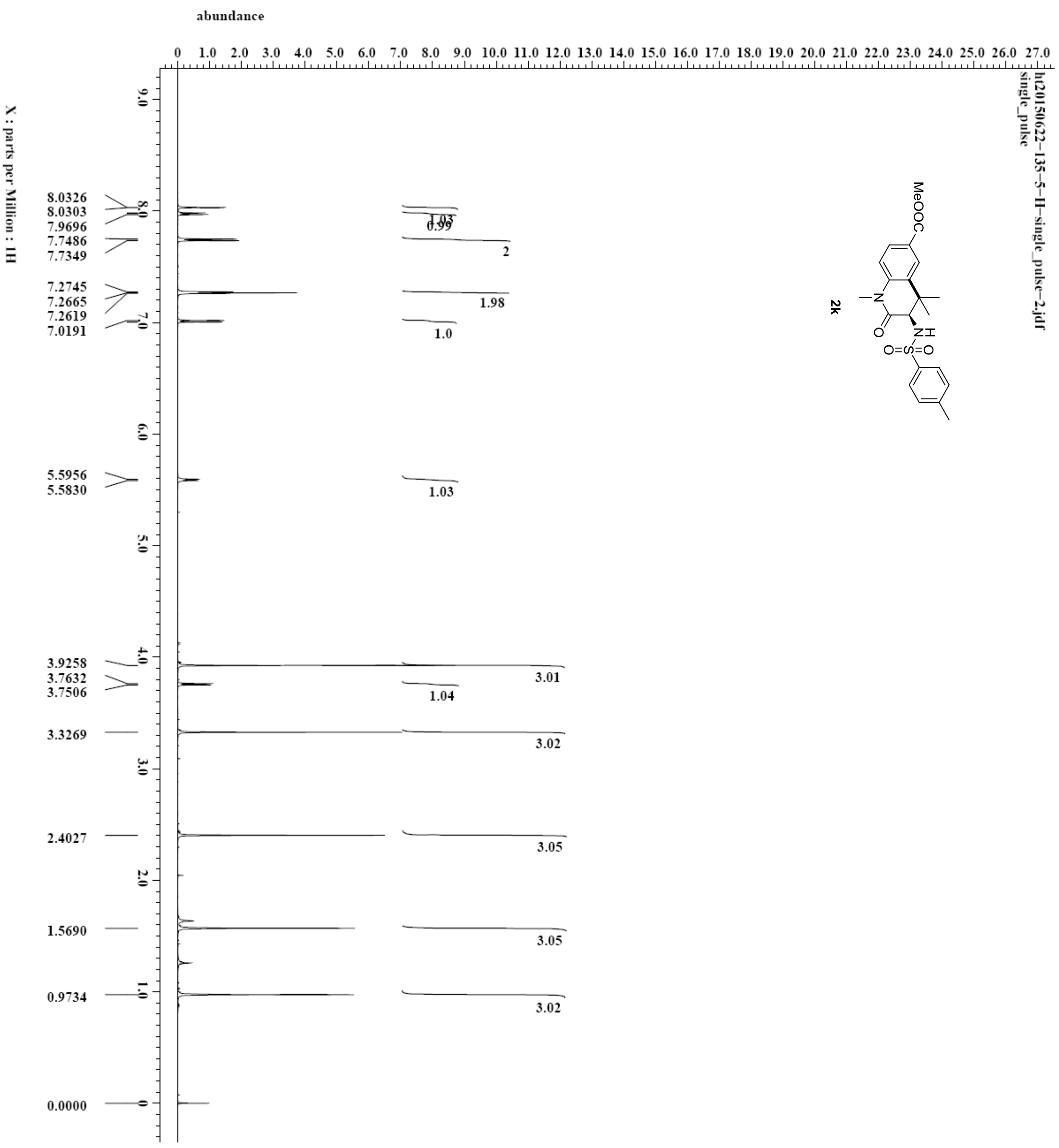
abundance
X : parts per Million : 1H
ht20150622-135-5-H-single_pulse-2.jdf
single_pulse
2k
MeOOC
8.0326
8.0303
7.9696
7.7486
7.7349
7.2745
7.2665
7.2619
7.0191
5.5956
5.5830
3.9258
3.7632
3.7506
3.3269
2.4027
1.5690
0.9734
0.0000
1.03
0.99
2
1.98
1.0
1.03
3.01
1.04
3.02
3.05
3.05
3.02

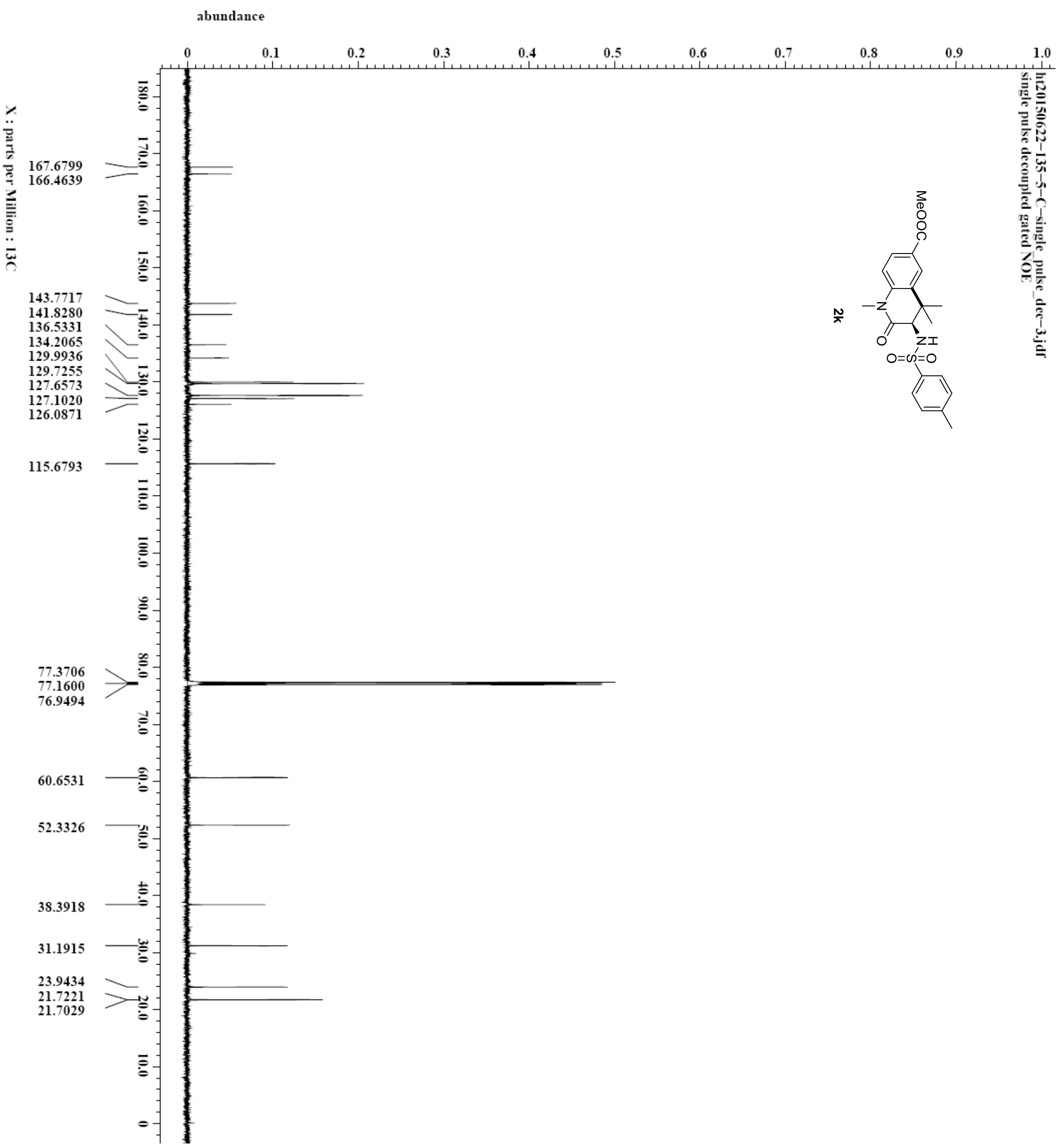
abundance
h20150622-135-5-C-single_pulse_dec-3.jdf
single pulse decoupled gated NOE
X : parts per Million : 13C
2k
MeOOC
167.6799
166.4639
143.7717
141.8280
136.5331
134.2065
129.9936
129.7255
127.6573
127.1020
126.0871
115.6793
77.3706
77.1600
76.9494
60.6531
52.3326
38.3918
31.1915
23.9434
21.7221
21.7029

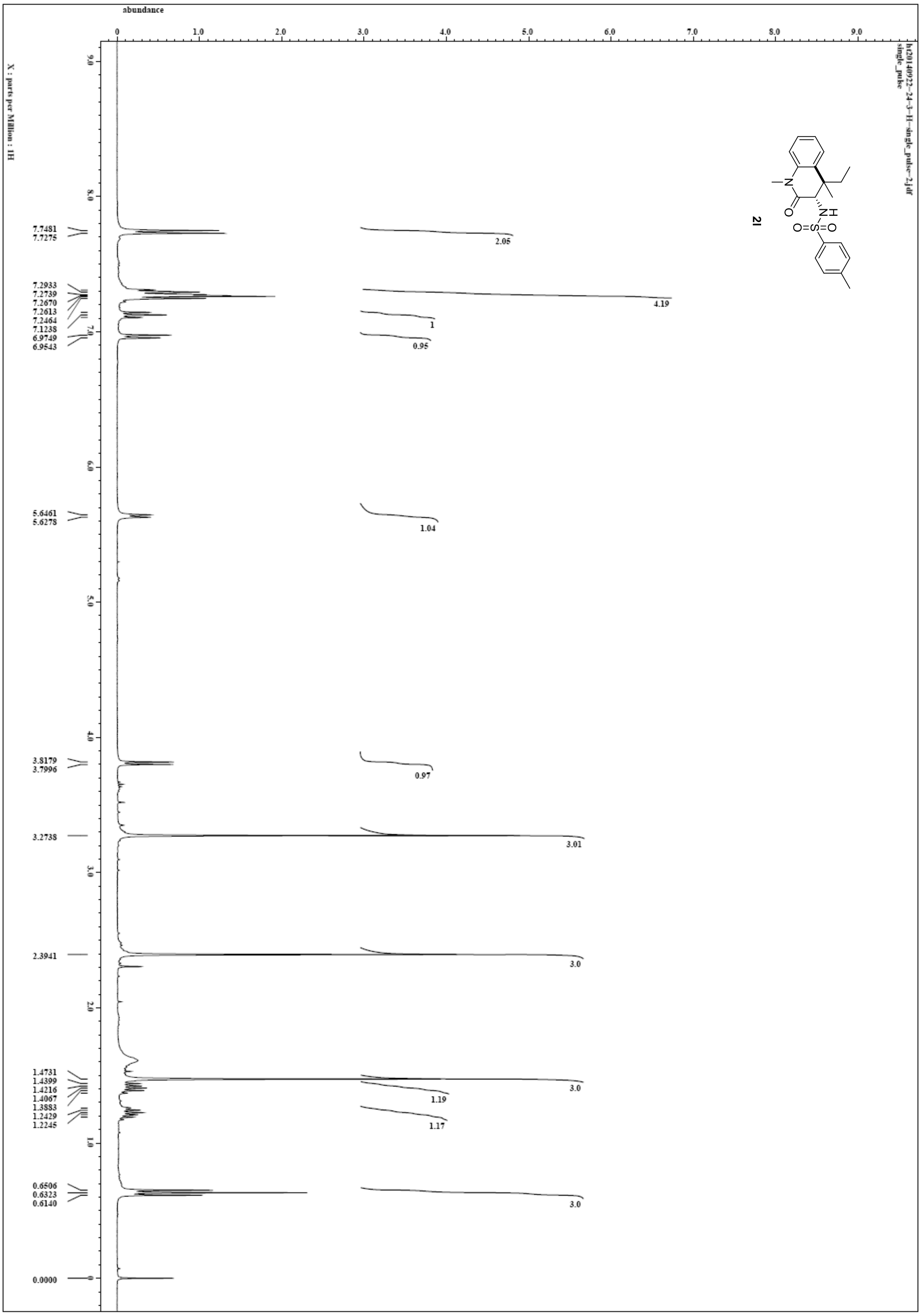
abundance
X : parts per Million : 1H
h20140922--24-3--H--single_pulse-2.jdf
single_pulse
2l
7.7481
7.7275
7.2933
7.2739
7.2670
7.2613
7.2464
7.1238
6.9749
6.9543
5.6461
5.6278
3.8179
3.7996
3.2738
2.3941
1.4731
1.4399
1.4216
1.4067
1.3883
1.2429
1.2245
0.6506
0.6323
0.6140
0.0000
2.05
4.19
1
0.95
1.04
0.97
3.01
3.0
3.0
1.19
1.17
3.0

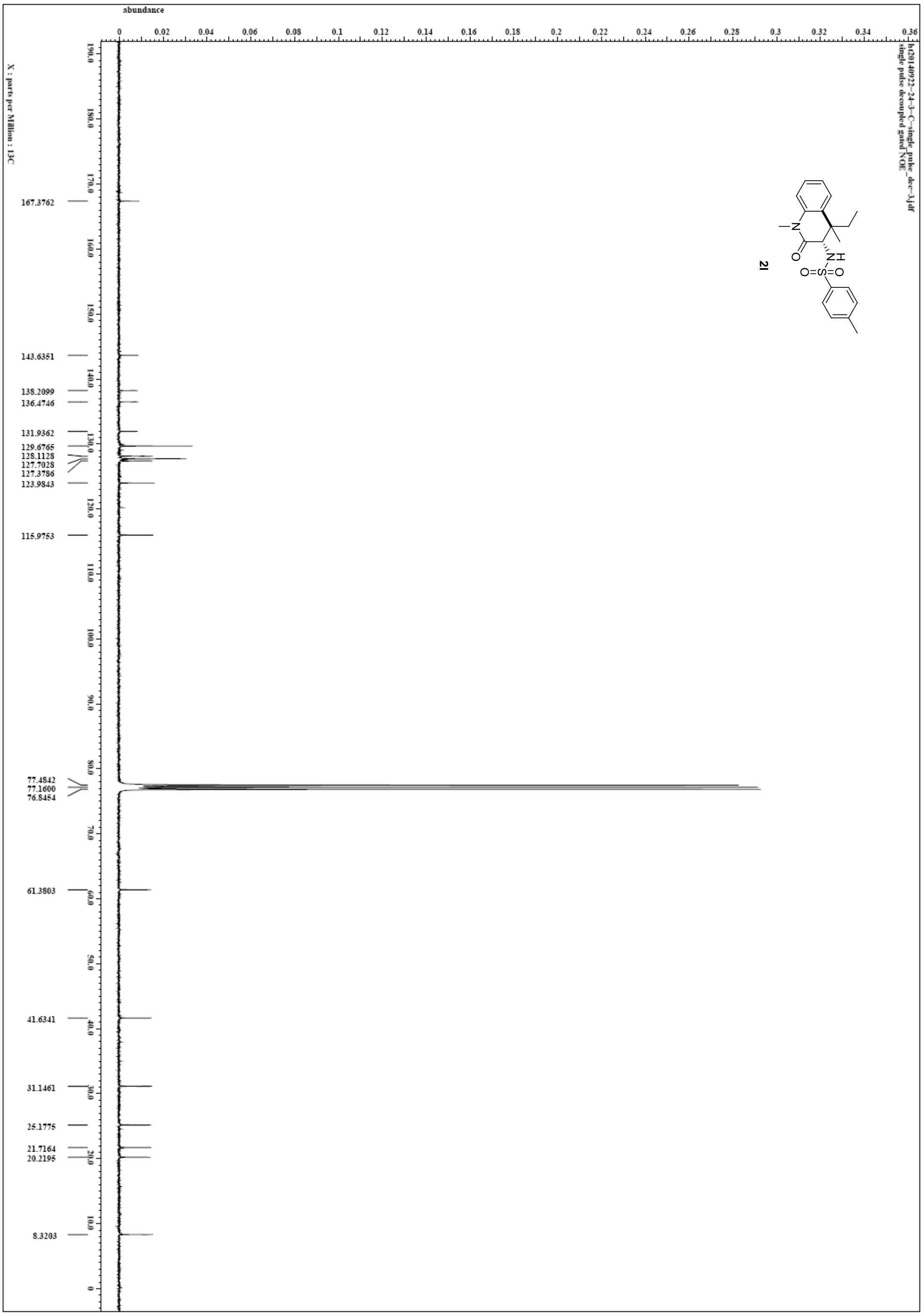
abundance
X : parts per Million : 13C
167.3762
143.6351
138.2099
136.4746
131.9362
129.6765
128.1128
127.7028
127.3786
123.9843
115.9753
77.4842
77.1600
76.8454
61.3803
41.6341
31.1461
25.1775
21.7164
20.2195
8.3203
single pulse decoupled gated NOE
2l

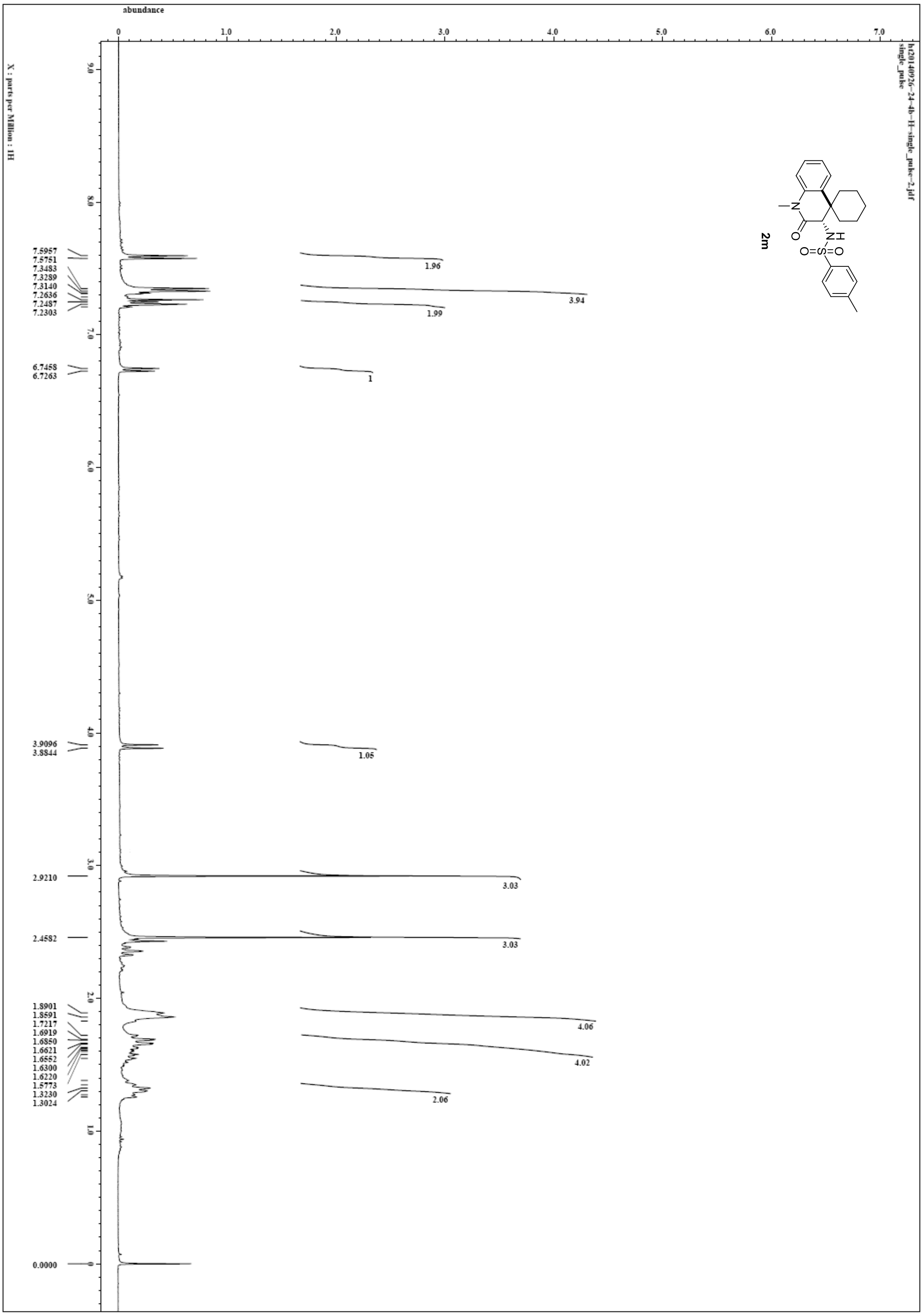
abundance
X : parts per Million : 1H
2m
7.5957
7.5751
7.3483
7.3289
7.3140
7.2636
7.2487
7.2303
6.7458
6.7263
3.9096
3.8844
2.9210
2.4582
1.8901
1.8591
1.7217
1.6919
1.6850
1.6621
1.6552
1.6300
1.6220
1.5773
1.3230
1.3024
0.0000
1.96
3.94
1.99
1
1.05
3.03
3.03
4.06
4.02
2.06

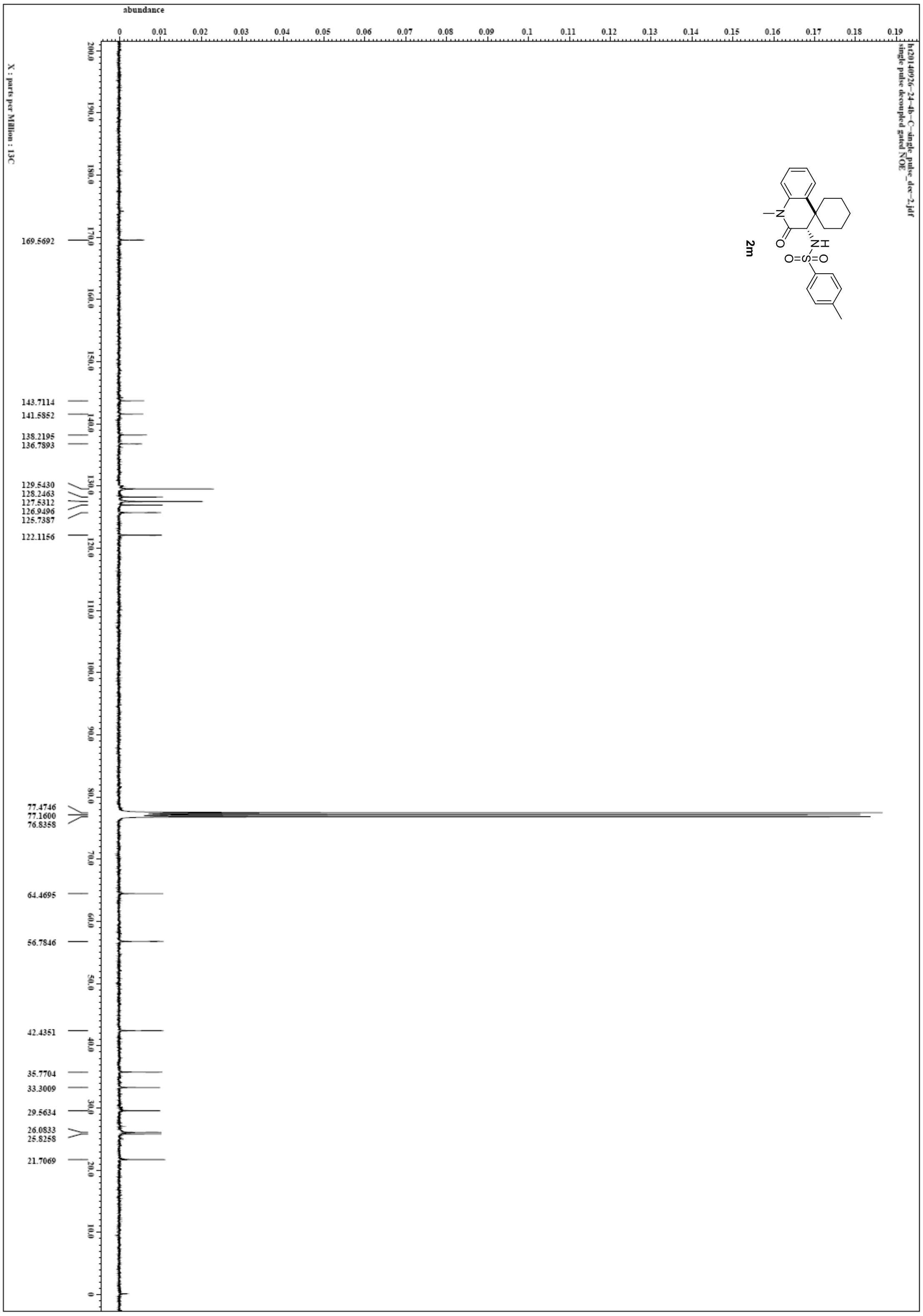
abundance
X : parts per Million : 13C
2m
169.5692
143.7114
141.5852
138.2195
136.7893
129.5430
128.2463
127.5312
126.9496
125.7387
122.1156
77.4746
77.1600
76.8358
64.4695
56.7846
42.4351
35.7704
33.3009
29.5634
26.0833
25.8258
21.7069

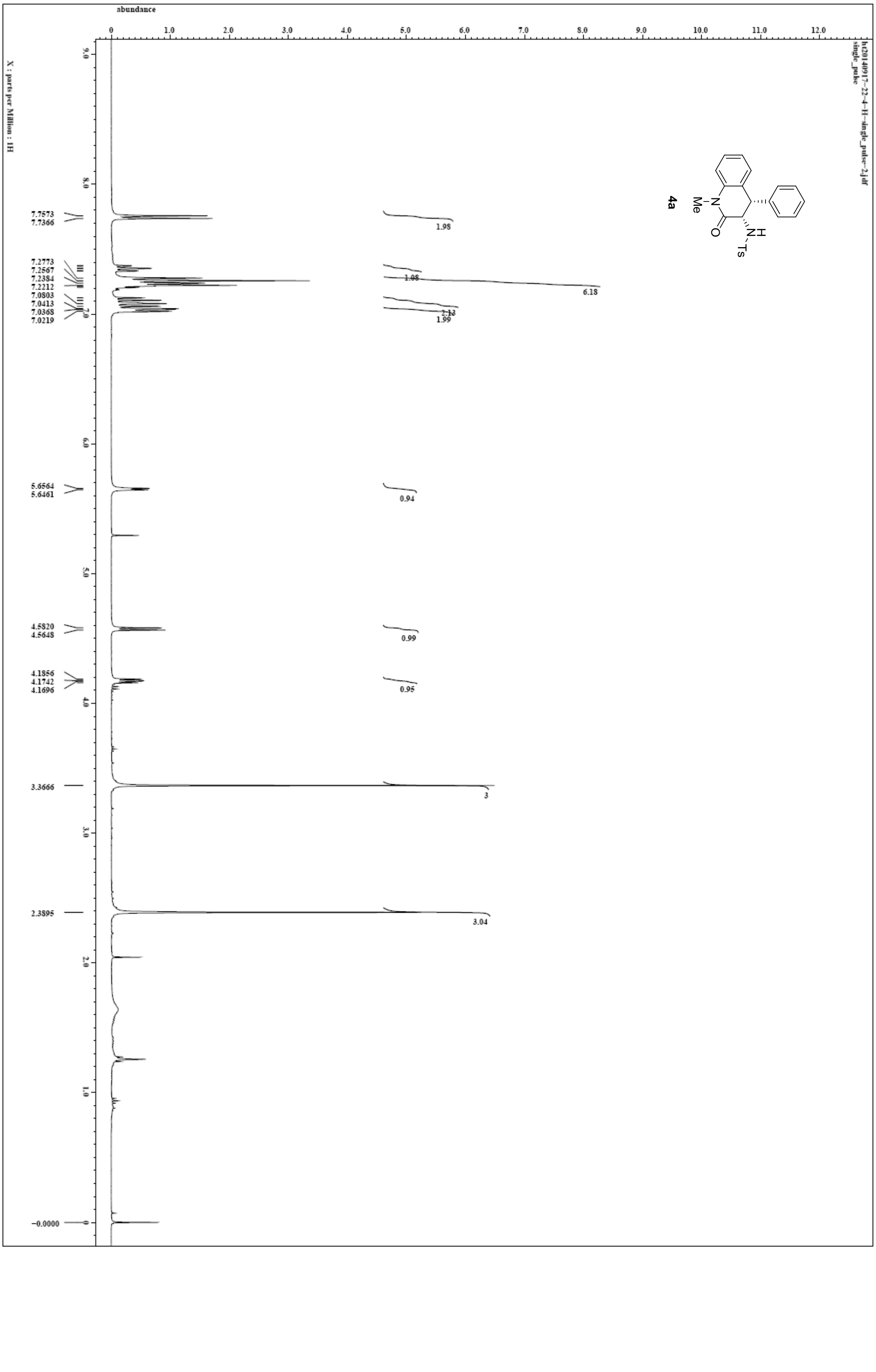
abundance
X : parts per Million : 1H
4a
Me
N
O
H
N
Ts
7.7573
7.7366
7.2773
7.2567
7.2384
7.2212
7.0803
7.0413
7.0368
7.0219
5.6564
5.6461
4.5820
4.5648
4.1856
4.1742
4.1696
3.3666
2.3895
-0.0000
1.98
1.08
6.18
2.13
1.99
0.94
0.99
0.95
3
3.04

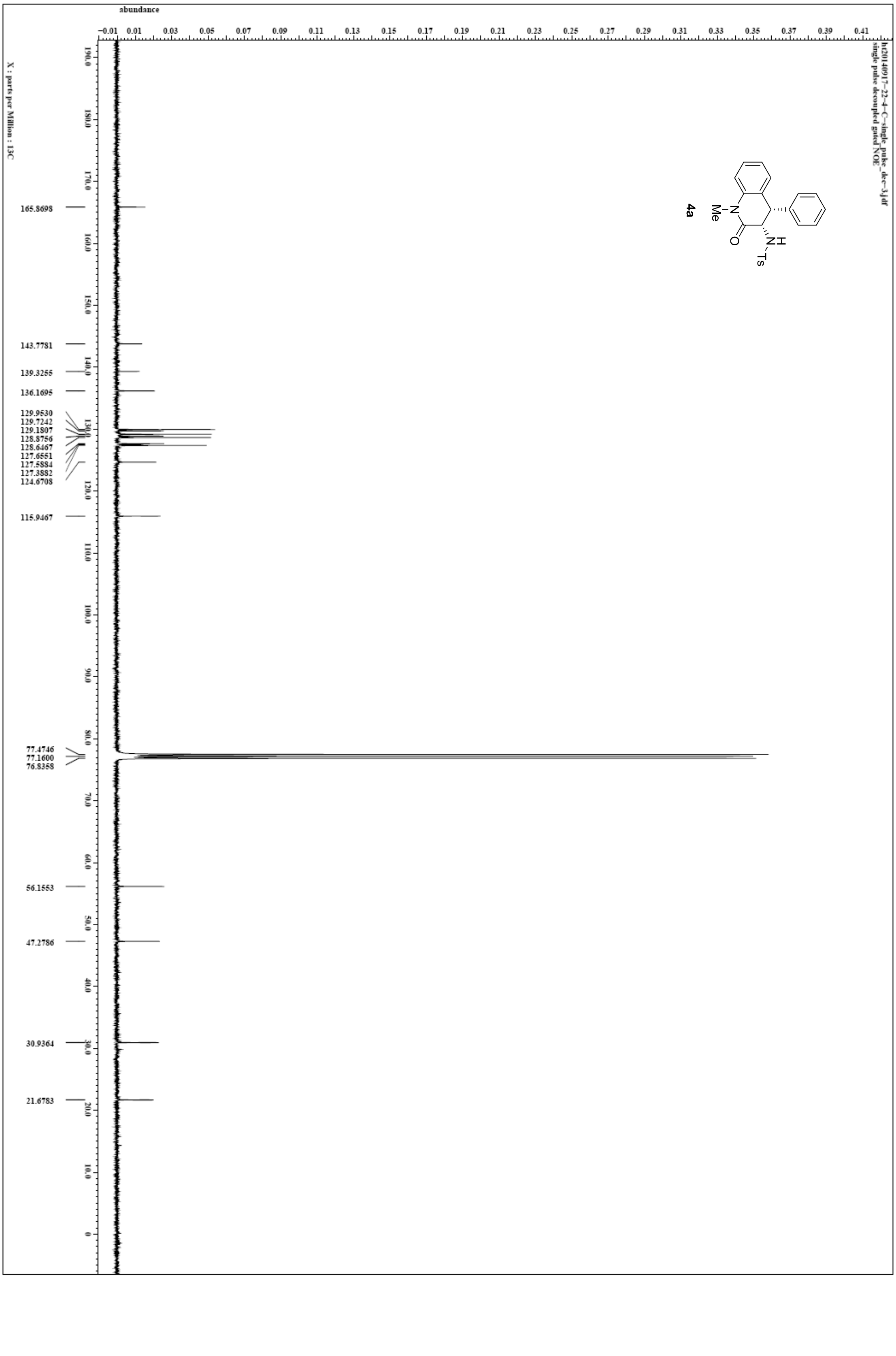
4a
N
Me
O
H
N
Ts
abundance
X : parts per Million : 13C
165.8698
143.7781
139.3255
136.1695
129.9530
129.7242
129.1807
128.8756
128.6467
127.6551
127.5884
127.3882
124.6708
115.9467
77.4746
77.1600
76.8358
56.1553
47.2786
30.9364
21.6783

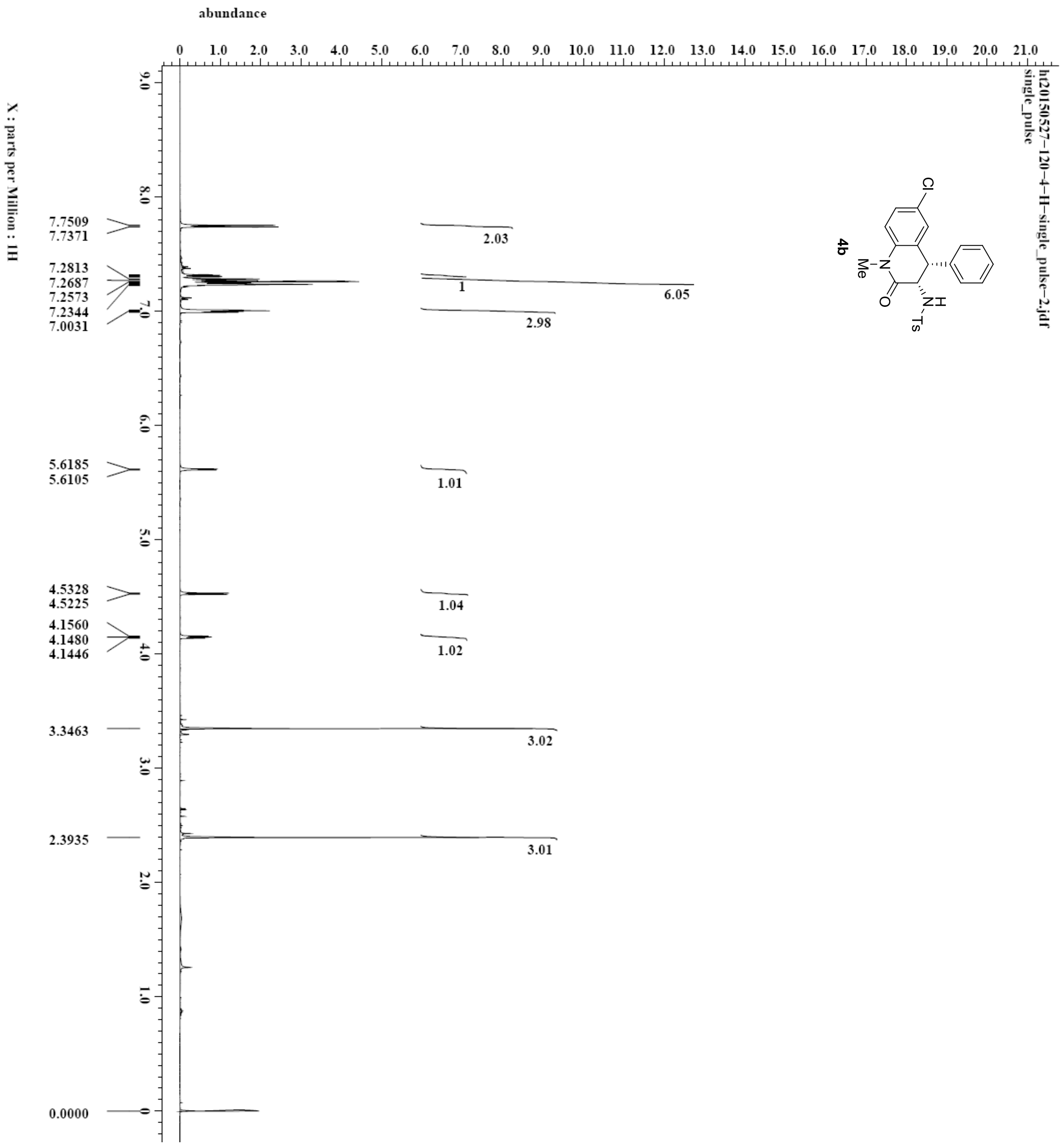
h20150527-120-4-H-single_pulse-2.jdf
single_pulse
abundance
X : parts per Million : 1H
7.7509
7.7371
7.2813
7.2687
7.2573
7.2344
7.0031
5.6185
5.6105
4.5328
4.5225
4.1560
4.1480
4.1446
3.3463
2.3935
0.0000
2.03
1
6.05
2.98
1.01
1.04
1.02
3.02
3.01
4b
Cl
N
Me
O
H
N
Ts

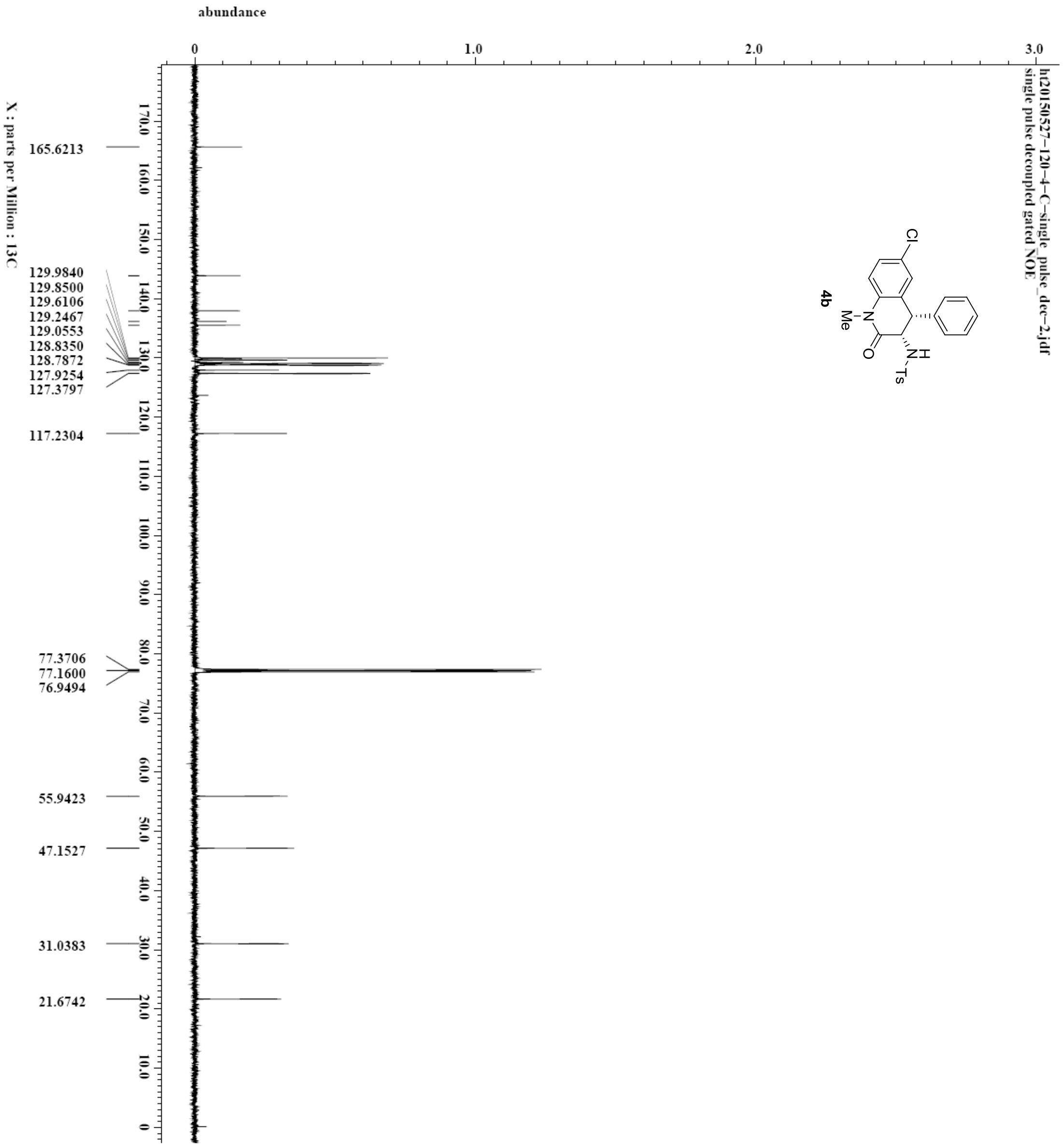
abundance
0
1.0
2.0
3.0
h20150527-120-4-C-single_pulse_dec-2.jdf
single pulse decoupled gated NOE
4b
Cl
N
Me
O
H
N
Ts
165.6213
129.9840
129.8500
129.6106
129.2467
129.0553
128.8350
128.7872
127.9254
127.3797
117.2304
77.3706
77.1600
76.9494
55.9423
47.1527
31.0383
21.6742
X : parts per Million : 13C

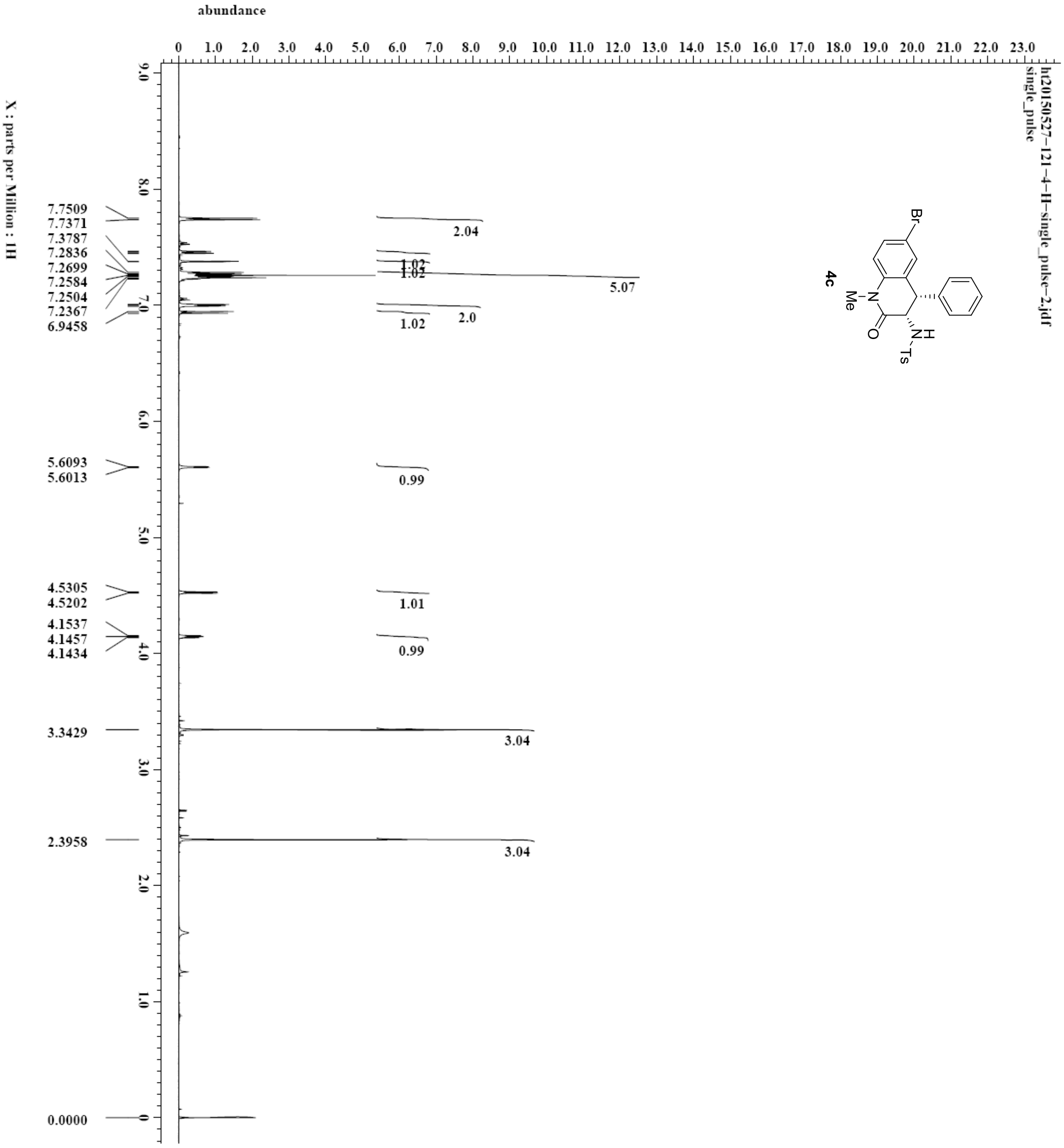
abundance
X : parts per Million : 1H
ht20150527--121--4--H--single_pulse-2.jdf
single_pulse
4c
Br
N
Me
O
H
N
Ts
7.7509
7.7371
7.3787
7.2836
7.2699
7.2584
7.2504
7.2367
6.9458
5.6093
5.6013
4.5305
4.5202
4.1537
4.1457
4.1434
3.3429
2.3958
0.0000
2.04
1.02
1.02
5.07
2.0
1.02
0.99
1.01
0.99
3.04
3.04

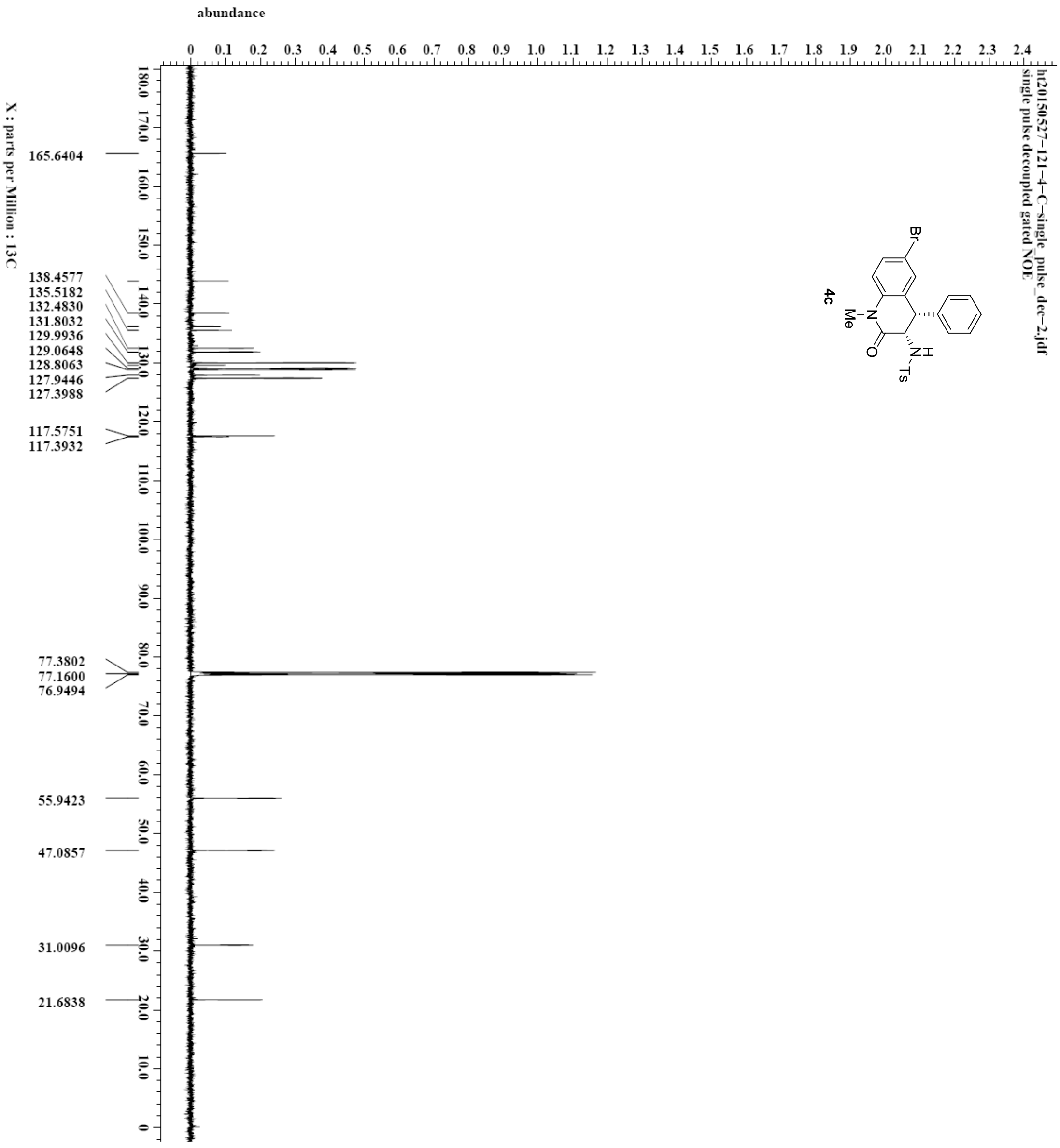
abundance
X : parts per Million : 13C
h20150527-121-4-C~single_pulse_dec~2.jdf
single pulse decoupled gated NOE
165.6404
138.4577
135.5182
132.4830
131.8032
129.9936
129.0648
128.8063
127.9446
127.3988
117.5751
117.3932
77.3802
77.1600
76.9494
55.9423
47.0857
31.0096
21.6838
4c
Br
N
Me
O
H
N
Ts

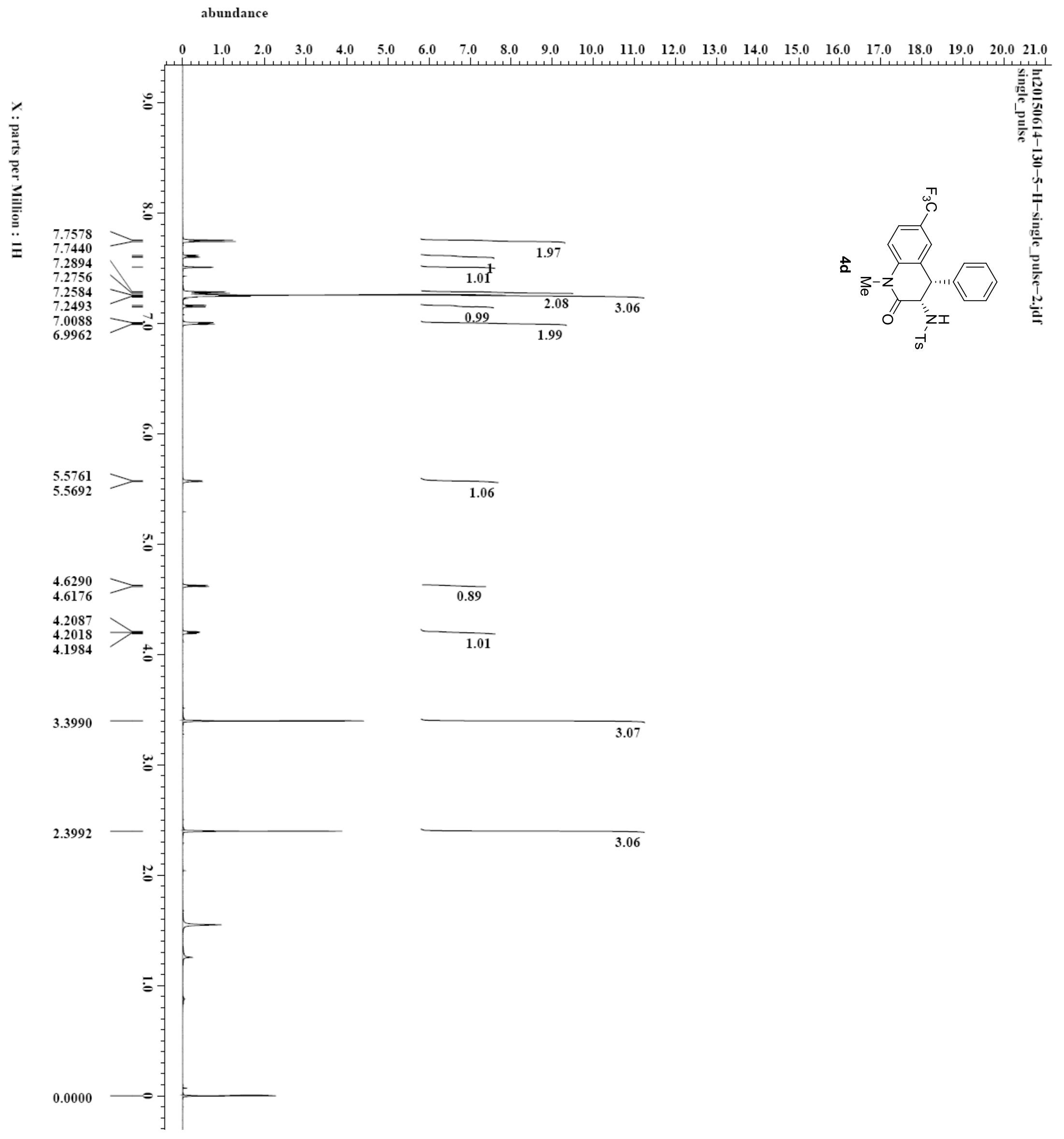
abundance
X : parts per Million : 1H
ht20150614-130-5-H-single_pulse-2.jdf
single_pulse
4d
F3C
N
Me
O
H
N
Ts
7.7578
7.7440
7.2894
7.2756
7.2584
7.2493
7.0088
6.9962
5.5761
5.5692
4.6290
4.6176
4.2087
4.2018
4.1984
3.3990
2.3992
0.0000
1.97
1.01
2.08
3.06
0.99
1.99
1.06
0.89
1.01
3.07
3.06

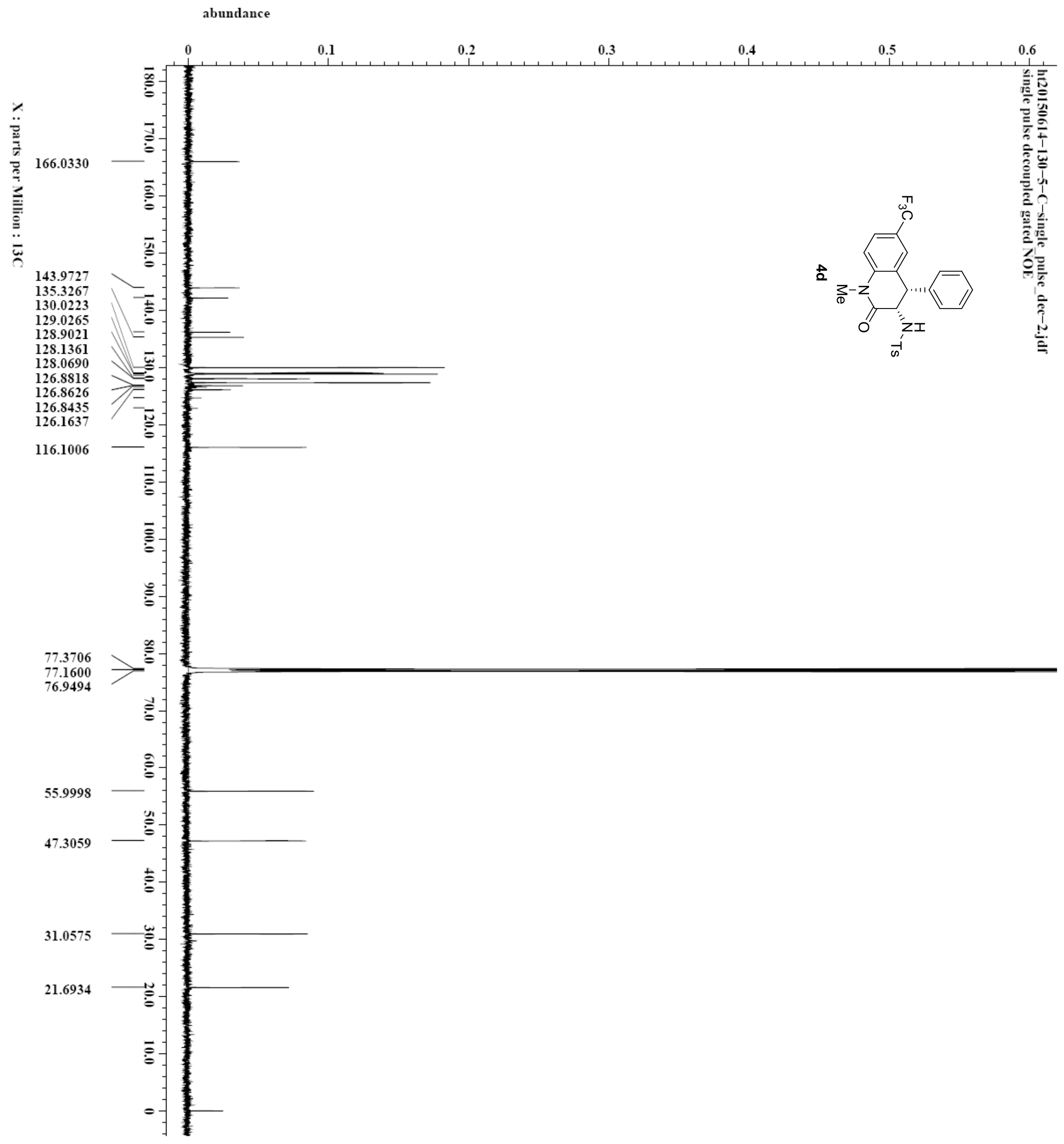
abundance
X : parts per Million : 13C
h20150614-130-5-C-single_pulse_dec-2.jdf
single pulse decoupled gated NOE
166.0330
143.9727
135.3267
130.0223
129.0265
128.9021
128.1361
128.0690
126.8818
126.8626
126.8435
126.1637
116.1006
77.3706
77.1600
76.9494
55.9998
47.3059
31.0575
21.6934
4d

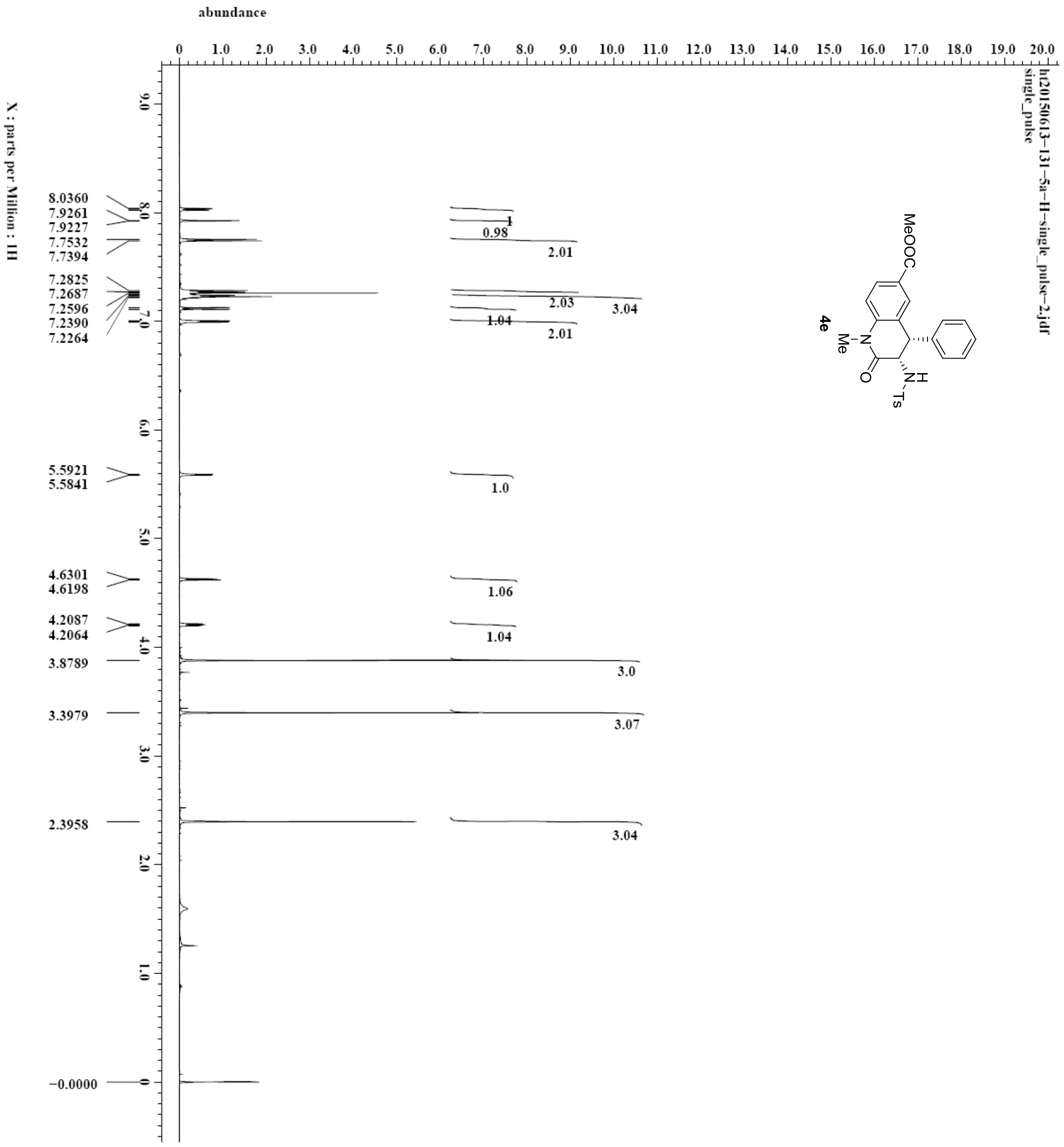
abundance
X : parts per Million : 1H
ht20150613-131-5a-H-single_pulse-2.jdf
single_pulse
8.0360
7.9261
7.9227
7.7532
7.7394
7.2825
7.2687
7.2596
7.2390
7.2264
5.5921
5.5841
4.6301
4.6198
4.2087
4.2064
3.8789
3.3979
2.3958
-0.0000
MeOOC
4e

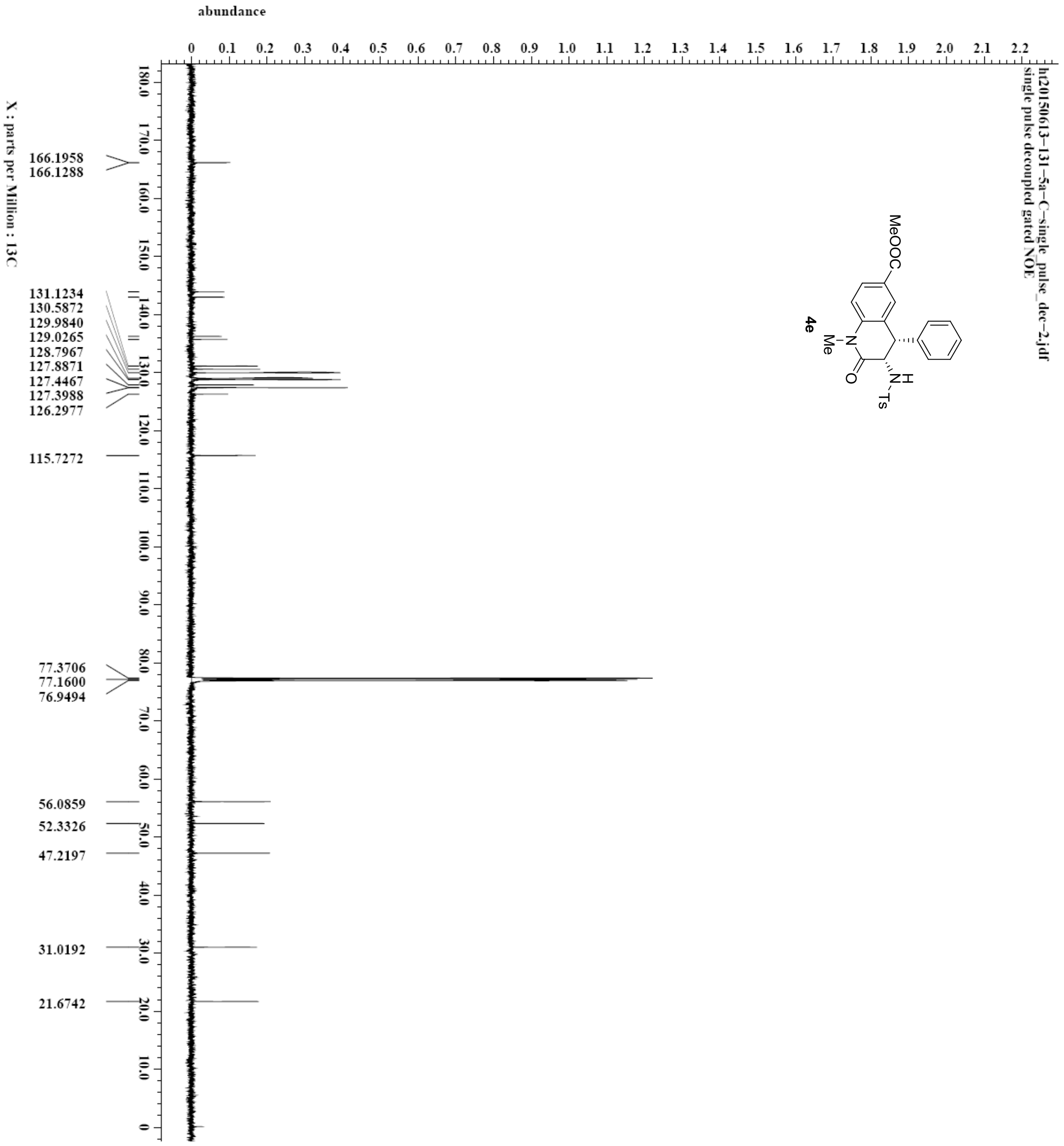
abundance
ht20150613-131-5a-C-single_pulse_dec-2.jdf
single pulse decoupled gated NOE
X : parts per Million : 13C
166.1958
166.1288
131.1234
130.5872
129.9840
129.0265
128.7967
127.8871
127.4467
127.3988
126.2977
115.7272
77.3706
77.1600
76.9494
56.0859
52.3326
47.2197
31.0192
21.6742
MeOOC
N
Me
O
H
N
Ts
4e

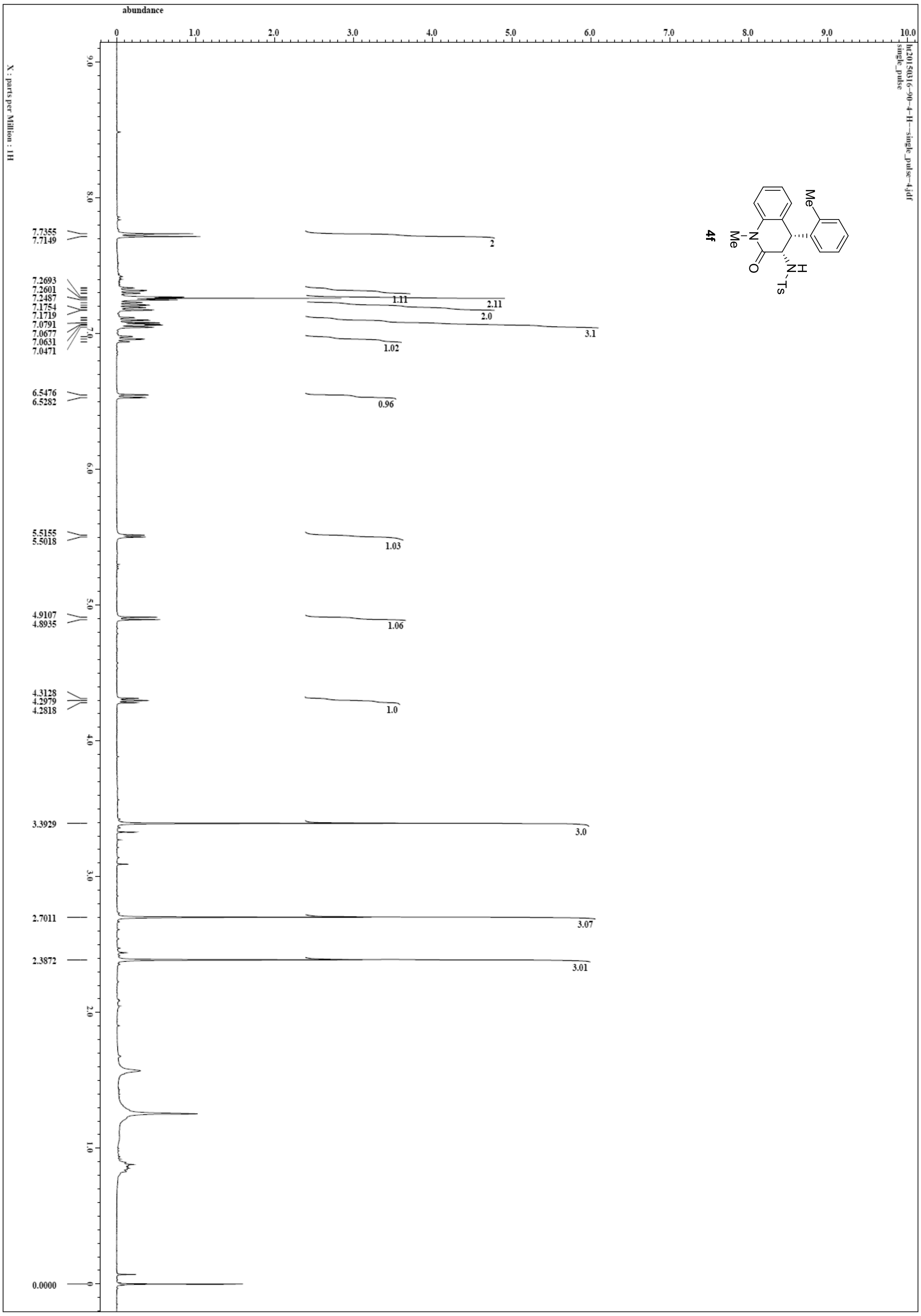
abundance
X : parts per Million : 1H
4f
7.7355
7.7149
7.2693
7.2601
7.2487
7.1754
7.1719
7.0791
7.0677
7.0631
7.0471
6.5476
6.5282
5.5155
5.5018
4.9107
4.8935
4.3128
4.2979
4.2818
3.3929
2.7011
2.3872
0.0000
2
1.11
2.11
2.0
3.1
1.02
0.96
1.03
1.06
1.0
3.0
3.07
3.01

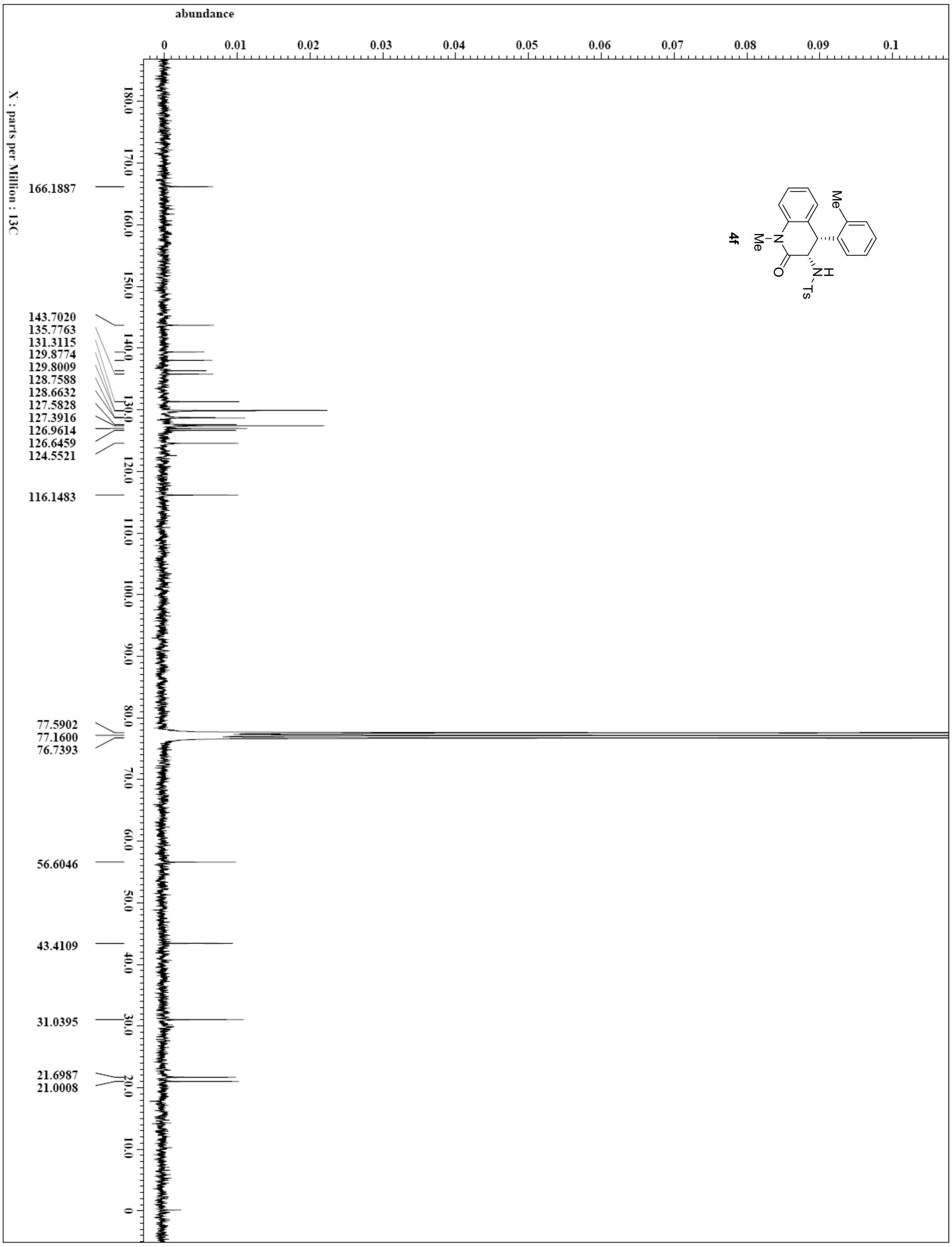
abundance
X : parts per Million : 13C
166.1887
143.7020
135.7763
131.3115
129.8774
129.8009
128.7588
128.6632
127.5828
127.3916
126.9614
126.6459
124.5521
116.1483
77.5902
77.1600
76.7393
56.6046
43.4109
31.0395
21.6987
21.0008
Me
N
Me
O
H
N
Ts
4f

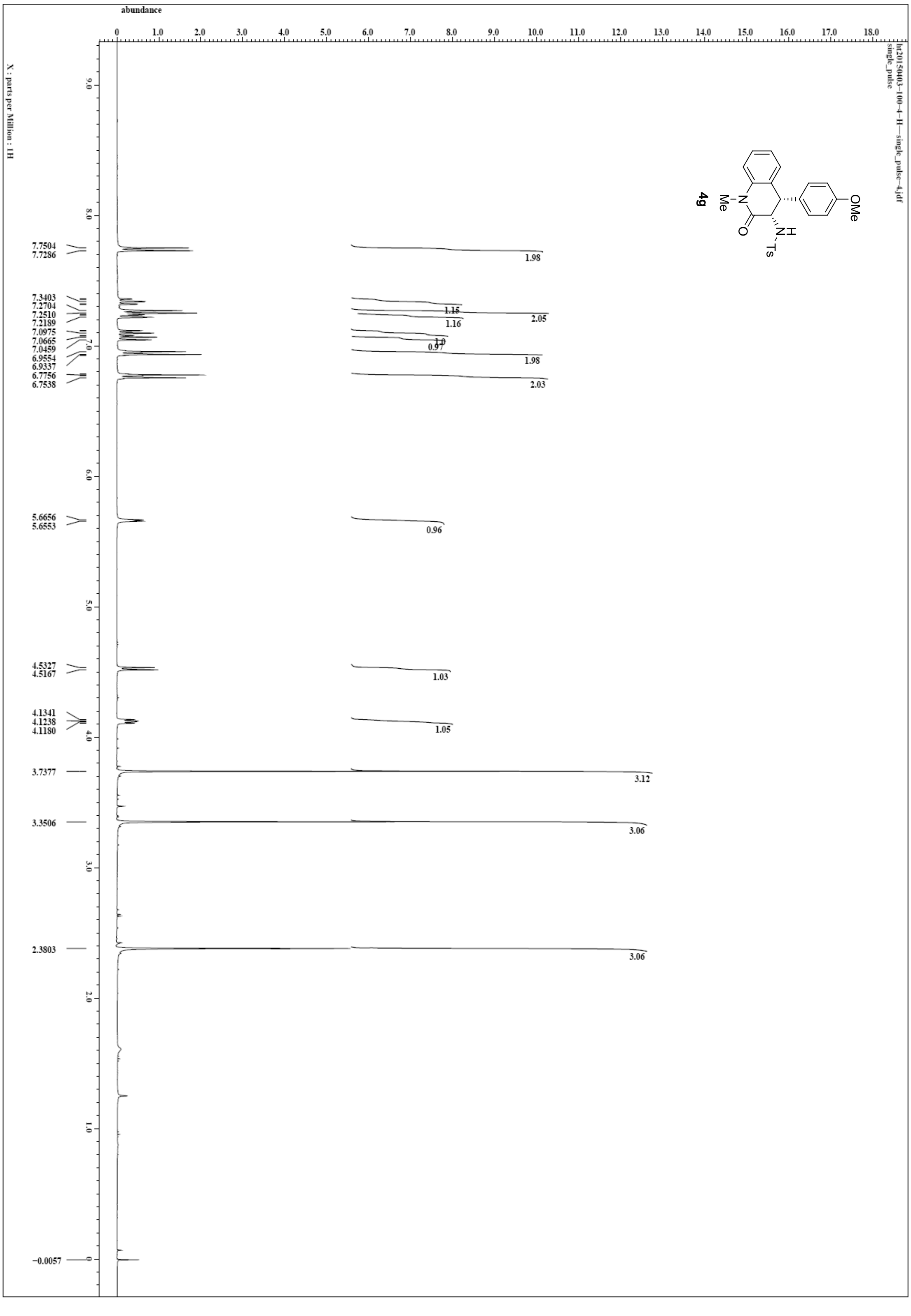
abundance
X : parts per Million : 1H
7.7504
7.7286
7.3403
7.2704
7.2510
7.2189
7.0975
7.0665
7.0459
6.9554
6.9337
6.7756
6.7538
5.6656
5.6553
4.5327
4.5167
4.1341
4.1238
4.1180
3.7377
3.3506
2.3803
−0.0057
1.98
1.15
2.05
1.16
0.97
1.98
2.03
0.96
1.03
1.05
3.12
3.06
3.06
4g
OMe
Me
Ts

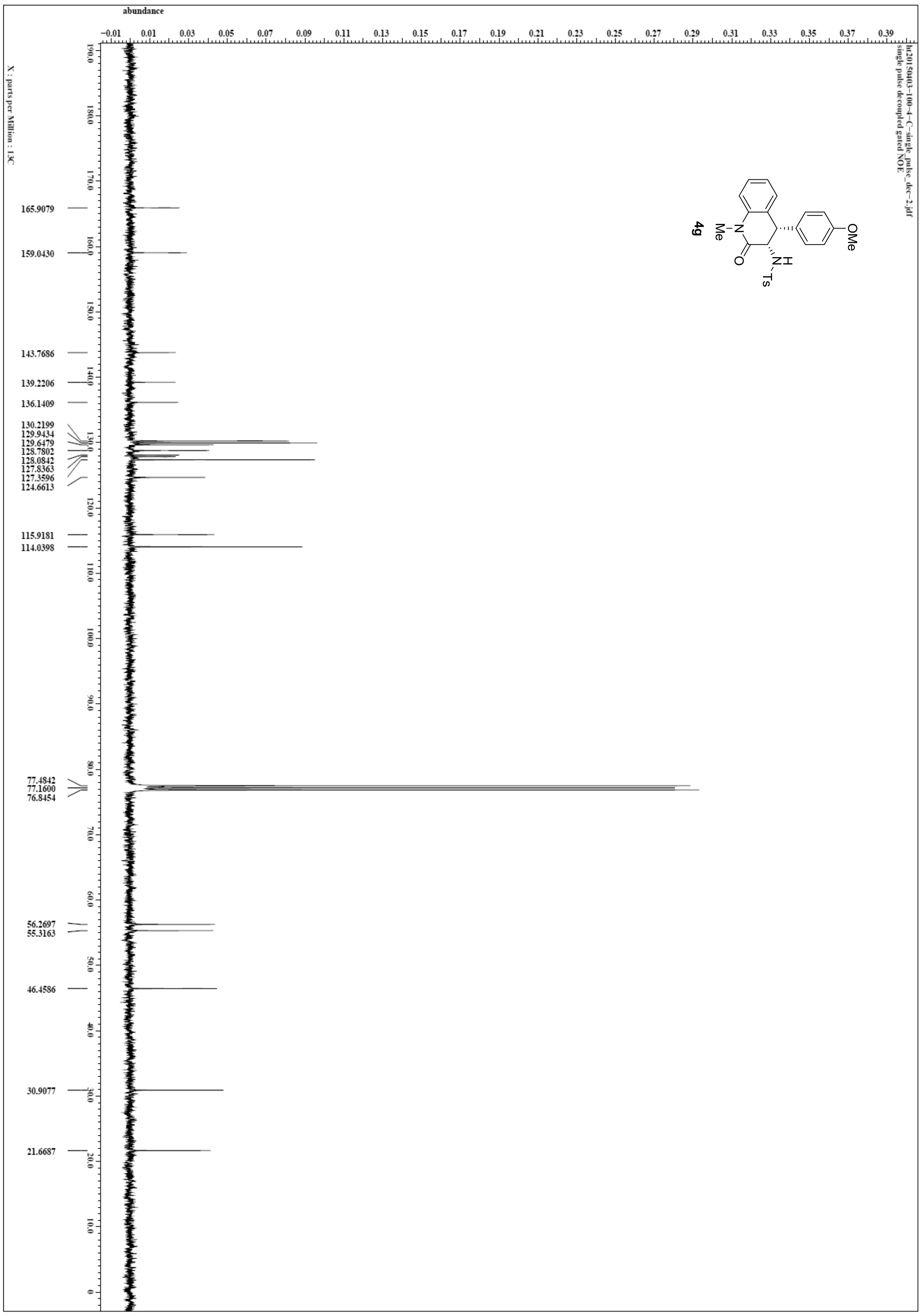
165.9079
159.0430
143.7686
139.2206
136.1409
130.2199
129.9434
129.6479
128.7802
128.0842
127.8363
127.3596
124.6613
115.9181
114.0398
77.4842
77.1600
76.8454
56.2697
55.3163
46.4586
30.9077
21.6687
4g

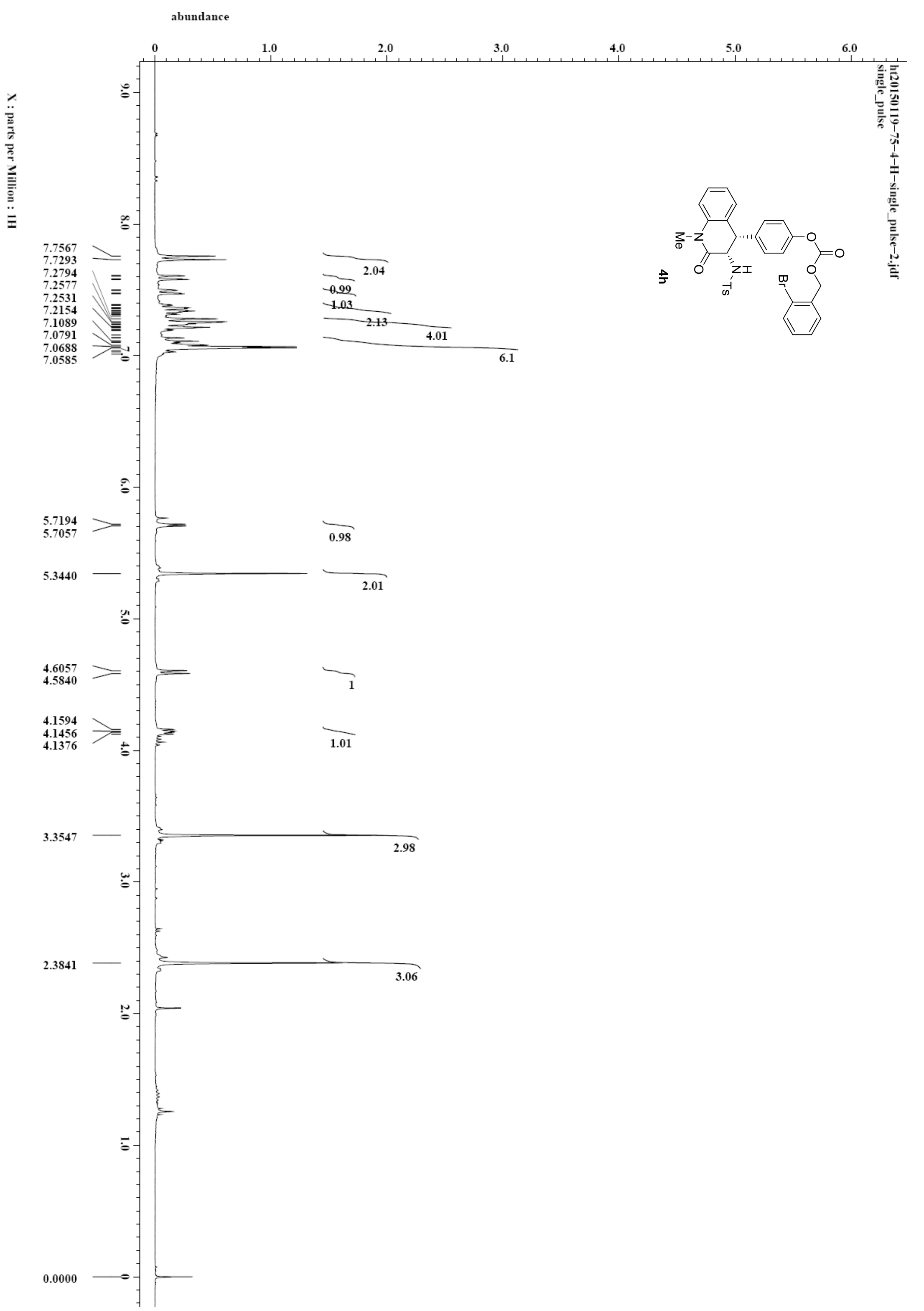
abundance
X : parts per Million : 1H
ht20150119-75-4-H-single_pulse-2.jdf
single_pulse
4h
7.7567
7.7293
7.2794
7.2577
7.2531
7.2154
7.1089
7.0791
7.0688
7.0585
5.7194
5.7057
5.3440
4.6057
4.5840
4.1594
4.1456
4.1376
3.3547
2.3841
0.0000
2.04
0.99
1.03
2.13
4.01
6.1
0.98
2.01
1
1.01
2.98
3.06

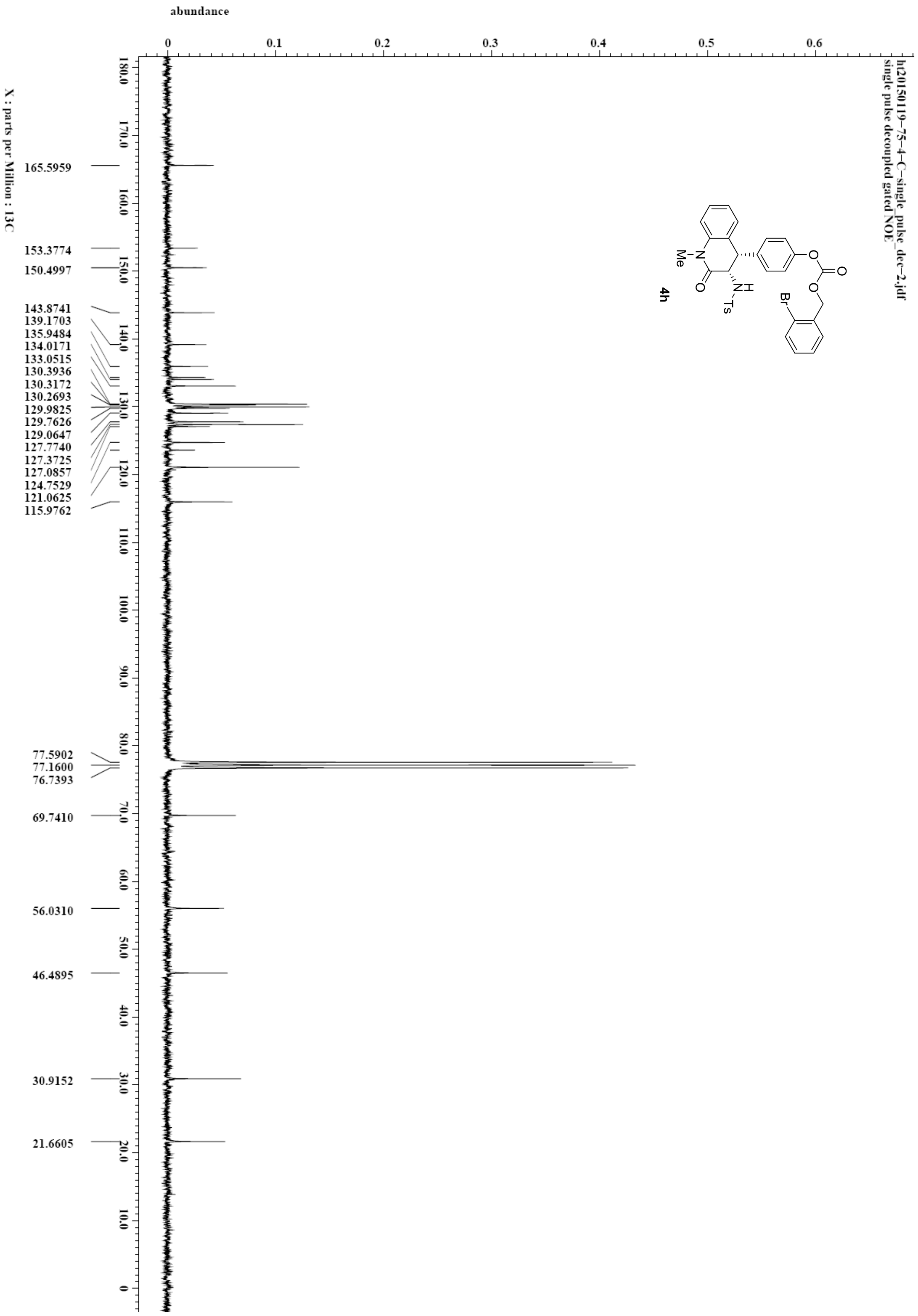
abundance
0
0.1
0.2
0.3
0.4
0.5
0.6
X : parts per Million : 13C
hl20150119-75-4-C-single_pulse_dec-2.jdf
single pulse decoupled gated NOE
165.5959
153.3774
150.4997
143.8741
139.1703
135.9484
134.0171
133.0515
130.3936
130.3172
130.2693
129.9825
129.7626
129.0647
127.7740
127.3725
127.0857
124.7529
121.0625
115.9762
77.5902
77.1600
76.7393
69.7410
56.0310
46.4895
30.9152
21.6605
4h

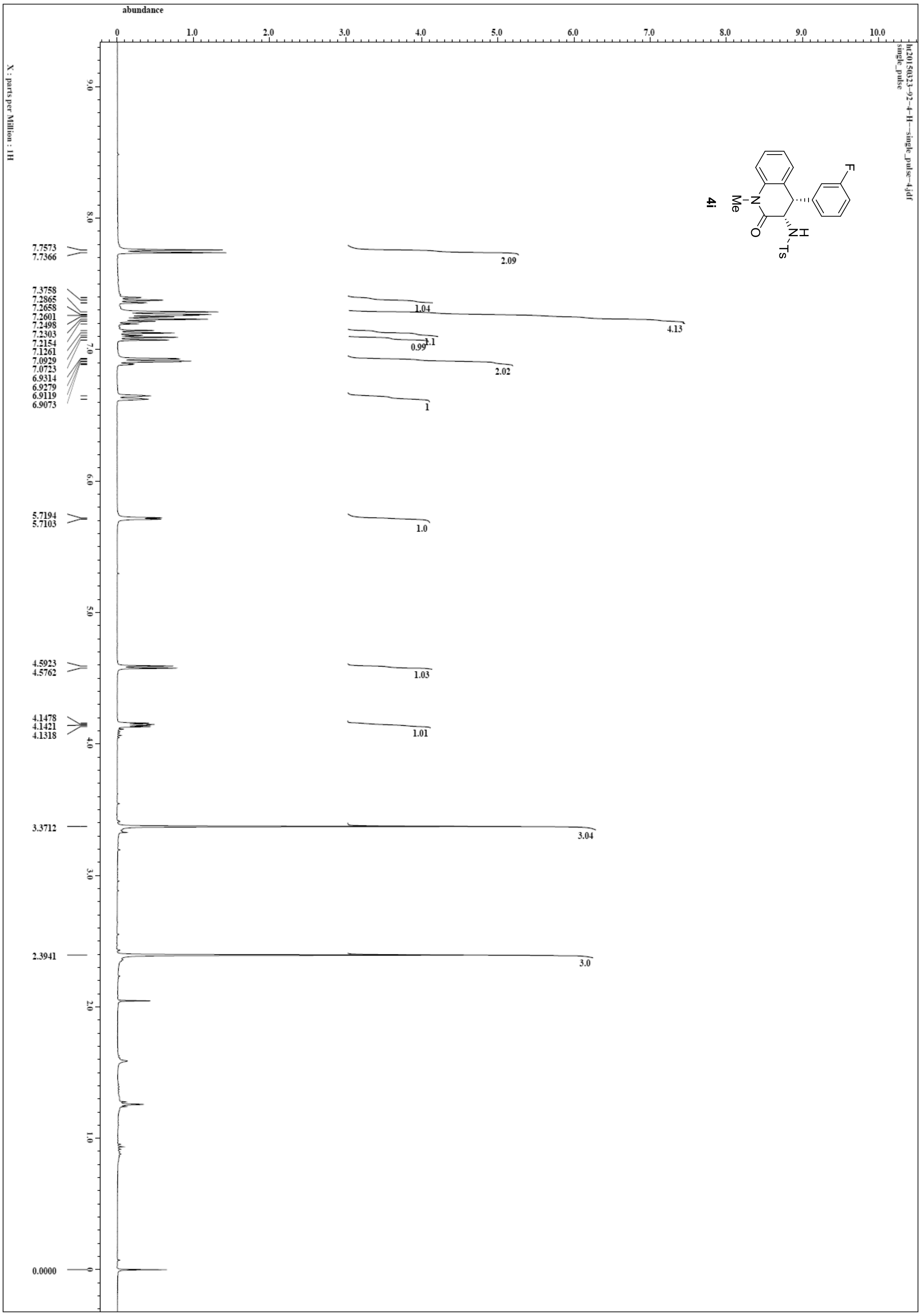
abundance
X : parts per Million : 1H
4i
7.7573
7.7366
7.3758
7.2865
7.2658
7.2601
7.2498
7.2303
7.2154
7.1261
7.0929
7.0723
6.9314
6.9279
6.9119
6.9073
5.7194
5.7103
4.5923
4.5762
4.1478
4.1421
4.1318
3.3712
2.3941
0.0000
2.09
1.04
4.13
1.1
0.99
2.02
1
1.0
1.03
1.01
3.04
3.0

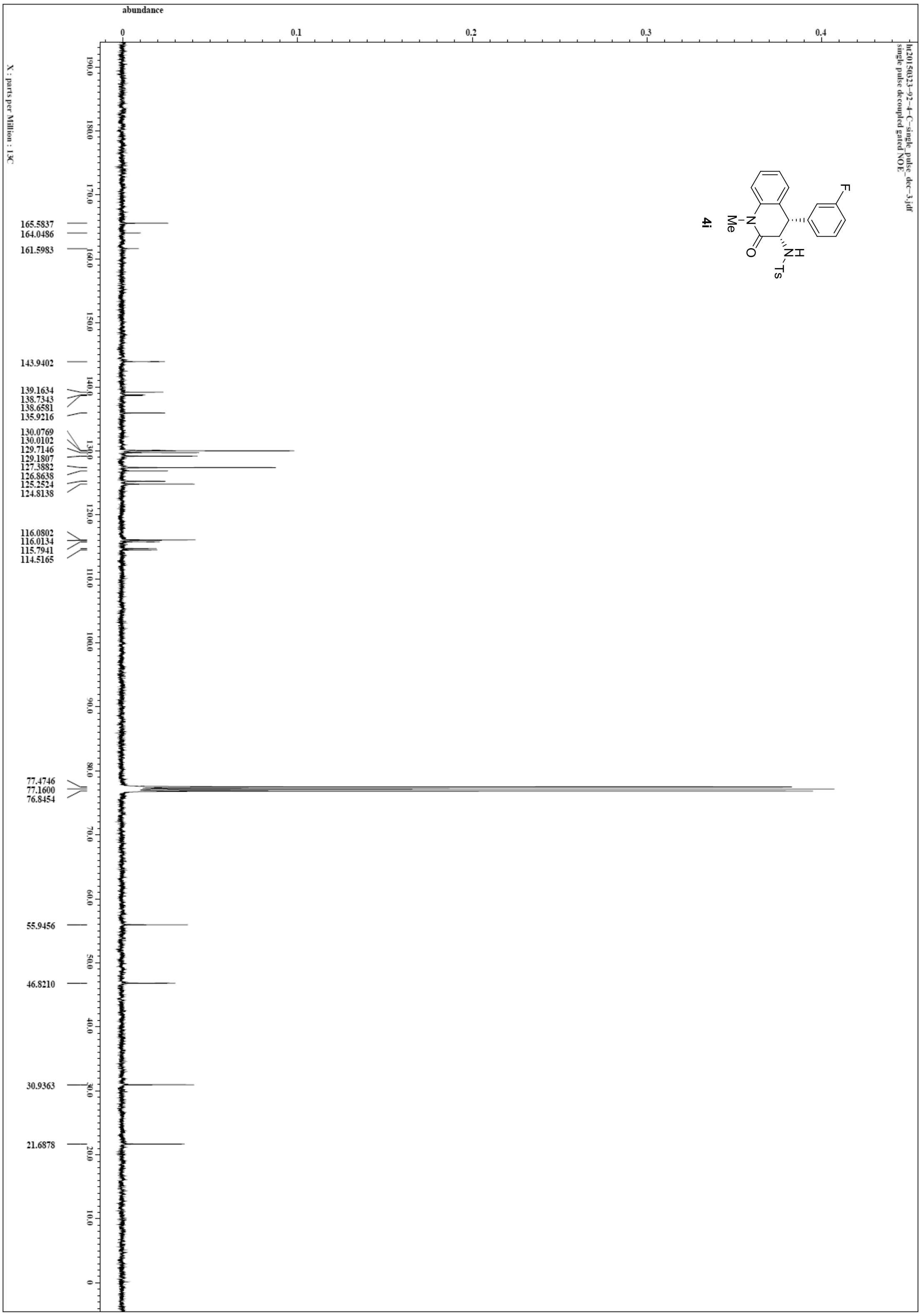
abundance
X : parts per Million : 13C
hl20150323-92-4-C-single_pulse_dec-3.jdf
single pulse decoupled gated NOE
4i
165.5837
164.0486
161.5983
143.9402
139.1634
138.7343
138.6581
135.9216
130.0769
130.0102
129.7146
129.1807
127.3882
126.8638
125.2524
124.8138
116.0802
116.0134
115.7941
114.5165
77.4746
77.1600
76.8454
55.9456
46.8210
30.9363
21.6878

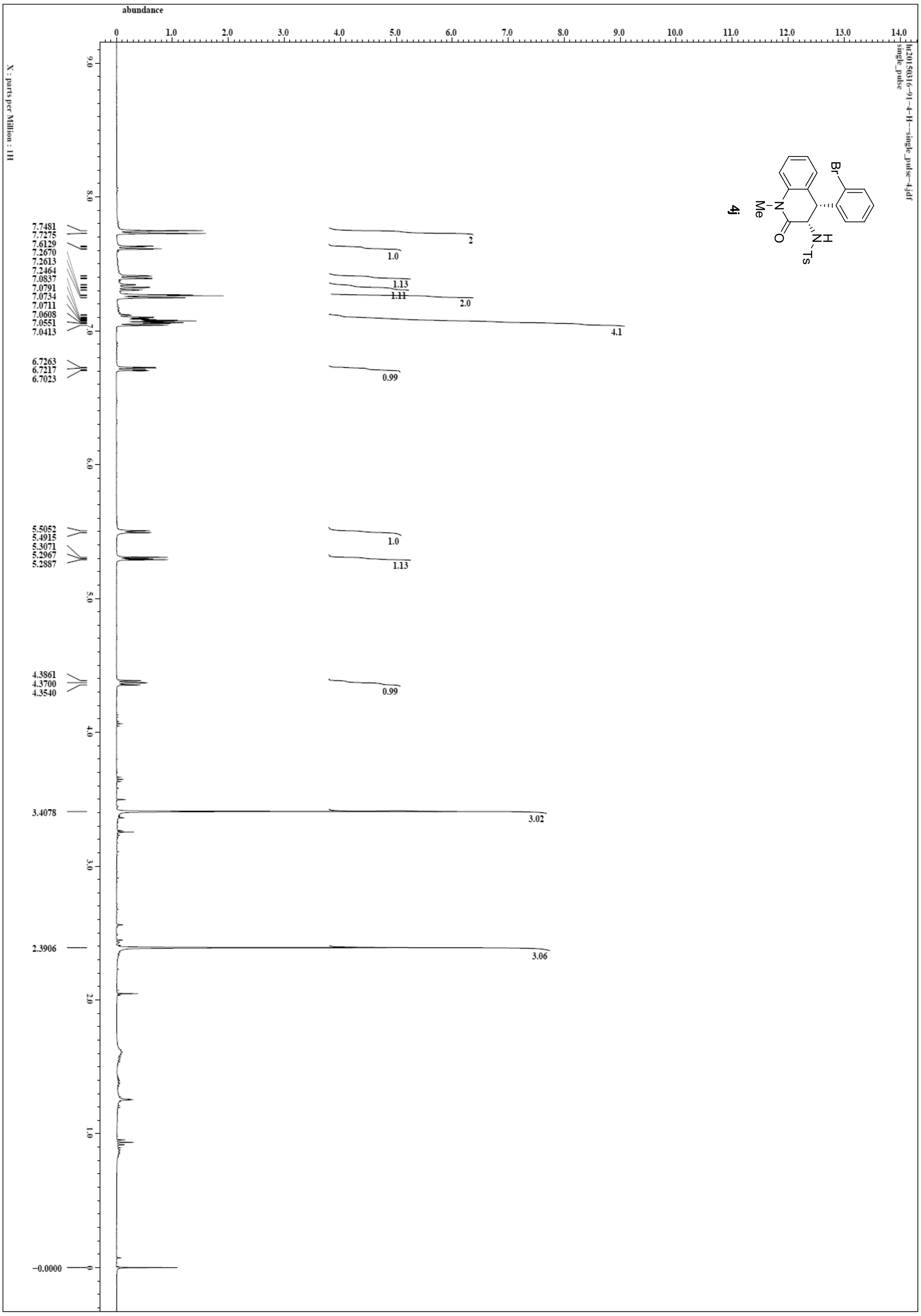
abundance
X : parts per Million : 1H
hc20150116-91--4-H---single_pulse-4j.jdf
single_pulse
4j
Br
N
Me
O
H
N
Ts
7.7481
7.7275
7.6129
7.2670
7.2613
7.2464
7.0837
7.0791
7.0734
7.0711
7.0608
7.0551
7.0413
6.7263
6.7217
6.7023
5.5052
5.4915
5.3071
5.2967
5.2887
4.3861
4.3700
4.3540
3.4078
2.3906
-0.0000
2
1.0
1.13
1.11
2.0
4.1
0.99
1.0
1.13
0.99
3.02
3.06

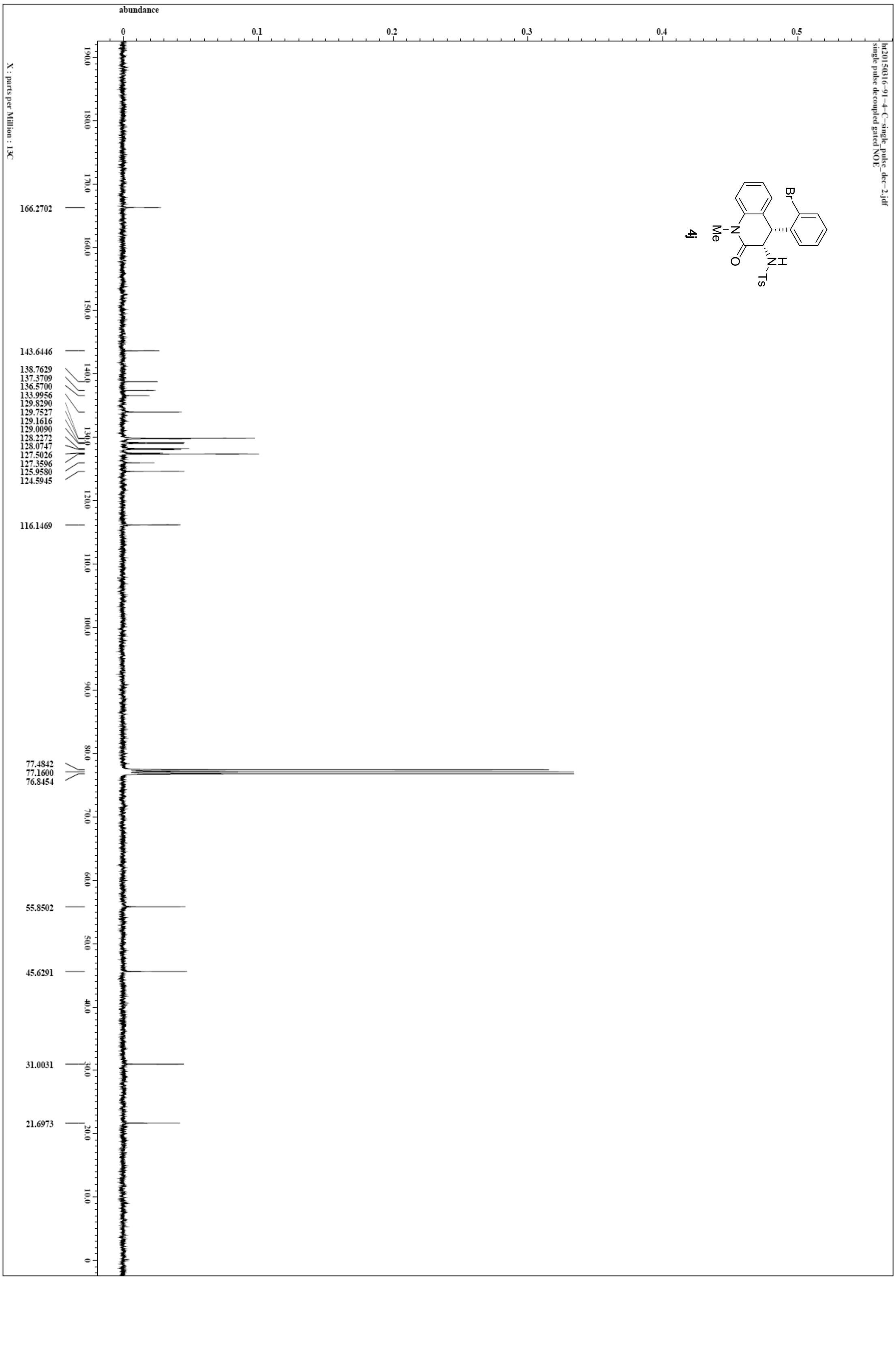
abundance
0
0.1
0.2
0.3
0.4
0.5
X : parts per Million : 13C
4j
Br
N
Me
O
H
N
Ts
166.2702
143.6446
138.7629
137.3709
136.5700
133.9956
129.8290
129.7527
129.1616
129.0090
128.2272
128.0747
127.5026
127.3596
125.9580
124.5945
116.1469
77.4842
77.1600
76.8454
55.8502
45.6291
31.0031
21.6973
single pulse decoupled gated NOE

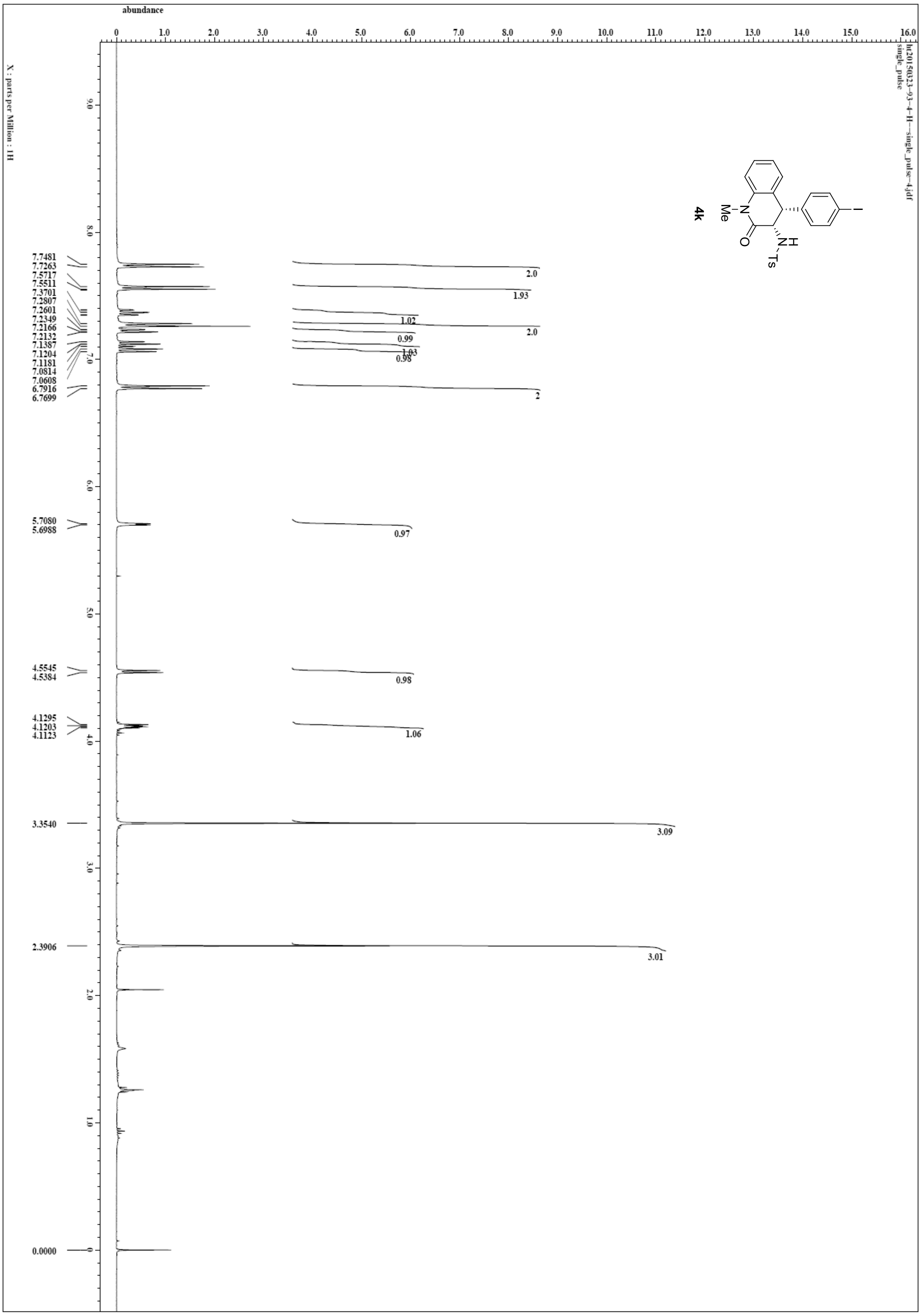

abundance
X : parts per Million : 1H
hl20150323-93-4-H---single_pulse-4.jdf
single_pulse
4k
N
Me
O
H
N
Ts
I
7.7481
7.7263
7.5717
7.5511
7.3701
7.2807
7.2601
7.2349
7.2166
7.2132
7.1387
7.1204
7.1181
7.0814
7.0608
6.7916
6.7699
5.7080
5.6988
4.5545
4.5384
4.1295
4.1203
4.1123
3.3540
2.3906
0.0000
2.0
1.93
1.02
2.0
0.99
1.03
0.98
2
0.97
0.98
1.06
3.09
3.01

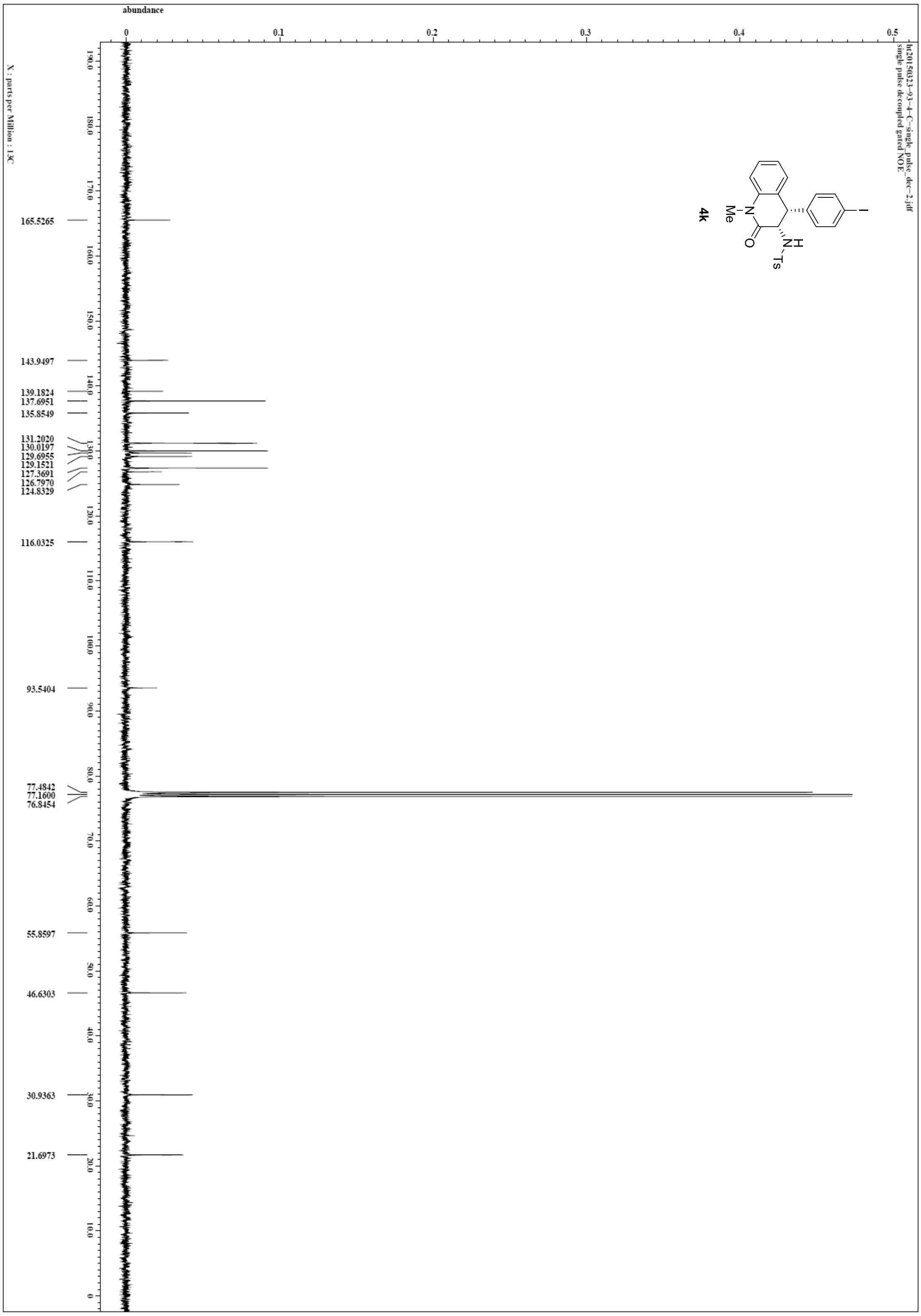

abundance
X : parts per Million : 13C
4k
165.5265
143.9497
139.1824
137.6951
135.8549
131.2020
130.0197
129.6955
129.1521
127.3691
126.7970
124.8329
116.0325
93.5404
77.4842
77.1600
76.8454
55.8597
46.6303
30.9363
21.6973

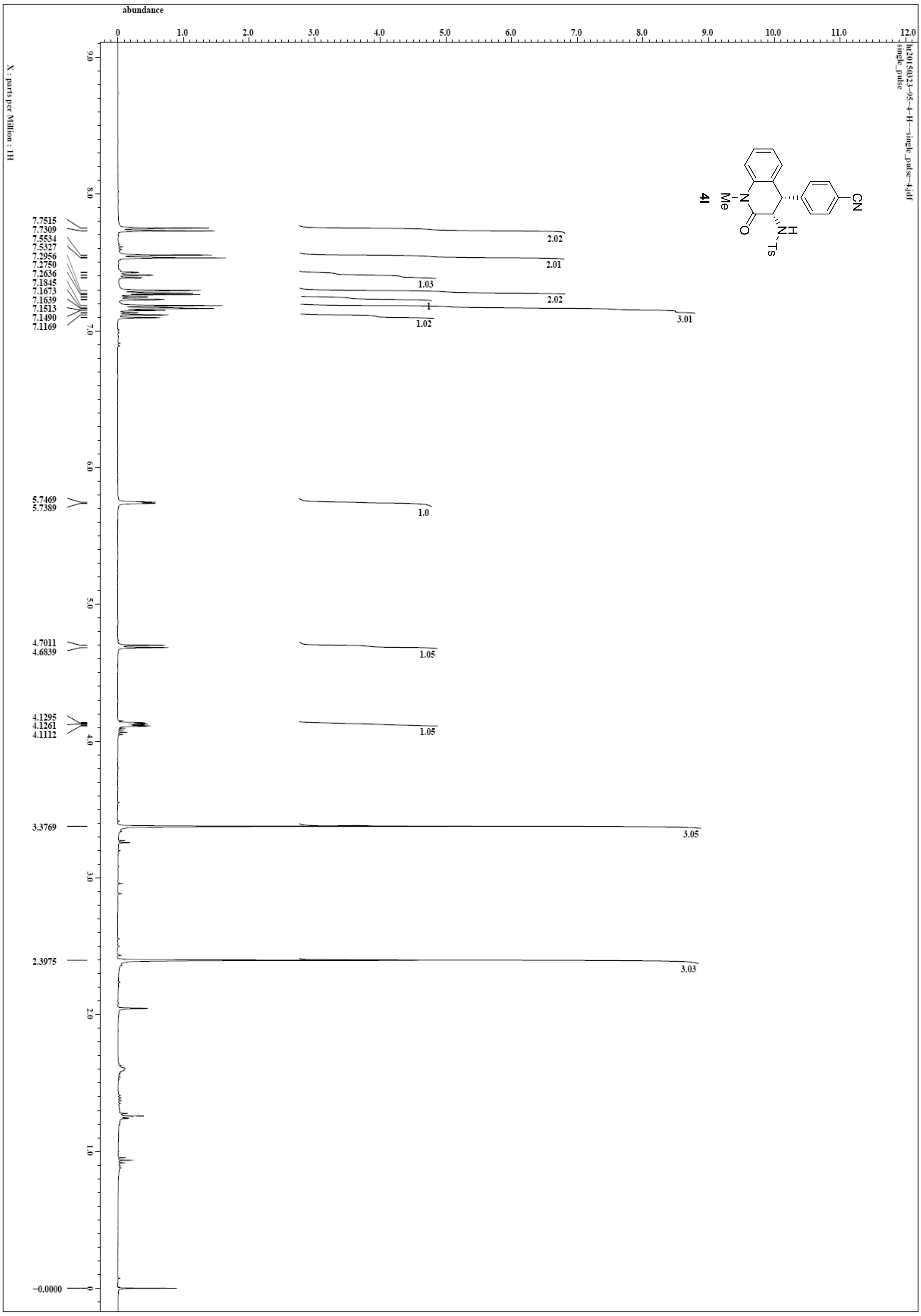

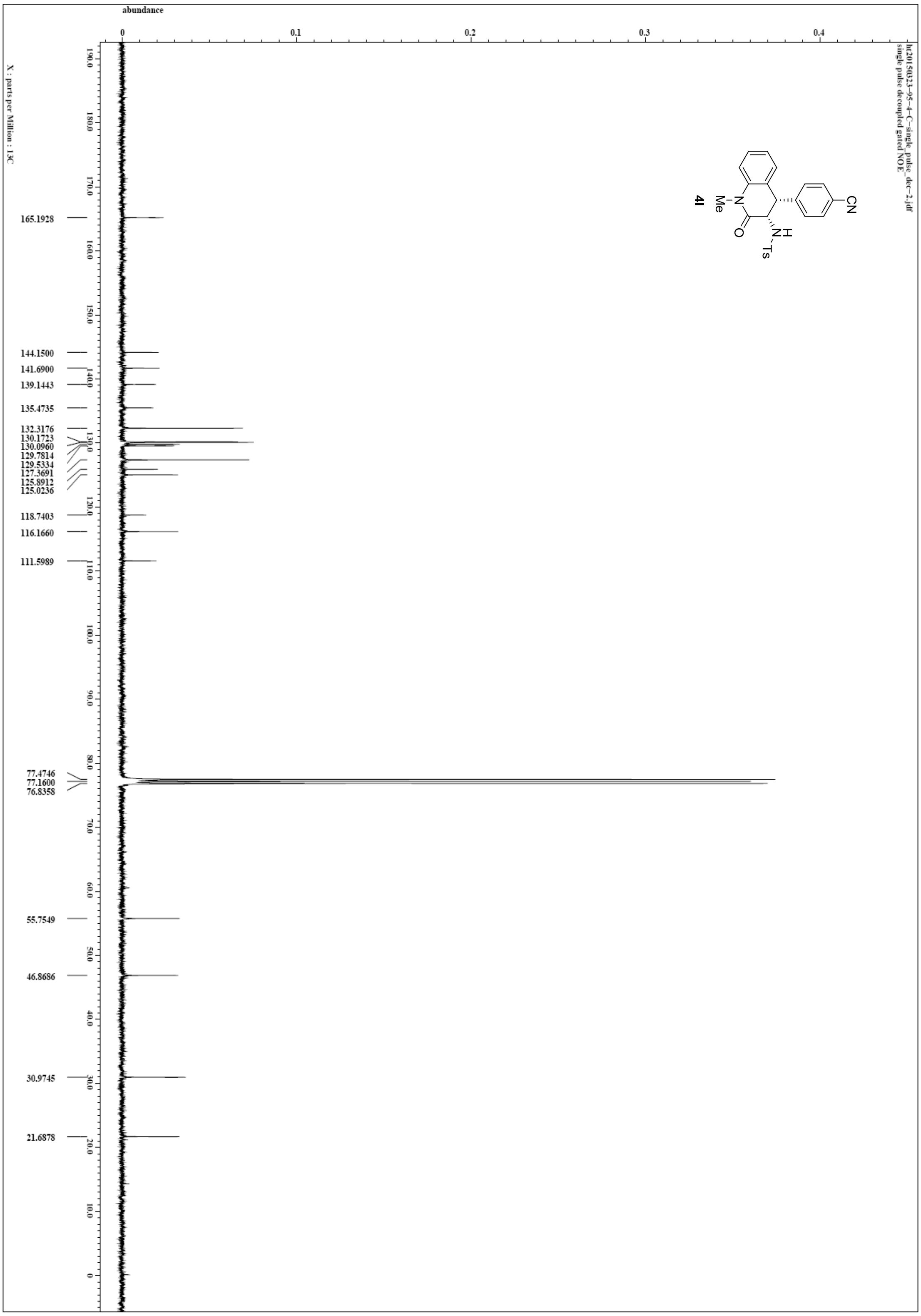
abundance
0
0.1
0.2
0.3
0.4
X : parts per Million : 13C
hz20150323-95-4-C-single_pulse_dec-2.jdf
single pulse decoupled gated NOE
4l
CN
Me
N
O
H
N
Ts
165.1928
144.1500
141.6900
139.1443
135.4735
132.3176
130.1723
130.0960
129.7814
129.5334
127.3691
125.8912
125.0236
118.7403
116.1660
111.5989
77.4746
77.1600
76.8358
55.7549
46.8686
30.9745
21.6878

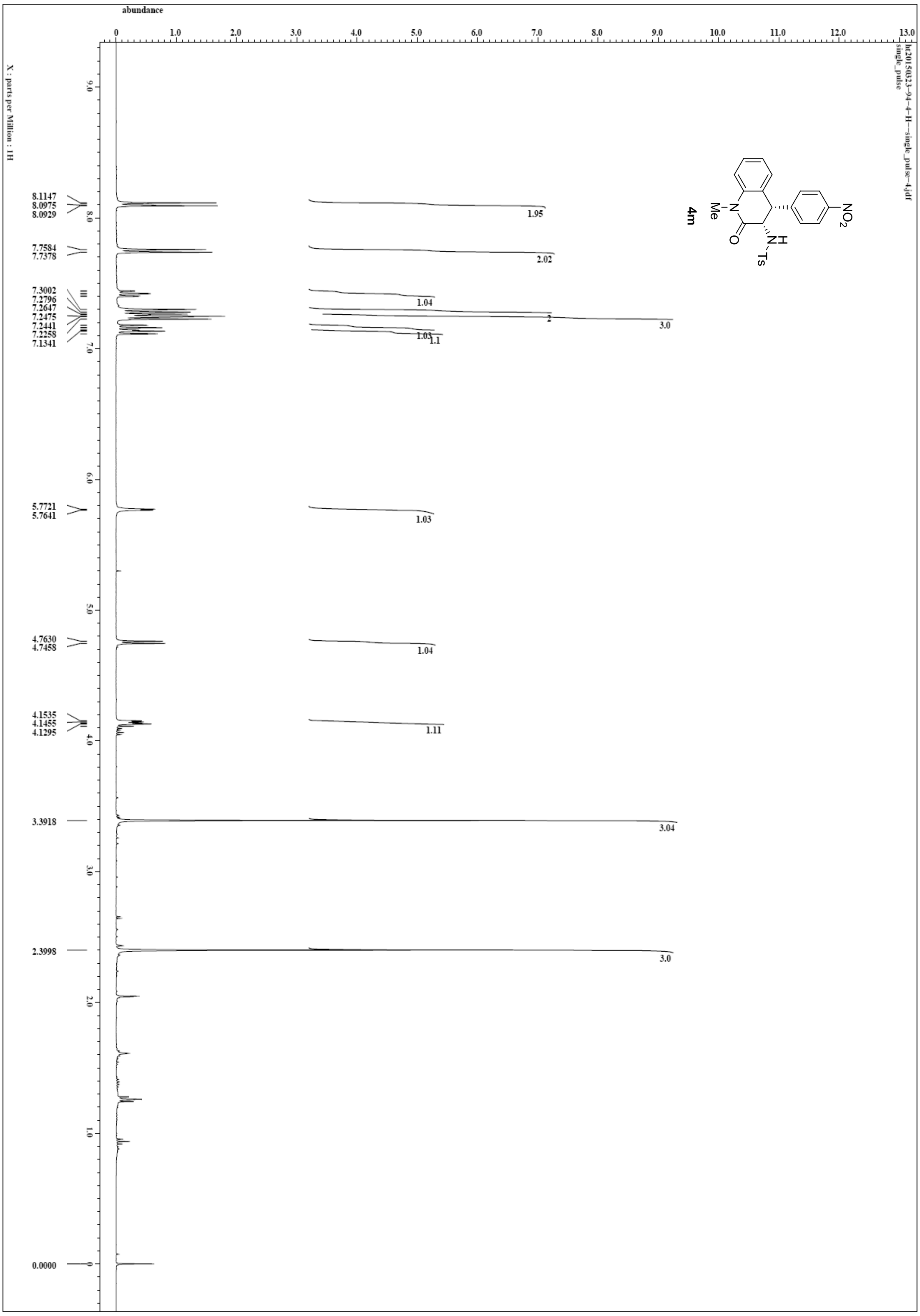
abundance
X : parts per Million : 1H
4m
8.1147
8.0975
8.0929
7.7584
7.7378
7.3002
7.2796
7.2647
7.2475
7.2441
7.2258
7.1341
5.7721
5.7641
4.7630
4.7458
4.1535
4.1455
4.1295
3.3918
2.3998
0.0000

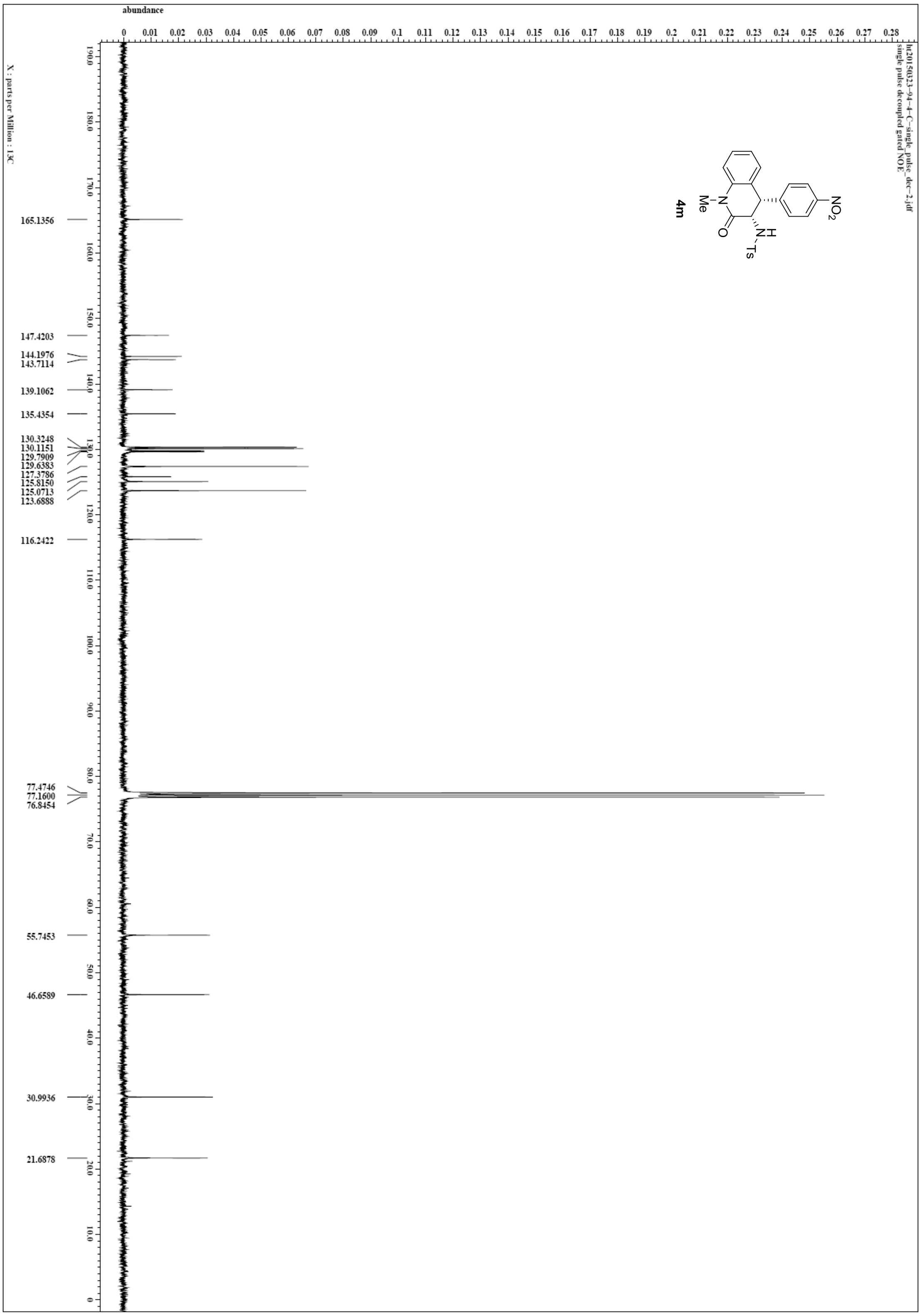

abundance
X : parts per Million : 13C
single pulse decoupled gated NOE
4m
165.1356
147.4203
144.1976
143.7114
139.1062
135.4354
130.3248
130.1151
129.7909
129.6383
127.3786
125.8150
125.0713
123.6888
116.2422
77.4746
77.1600
76.8454
55.7453
46.6589
30.9936
21.6878

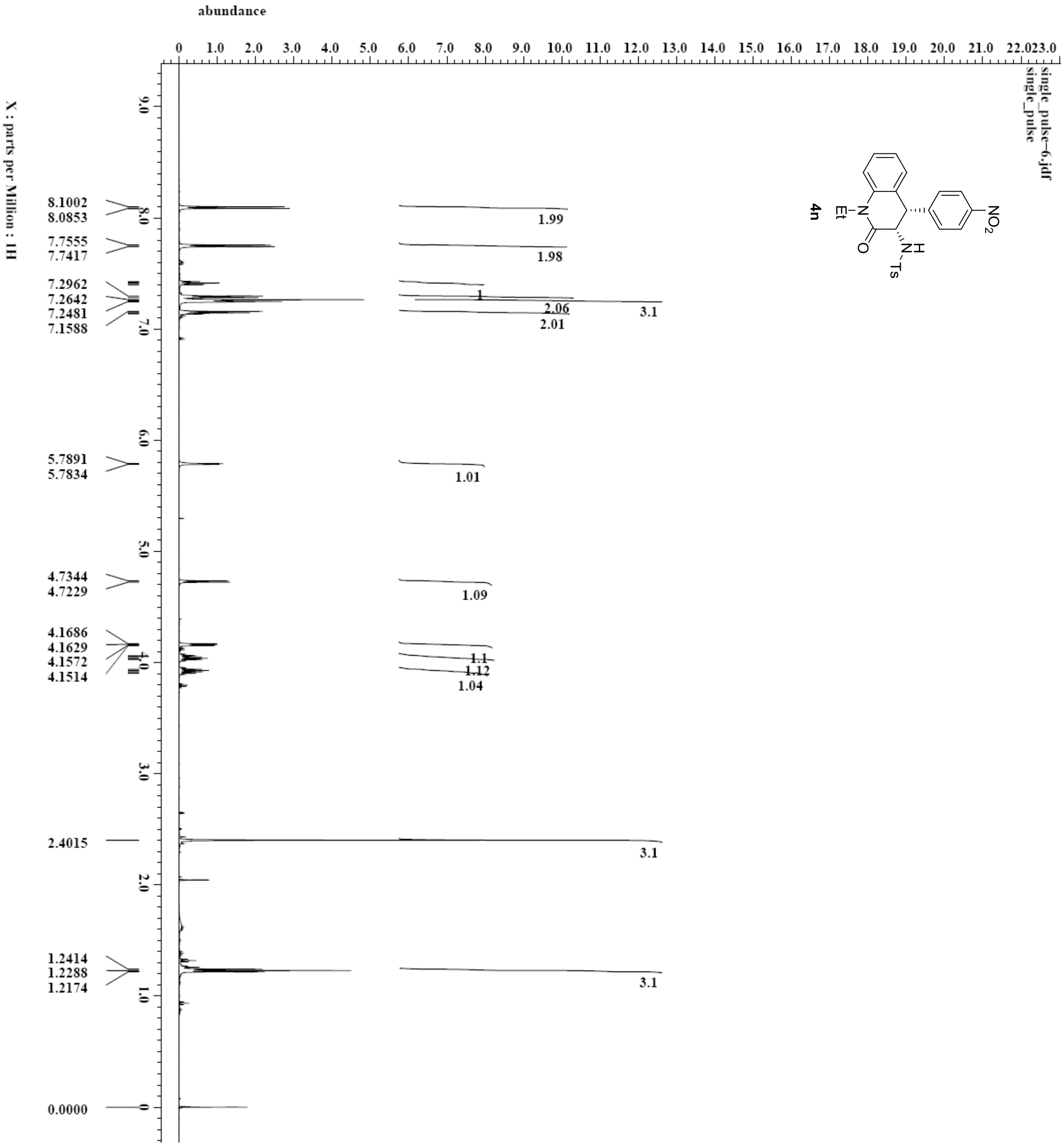
abundance
X : parts per Million : 1H
single_pulse-6.jdf
single_pulse
4n
8.1002
8.0853
7.7555
7.7417
7.2962
7.2642
7.2481
7.1588
5.7891
5.7834
4.7344
4.7229
4.1686
4.1629
4.1572
4.1514
2.4015
1.2414
1.2288
1.2174
0.0000
1.99
1.98
1
2.06
2.01
3.1
1.01
1.09
1.1
1.12
1.04

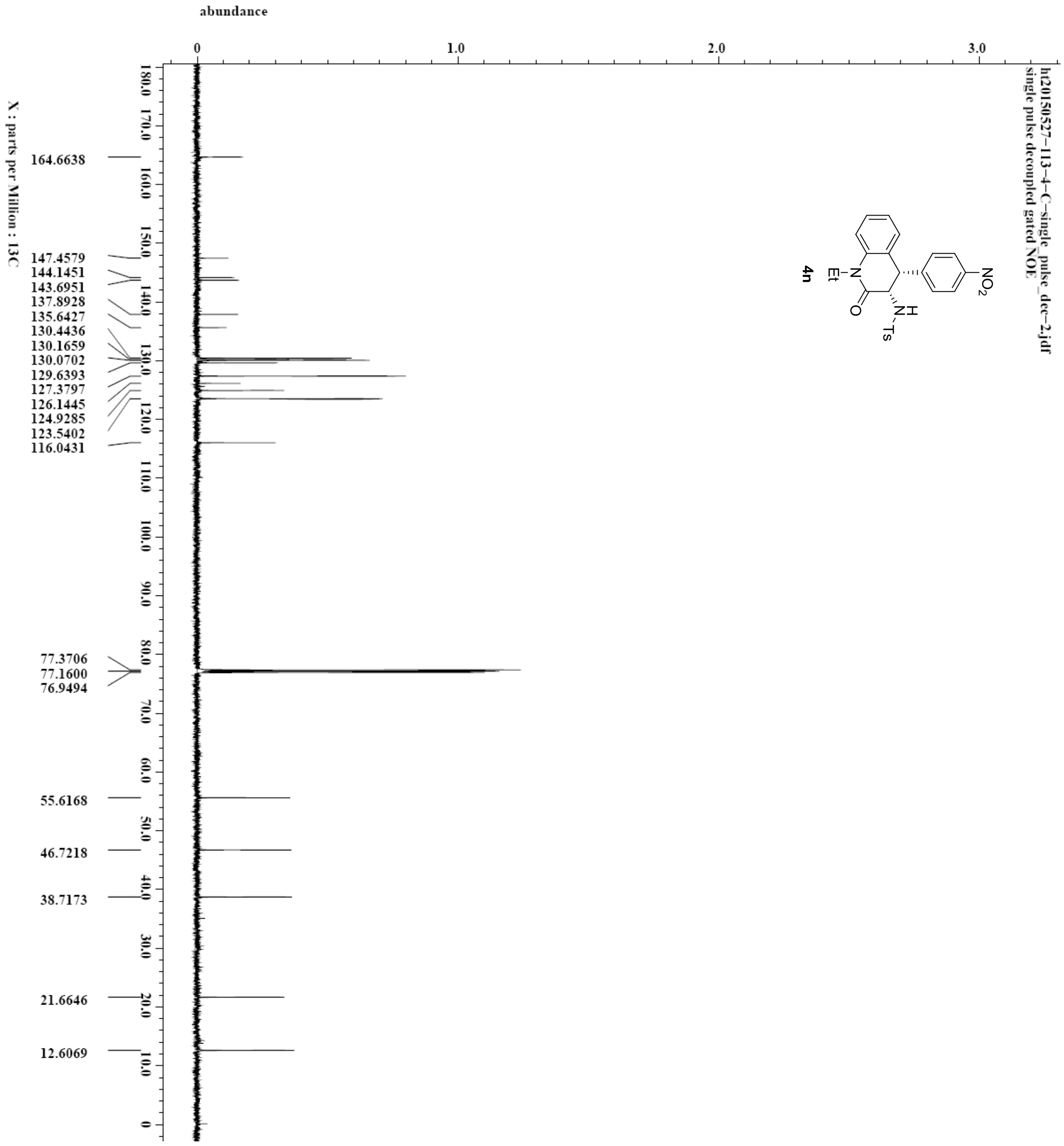

abundance
0
1.0
2.0
3.0
ht20150527–113–4–C–single_pulse_dec–2.jdf
single pulse decoupled gated NOE
4n
X : parts per Million : 13C
164.6638
147.4579
144.1451
143.6951
137.8928
135.6427
130.4436
130.1659
130.0702
129.6393
127.3797
126.1445
124.9285
123.5402
116.0431
77.3706
77.1600
76.9494
55.6168
46.7218
38.7173
21.6646
12.6069

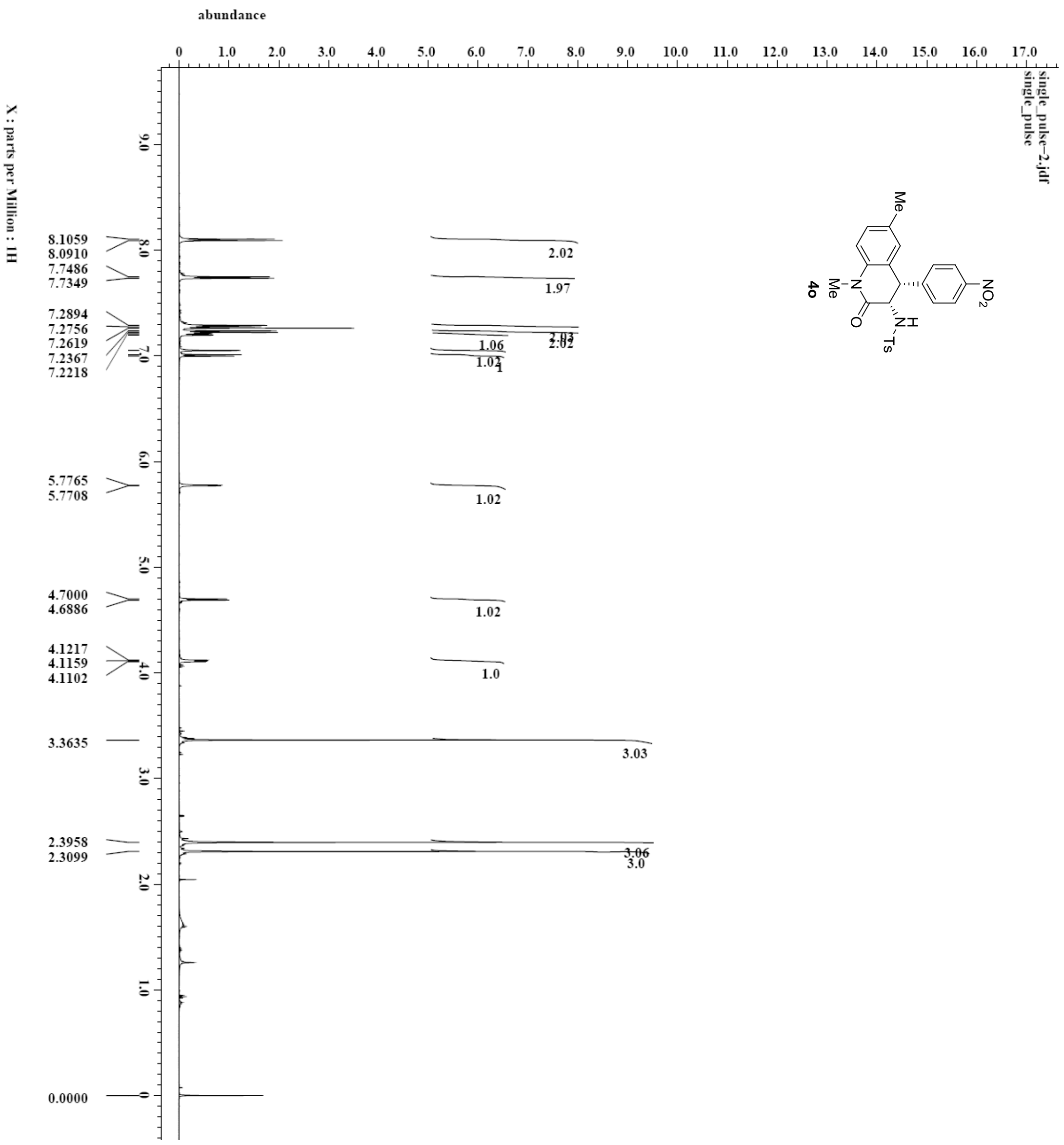
abundance
X : parts per Million : 1H
single_pulse-2.jdf
single_pulse
8.1059
8.0910
7.7486
7.7349
7.2894
7.2756
7.2619
7.2367
7.2218
5.7765
5.7708
4.7000
4.6886
4.1217
4.1159
4.1102
3.3635
2.3958
2.3099
0.0000
2.02
1.97
2.03
2.02
1.06
1.02
1.02
1.02
1.0
3.03
3.06
3.0
Me
N
Me
O
H
N
Ts
NO2
4o

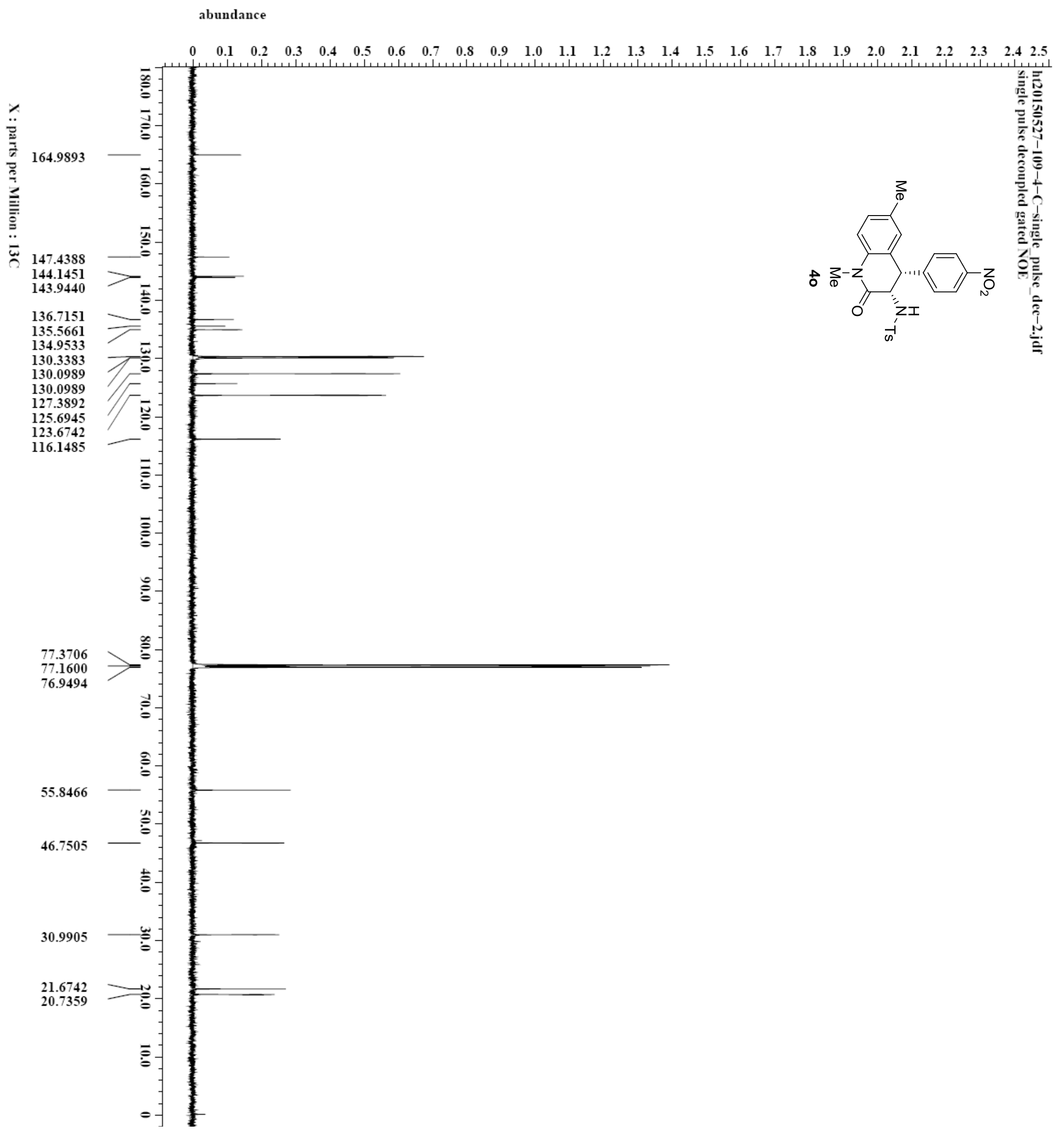
abundance
X : parts per Million : 13C
h20150527-109-4-C-single_pulse_dec-2.jdf
single pulse decoupled gated NOE
164.9893
147.4388
144.1451
143.9440
136.7151
135.5661
134.9533
130.3383
130.0989
130.0989
127.3892
125.6945
123.6742
116.1485
77.3706
77.1600
76.9494
55.8466
46.7505
30.9905
21.6742
20.7359
Me
N
Me
O
H
N
Ts
NO2
4o

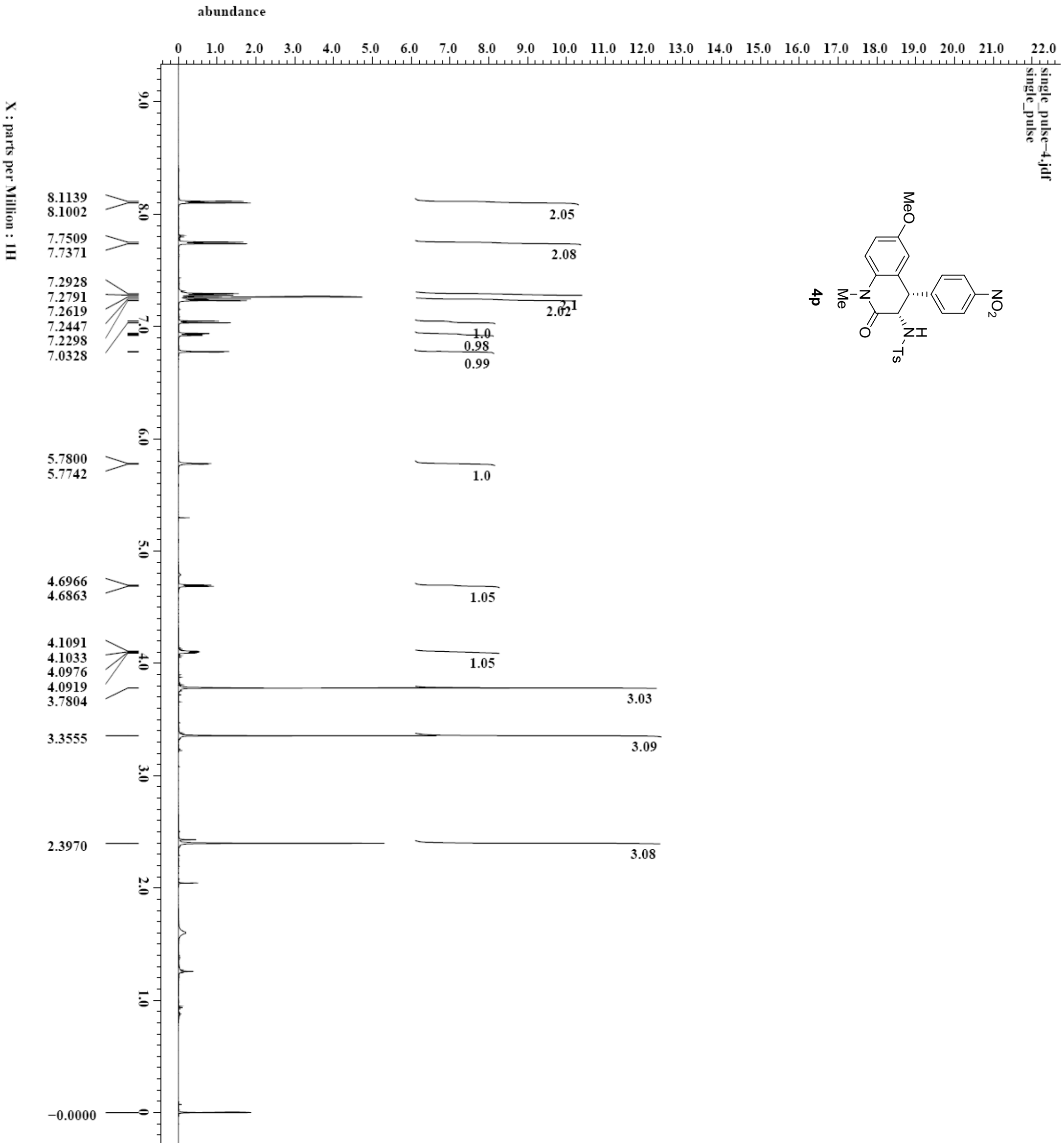
abundance
X : parts per Million : 1H
single_pulse-4.jdf
single_pulse
8.1139
8.1002
7.7509
7.7371
7.2928
7.2791
7.2619
7.2447
7.2298
7.0328
5.7800
5.7742
4.6966
4.6863
4.1091
4.1033
4.0976
4.0919
3.7804
3.3555
2.3970
-0.0000
2.05
2.08
2.02
2.1
1.0
0.98
0.99
1.0
1.05
1.05
3.03
3.09
3.08
MeO
NO2
Me
N
O
H
Ts
4p

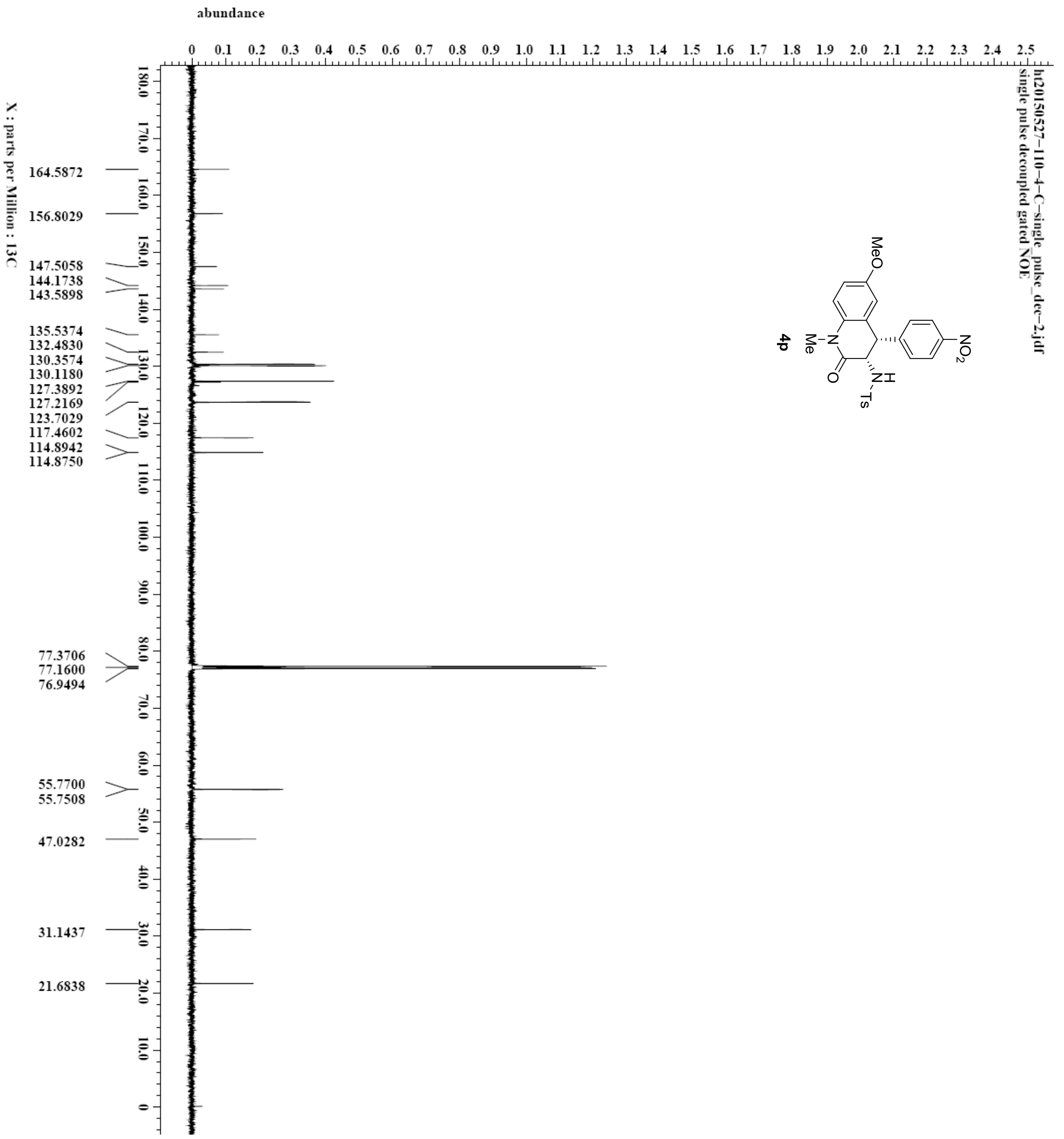
abundance
h20150527-110-4-C-single_pulse_dec-2.jdf
single pulse decoupled gated NOE
X : parts per Million : 13C
164.5872
156.8029
147.5058
144.1738
143.5898
135.5374
132.4830
130.3574
130.1180
127.3892
127.2169
123.7029
117.4602
114.8942
114.8750
77.3706
77.1600
76.9494
55.7700
55.7508
47.0282
31.1437
21.6838
MeO
NO2
N
Me
O
H
N
Ts
4p

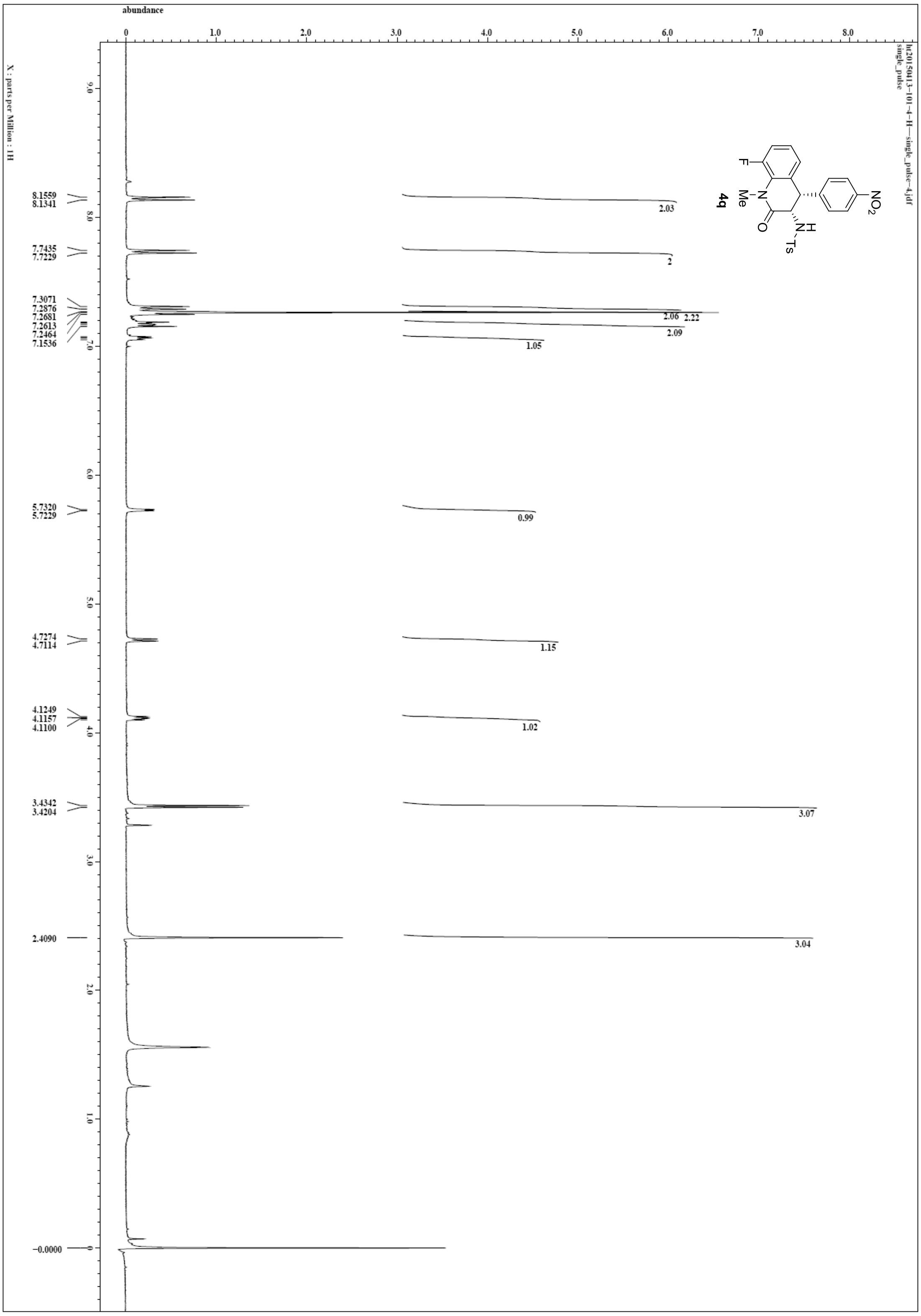

abundance
X : parts per Million : 1H
4q
8.1559
8.1341
7.7435
7.7229
7.3071
7.2876
7.2681
7.2613
7.2464
7.1536
5.7320
5.7229
4.7274
4.7114
4.1249
4.1157
4.1100
3.4342
3.4204
2.4090
-0.0000
2.03
2
2.06
2.22
2.09
1.05
0.99
1.15
1.02
3.07
3.04

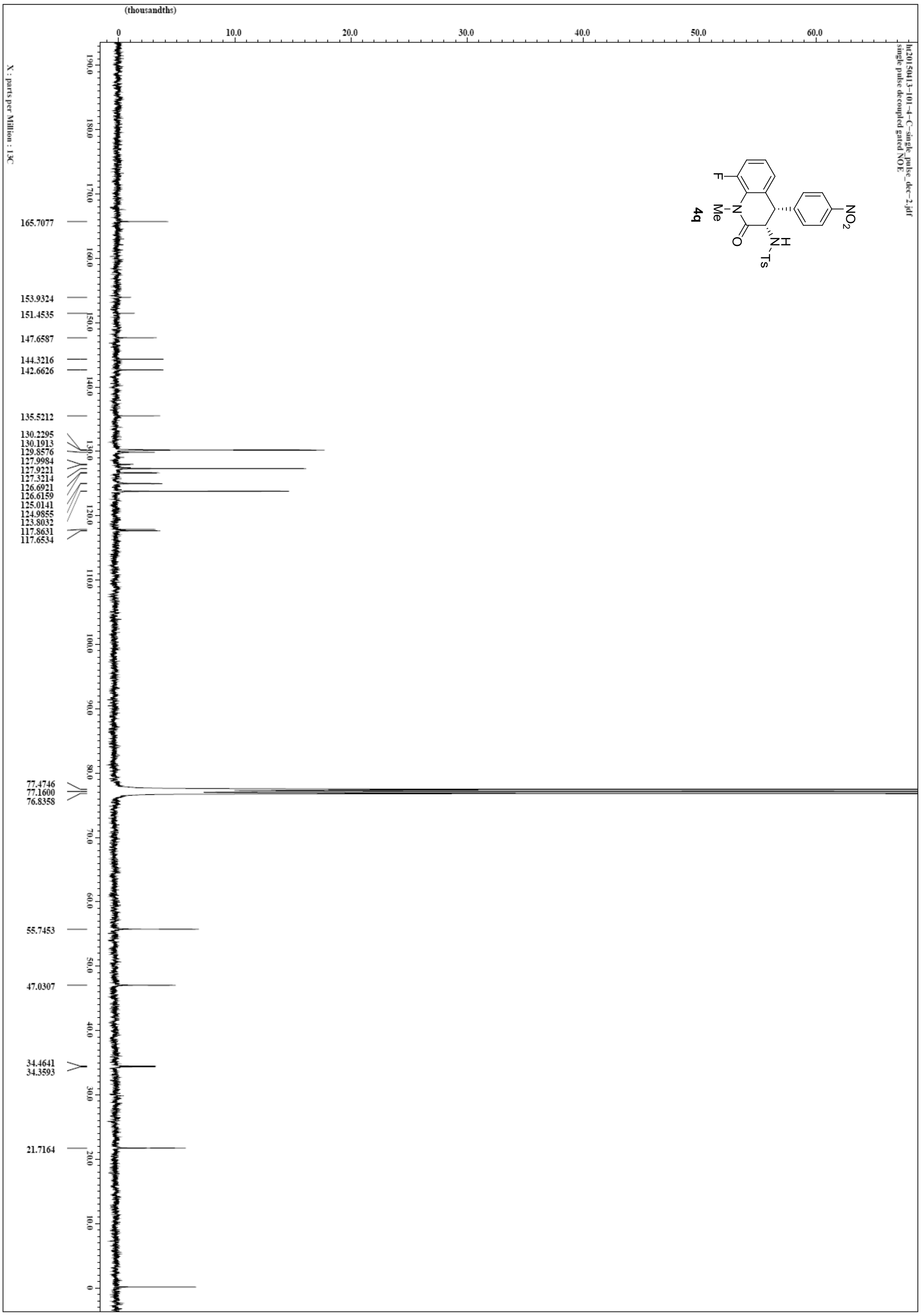
(thousandths)
X : parts per Million : 13C
165.7077
153.9324
151.4535
147.6587
144.3216
142.6626
135.5212
130.2295
130.1913
129.8576
127.9984
127.9221
127.3214
126.6921
126.6159
125.0141
124.9855
123.8032
117.8631
117.6534
77.4746
77.1600
76.8358
55.7453
47.0307
34.4641
34.3593
21.7164
4q

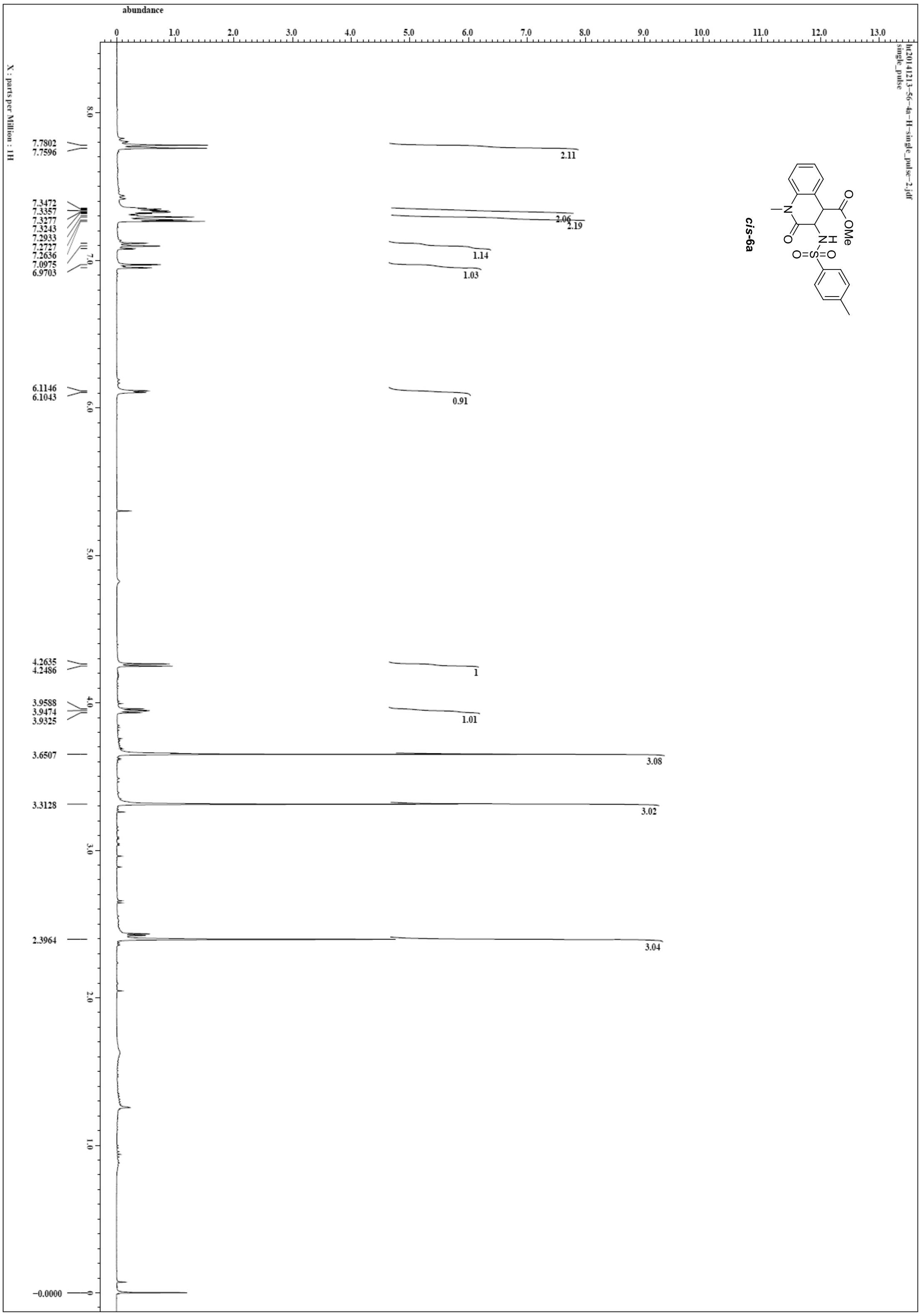
abundance
X : parts per Million : 1H
7.7802
7.7596
7.3472
7.3357
7.3277
7.3243
7.2933
7.2727
7.2636
7.0975
6.9703
6.1146
6.1043
4.2635
4.2486
3.9588
3.9474
3.9325
3.6507
3.3128
2.3964
-0.0000
2.11
2.06
2.19
1.14
1.03
0.91
1
1.01
3.08
3.02
3.04
OMe
cis-6a
single_pulse

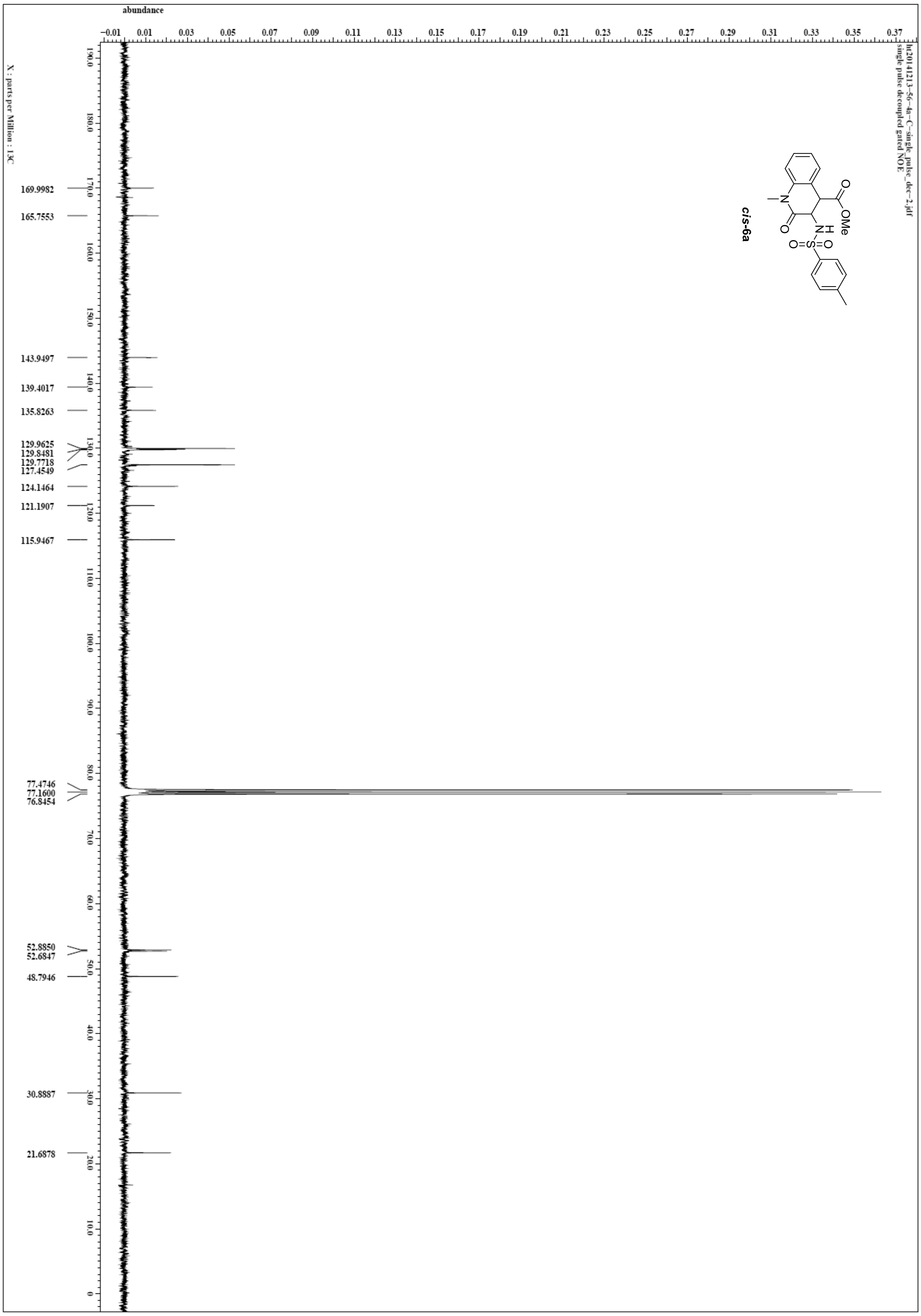
abundance
X : parts per Million : 13C
cis-6a
OMe
169.9982
165.7553
143.9497
139.4017
135.8263
129.9625
129.8481
129.7718
127.4549
124.1464
121.1907
115.9467
77.4746
77.1600
76.8454
52.8850
52.6847
48.7946
30.8887
21.6878

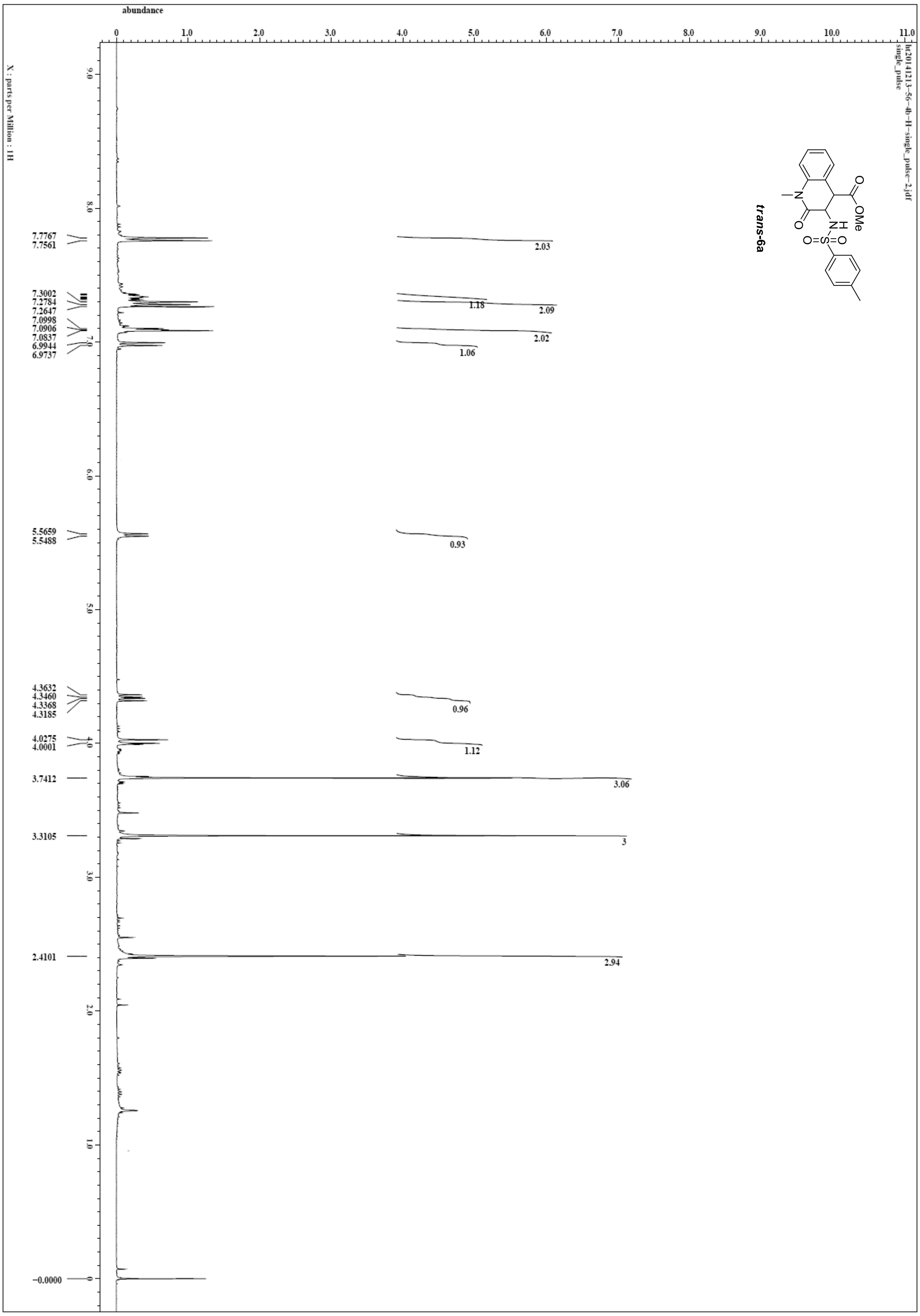
trans-6a
X : parts per Million : 1H
abundance
7.7767
7.7561
7.3002
7.2784
7.2647
7.0998
7.0906
7.0837
6.9944
6.9737
5.5659
5.5488
4.3632
4.3460
4.3368
4.3185
4.0275
4.0001
3.7412
3.3105
2.4101
-0.0000
2.03
1.18
2.09
2.02
1.06
0.93
0.96
1.12
3.06
3
2.94

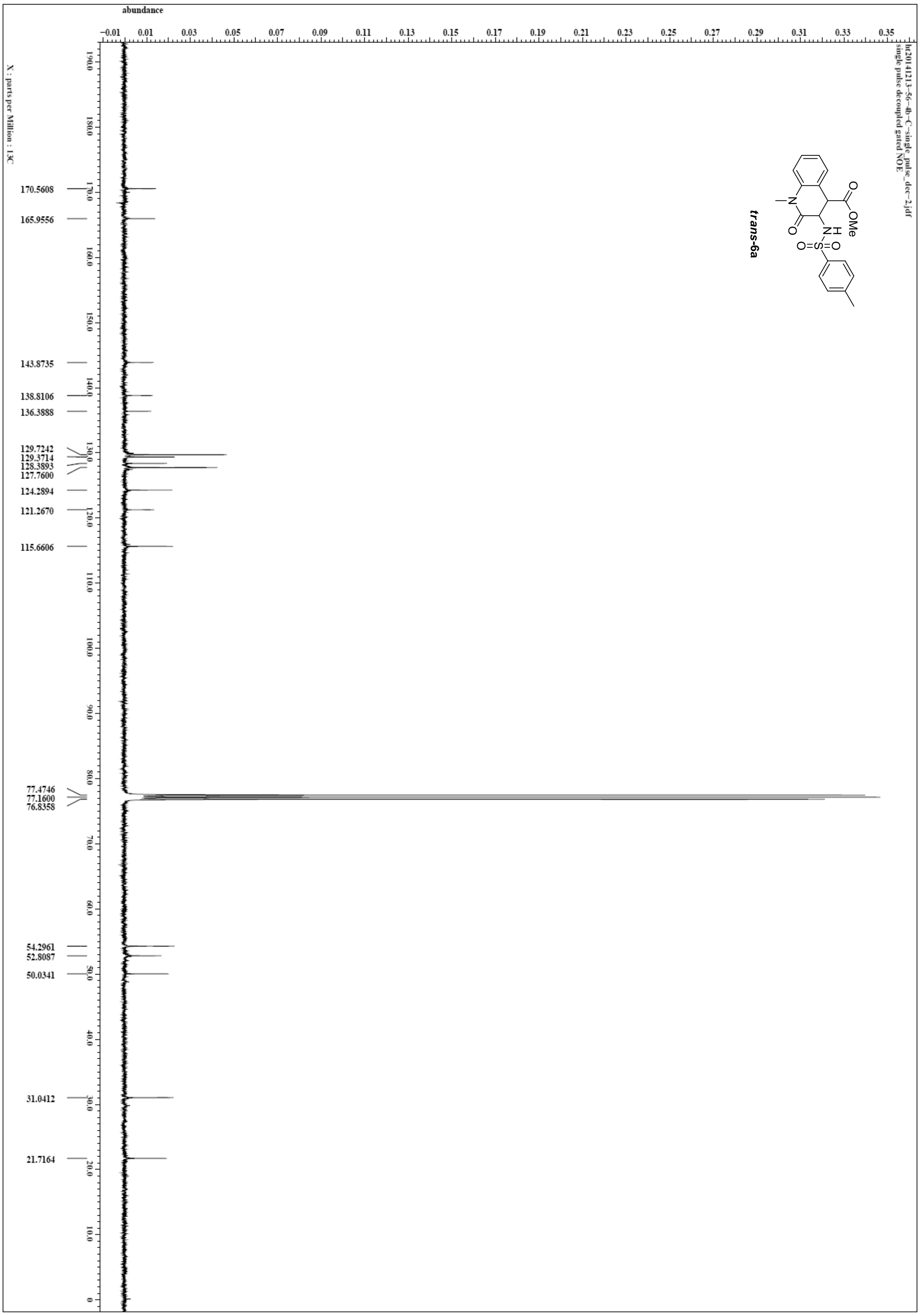
abundance
X : parts per Million : 13C
170.5608
165.9556
143.8735
138.8106
136.3888
129.7242
129.3714
128.3893
127.7600
124.2894
121.2670
115.6606
77.4746
77.1600
76.8358
54.2961
52.8087
50.0341
31.0412
21.7164
trans-6a

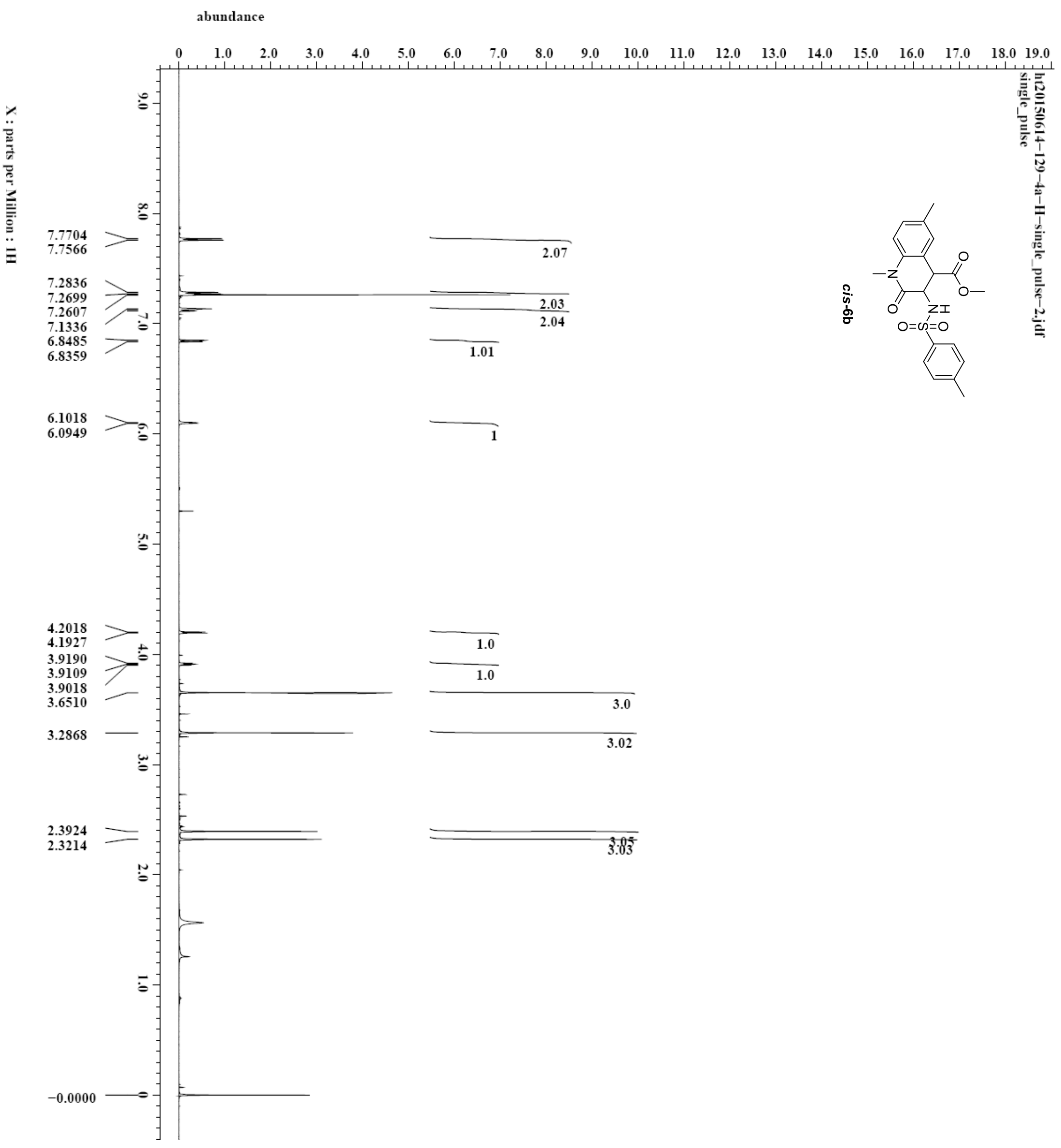
abundance
X : parts per Million : 1H
ht20150614−129−4a−H−single_pulse−2.jdf
single_pulse
cis-6b
7.7704
7.7566
7.2836
7.2699
7.2607
7.1336
6.8485
6.8359
6.1018
6.0949
4.2018
4.1927
3.9190
3.9109
3.9018
3.6510
3.2868
2.3924
2.3214
−0.0000
2.07
2.03
2.04
1.01
1
1.0
1.0
3.0
3.02
3.05
3.03

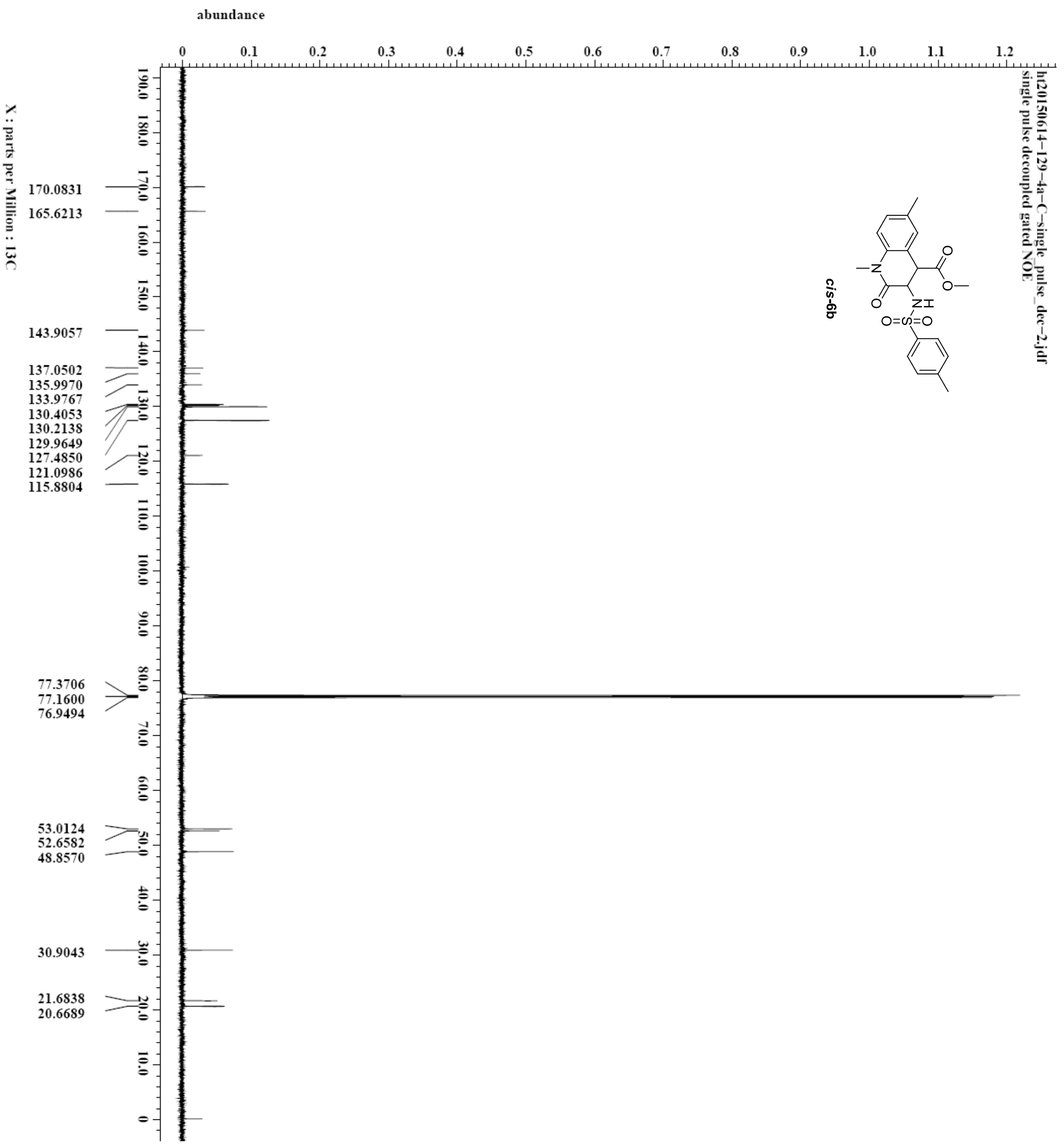

h20150614−129−4a−C−single_pulse_dec−2.jdf
single pulse decoupled gated NOE
cis-6b
abundance
X : parts per Million : 13C
170.0831
165.6213
143.9057
137.0502
135.9970
133.9767
130.4053
130.2138
129.9649
127.4850
121.0986
115.8804
77.3706
77.1600
76.9494
53.0124
52.6582
48.8570
30.9043
21.6838
20.6689

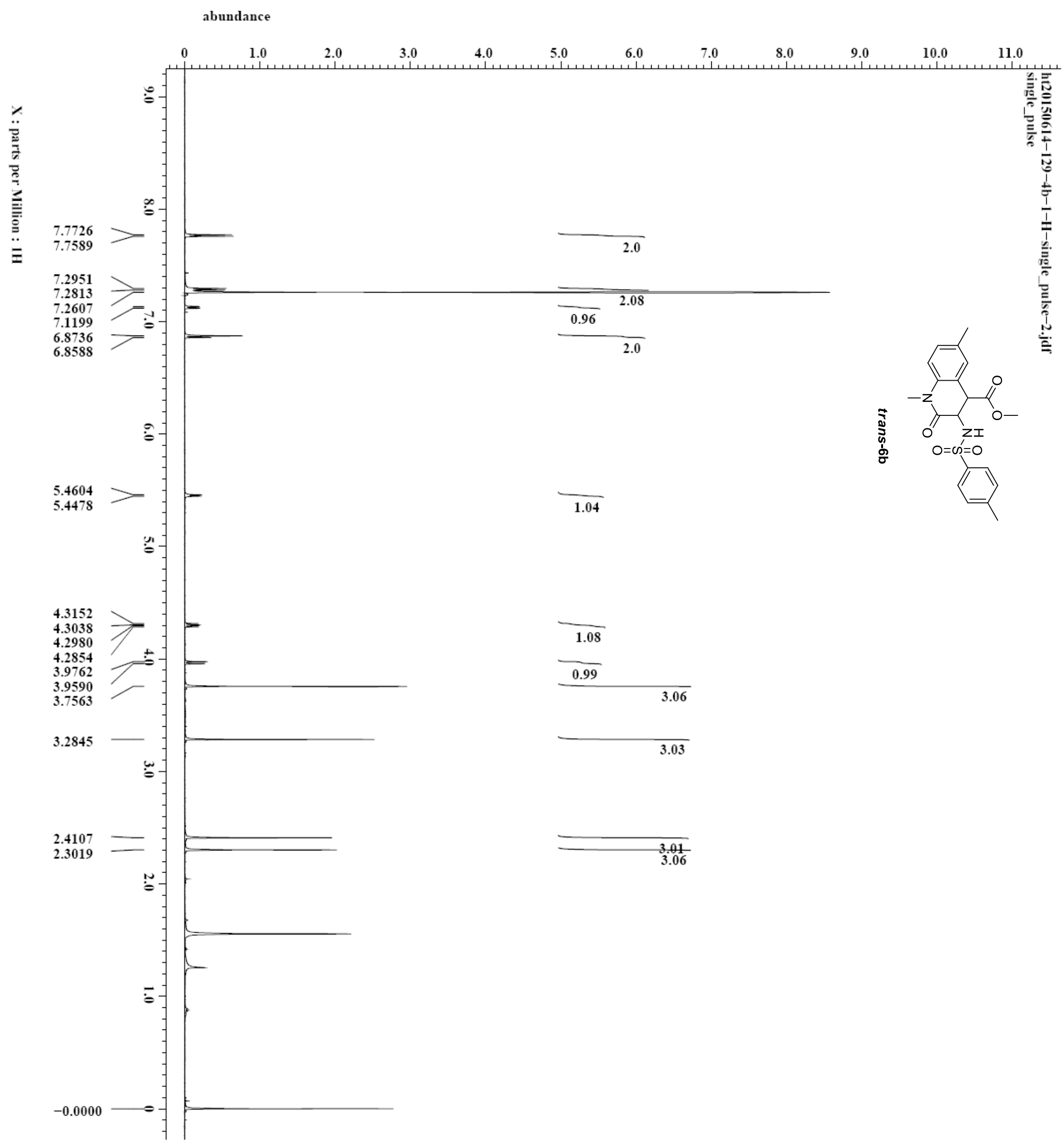

abundance
X : parts per Million : 1H
ht20150614-129-4b-1-H-single_pulse-2.jdf
single_pulse
trans-6b
7.7726
7.7589
7.2951
7.2813
7.2607
7.1199
6.8736
6.8588
5.4604
5.4478
4.3152
4.3038
4.2980
4.2854
3.9762
3.9590
3.7563
3.2845
2.4107
2.3019
-0.0000
2.0
2.08
0.96
2.0
1.04
1.08
0.99
3.06
3.03
3.01
3.06

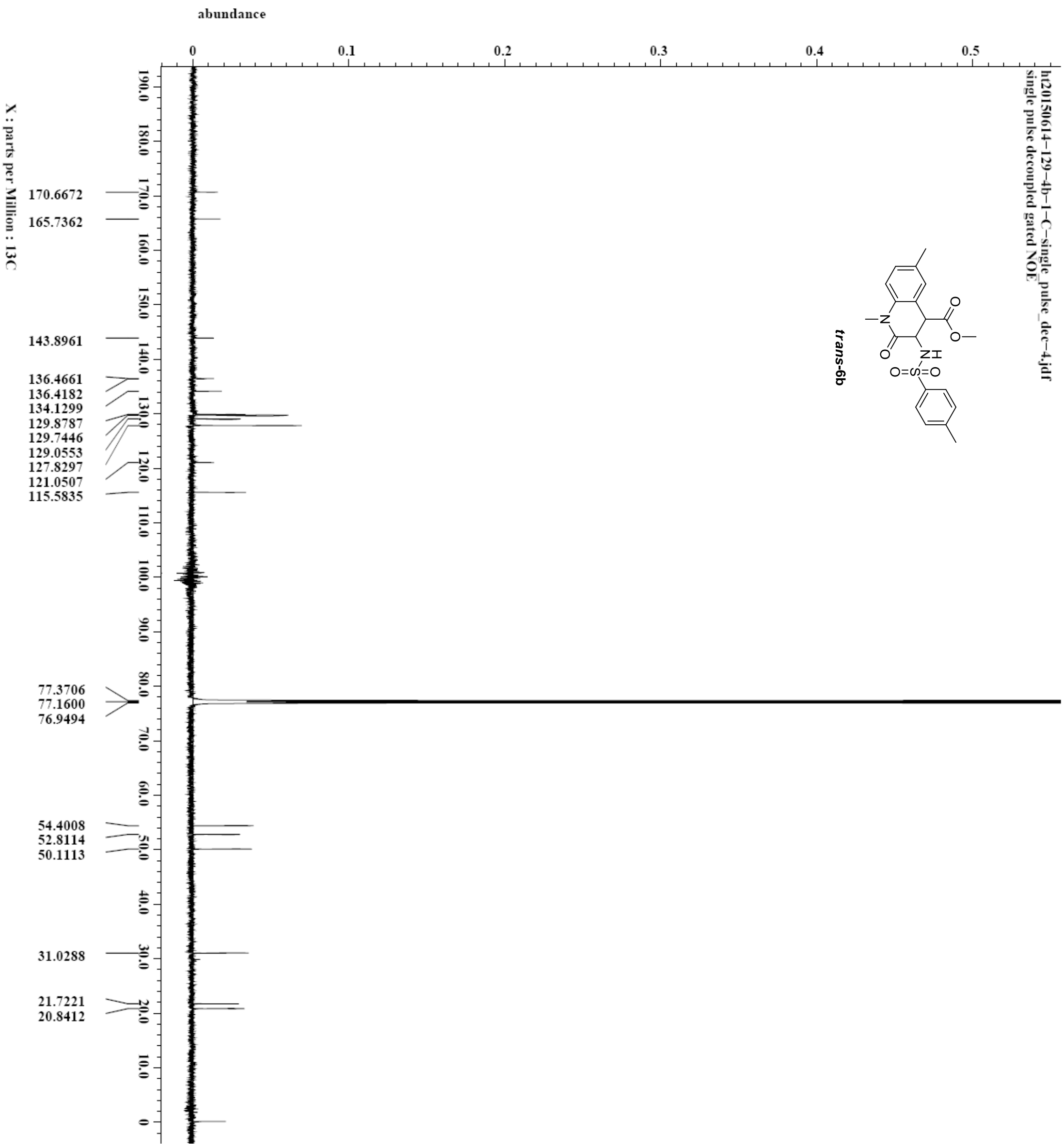
abundance
0
0.1
0.2
0.3
0.4
0.5
ht20150614-129-4b-1-C-single_pulse_dec-4.jdf
single pulse decoupled gated NOE
trans-6b
X : parts per Million : 13C
170.6672
165.7362
143.8961
136.4661
136.4182
134.1299
129.8787
129.7446
129.0553
127.8297
121.0507
115.5835
77.3706
77.1600
76.9494
54.4008
52.8114
50.1113
31.0288
21.7221
20.8412

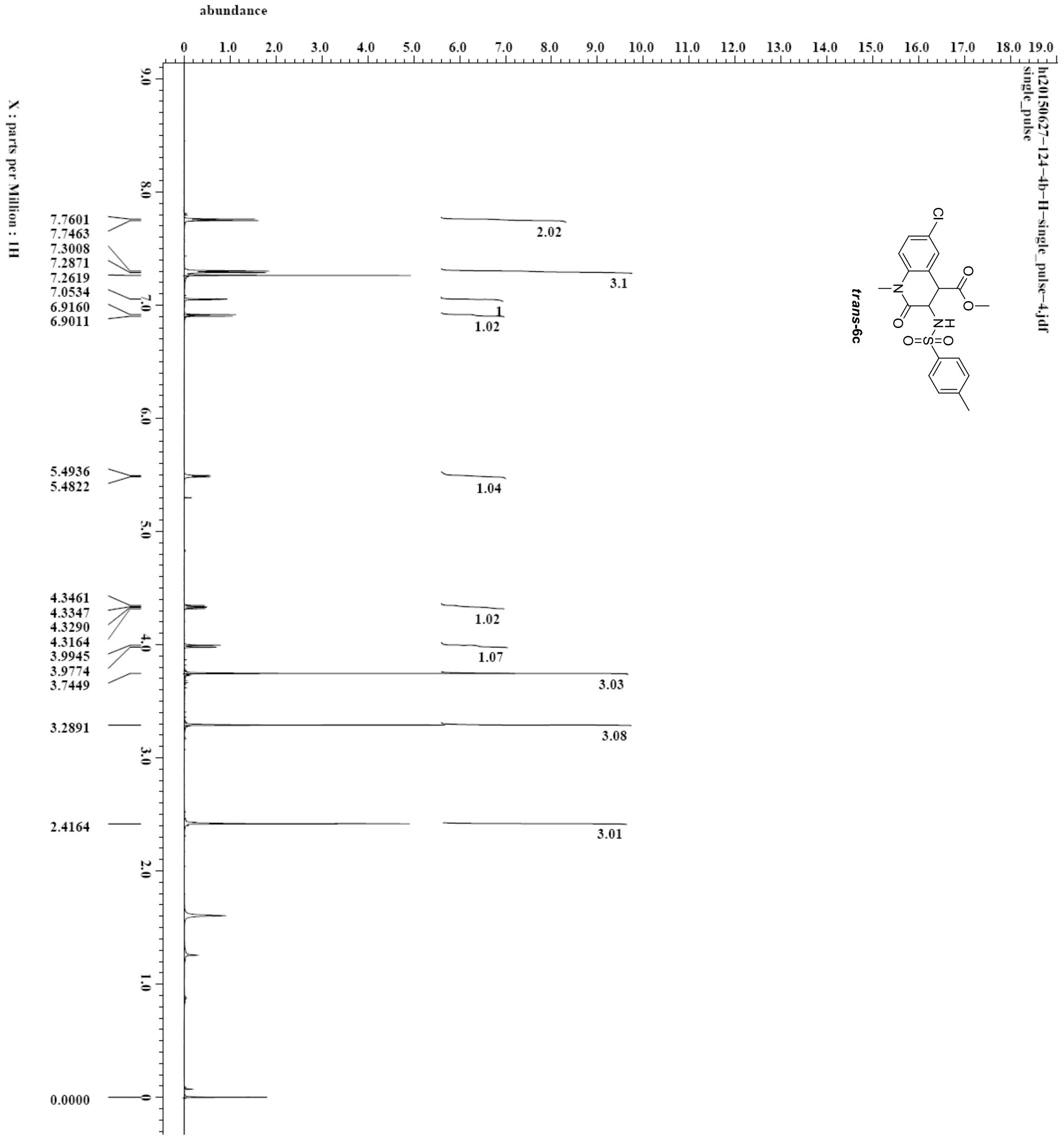
abundance
X : parts per Million : 1H
h20150627-124-4b-H-single_pulse-4.jdf
single_pulse
7.7601
7.7463
7.3008
7.2871
7.2619
7.0534
6.9160
6.9011
5.4936
5.4822
4.3461
4.3347
4.3290
4.3164
3.9945
3.9774
3.7449
3.2891
2.4164
0.0000
2.02
3.1
1
1.02
1.04
1.02
1.07
3.03
3.08
3.01
trans-6c

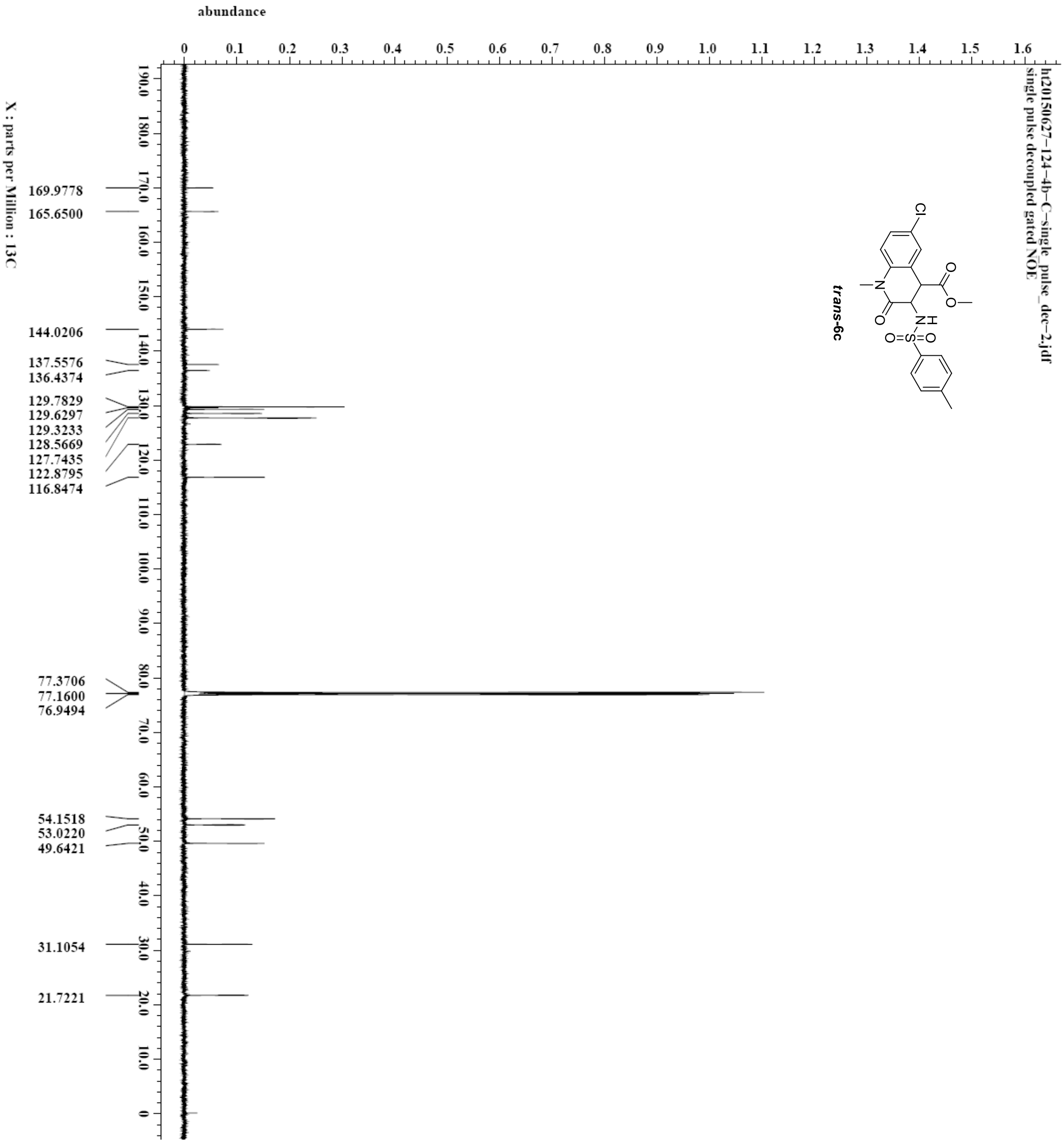
abundance
ht20150627-124-4b-C-single_pulse_dec-2.jdf
single pulse decoupled gated NOE
trans-6c
X : parts per Million : 13C
169.9778
165.6500
144.0206
137.5576
136.4374
129.7829
129.6297
129.3233
128.5669
127.7435
122.8795
116.8474
77.3706
77.1600
76.9494
54.1518
53.0220
49.6421
31.1054
21.7221

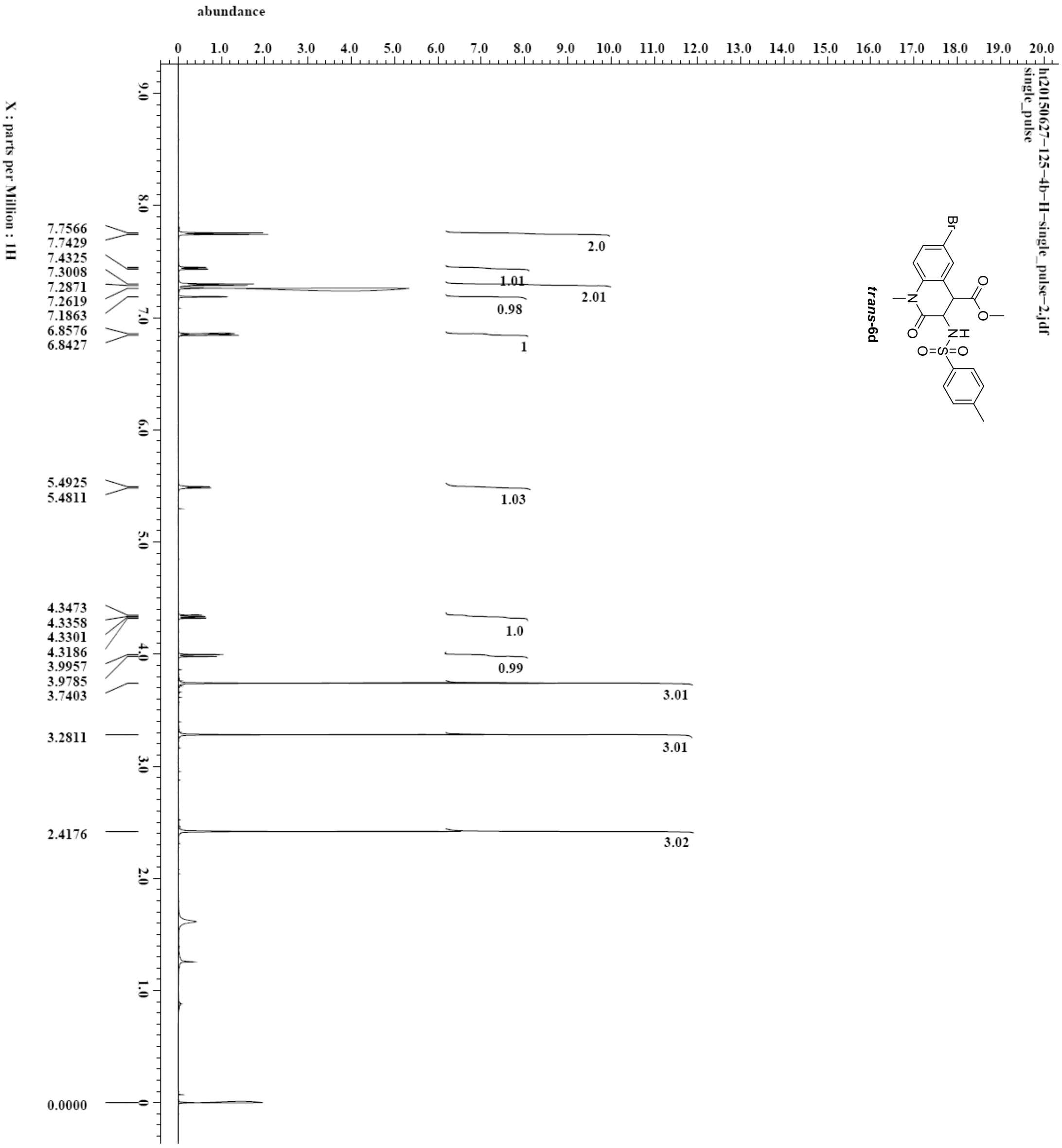
abundance
X : parts per Million : 1H
h20150627−125−4b−H−single_pulse−2.jdf
single_pulse
7.7566
7.7429
7.4325
7.3008
7.2871
7.2619
7.1863
6.8576
6.8427
5.4925
5.4811
4.3473
4.3358
4.3301
4.3186
3.9957
3.9785
3.7403
3.2811
2.4176
0.0000
2.0
1.01
2.01
0.98
1
1.03
1.0
0.99
3.01
3.01
3.02
Br
trans-6d

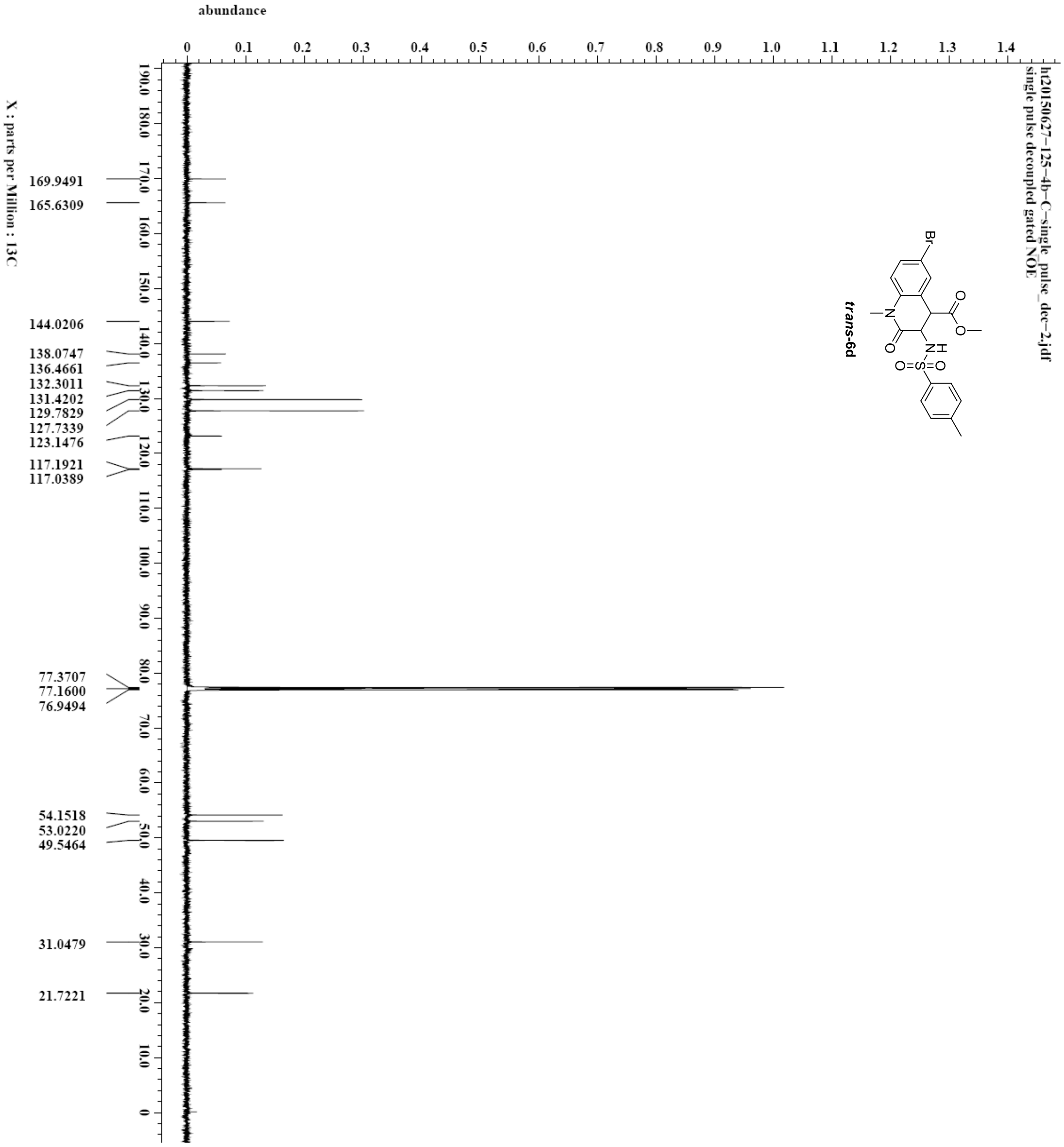
abundance
0
0.1
0.2
0.3
0.4
0.5
0.6
0.7
0.8
0.9
1.0
1.1
1.2
1.3
1.4
hz20150627-125-4b-C-single_pulse_dec-2.jdf
single pulse decoupled gated NOE
X : parts per Million : 13C
169.9491
165.6309
144.0206
138.0747
136.4661
132.3011
131.4202
129.7829
127.7339
123.1476
117.1921
117.0389
77.3707
77.1600
76.9494
54.1518
53.0220
49.5464
31.0479
21.7221
190.0
180.0
170.0
160.0
150.0
140.0
130.0
120.0
110.0
100.0
90.0
80.0
70.0
60.0
50.0
40.0
30.0
20.0
10.0
0
Br
N
O
H
S
trans-6d

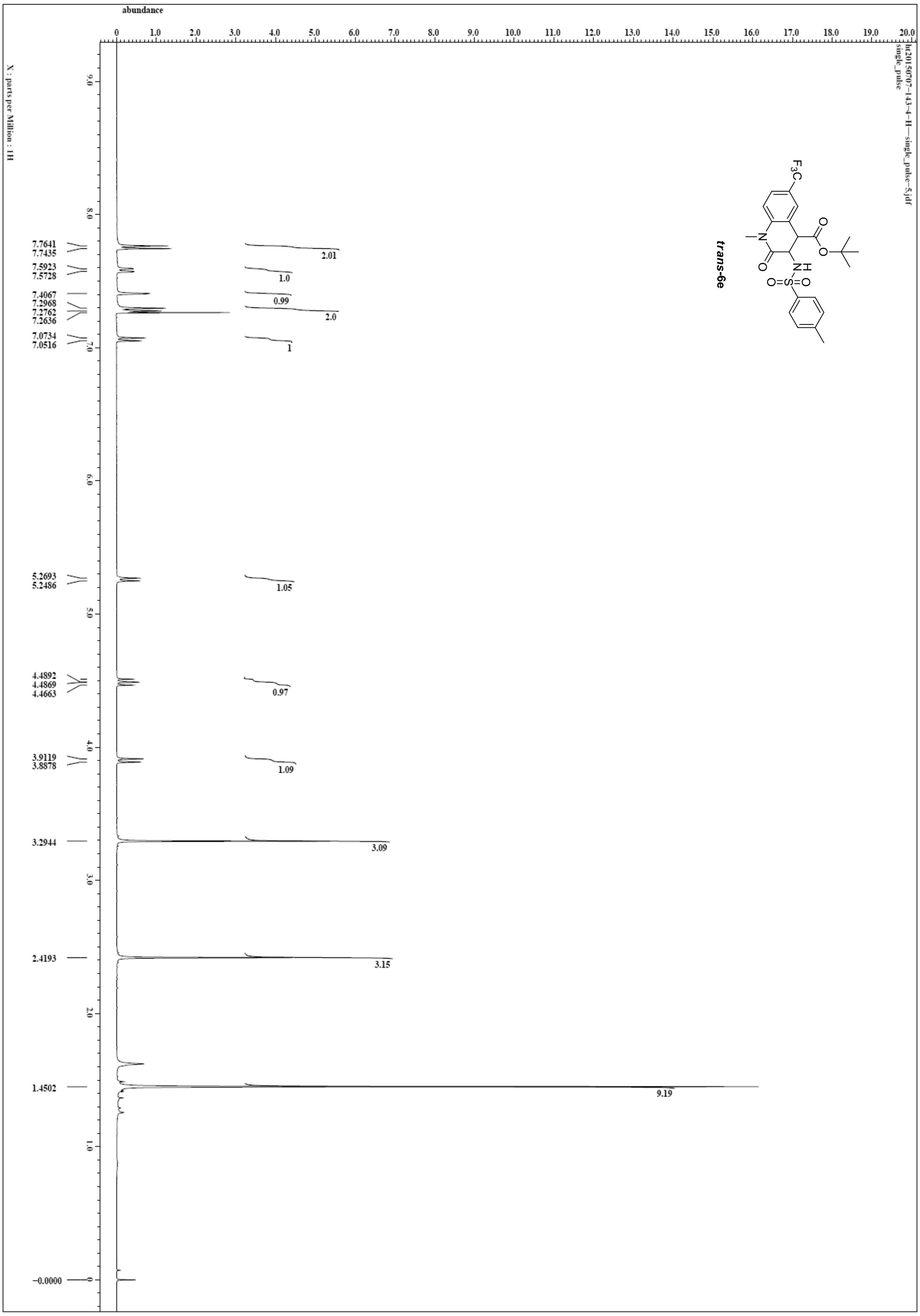
abundance
X : parts per Million : 1H
trans-6e
7.7641
7.7435
7.5923
7.5728
7.4067
7.2968
7.2762
7.2636
7.0734
7.0516
5.2693
5.2486
4.4892
4.4869
4.4663
3.9119
3.8878
3.2944
2.4193
1.4502
-0.0000
2.01
1.0
0.99
2.0
1
1.05
0.97
1.09
3.09
3.15
9.19

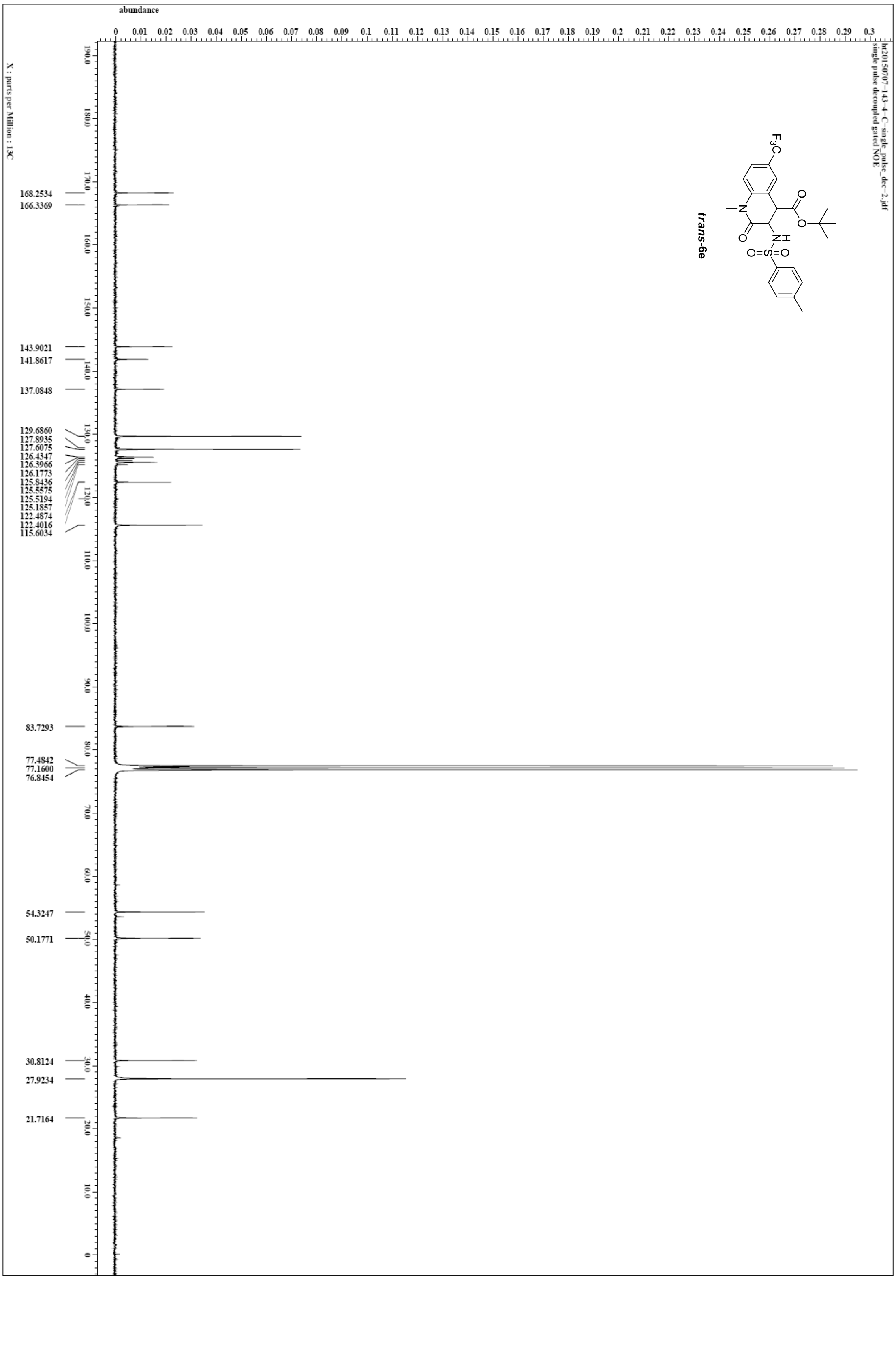
abundance
X : parts per Million : 13C
trans-6e
168.2534
166.3369
143.9021
141.8617
137.0848
129.6860
127.8935
127.6075
126.4347
126.3966
126.1773
125.8436
125.5575
125.5194
125.1857
122.4874
122.4016
115.6034
83.7293
77.4842
77.1600
76.8454
54.3247
50.1771
30.8124
27.9234
21.7164

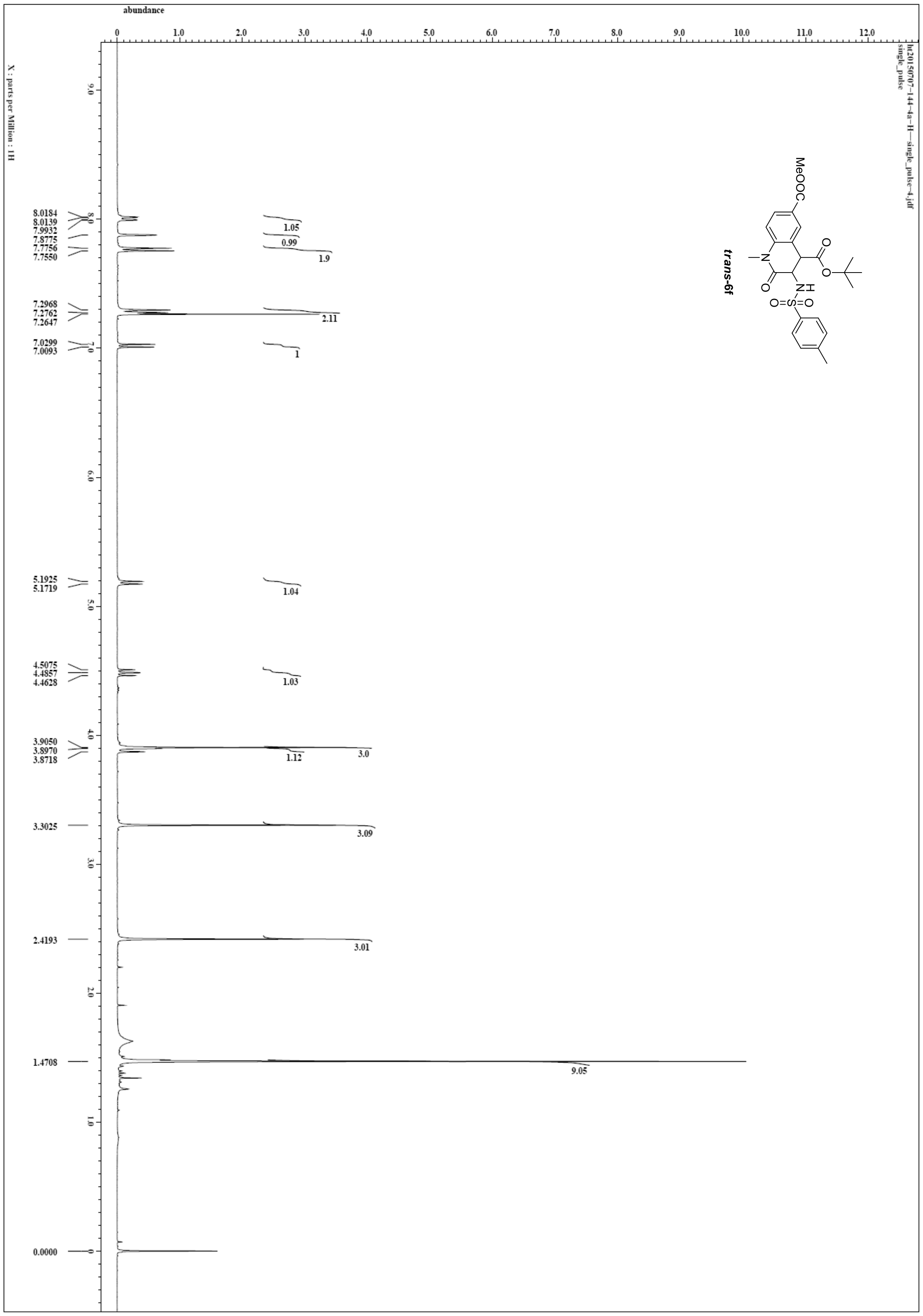
abundance
X : parts per Million : 1H
8.0184
8.0139
7.9932
7.8775
7.7756
7.7550
7.2968
7.2762
7.2647
7.0299
7.0093
5.1925
5.1719
4.5075
4.4857
4.4628
3.9050
3.8970
3.8718
3.3025
2.4193
1.4708
0.0000
1.05
0.99
1.9
2.11
1
1.04
1.03
1.12
3.0
3.09
3.01
9.05
MeOOC
trans-6f

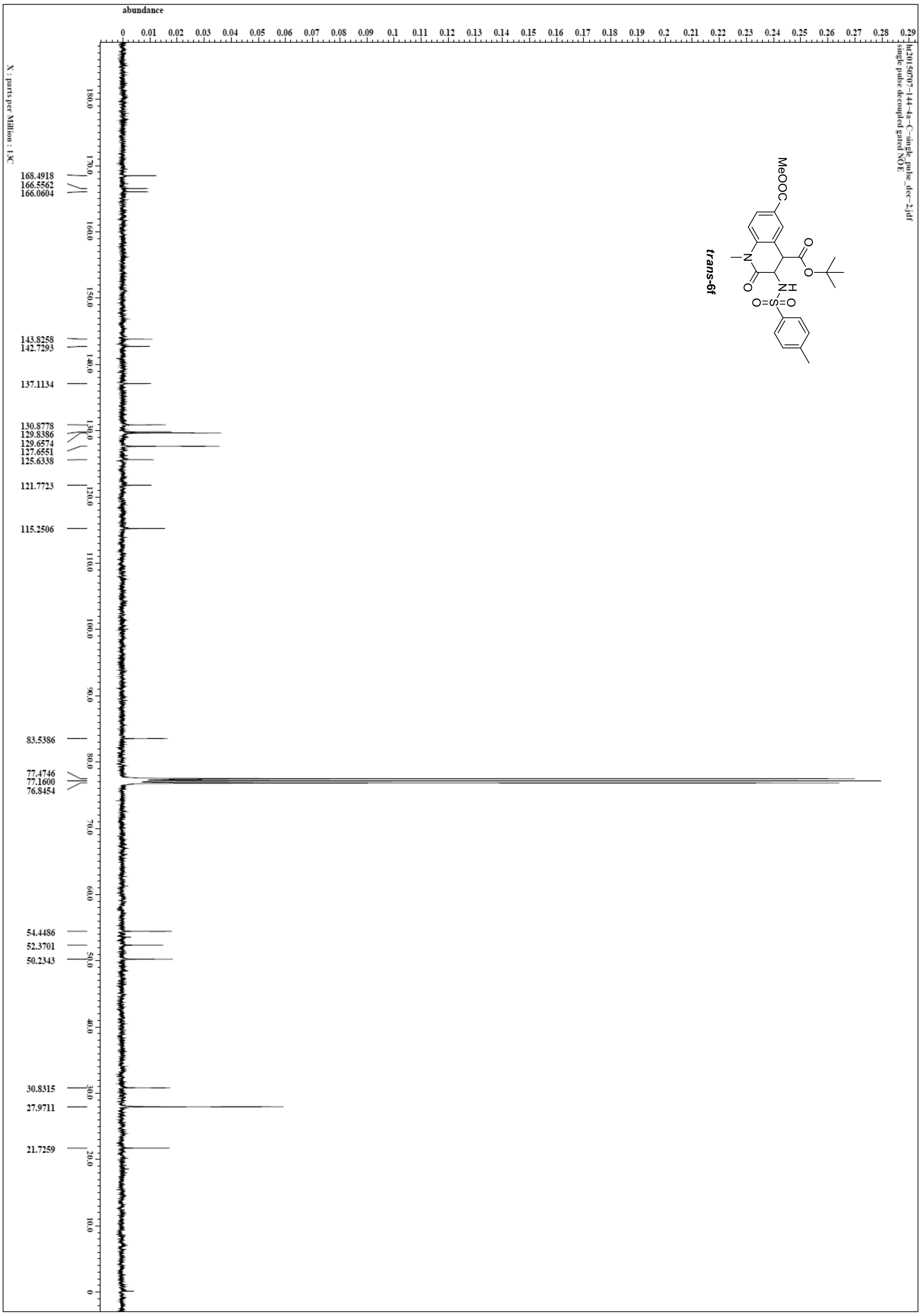
abundance
X : parts per Million : 13C
hr20150707-144-4a-C-single_pulse_dec-2.jdf
single pulse decoupled gated NOE
trans-6f
MeOOC
168.4918
166.5562
166.0604
143.8258
142.7293
137.1134
130.8778
129.8386
129.6574
127.6551
125.6338
121.7723
115.2506
83.5386
77.4746
77.1600
76.8454
54.4486
52.3701
50.2343
30.8315
27.9711
21.7259

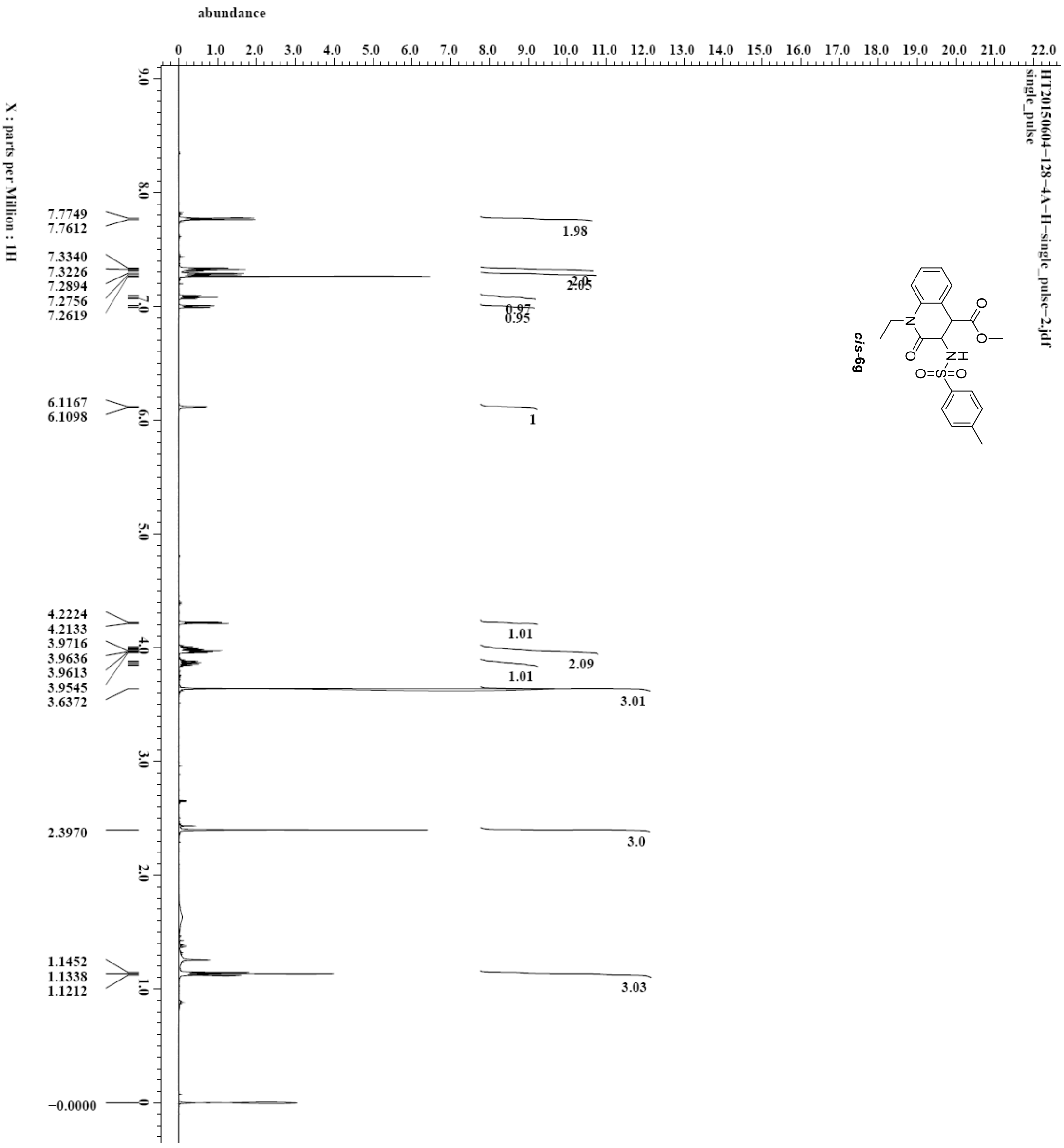
HT20150604−128−4A−H−single_pulse−2.jdf
single_pulse
cis-6g
abundance
X : parts per Million : 1H
7.7749
7.7612
7.3340
7.3226
7.2894
7.2756
7.2619
6.1167
6.1098
4.2224
4.2133
3.9716
3.9636
3.9613
3.9545
3.6372
2.3970
1.1452
1.1338
1.1212
−0.0000
1.98
2.05
0.97
0.95
1
1.01
2.09
1.01
3.01
3.0
3.03

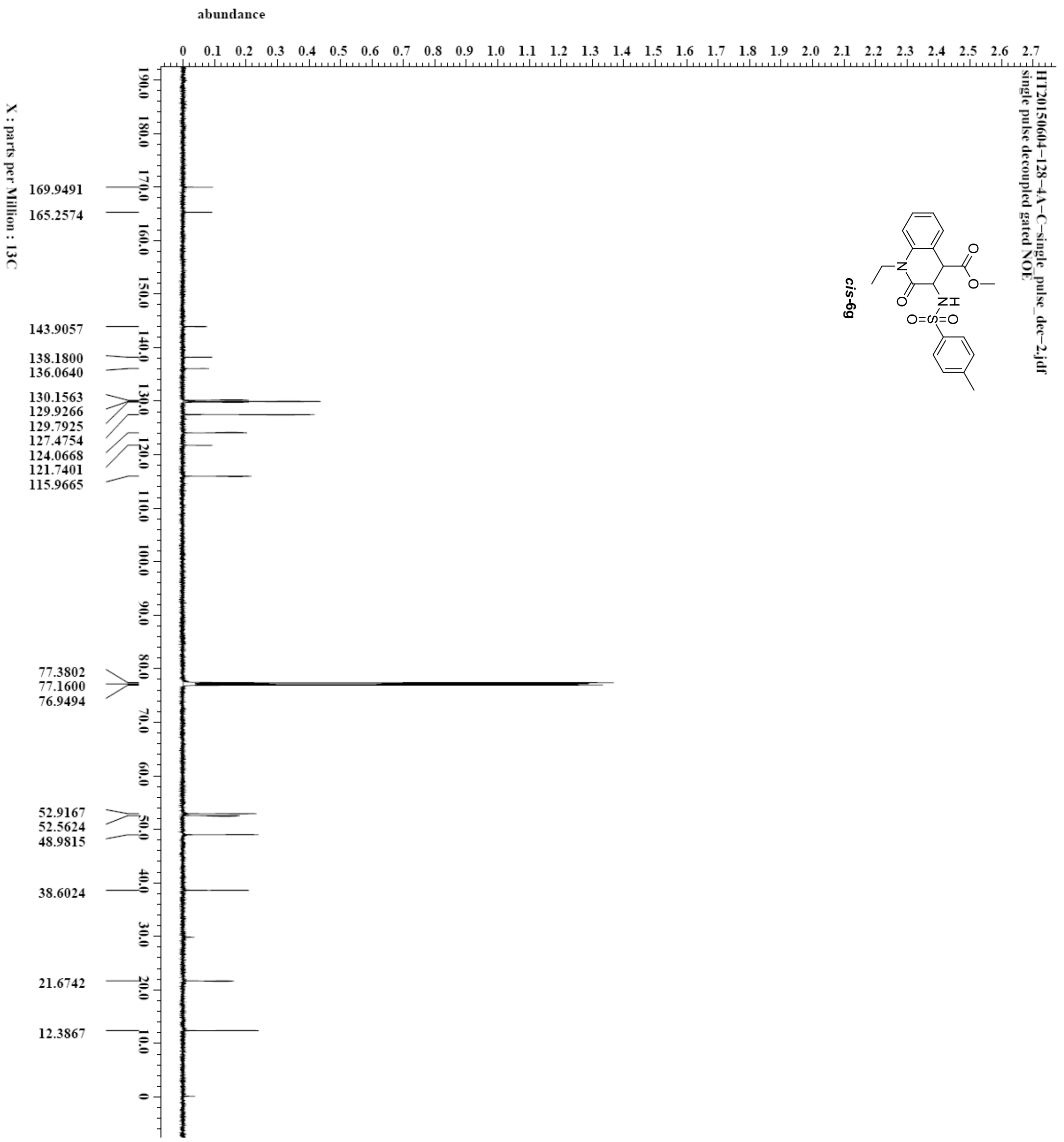
HT20150604−128−4A−C−single_pulse_dec−2.jdf
single pulse decoupled gated NOE
abundance
X : parts per Million : 13C
cis-6g
169.9491
165.2574
143.9057
138.1800
136.0640
130.1563
129.9266
129.7925
127.4754
124.0668
121.7401
115.9665
77.3802
77.1600
76.9494
52.9167
52.5624
48.9815
38.6024
21.6742
12.3867

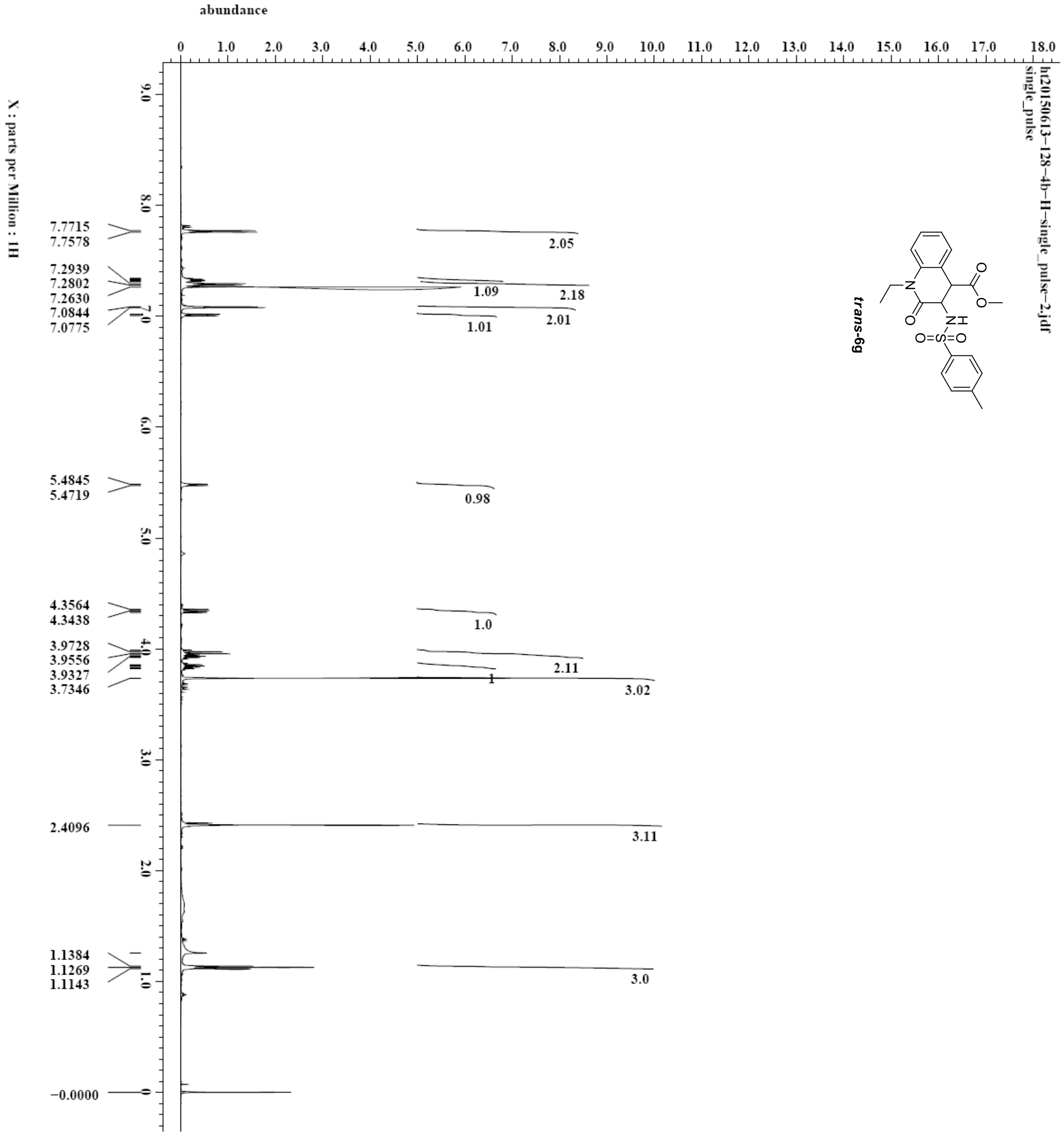

abundance
X : parts per Million : 1H
ht20150613-128-4b-H-single_pulse-2.jdf
single_pulse
7.7715
7.7578
7.2939
7.2802
7.2630
7.0844
7.0775
5.4845
5.4719
4.3564
4.3438
3.9728
3.9556
3.9327
3.7346
2.4096
1.1384
1.1269
1.1143
−0.0000
2.05
1.09
2.18
1.01
2.01
0.98
1.0
2.11
1
3.02
3.11
3.0
trans-6g

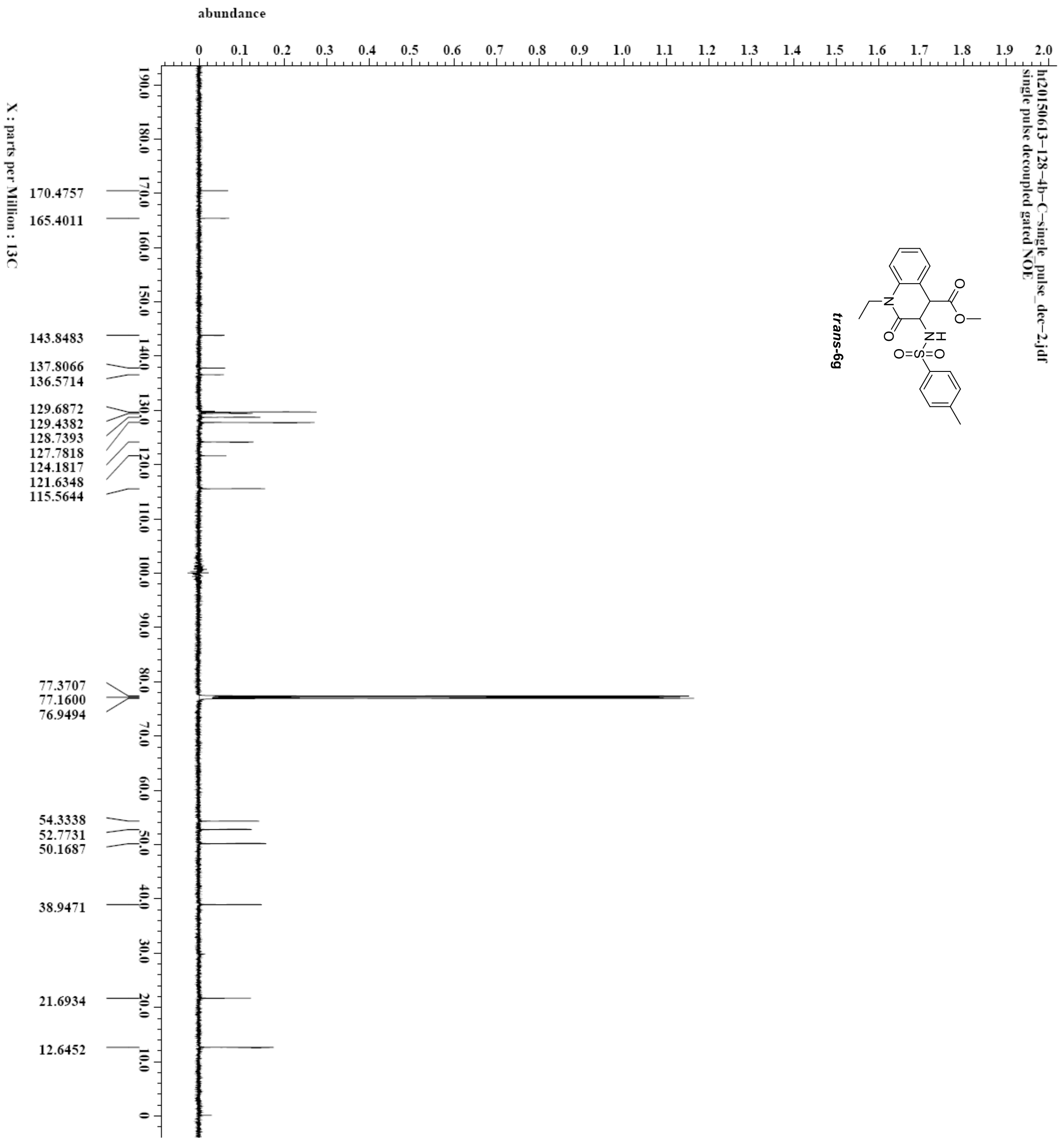
abundance
X : parts per Million : 13C
h20150613-128-4b-C-single_pulse_dec-2.jdf
single pulse decoupled gated NOE
trans-6g
170.4757
165.4011
143.8483
137.8066
136.5714
129.6872
129.4382
128.7393
127.7818
124.1817
121.6348
115.5644
77.3707
77.1600
76.9494
54.3338
52.7731
50.1687
38.9471
21.6934
12.6452

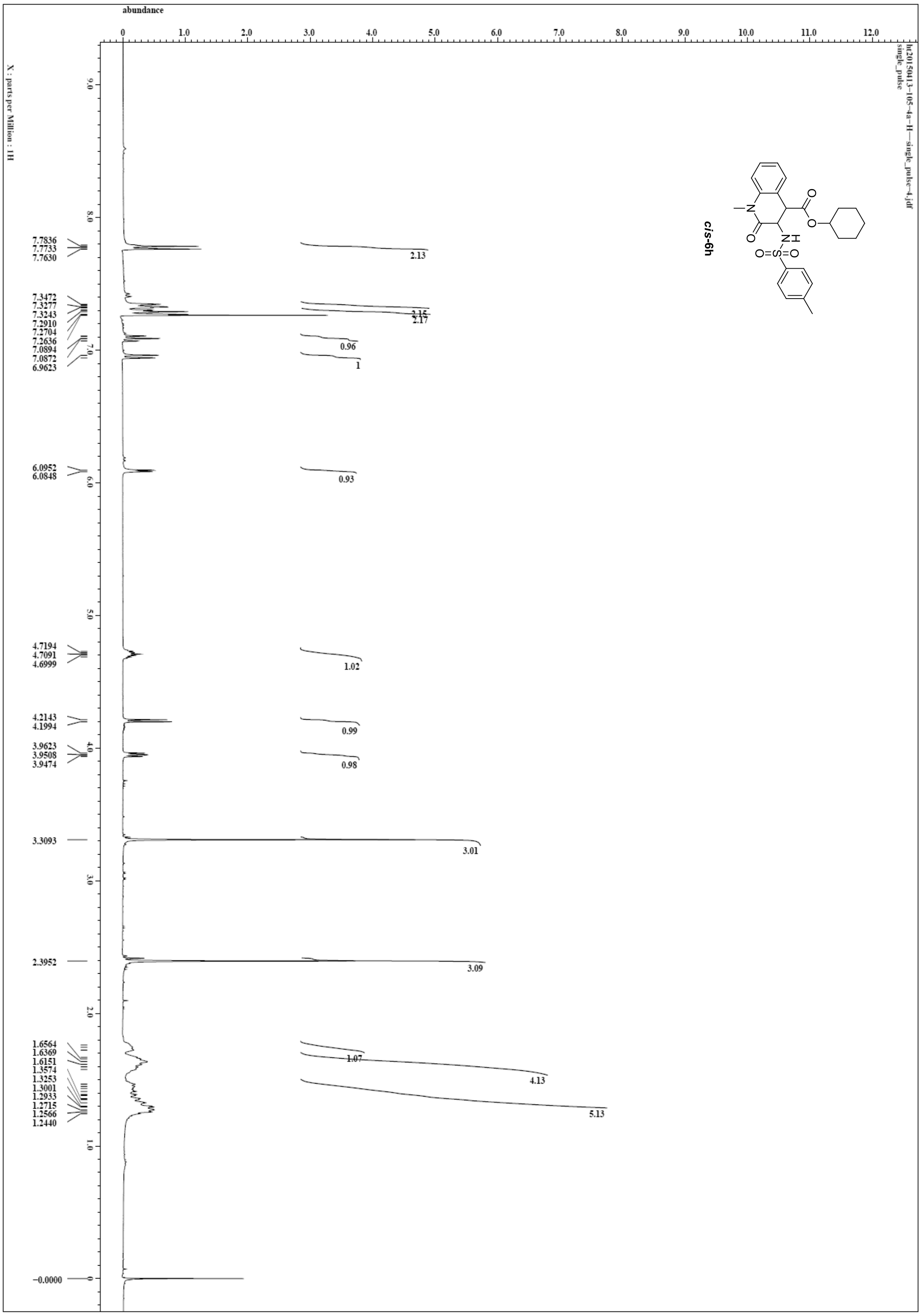

cis-**6h**

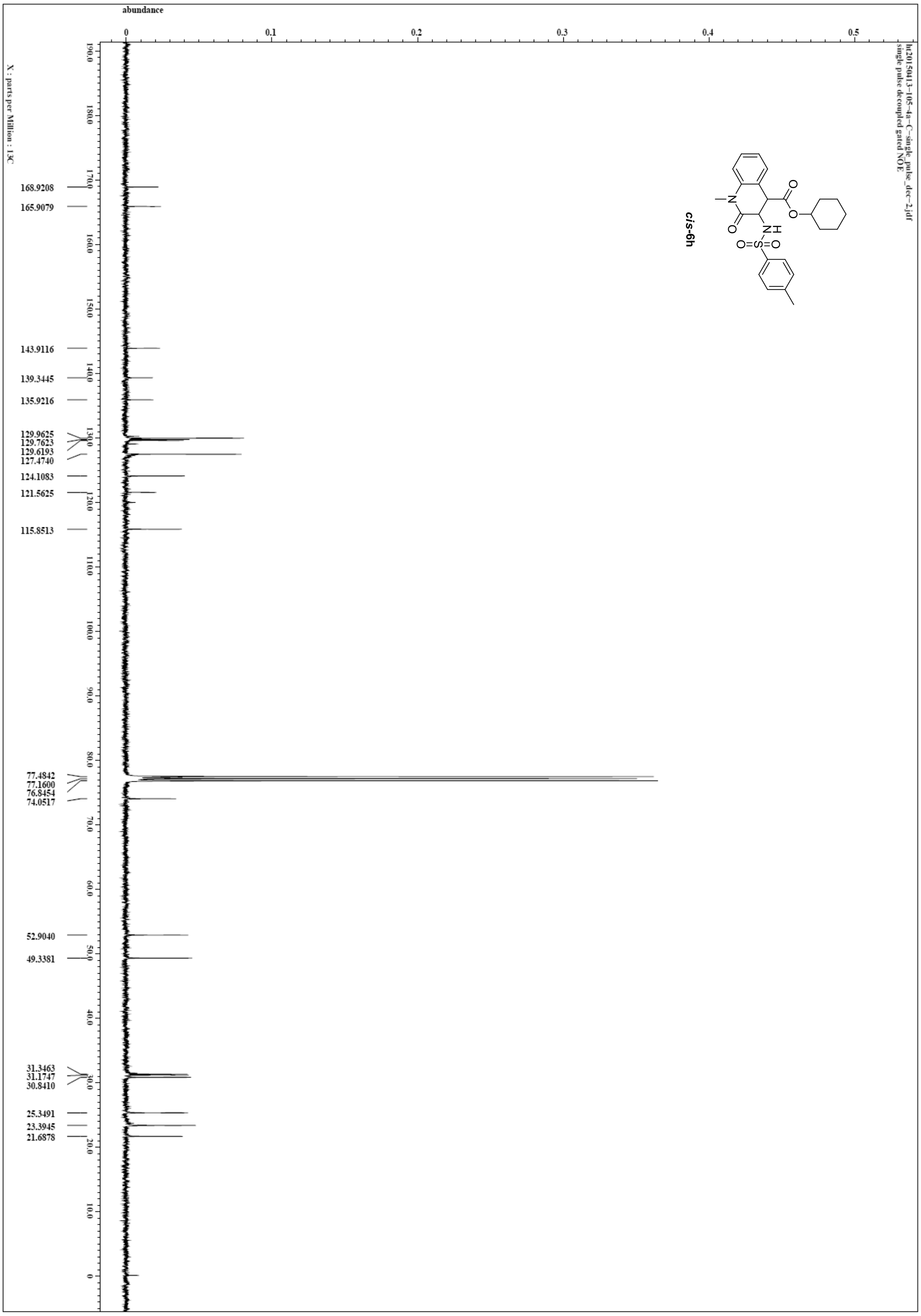
abundance
X : parts per Million : 13C
168.9208
165.9079
143.9116
139.3445
135.9216
129.9625
129.7623
129.6193
127.4740
124.1083
121.5625
115.8513
77.4842
77.1600
76.8454
74.0517
52.9040
49.3381
31.3463
31.1747
30.8410
25.3491
23.3945
21.6878
cis-6h

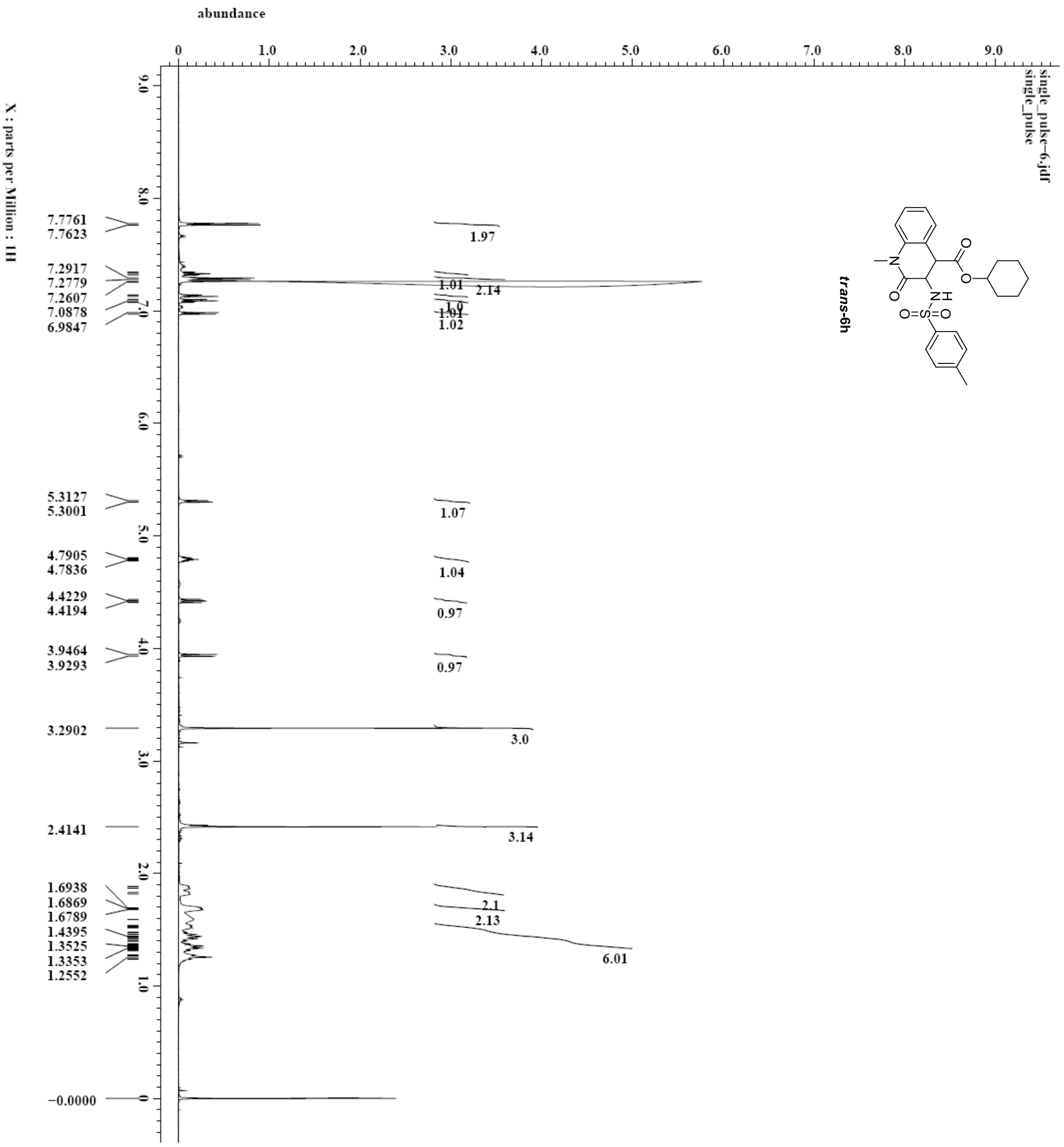
abundance
X : parts per Million : 1H
single_pulse-6.jdf
single_pulse
trans-6h
7.7761
7.7623
7.2917
7.2779
7.2607
7.0878
6.9847
5.3127
5.3001
4.7905
4.7836
4.4229
4.4194
3.9464
3.9293
3.2902
2.4141
1.6938
1.6869
1.6789
1.4395
1.3525
1.3353
1.2552
-0.0000

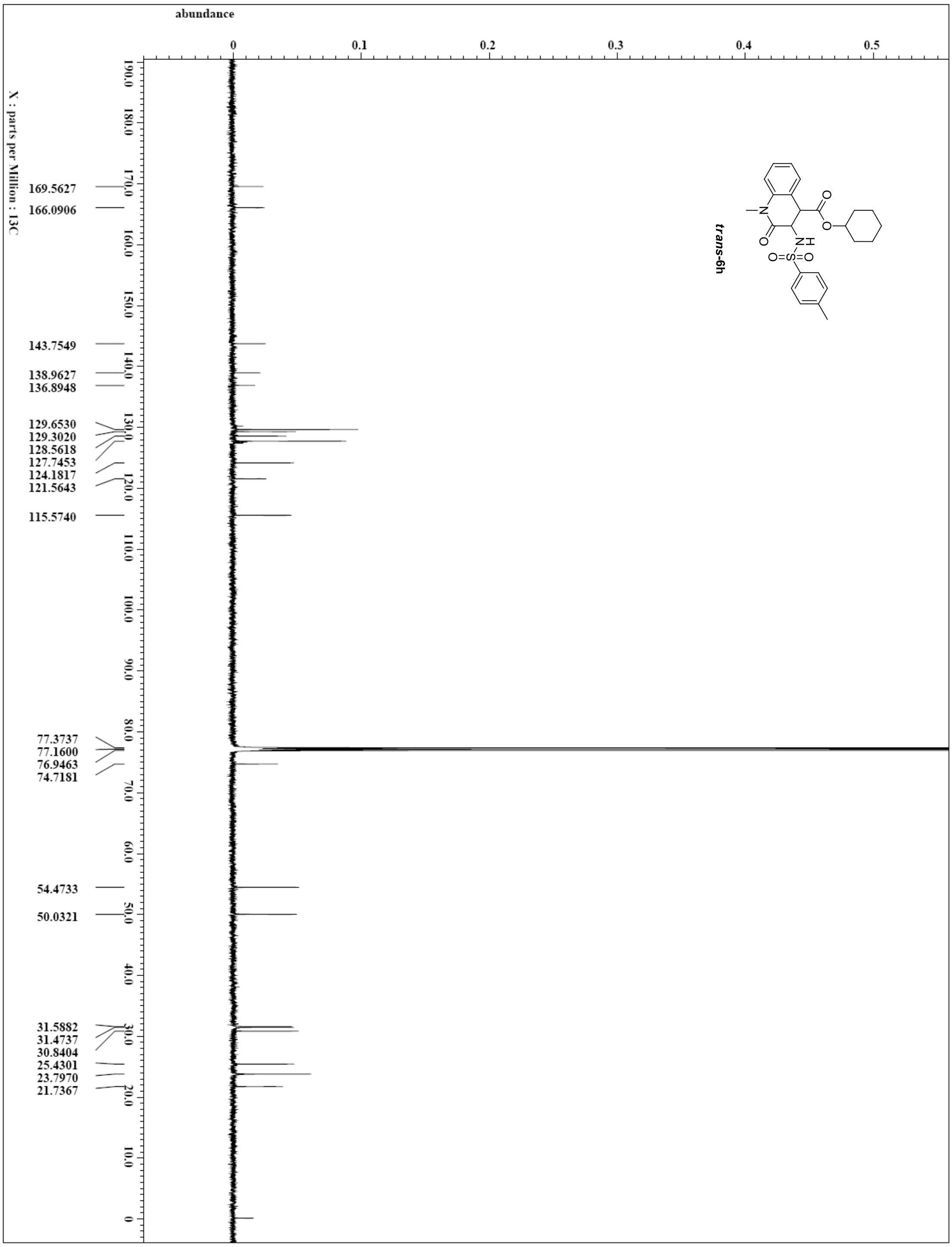
abundance
0
0.1
0.2
0.3
0.4
0.5
X : parts per Million : 13C
169.5627
166.0906
143.7549
138.9627
136.8948
129.6530
129.3020
128.5618
127.7453
124.1817
121.5643
115.5740
77.3737
77.1600
76.9463
74.7181
54.4733
50.0321
31.5882
31.4737
30.8404
25.4301
23.7970
21.7367
trans-6h

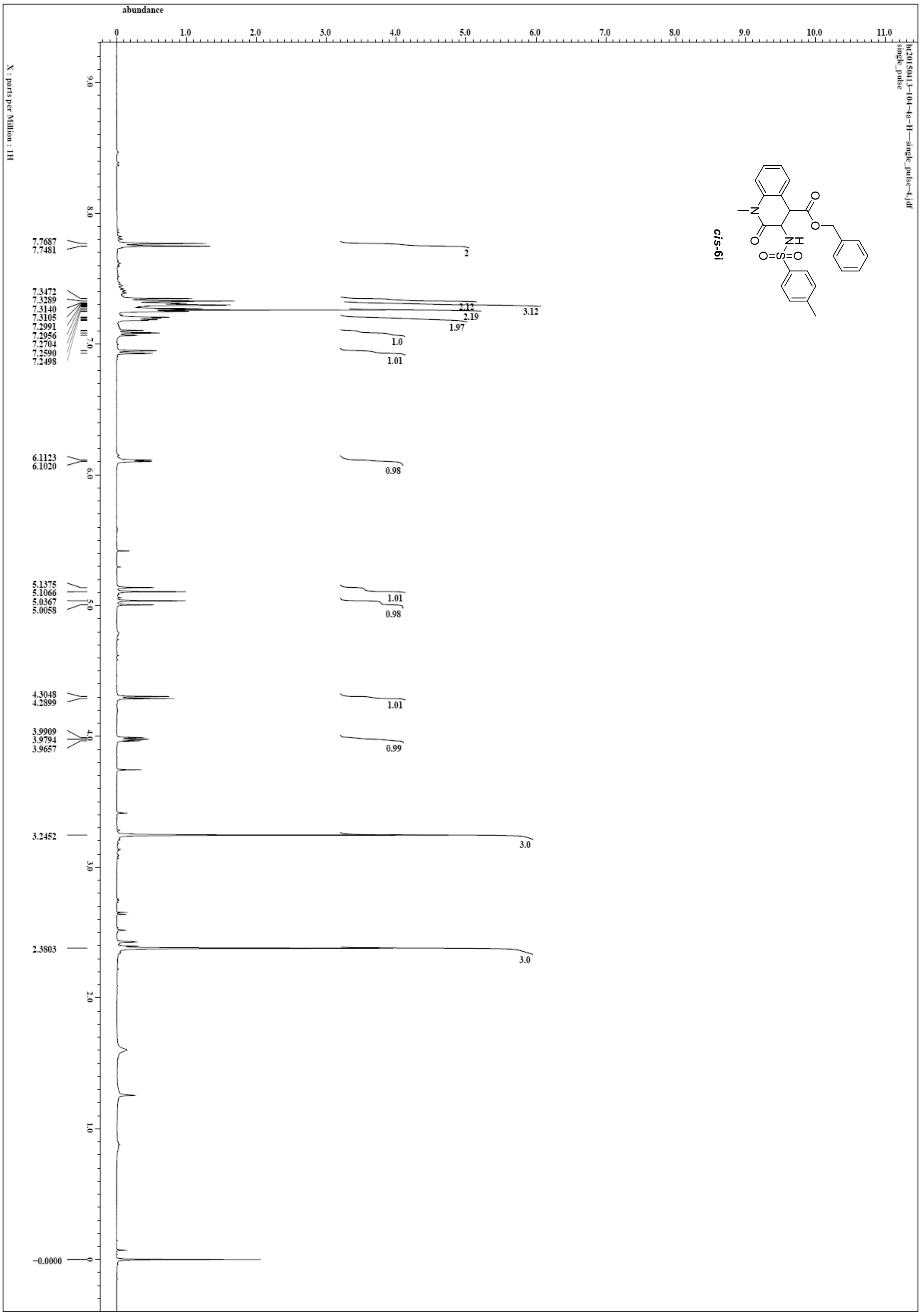

abundance
X : parts per Million : 1H
7.7687
7.7481
7.3472
7.3289
7.3140
7.3105
7.2991
7.2956
7.2704
7.2590
7.2498
6.1123
6.1020
5.1375
5.1066
5.0367
5.0058
4.3048
4.2899
3.9909
3.9794
3.9657
3.2452
2.3803
-0.0000
cis-6i

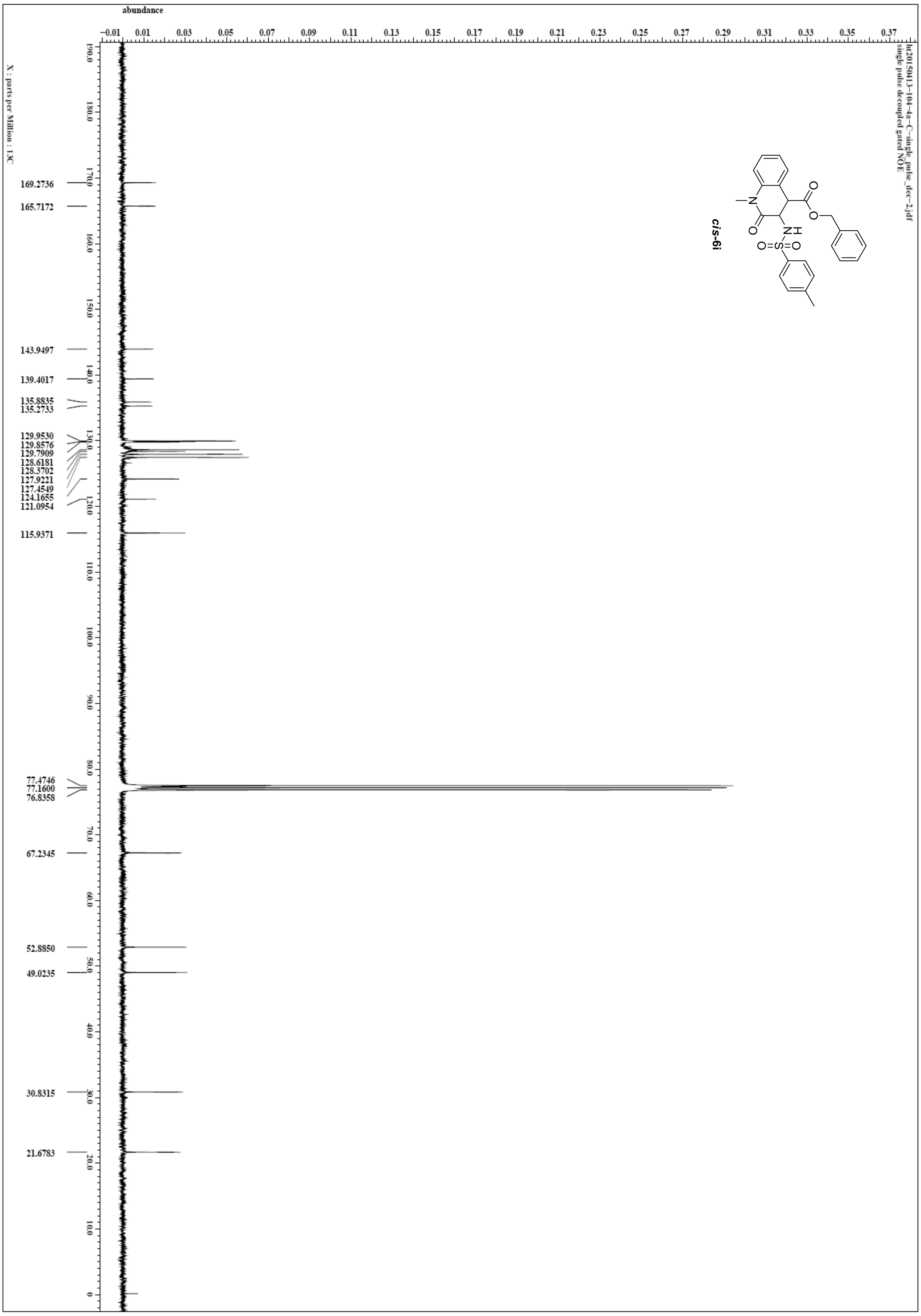
abundance
X : parts per Million : 13C
169.2736
165.7172
143.9497
139.4017
135.8835
135.2733
129.9530
129.8576
129.7909
128.6181
128.3702
127.9221
127.4549
124.1655
121.0954
115.9371
77.4746
77.1600
76.8358
67.2345
52.8850
49.0235
30.8315
21.6783
cis-6i

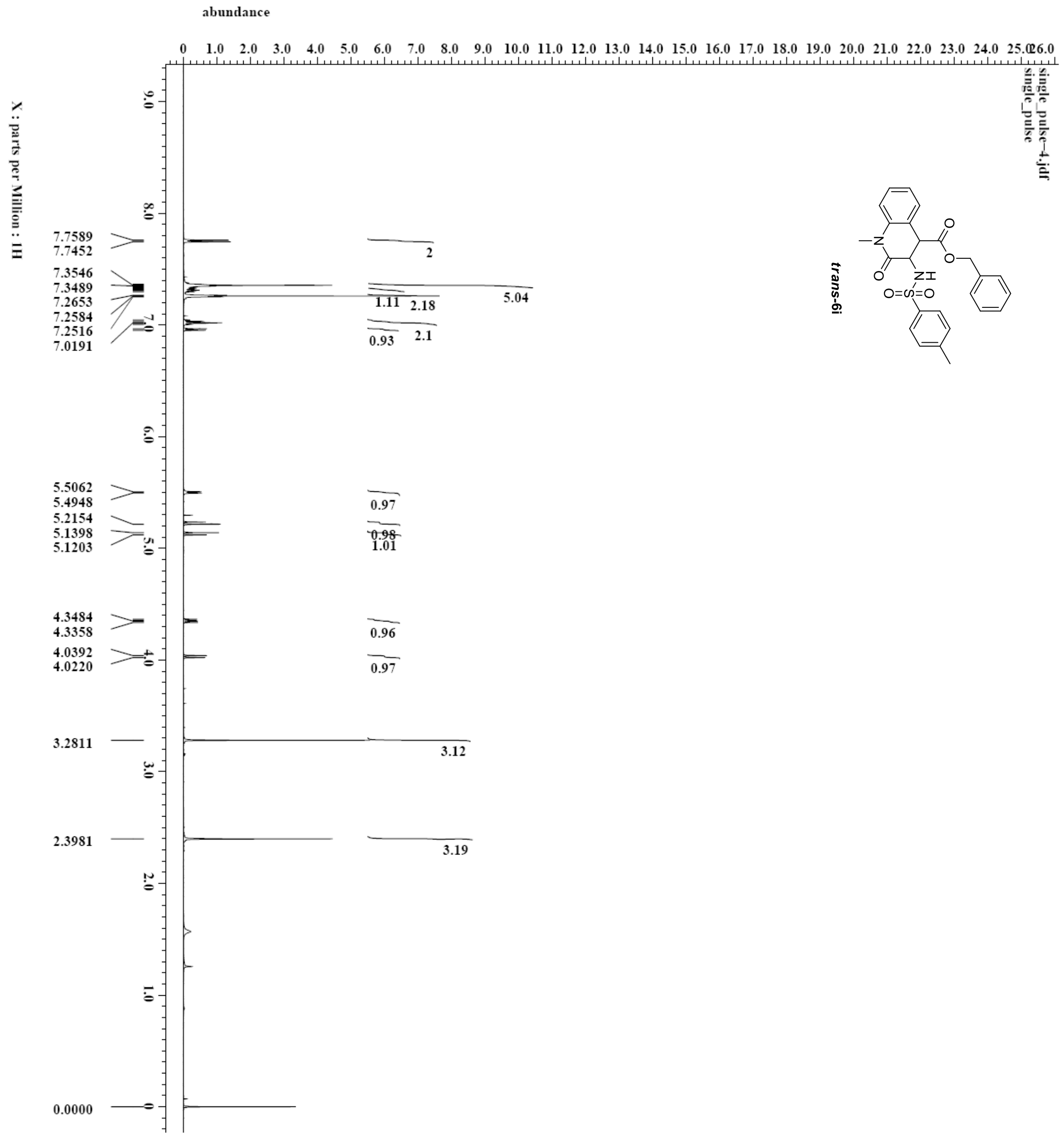
abundance
X : parts per Million : 1H
single_pulse-4.jdf
single_pulse
trans-6i
7.7589
7.7452
7.3546
7.3489
7.2653
7.2584
7.2516
7.0191
5.5062
5.4948
5.2154
5.1398
5.1203
4.3484
4.3358
4.0392
4.0220
3.2811
2.3981
0.0000
2
1.11
2.18
5.04
0.93
2.1
0.97
0.98
1.01
0.96
0.97
3.12
3.19

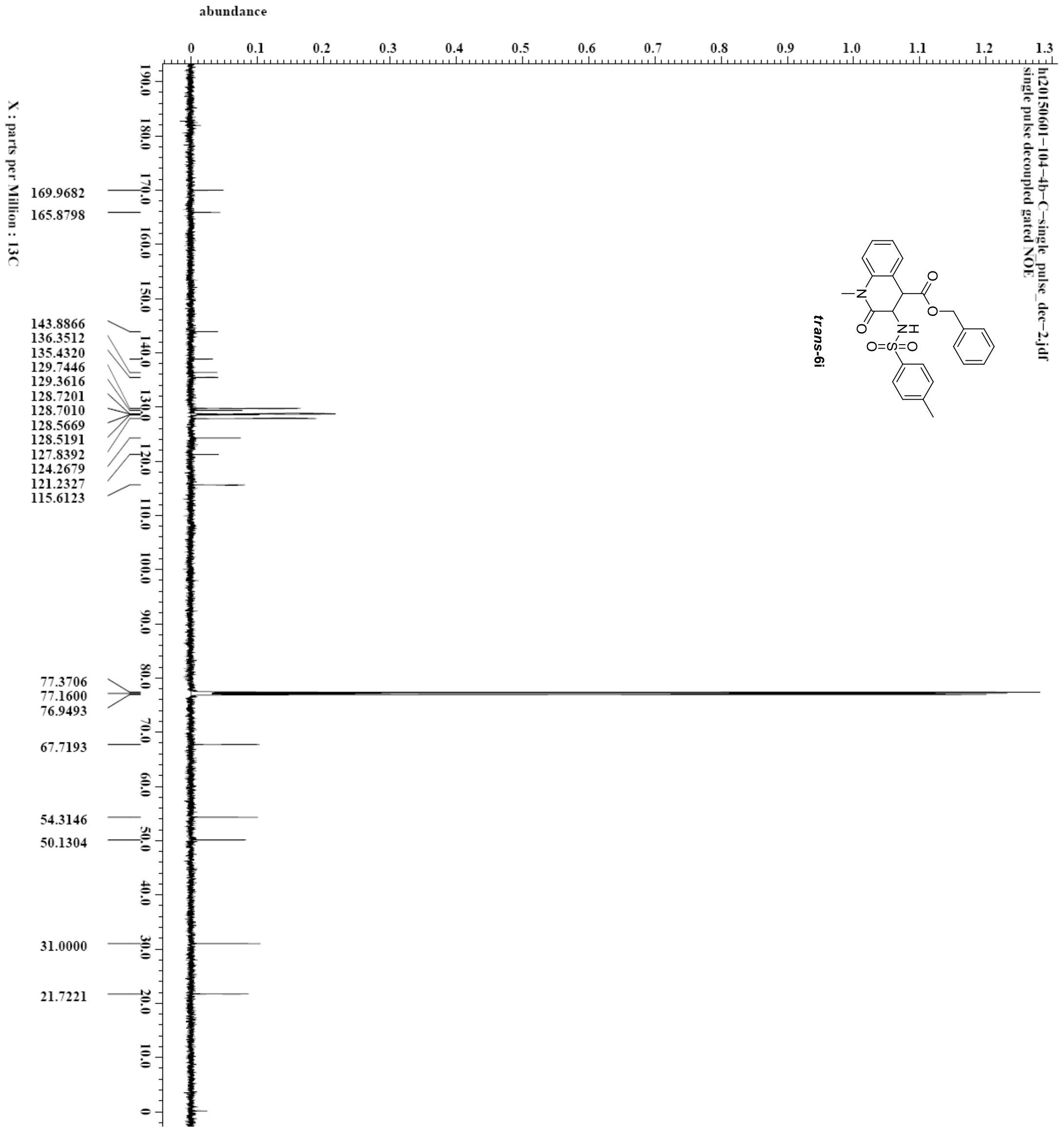

abundance
X : parts per Million : 13C
169.9682
165.8798
143.8866
136.3512
135.4320
129.7446
129.3616
128.7201
128.7010
128.5669
128.5191
127.8392
124.2679
121.2327
115.6123
77.3706
77.1600
76.9493
67.7193
54.3146
50.1304
31.0000
21.7221
hl20150601-104-4b-C-single_pulse_dec-2.jdf
single pulse decoupled gated NOE
trans-6i

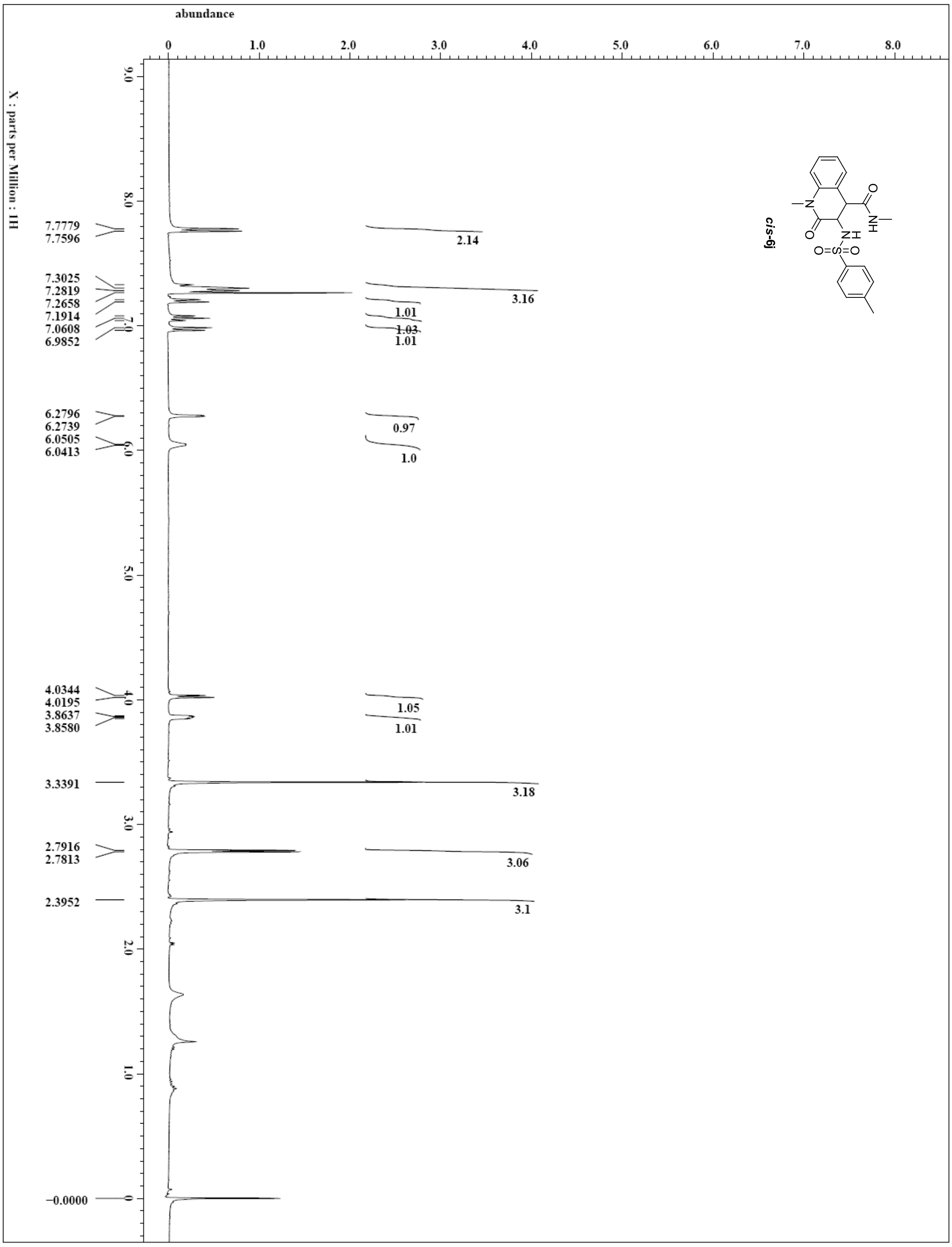
abundance
0
1.0
2.0
3.0
4.0
5.0
6.0
7.0
8.0
X : parts per Million : 1H
9.0
8.0
7.0
6.0
5.0
4.0
3.0
2.0
1.0
0
7.7779
7.7596
7.3025
7.2819
7.2658
7.1914
7.0608
6.9852
6.2796
6.2739
6.0505
6.0413
4.0344
4.0195
3.8637
3.8580
3.3391
2.7916
2.7813
2.3952
-0.0000
2.14
3.16
1.01
1.03
1.01
0.97
1.0
1.05
1.01
3.18
3.06
3.1
cis-6j

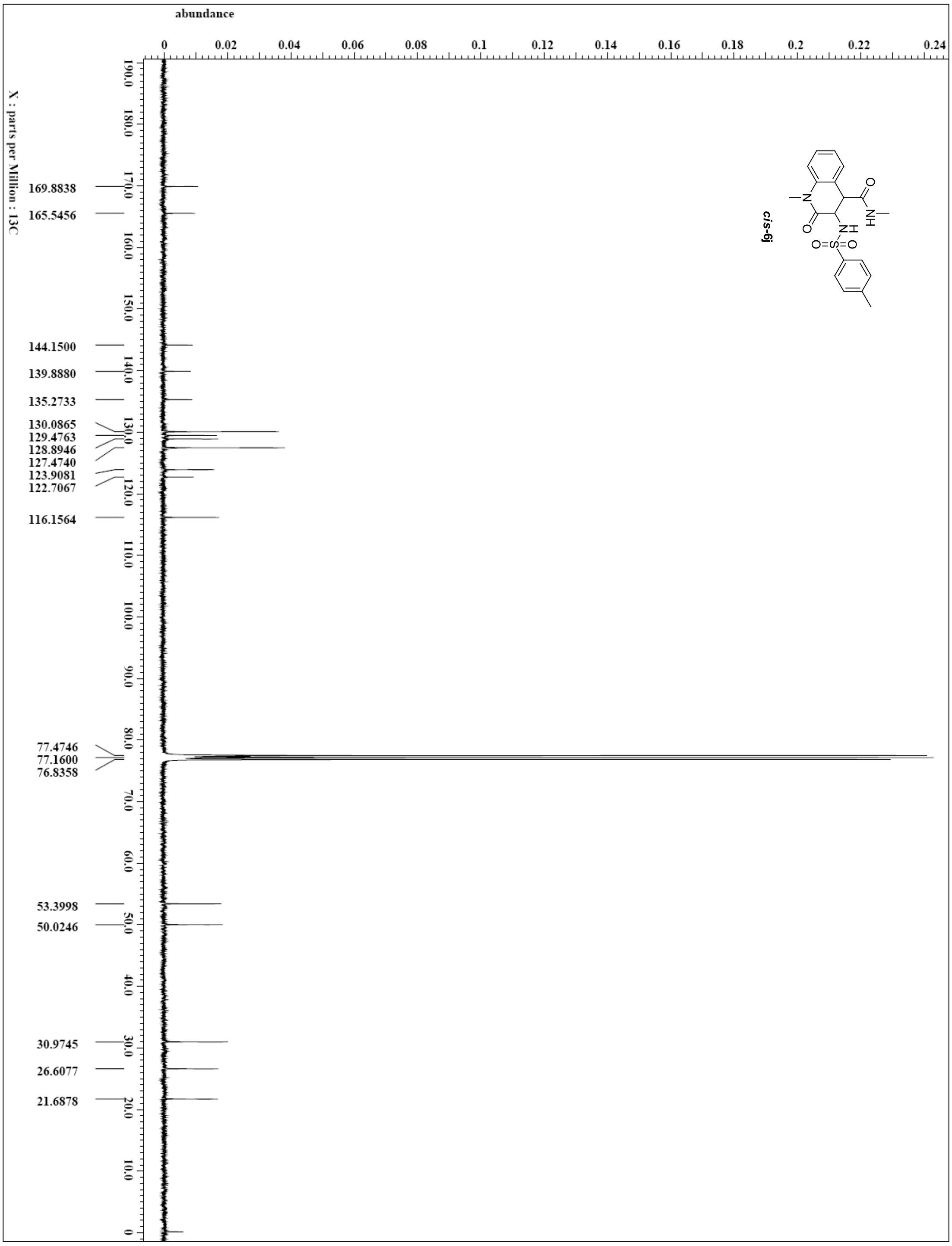
abundance
X : parts per Million : 13C
cis-6j
169.8838
165.5456
144.1500
139.8880
135.2733
130.0865
129.4763
128.8946
127.4740
123.9081
122.7067
116.1564
77.4746
77.1600
76.8358
53.3998
50.0246
30.9745
26.6077
21.6878

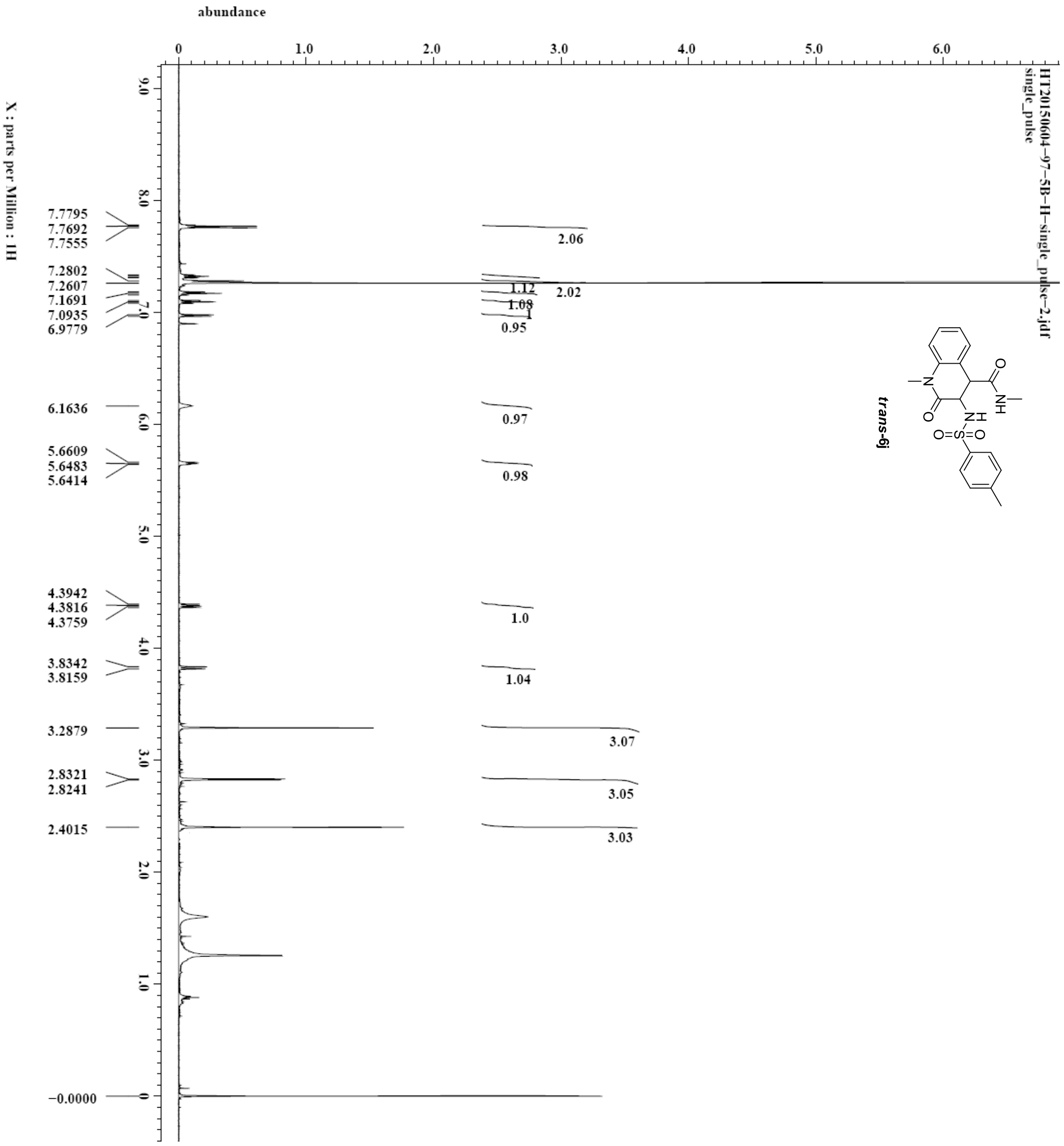

abundance
0
1.0
2.0
3.0
4.0
5.0
6.0
HT20150604-97-5B-H-single_pulse-2.jdf
single_pulse
X : parts per Million : 1H
9.0
8.0
7.0
6.0
5.0
4.0
3.0
2.0
1.0
0
7.7795
7.7692
7.7555
7.2802
7.2607
7.1691
7.0935
6.9779
6.1636
5.6609
5.6483
5.6414
4.3942
4.3816
4.3759
3.8342
3.8159
3.2879
2.8321
2.8241
2.4015
-0.0000
2.06
1.12
2.02
1.08
1
0.95
0.97
0.98
1.0
1.04
3.07
3.05
3.03
trans-6j

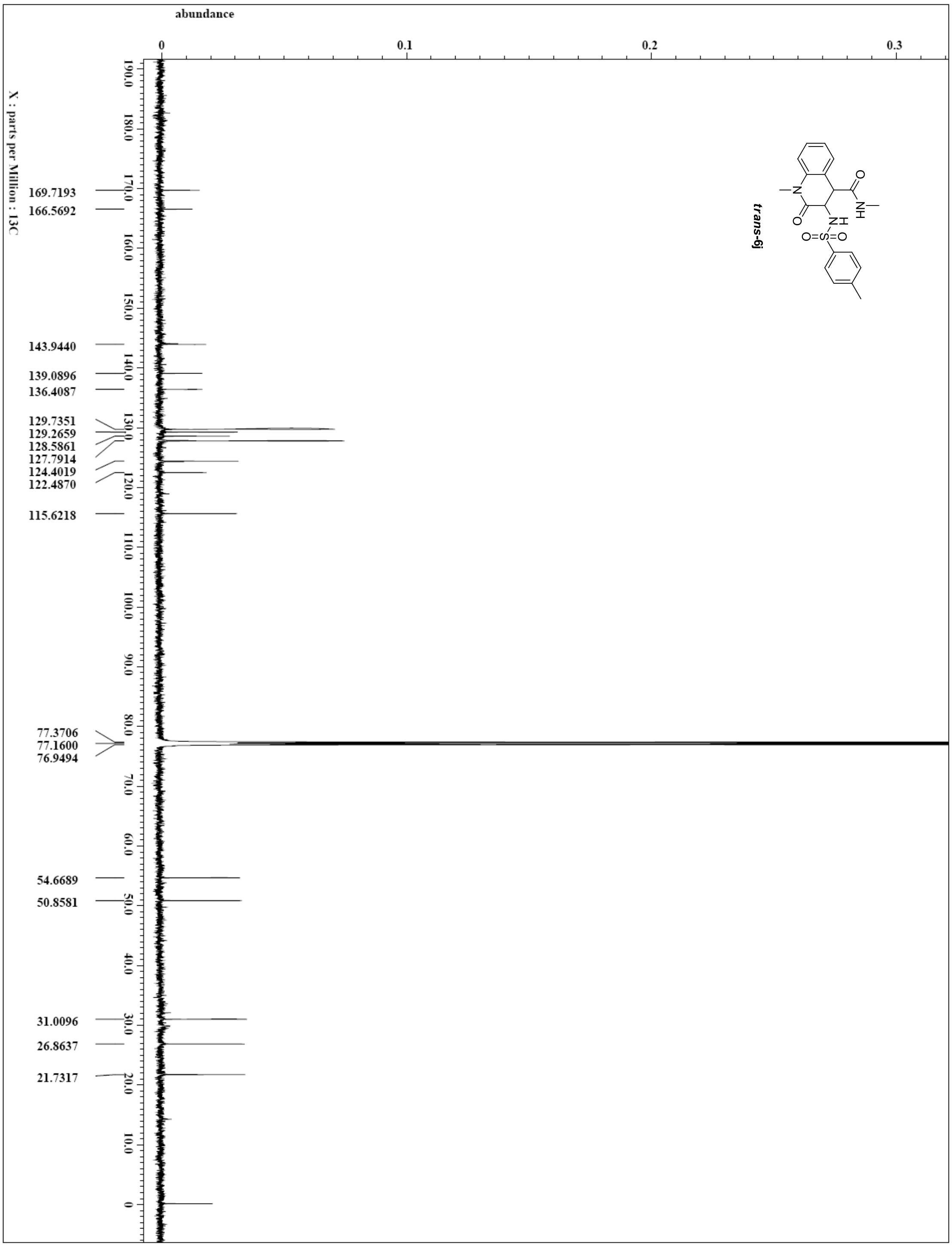
abundance
0
0.1
0.2
0.3
X : parts per Million : 13C
trans-6j
169.7193
166.5692
143.9440
139.0896
136.4087
129.7351
129.2659
128.5861
127.7914
124.4019
122.4870
115.6218
77.3706
77.1600
76.9494
54.6689
50.8581
31.0096
26.8637
21.7317

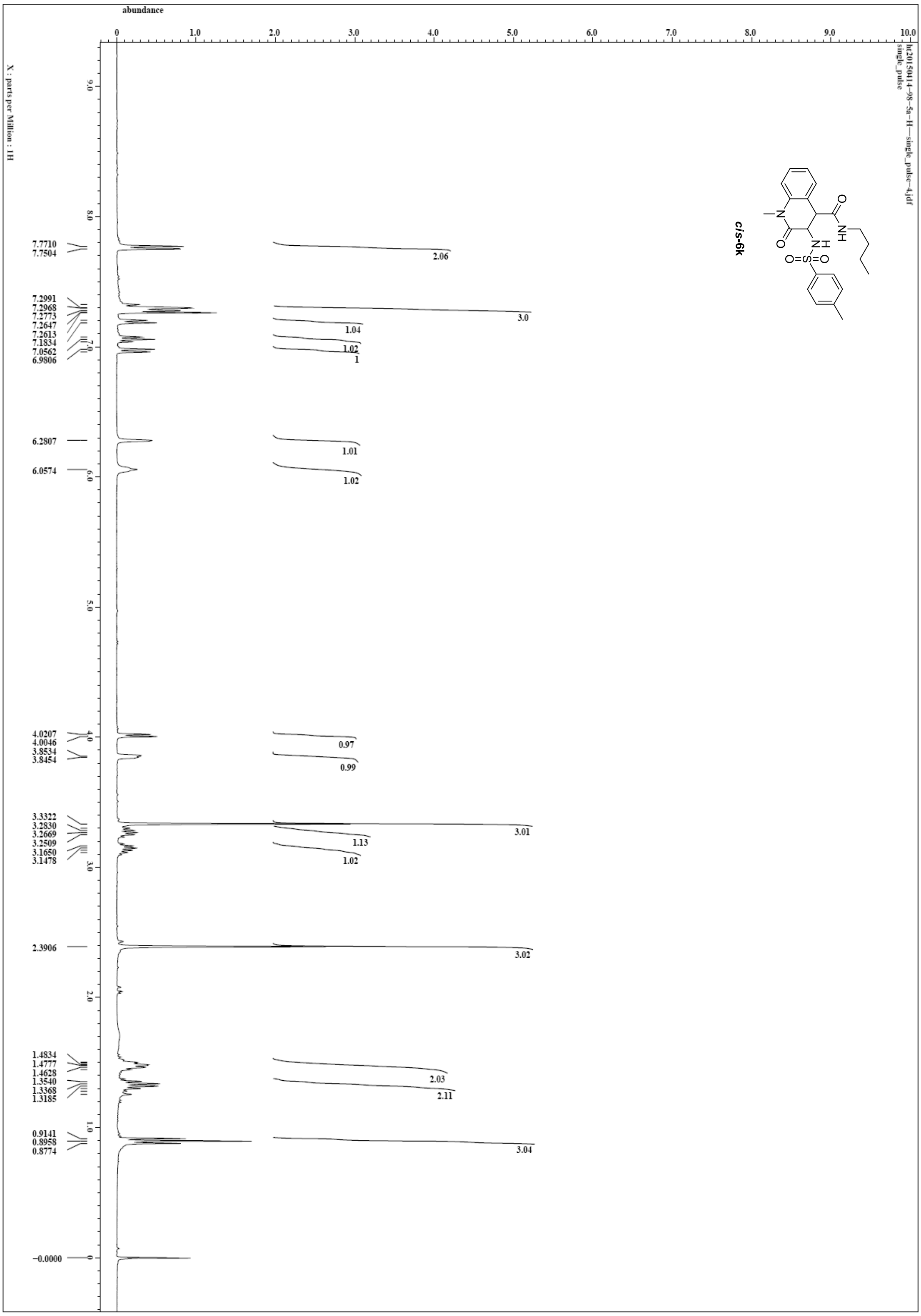
cis-6k

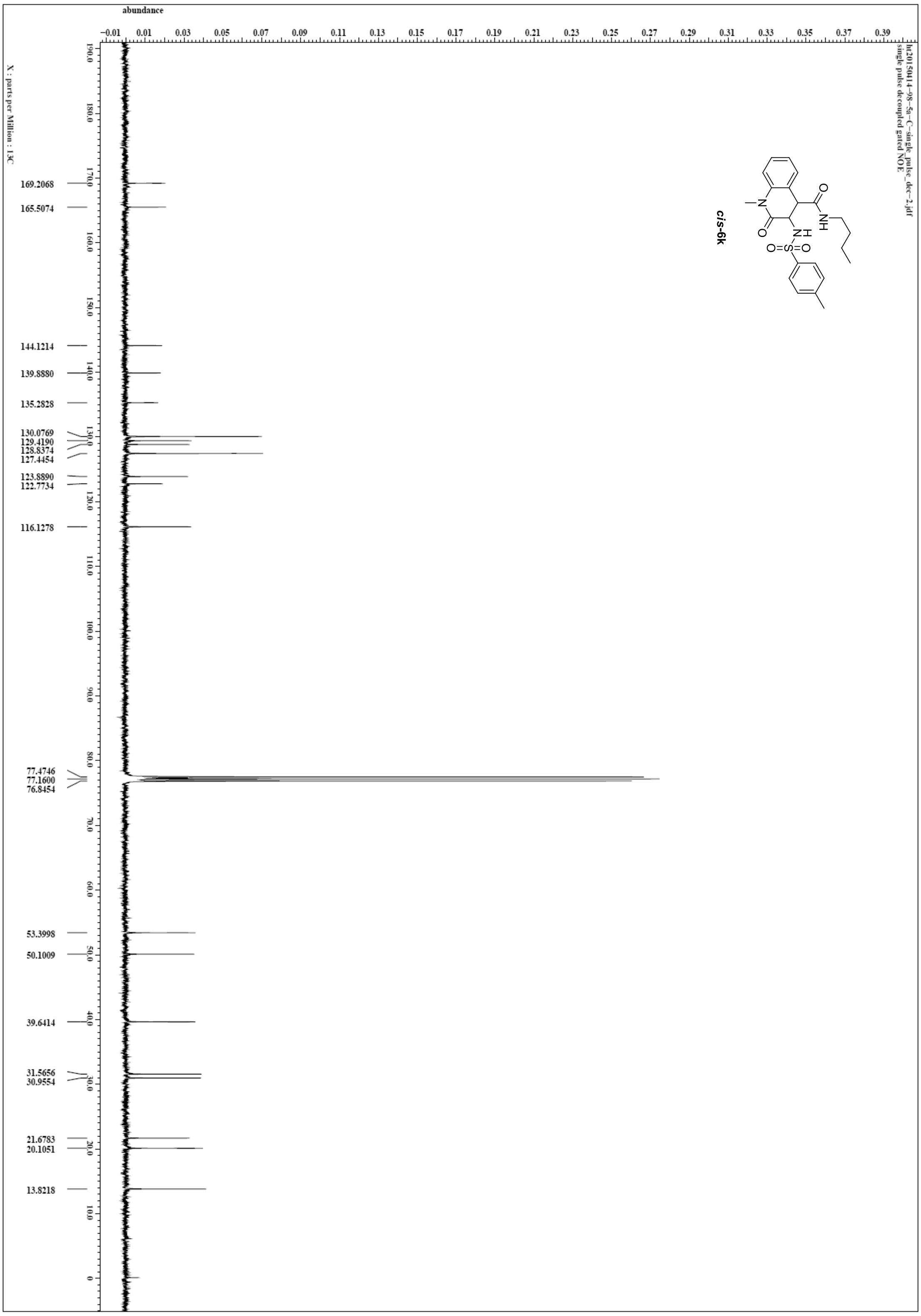

abundance
X : parts per Million : 13C
169.2068
165.5074
144.1214
139.8880
135.2828
130.0769
129.4190
128.8374
127.4454
123.8890
122.7734
116.1278
77.4746
77.1600
76.8454
53.3998
50.1009
39.6414
31.5656
30.9554
21.6783
20.1051
13.8218
single pulse decoupled gated NOE
cis-6k

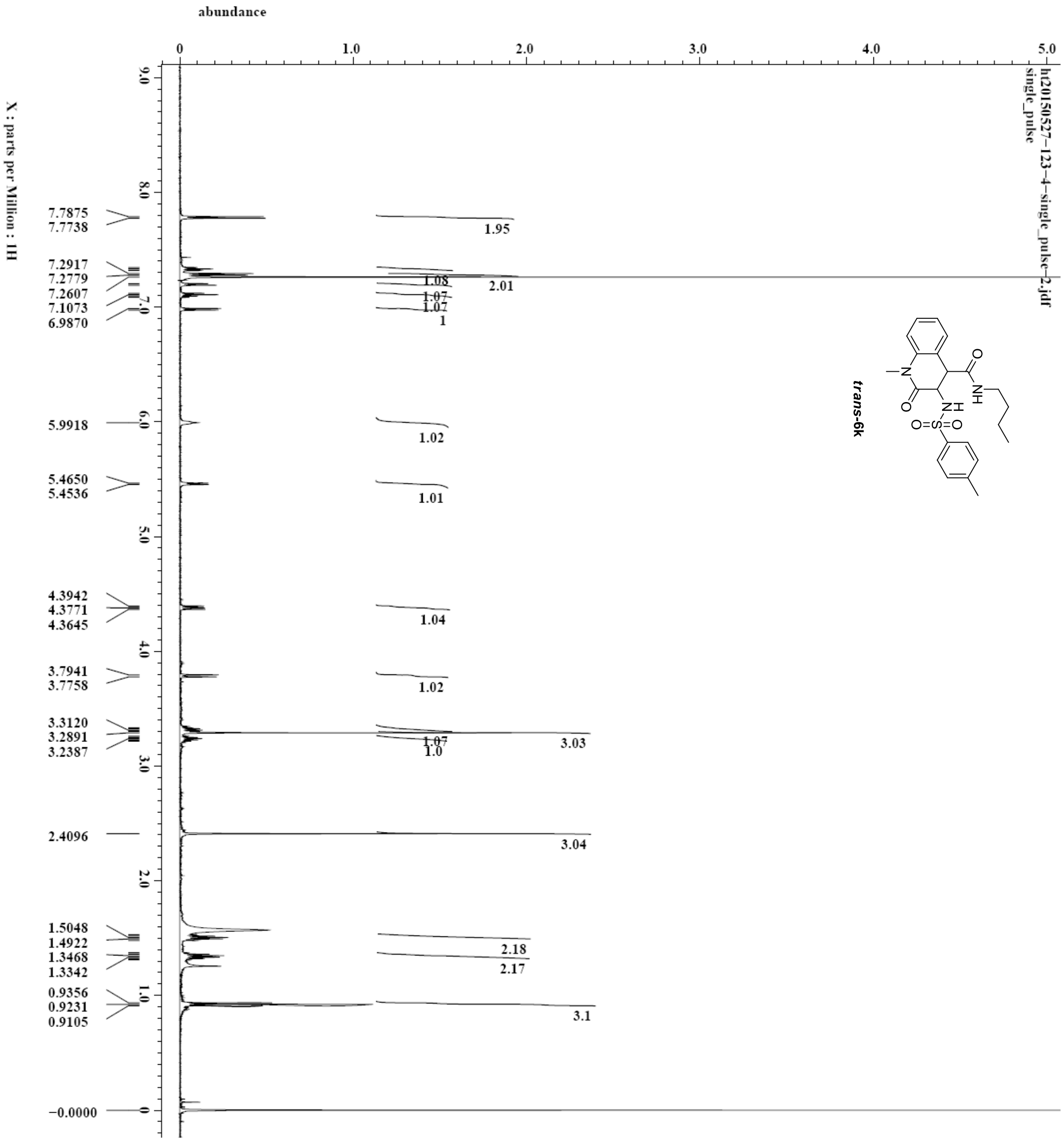
abundance
X : parts per Million : 1H
h20150527-123-4-single_pulse-2.jdf
single_pulse
trans-6k
7.7875
7.7738
7.2917
7.2779
7.2607
7.1073
6.9870
5.9918
5.4650
5.4536
4.3942
4.3771
4.3645
3.7941
3.7758
3.3120
3.2891
3.2387
2.4096
1.5048
1.4922
1.3468
1.3342
0.9356
0.9231
0.9105
−0.0000
1.95
1.08
2.01
1.07
1.07
1
1.02
1.01
1.04
1.02
1.07
1.0
3.03
3.04
2.18
2.17
3.1

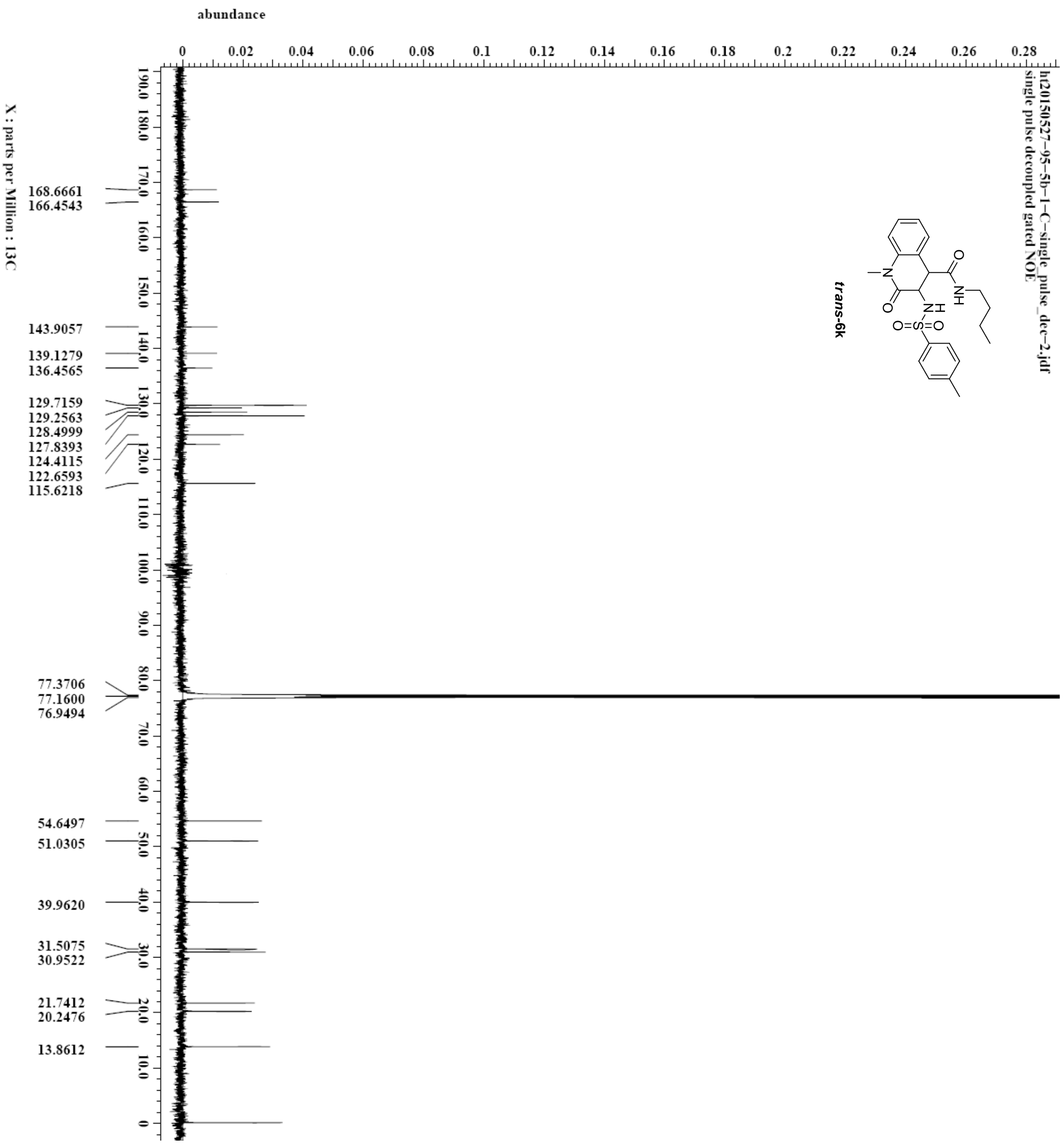

abundance
X : parts per Million : 13C
h20150527--95--5b--1--C--single_pulse_dec--2.jdf
single pulse decoupled gated NOE
trans-6k
168.6661
166.4543
143.9057
139.1279
136.4565
129.7159
129.2563
128.4999
127.8393
124.4115
122.6593
115.6218
77.3706
77.1600
76.9494
54.6497
51.0305
39.9620
31.5075
30.9522
21.7412
20.2476
13.8612

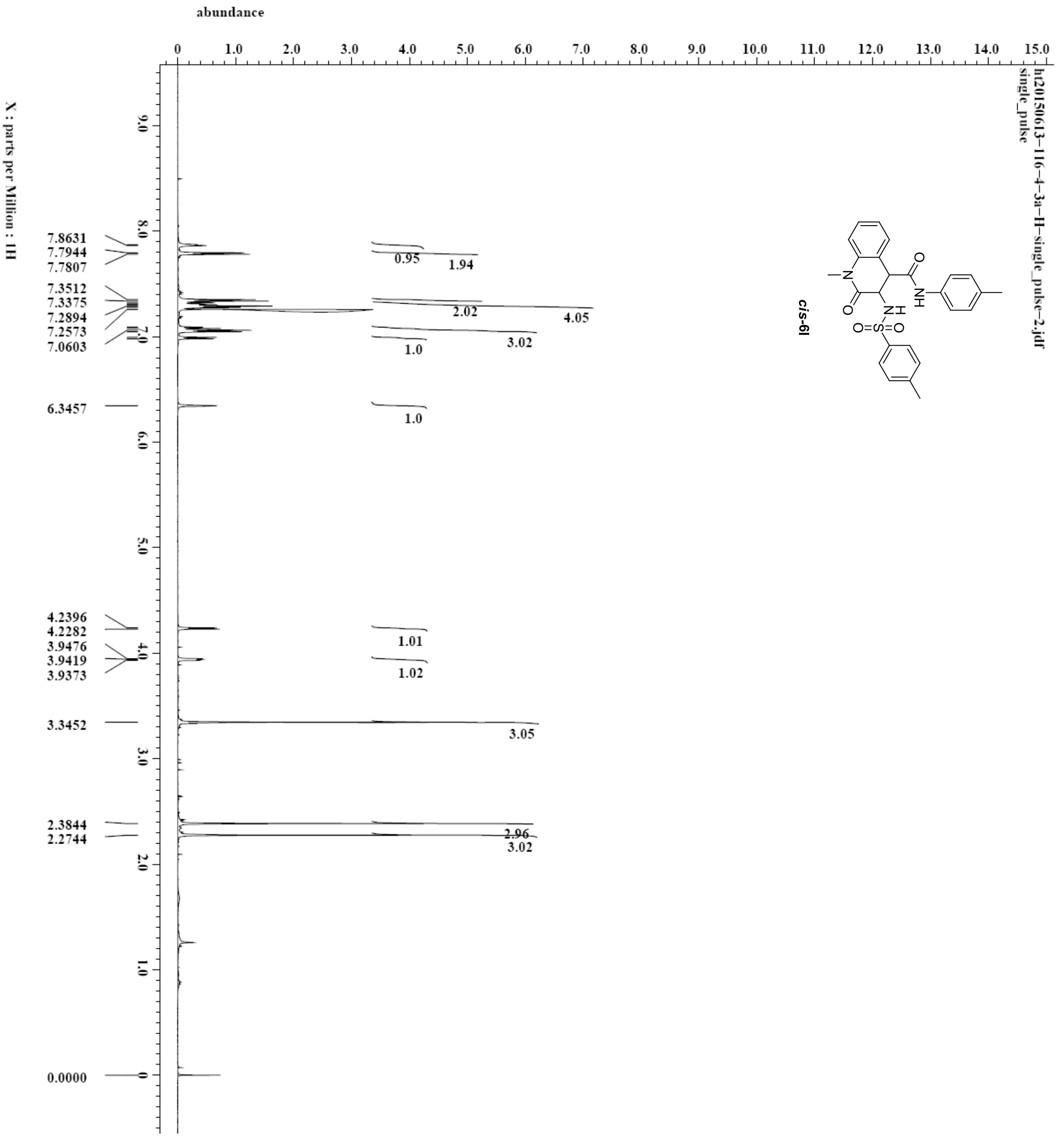

h20150613-116-4-3a-H-single_pulse-2.jdf
single_pulse
abundance
X : parts per Million : 1H
cis-6l
7.8631
7.7944
7.7807
7.3512
7.3375
7.2894
7.2573
7.0603
6.3457
4.2396
4.2282
3.9476
3.9419
3.9373
3.3452
2.3844
2.2744
0.0000
0.95
1.94
2.02
4.05
1.0
3.02
1.0
1.01
1.02
3.05
2.96
3.02

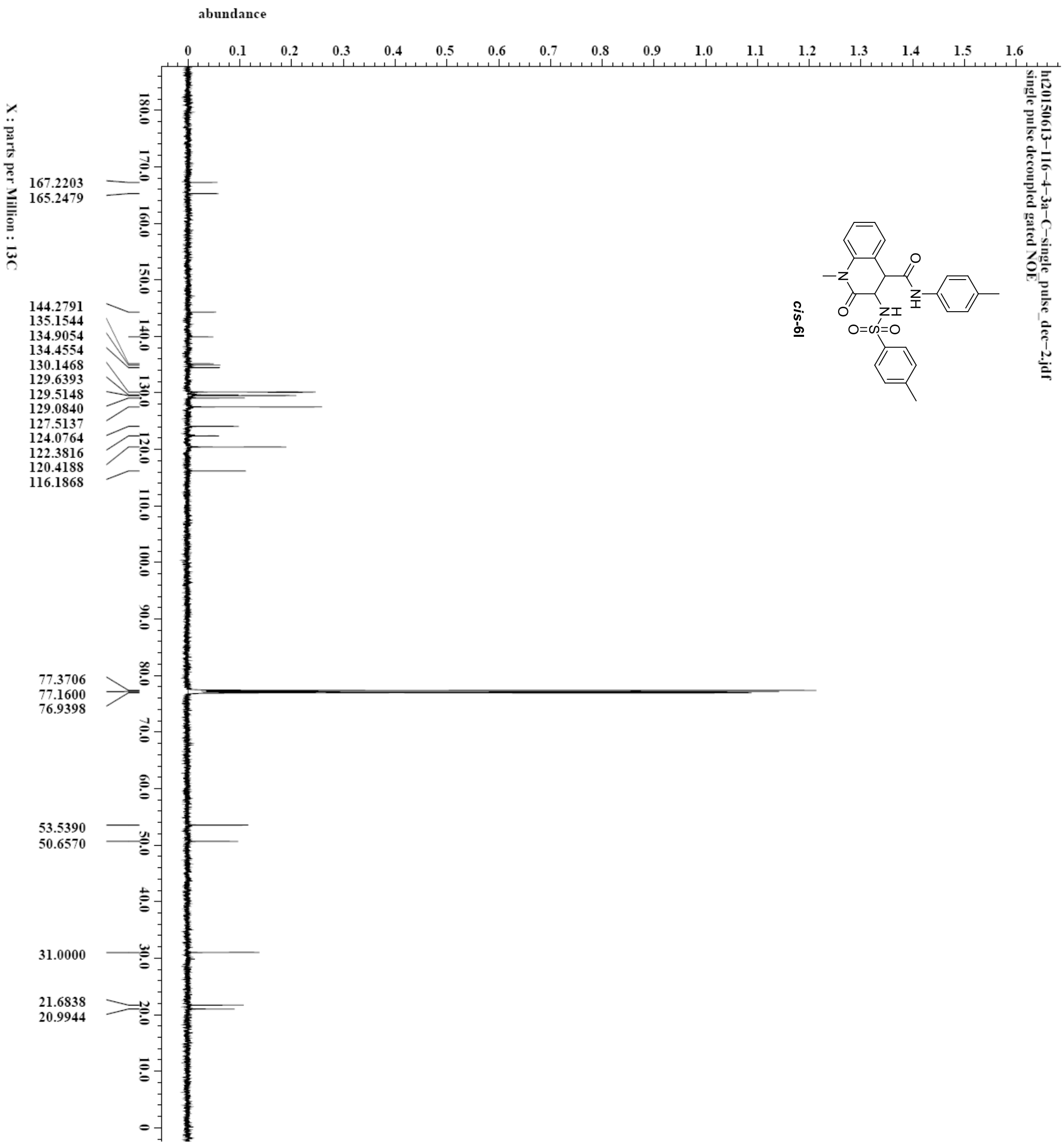
abundance
h20150613-116-4-3a-C-single_pulse_dec-2.jdf
single pulse decoupled gated NOE
cis-6l
X : parts per Million : 13C
167.2203
165.2479
144.2791
135.1544
134.9054
134.4554
130.1468
129.6393
129.5148
129.0840
127.5137
124.0764
122.3816
120.4188
116.1868
77.3706
77.1600
76.9398
53.5390
50.6570
31.0000
21.6838
20.9944

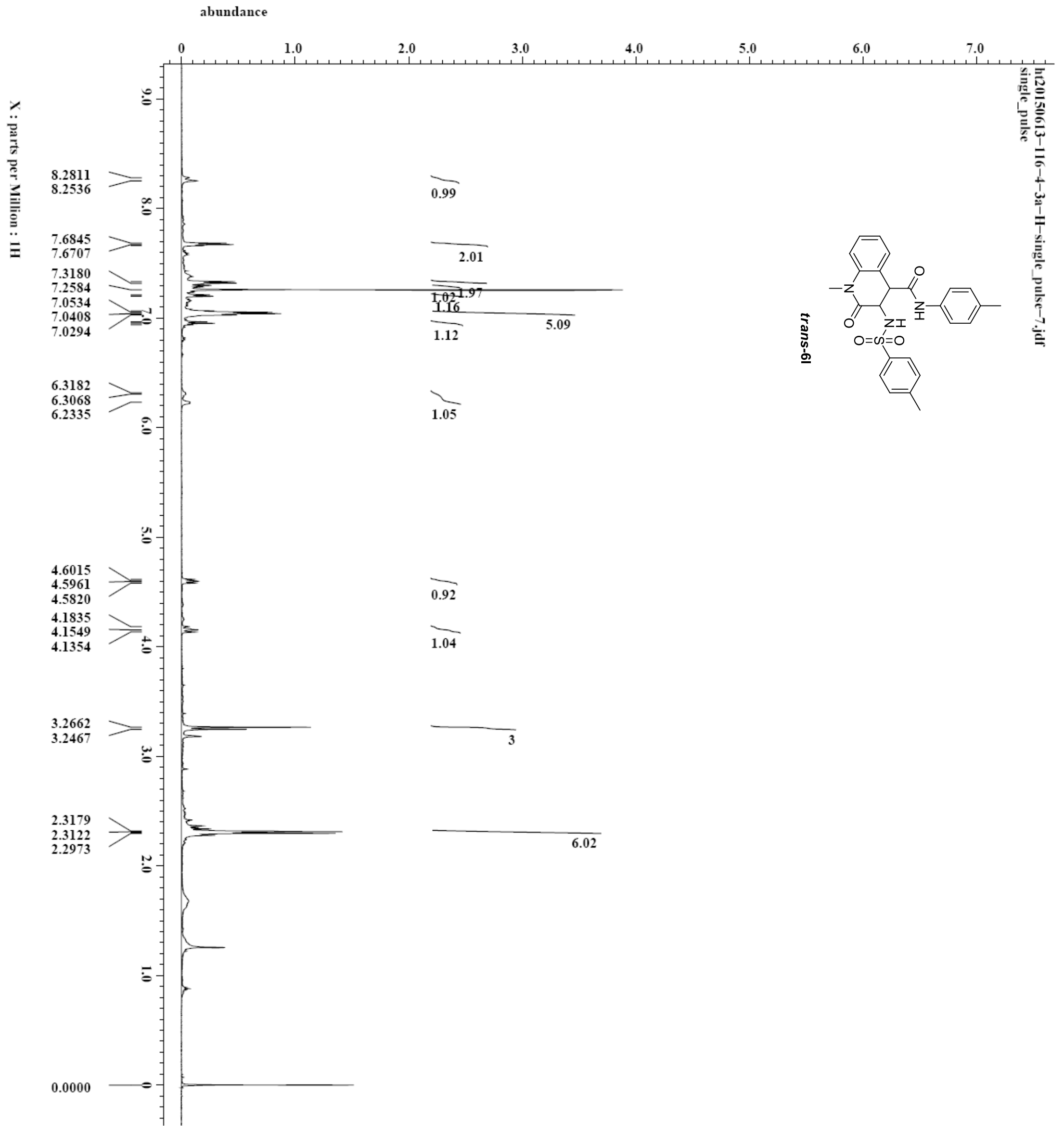
abundance
0
1.0
2.0
3.0
4.0
5.0
6.0
7.0
hi20150613−116−4−3a−H−single_pulse−7.jdf
single_pulse
X : parts per Million : 1H
8.2811
8.2536
7.6845
7.6707
7.3180
7.2584
7.0534
7.0408
7.0294
6.3182
6.3068
6.2335
4.6015
4.5961
4.5820
4.1835
4.1549
4.1354
3.2662
3.2467
2.3179
2.3122
2.2973
0.0000
0.99
2.01
1.02
1.97
1.16
5.09
1.12
1.05
0.92
1.04
3
6.02
trans-6l

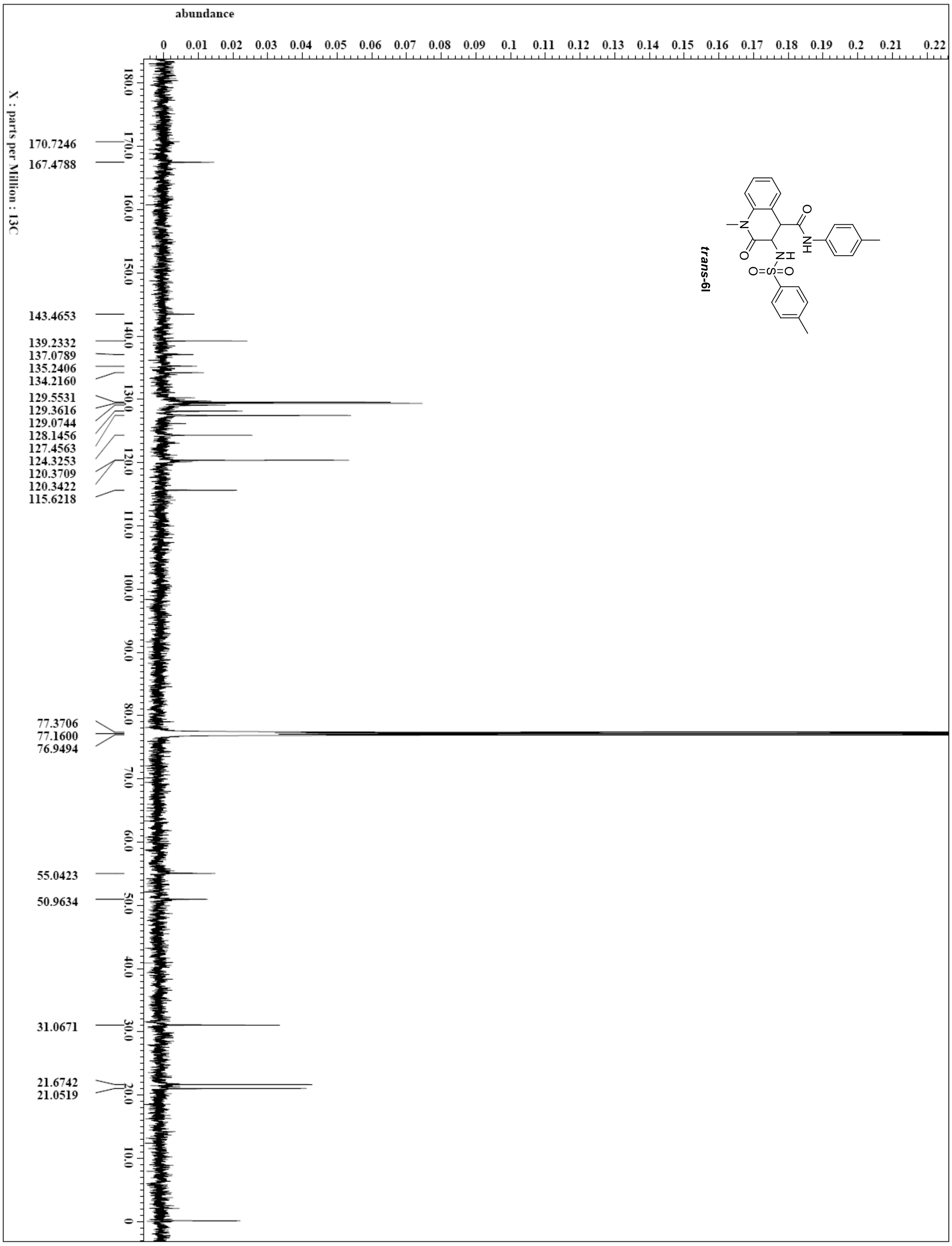
abundance
X : parts per Million : 13C
170.7246
167.4788
143.4653
139.2332
137.0789
135.2406
134.2160
129.5531
129.3616
129.0744
128.1456
127.4563
124.3253
120.3709
120.3422
115.6218
77.3706
77.1600
76.9494
55.0423
50.9634
31.0671
21.6742
21.0519
trans-6l

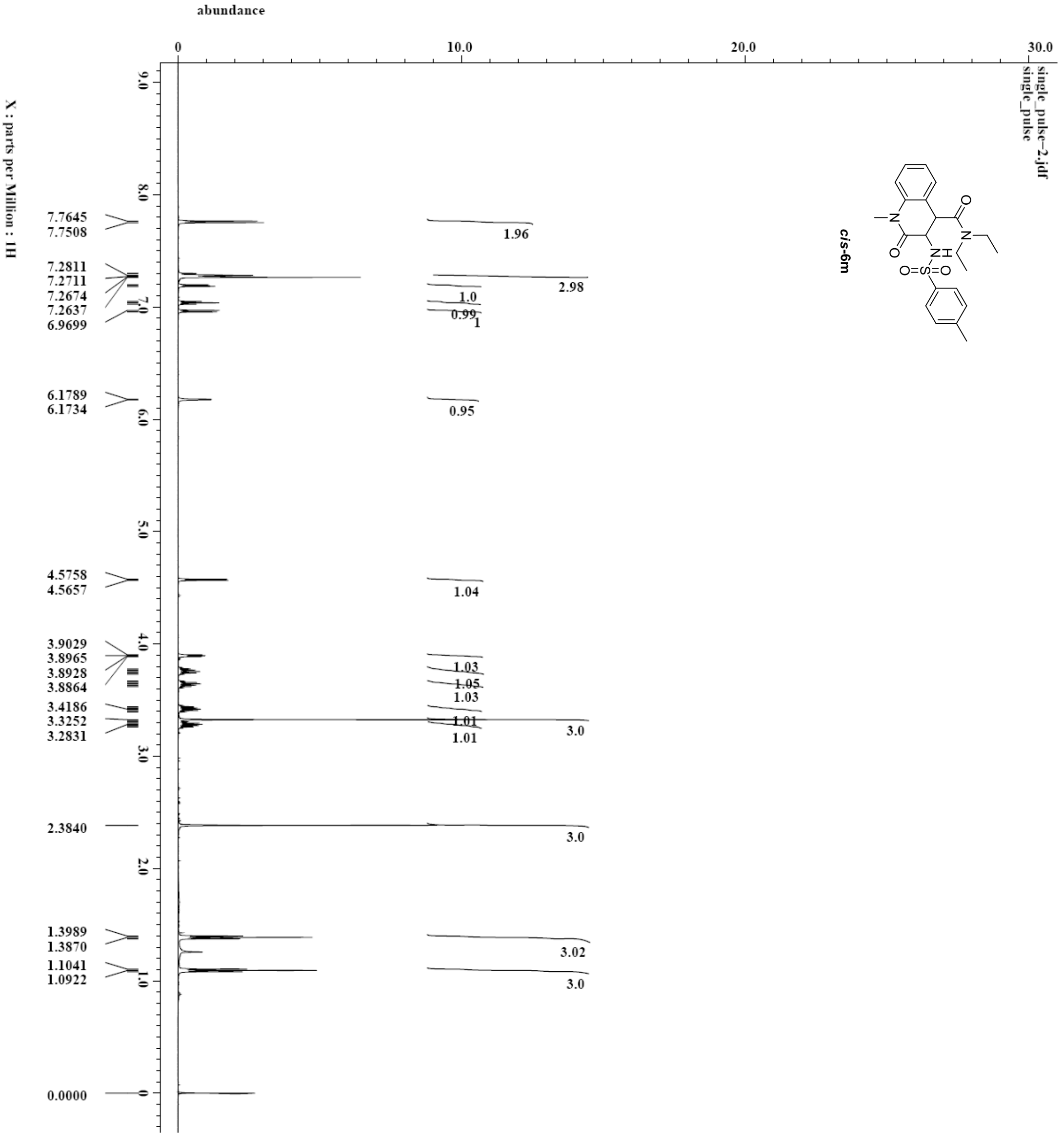

abundance
0
10.0
20.0
30.0
single_pulse-2.jdf
single_pulse
X : parts per Million : 1H
cis-6m
7.7645
7.7508
7.2811
7.2711
7.2674
7.2637
6.9699
6.1789
6.1734
4.5758
4.5657
3.9029
3.8965
3.8928
3.8864
3.4186
3.3252
3.2831
2.3840
1.3989
1.3870
1.1041
1.0922
0.0000
1.96
2.98
1.0
0.99
1
0.95
1.04
1.03
1.05
1.03
1.01
1.01
3.0
3.0
3.02
3.0

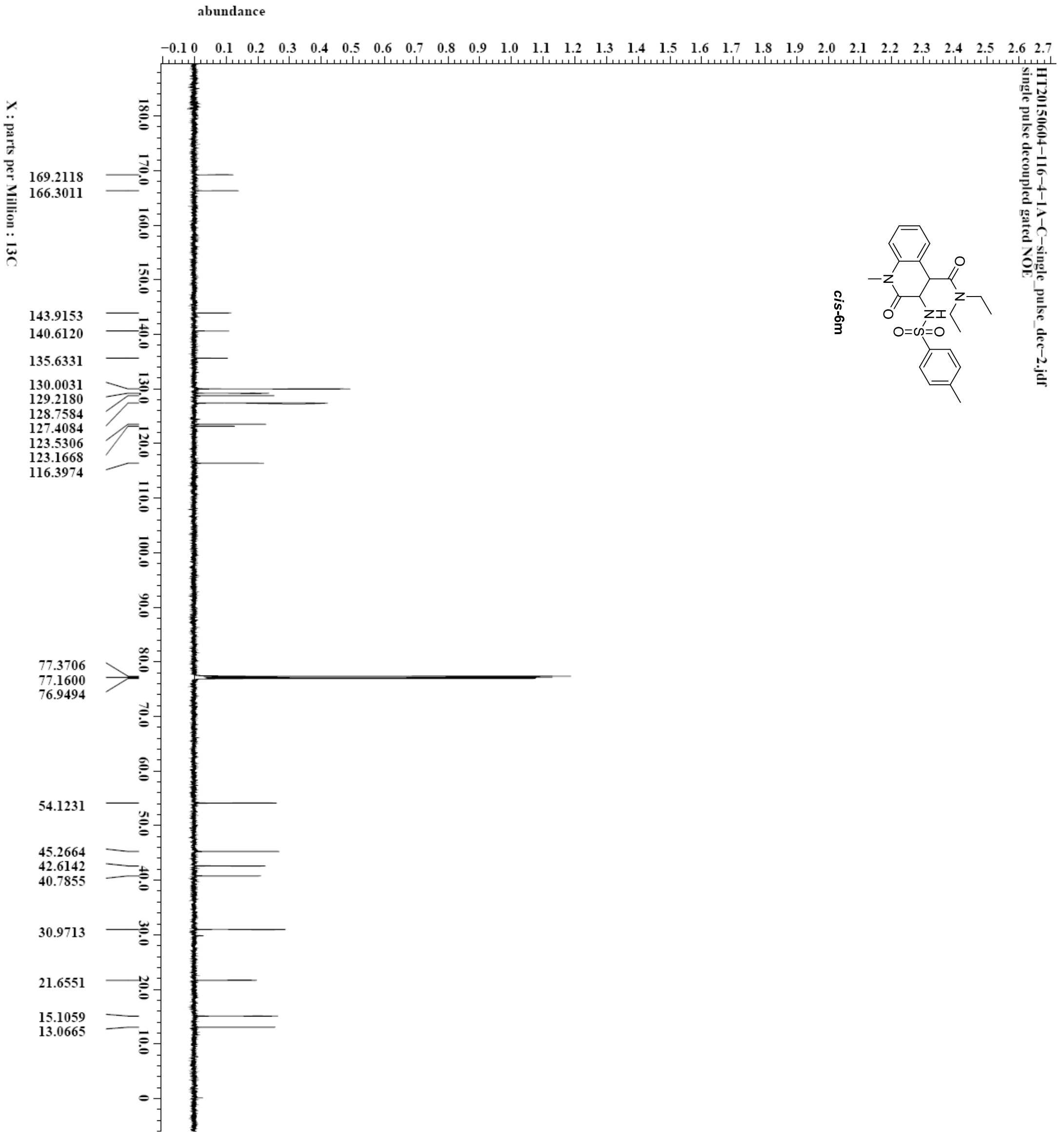
abundance
HT20150604-116-4-1A-C-single_pulse_dec-2.jdf
single pulse decoupled gated NOE
cis-6m
X : parts per Million : 13C
169.2118
166.3011
143.9153
140.6120
135.6331
130.0031
129.2180
128.7584
127.4084
123.5306
123.1668
116.3974
77.3706
77.1600
76.9494
54.1231
45.2664
42.6142
40.7855
30.9713
21.6551
15.1059
13.0665

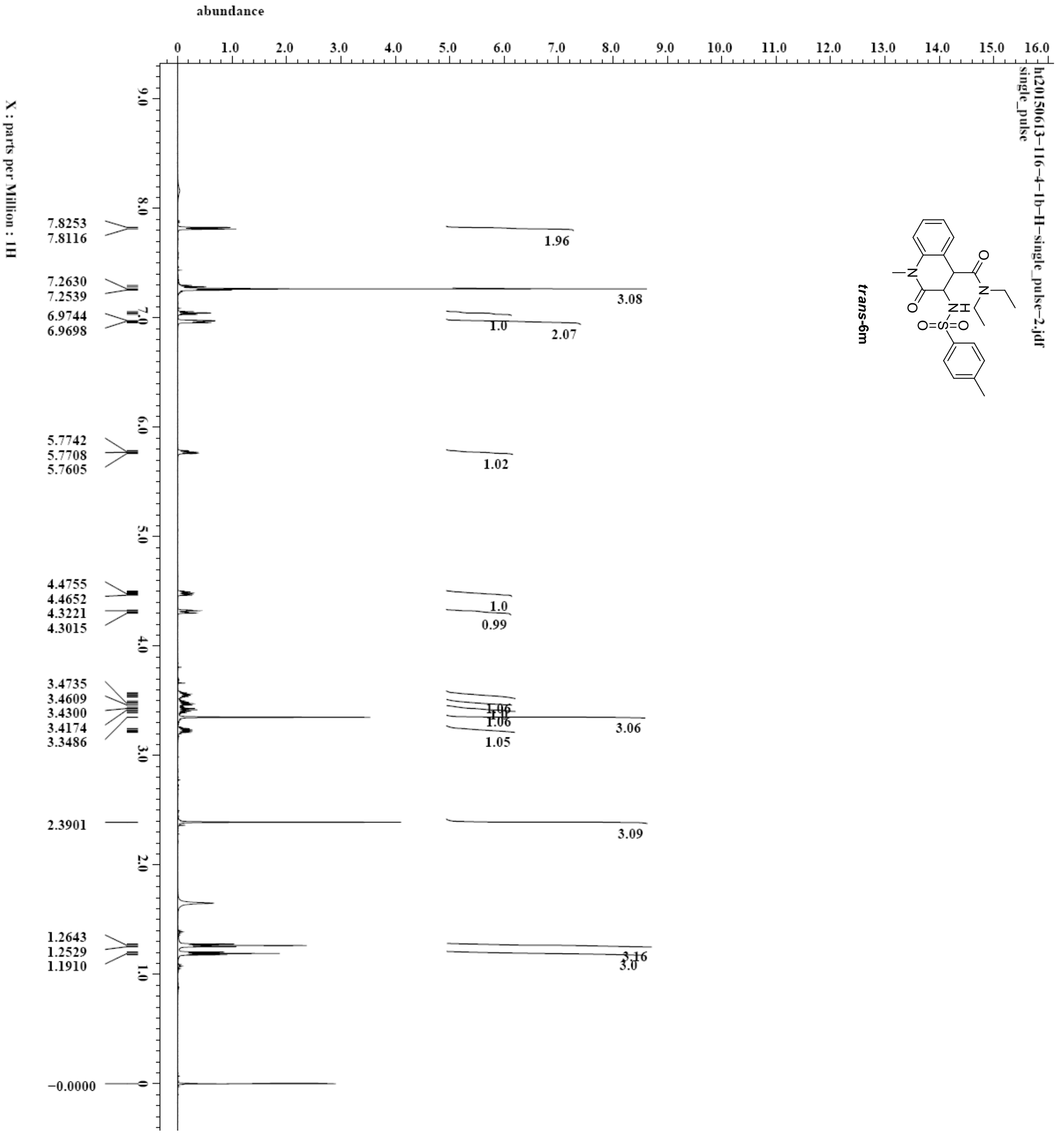
abundance
X : parts per Million : 1H
h20150613-116-4-1b-H-single_pulse-2.jdf
single_pulse
trans-6m
7.8253
7.8116
7.2630
7.2539
6.9744
6.9698
5.7742
5.7708
5.7605
4.4755
4.4652
4.3221
4.3015
3.4735
3.4609
3.4300
3.4174
3.3486
2.3901
1.2643
1.2529
1.1910
-0.0000
1.96
3.08
1.0
2.07
1.02
1.0
0.99
3.06
1.05
3.09
3.16
3.0

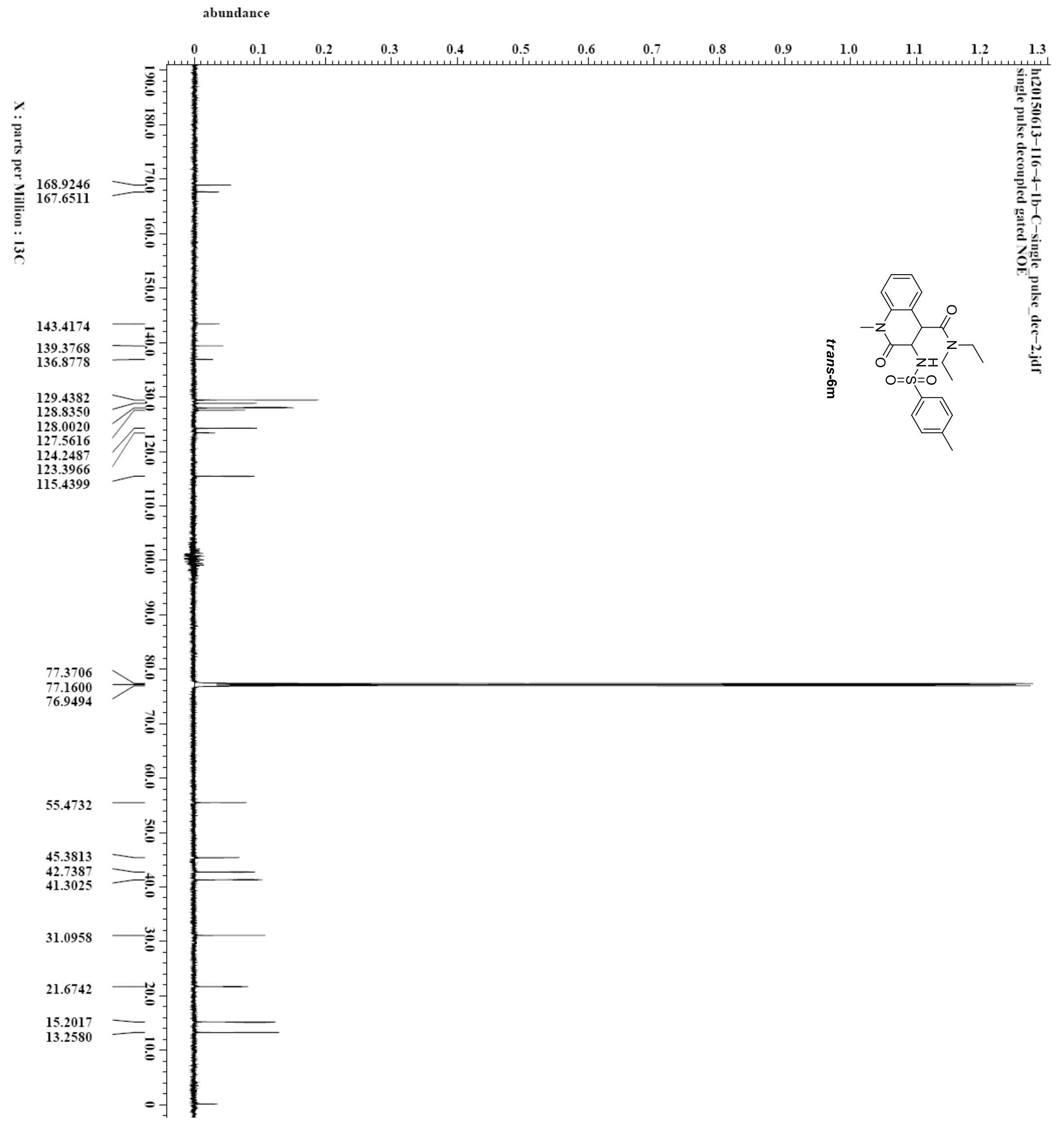
abundance
0 0.1 0.2 0.3 0.4 0.5 0.6 0.7 0.8 0.9 1.0 1.1 1.2 1.3
h20150613-116-4-1b-C-single_pulse_dec-2.jdf
single pulse decoupled gated NOE
trans-6m
X : parts per Million : 13C
190.0 180.0 170.0 160.0 150.0 140.0 130.0 120.0 110.0 100.0 90.0 80.0 70.0 60.0 50.0 40.0 30.0 20.0 10.0 0
168.9246
167.6511
143.4174
139.3768
136.8778
129.4382
128.8350
128.0020
127.5616
124.2487
123.3966
115.4399
77.3706
77.1600
76.9494
55.4732
45.3813
42.7387
41.3025
31.0958
21.6742
15.2017
13.2580

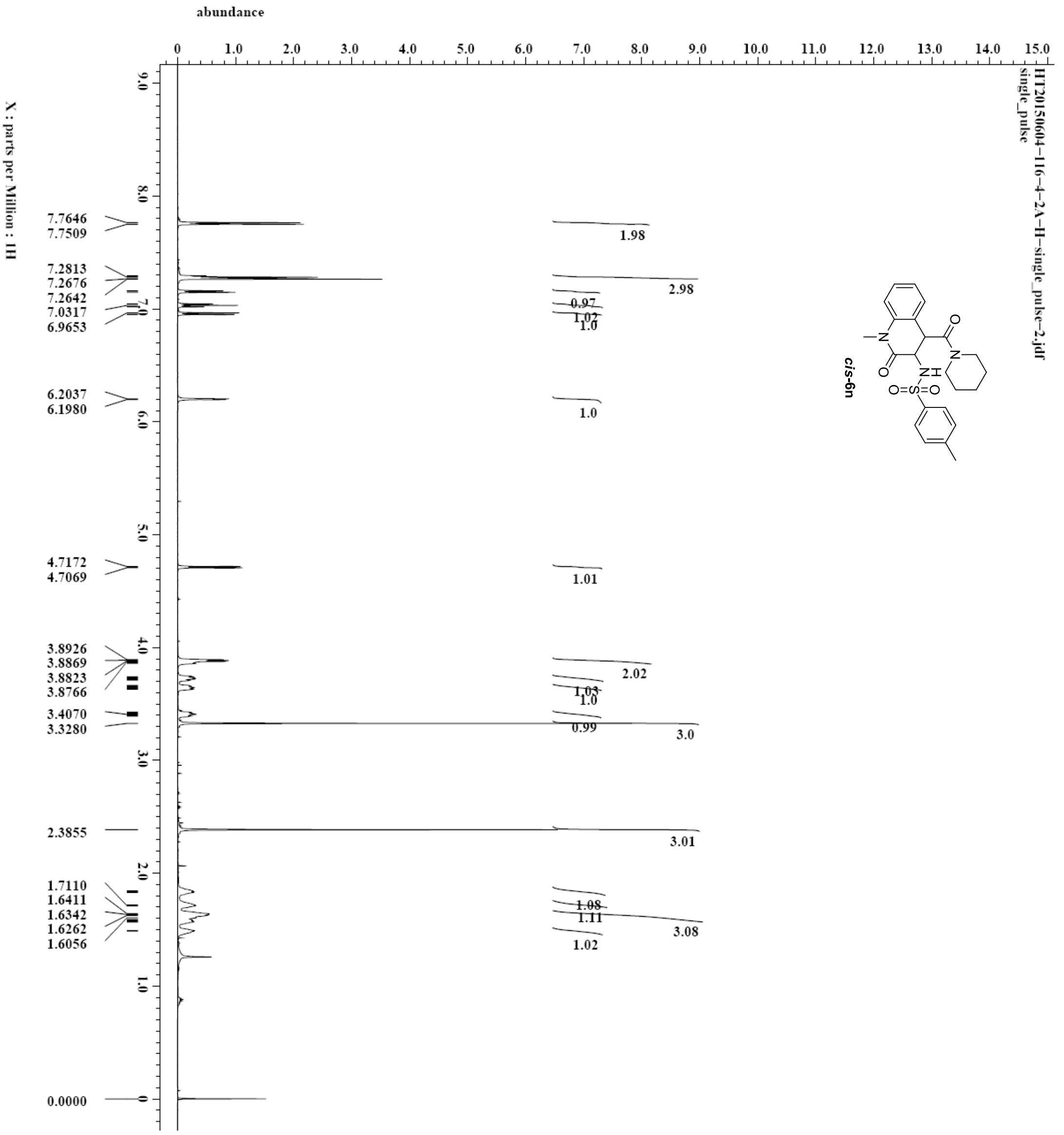
HT20150604-116-4-2A-H-single_pulse-2.jdf
single_pulse
abundance
X : parts per Million : 1H
7.7646
7.7509
7.2813
7.2676
7.2642
7.0317
6.9653
6.2037
6.1980
4.7172
4.7069
3.8926
3.8869
3.8823
3.8766
3.4070
3.3280
2.3855
1.7110
1.6411
1.6342
1.6262
1.6056
0.0000
1.98
2.98
0.97
1.02
1.0
1.0
1.01
2.02
1.03
1.0
0.99
3.0
3.01
1.08
1.11
3.08
1.02
cis-6n

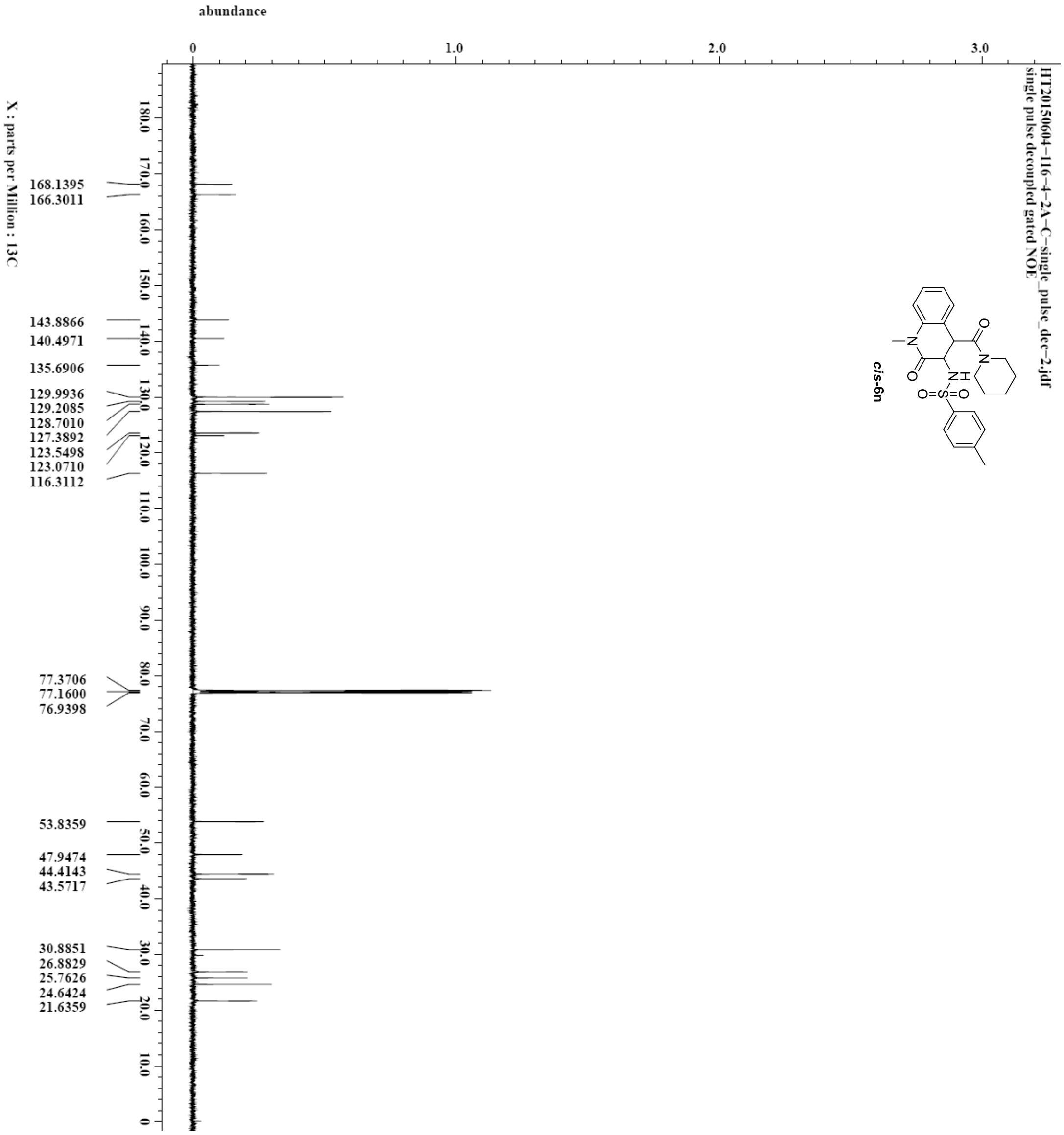
HT20150604-116-4-2A-C-single_pulse_dec-2.jdf
single pulse decoupled gated NOE
abundance
X : parts per Million : 13C
cis-6n
168.1395
166.3011
143.8866
140.4971
135.6906
129.9936
129.2085
128.7010
127.3892
123.5498
123.0710
116.3112
77.3706
77.1600
76.9398
53.8359
47.9474
44.4143
43.5717
30.8851
26.8829
25.7626
24.6424
21.6359

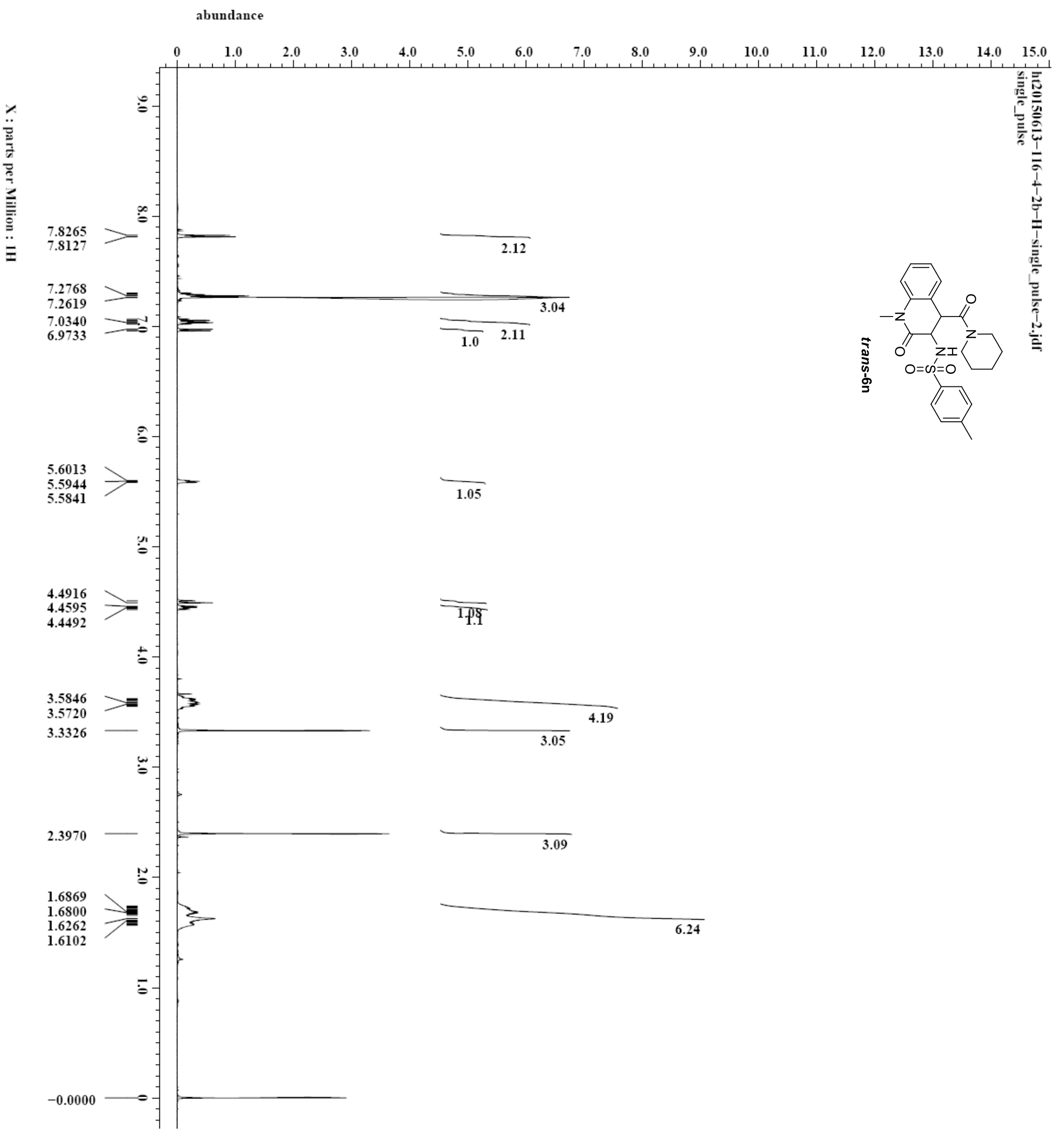
abundance
X : parts per Million : 1H
ht20150613-116-4-2b-H-single_pulse-2.jdf
single_pulse
trans-6n
7.8265
7.8127
7.2768
7.2619
7.0340
6.9733
5.6013
5.5944
5.5841
4.4916
4.4595
4.4492
3.5846
3.5720
3.3326
2.3970
1.6869
1.6800
1.6262
1.6102
-0.0000
2.12
3.04
2.11
1.0
1.05
1.08
1.1
4.19
3.05
3.09
6.24

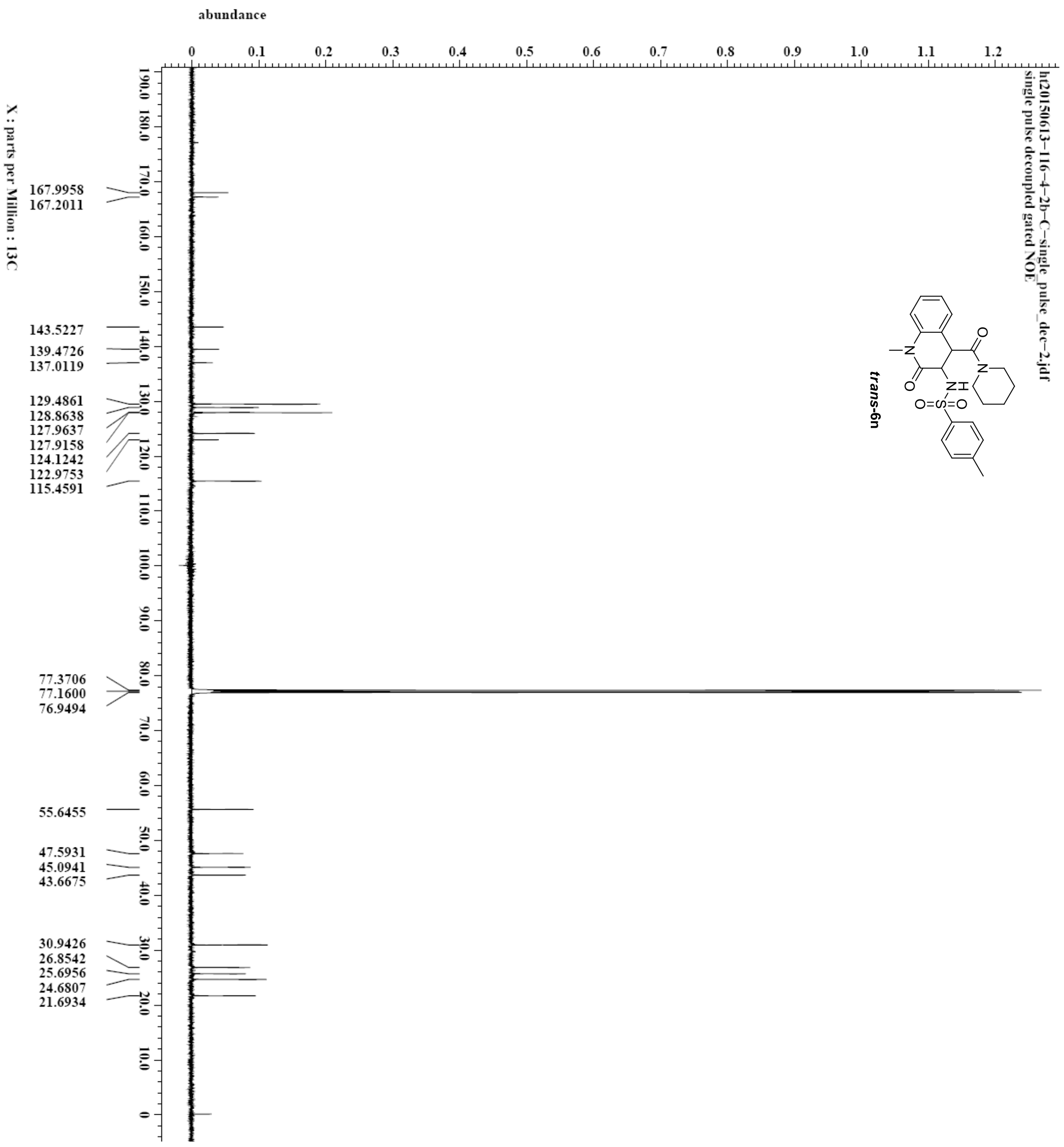
abundance
X : parts per Million : 13C
hl20150613-116-4-2b-C-single_pulse_dec-2.jdf
single pulse decoupled gated NOE
trans-6n
167.9958
167.2011
143.5227
139.4726
137.0119
129.4861
128.8638
127.9637
127.9158
124.1242
122.9753
115.4591
77.3706
77.1600
76.9494
55.6455
47.5931
45.0941
43.6675
30.9426
26.8542
25.6956
24.6807
21.6934

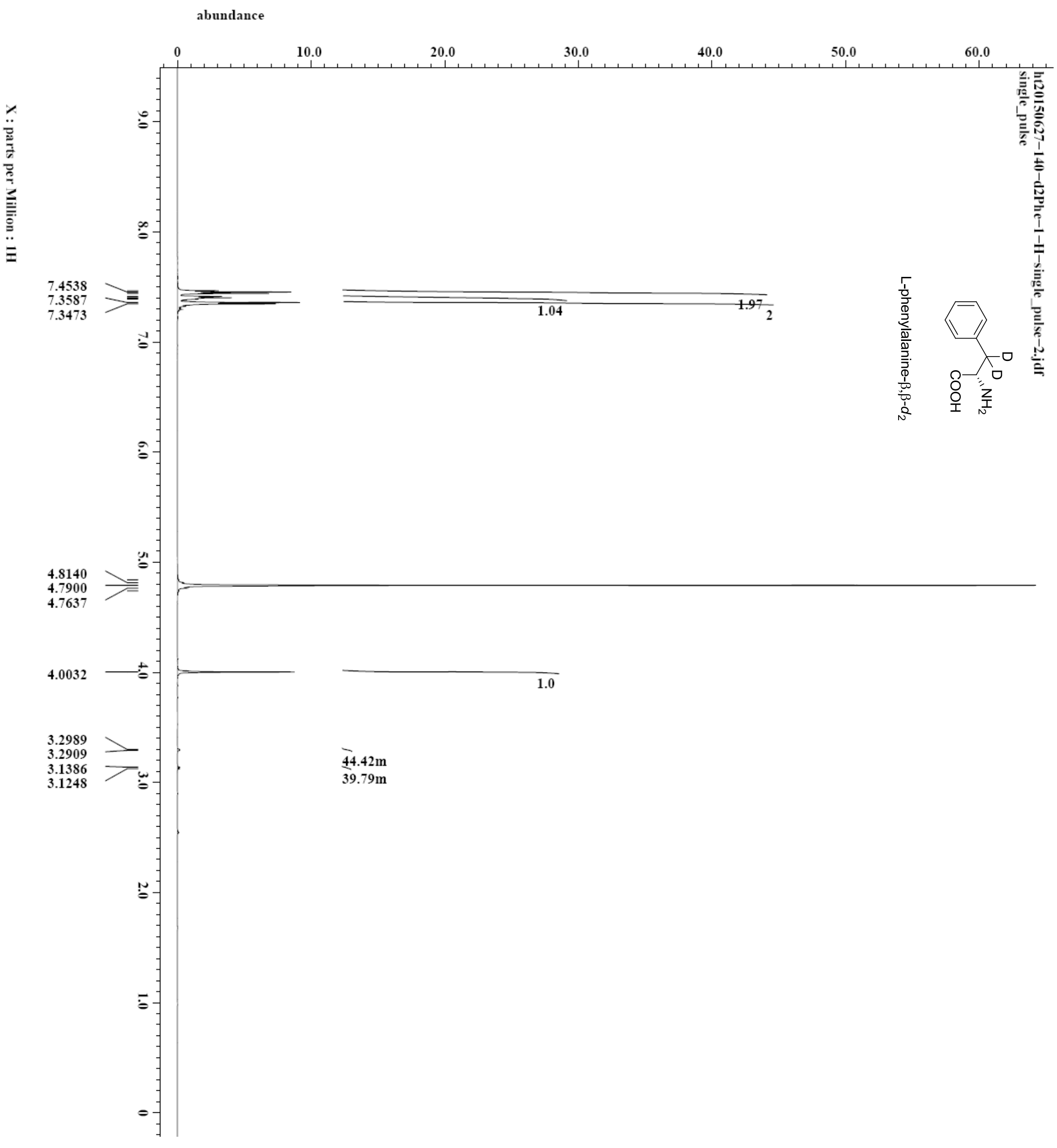
abundance
h20150627-140-d2Phe-1-1H-single_pulse-2.jdf
single_pulse
L-phenylalanine-β,β-d_2
X : parts per Million : 1H
7.4538
7.3587
7.3473
4.8140
4.7900
4.7637
4.0032
3.2989
3.2909
3.1386
3.1248
1.04
1.97
2
1.0
44.42m
39.79m

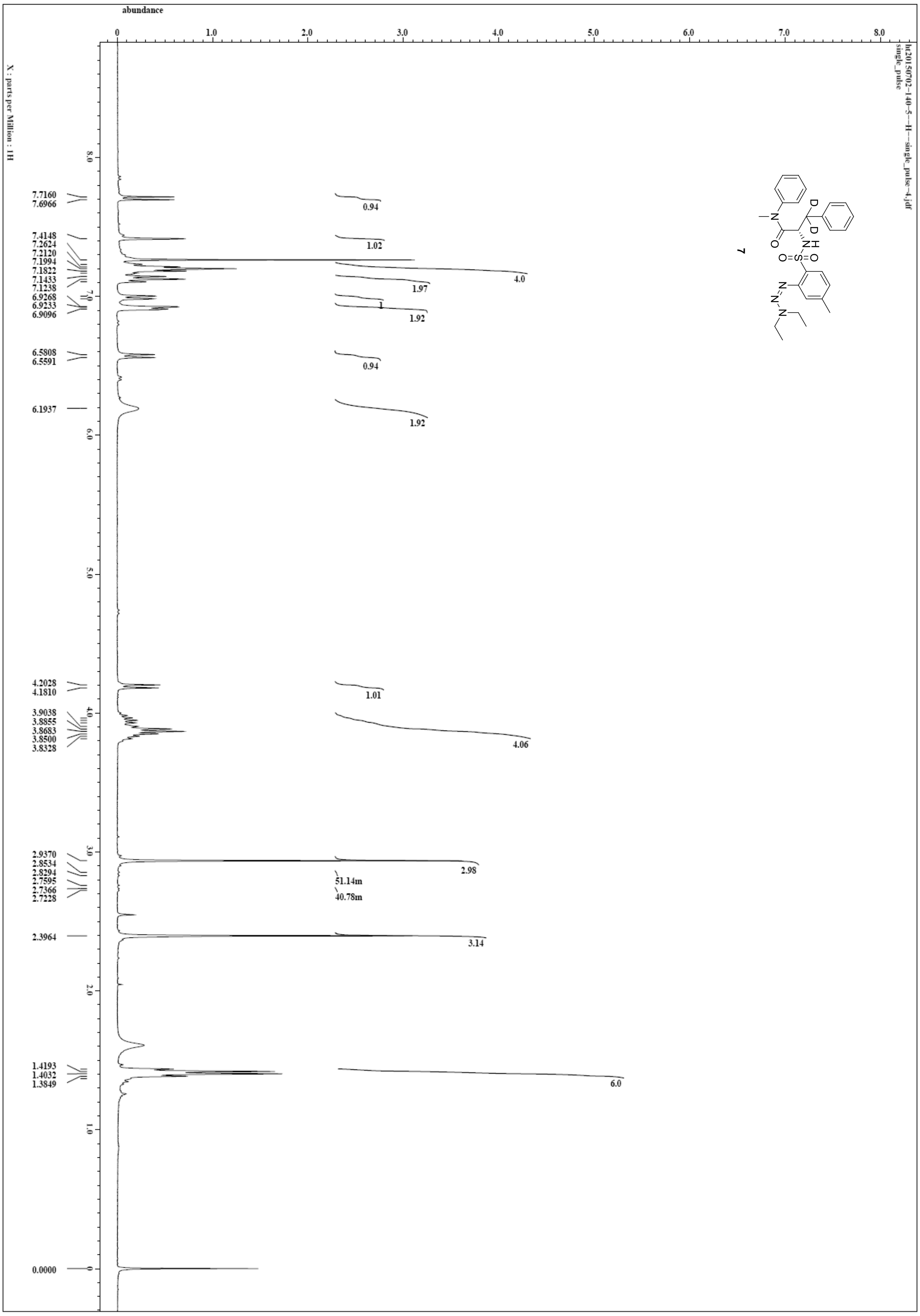

abundance
X : parts per Million : 1H
hz20150702-140-5---H---single_pulse-4.jdf
single_pulse
7
7.7160
7.6966
7.4148
7.2624
7.2120
7.1994
7.1822
7.1433
7.1238
6.9268
6.9233
6.9096
6.5808
6.5591
6.1937
4.2028
4.1810
3.9038
3.8855
3.8683
3.8500
3.8328
2.9370
2.8534
2.8294
2.7595
2.7366
2.7228
2.3964
1.4193
1.4032
1.3849
0.0000
0.94
1.02
4.0
1.97
1
1.92
0.94
1.92
1.01
4.06
2.98
51.14m
40.78m
3.14
6.0

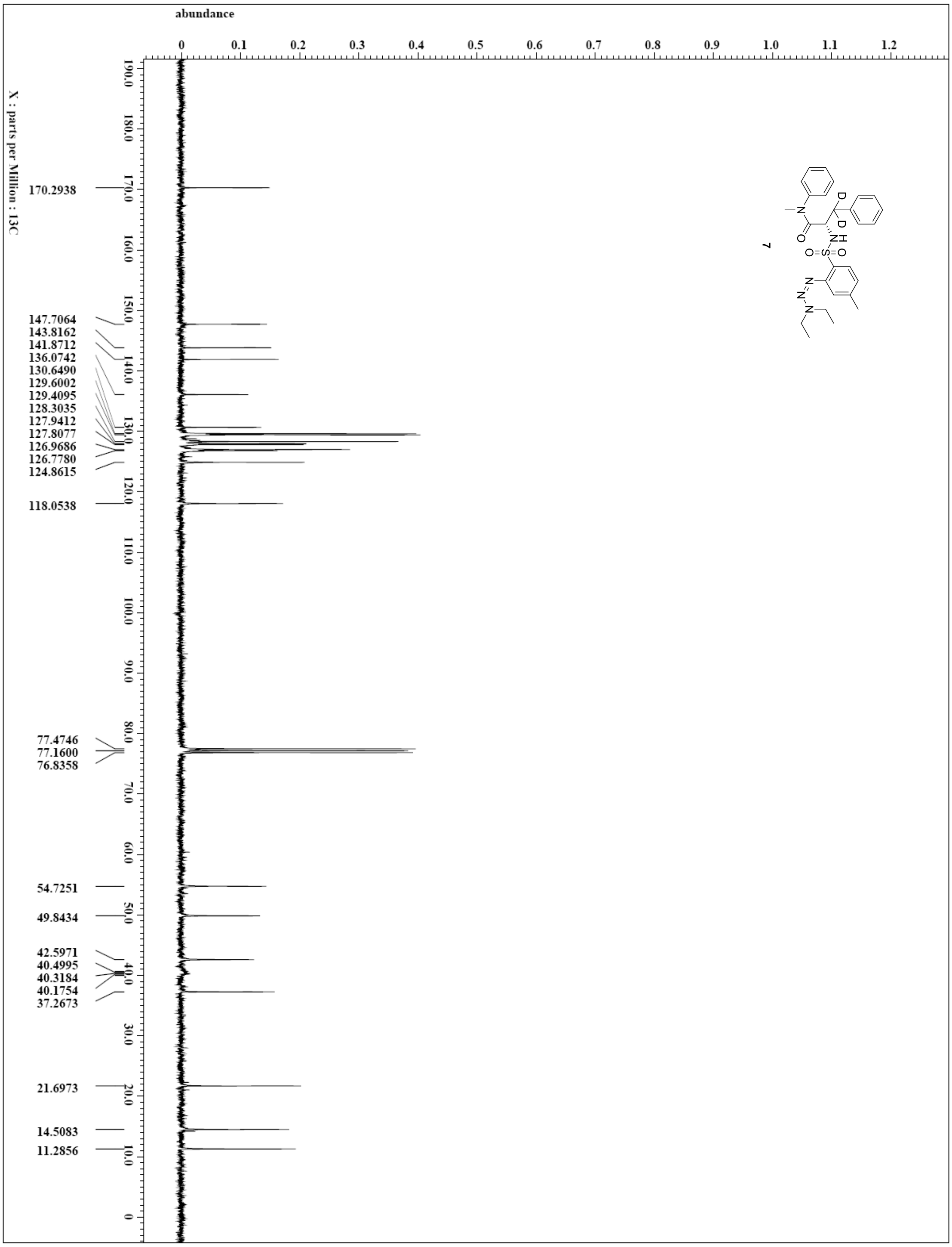
abundance
0 0.1 0.2 0.3 0.4 0.5 0.6 0.7 0.8 0.9 1.0 1.1 1.2
X : parts per Million : 13C
190.0 180.0 170.0 160.0 150.0 140.0 130.0 120.0 110.0 100.0 90.0 80.0 70.0 60.0 50.0 40.0 30.0 20.0 10.0 0
170.2938
147.7064
143.8162
141.8712
136.0742
130.6490
129.6002
129.4095
128.3035
127.9412
127.8077
126.9686
126.7780
124.8615
118.0538
77.4746
77.1600
76.8358
54.7251
49.8434
42.5971
40.4995
40.3184
40.1754
37.2673
21.6973
14.5083
11.2856
7

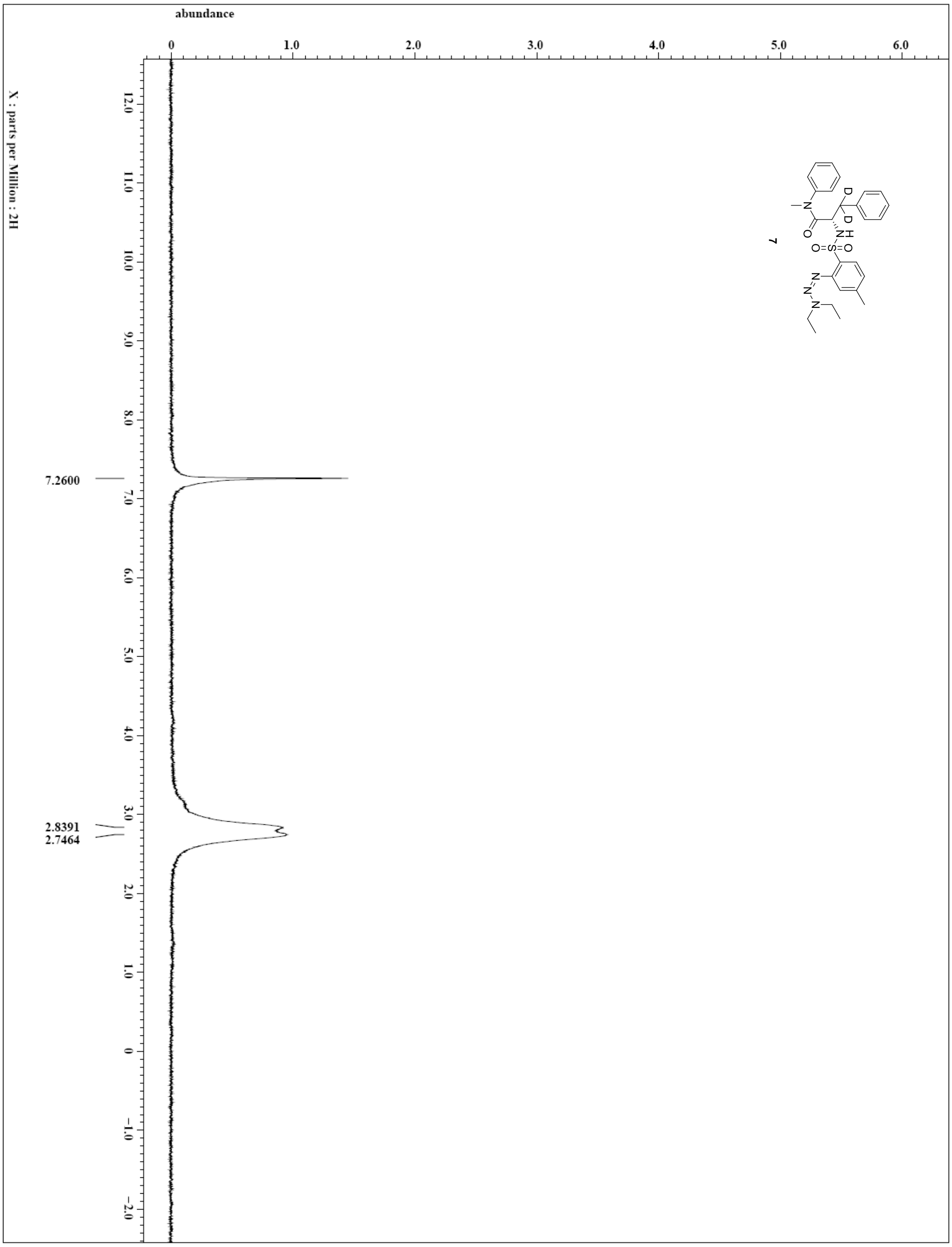
abundance
X : parts per Million : 2H
7.2600
2.8391
2.7464
7

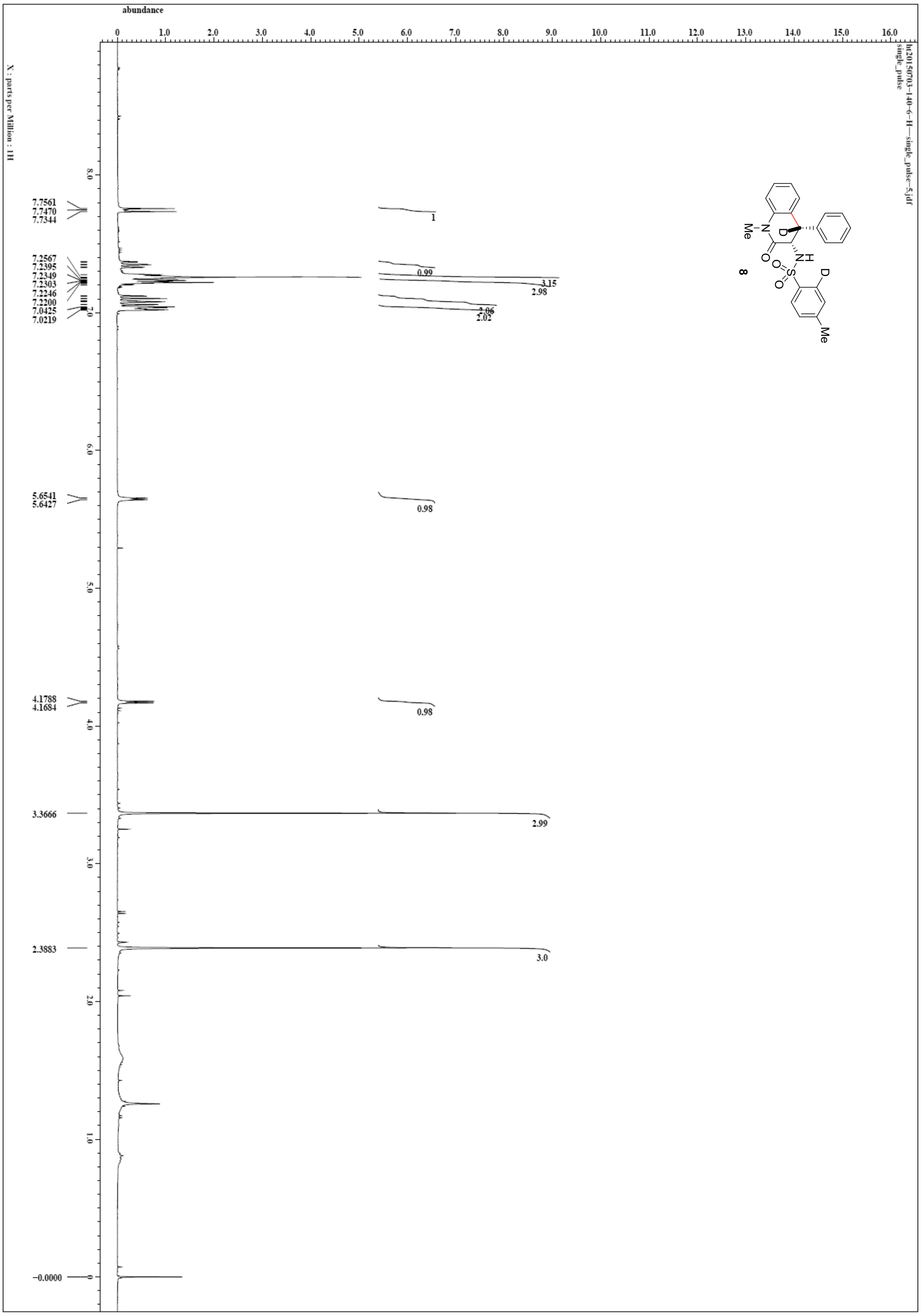
abundance
X : parts per Million : 1H
hc20150703-140-6-H---single_pulse-5.jdf
single_pulse
7.7561
7.7470
7.7344
7.2567
7.2395
7.2349
7.2303
7.2246
7.2200
7.0425
7.0219
5.6541
5.6427
4.1788
4.1684
3.3666
2.3883
-0.0000
1
0.99
3.15
2.98
2.06
2.02
0.98
0.98
2.99
3.0
8

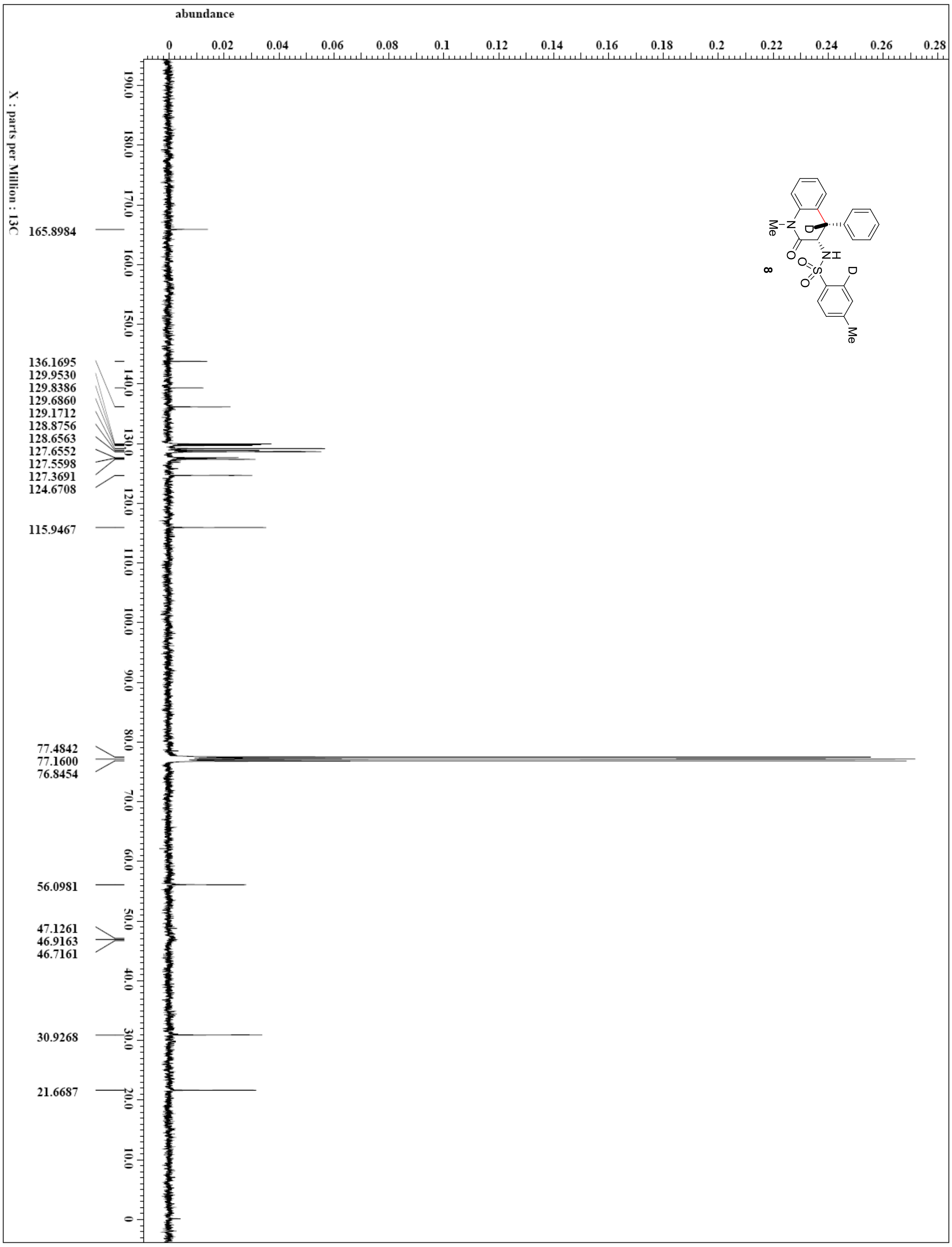

abundance
X : parts per Million : 13C
165.8984
136.1695
129.9530
129.8386
129.6860
129.1712
128.8756
128.6563
127.6552
127.5598
127.3691
124.6708
115.9467
77.4842
77.1600
76.8454
56.0981
47.1261
46.9163
46.7161
30.9268
21.6687
8

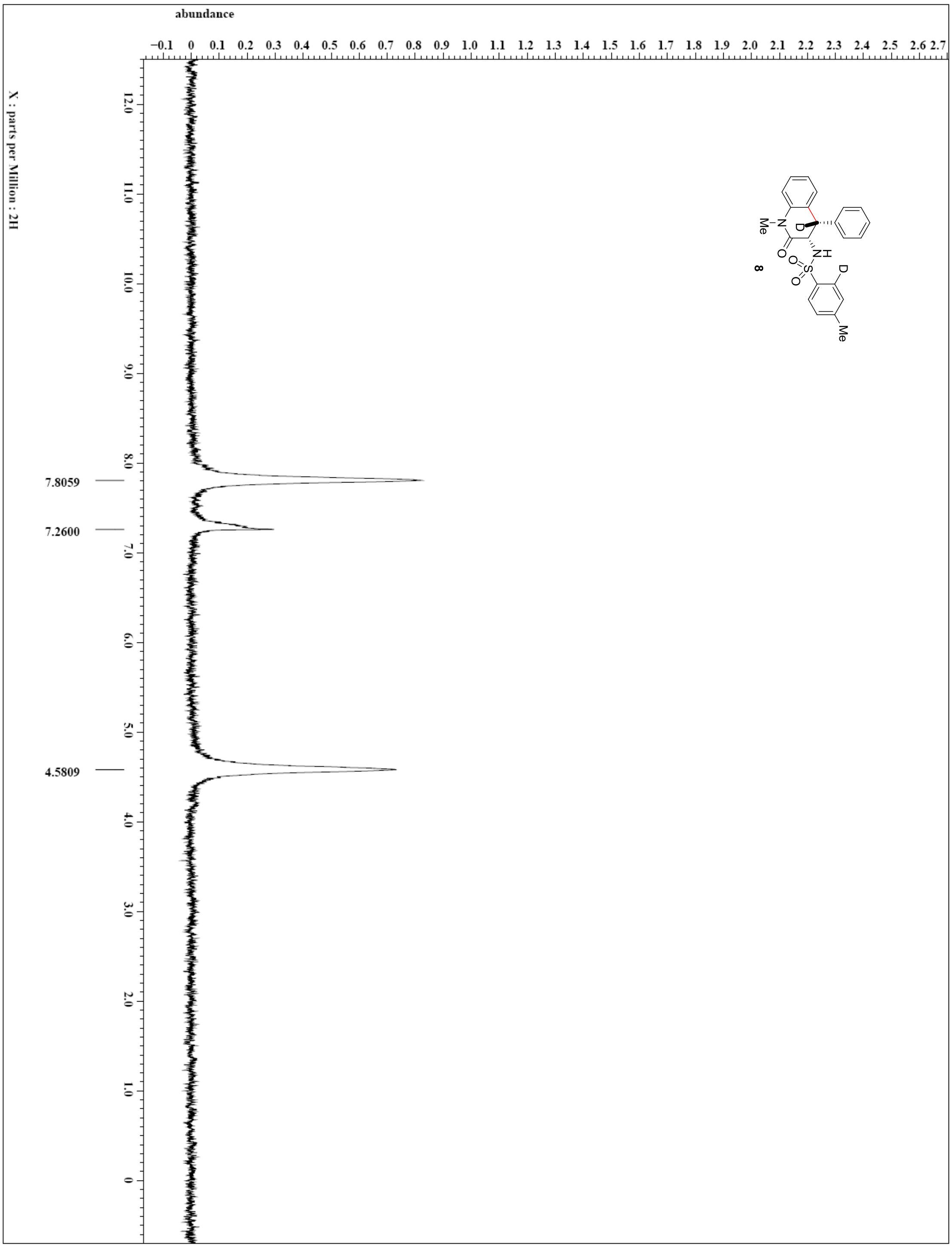
abundance
X : parts per Million : 2H
7.8059
7.2600
4.5809
8

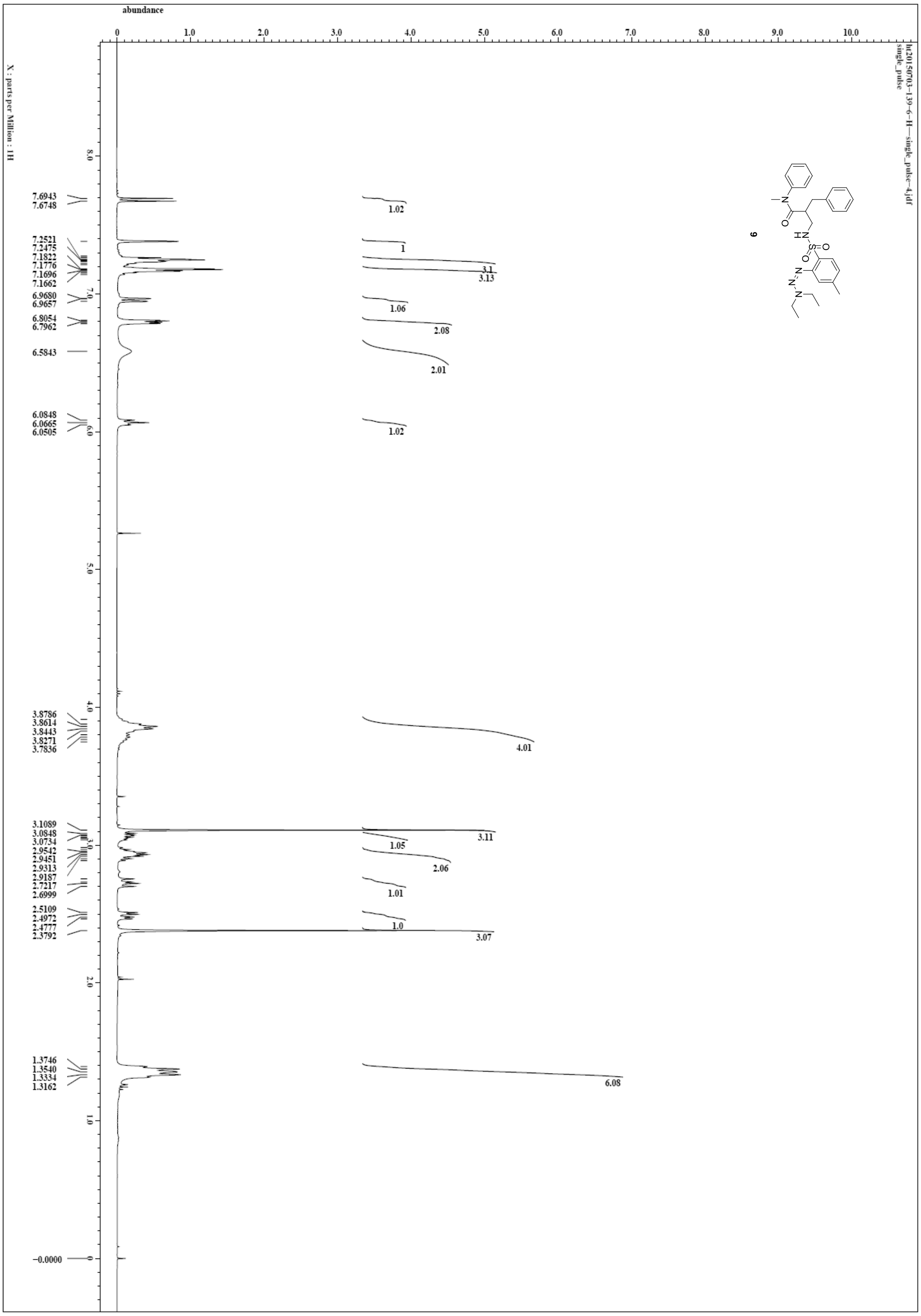
abundance
X : parts per Million : 1H
6
7.6943
7.6748
7.2521
7.2475
7.1822
7.1776
7.1696
7.1662
6.9680
6.9657
6.8054
6.7962
6.5843
6.0848
6.0665
6.0505
3.8786
3.8614
3.8443
3.8271
3.7836
3.1089
3.0848
3.0734
2.9542
2.9451
2.9313
2.9187
2.7217
2.6999
2.5109
2.4972
2.4777
2.3792
1.3746
1.3540
1.3334
1.3162
-0.0000
1.02
1
3.1
3.13
1.06
2.08
2.01
1.02
4.01
3.11
1.05
2.06
1.01
1.0
3.07
6.08

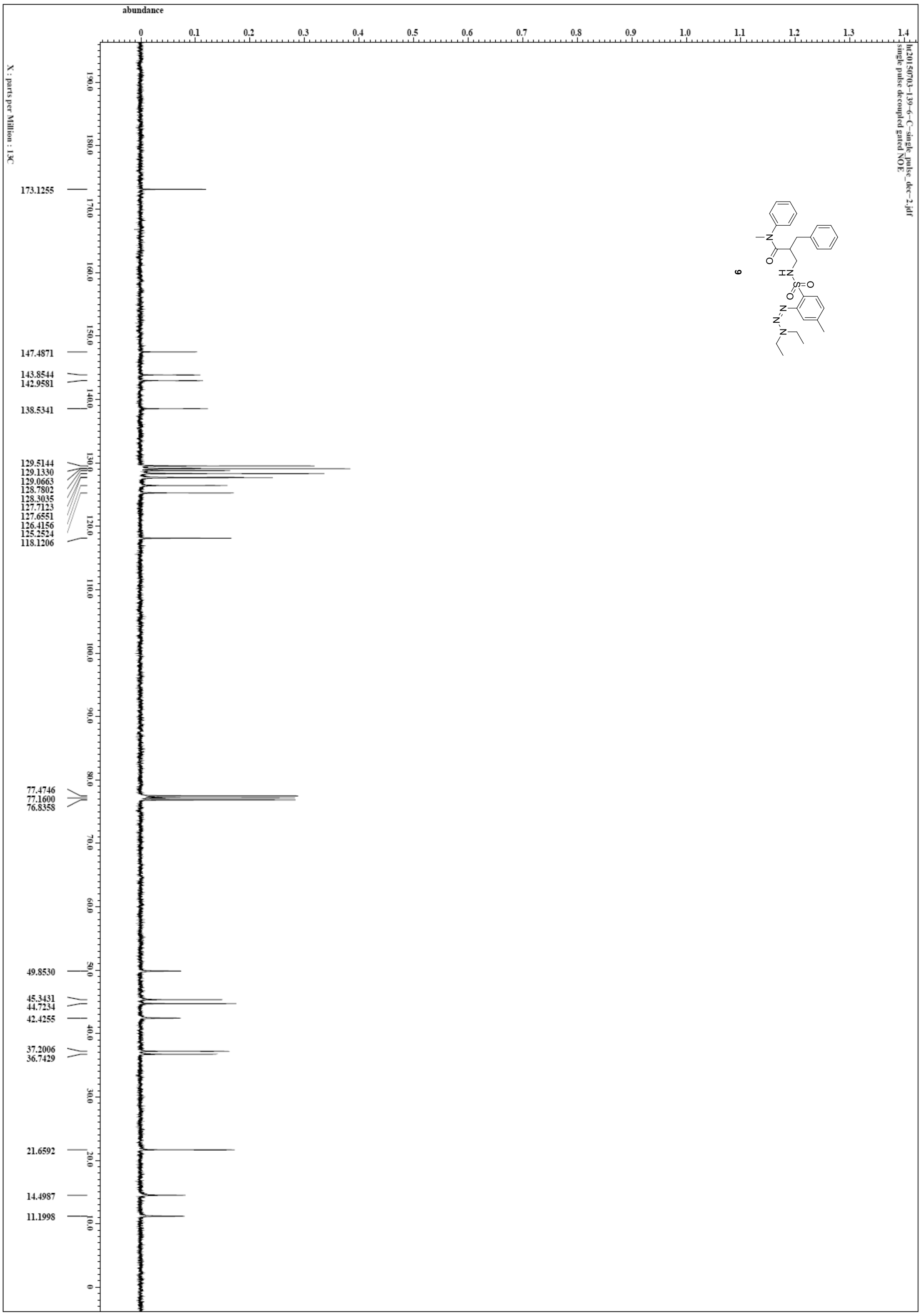
abundance
X : parts per Million : 13C
hi20150703-139-6-C-single_pulse_dec-2.jdf
single pulse decoupled gated NOE
173.1255
147.4871
143.8544
142.9581
138.5341
129.5144
129.1330
129.0663
128.7802
128.3035
127.7123
127.6551
126.4156
125.2524
118.1206
77.4746
77.1600
76.8358
49.8530
45.3431
44.7234
42.4255
37.2006
36.7429
21.6592
14.4987
11.1998
9

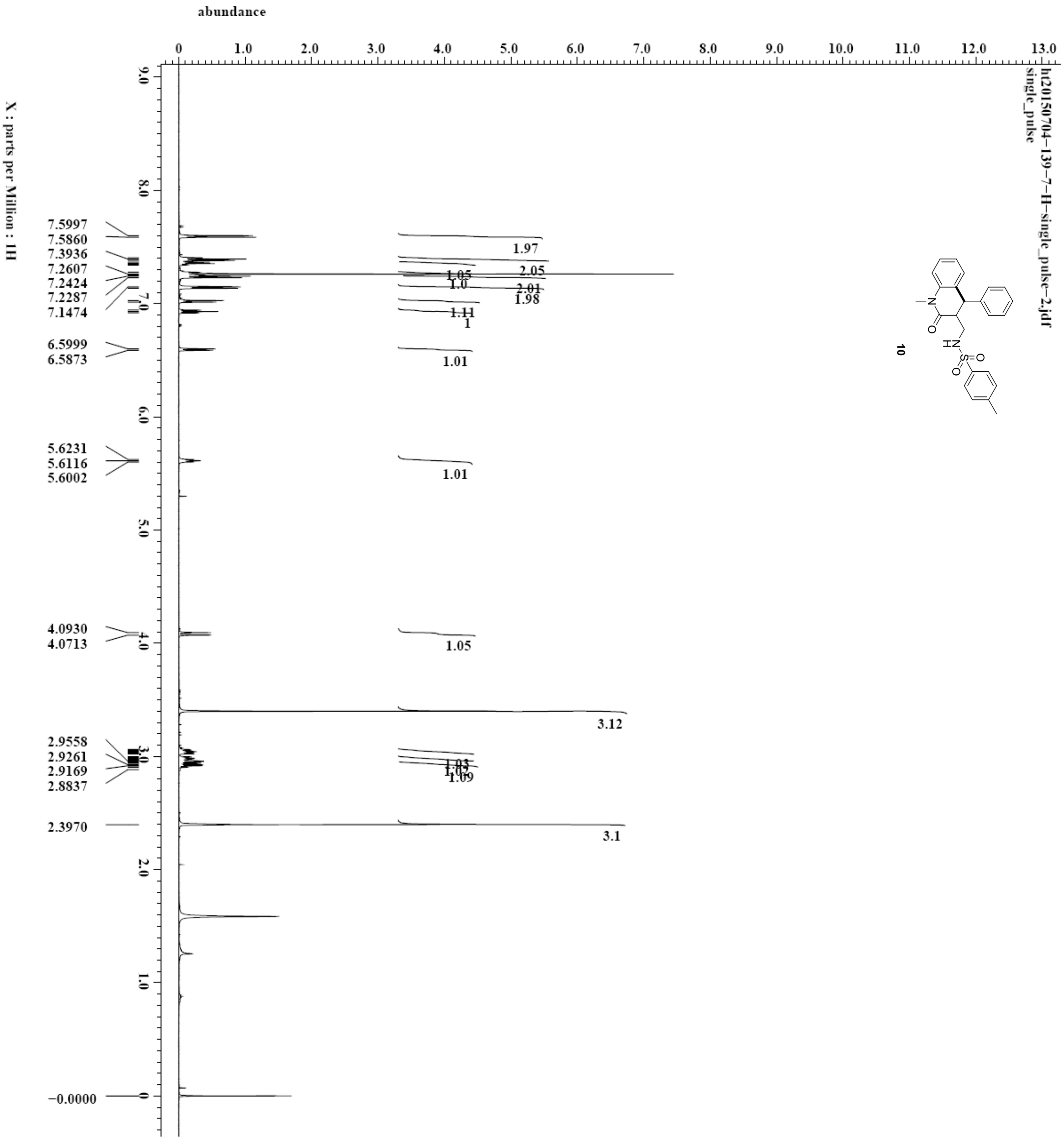
abundance
X : parts per Million : 1H
h20150704-139-7-H-single_pulse-2.jdf
single_pulse
7.5997
7.5860
7.3936
7.2607
7.2424
7.2287
7.1474
6.5999
6.5873
5.6231
5.6116
5.6002
4.0930
4.0713
2.9558
2.9261
2.9169
2.8837
2.3970
−0.0000
10

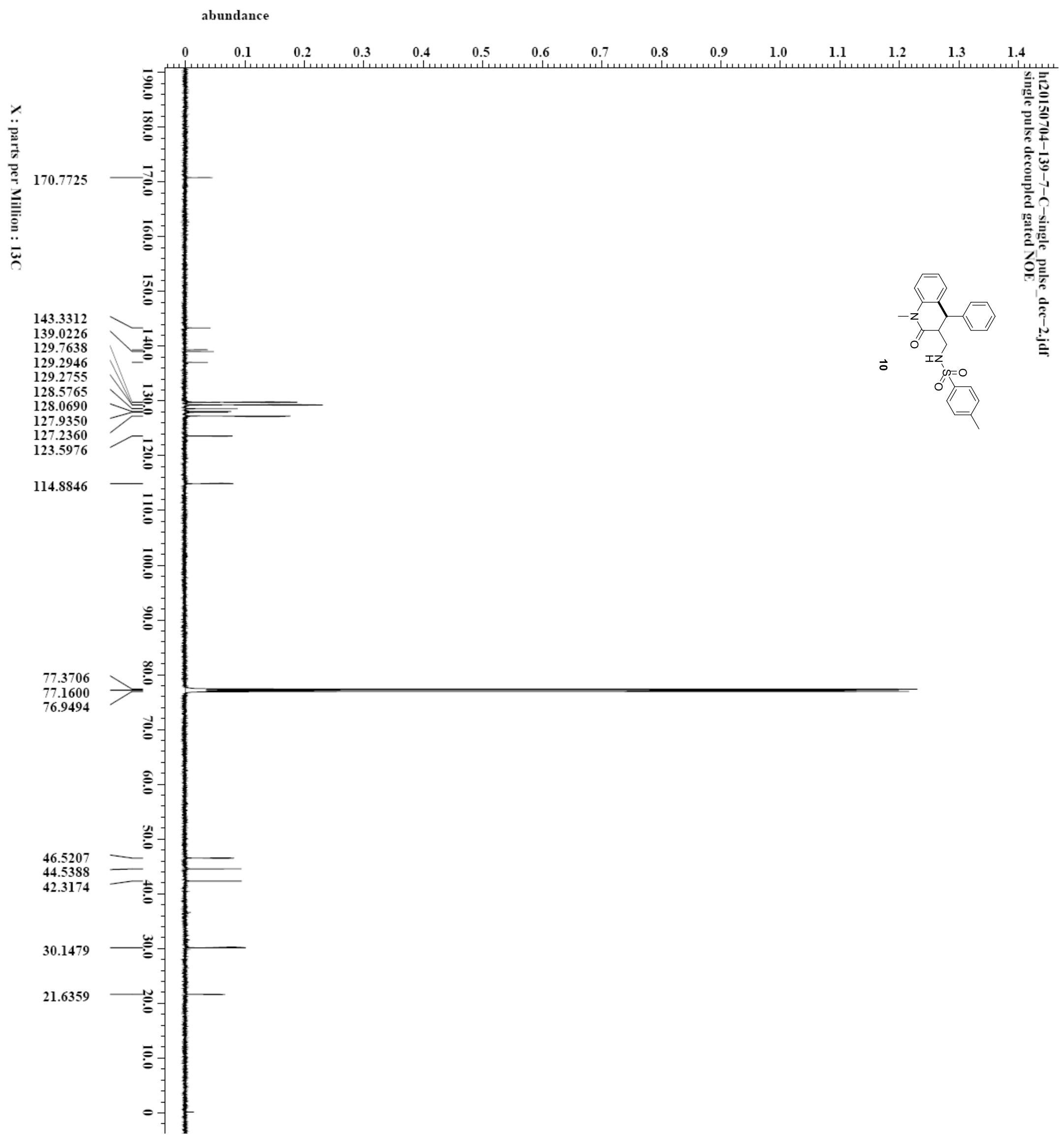
abundance
X : parts per Million : 13C
ht20150704-139-7-C-single_pulse_dec-2.jdf
single pulse decoupled gated NOE
10
170.7725
143.3312
139.0226
129.7638
129.2946
129.2755
128.5765
128.0690
127.9350
127.2360
123.5976
114.8846
77.3706
77.1600
76.9494
46.5207
44.5388
42.3174
30.1479
21.6359

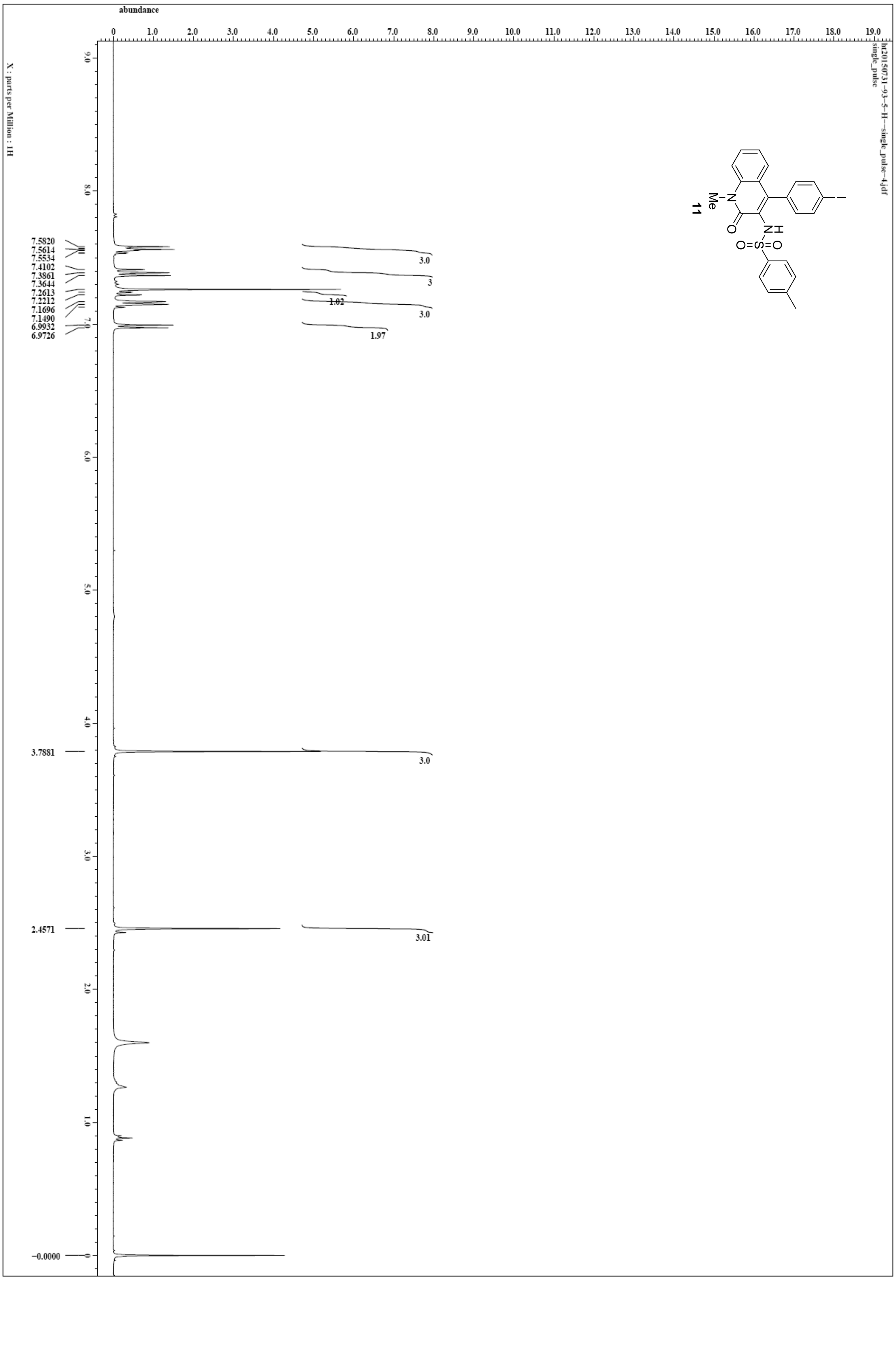
abundance
X : parts per Million : 1H
7.5820
7.5614
7.5534
7.4102
7.3861
7.3644
7.2613
7.2212
7.1696
7.1490
6.9932
6.9726
3.7881
2.4571
-0.0000
3.0
3
1.02
3.0
1.97
3.0
3.01
11
Me
N
O
H
S
O
I

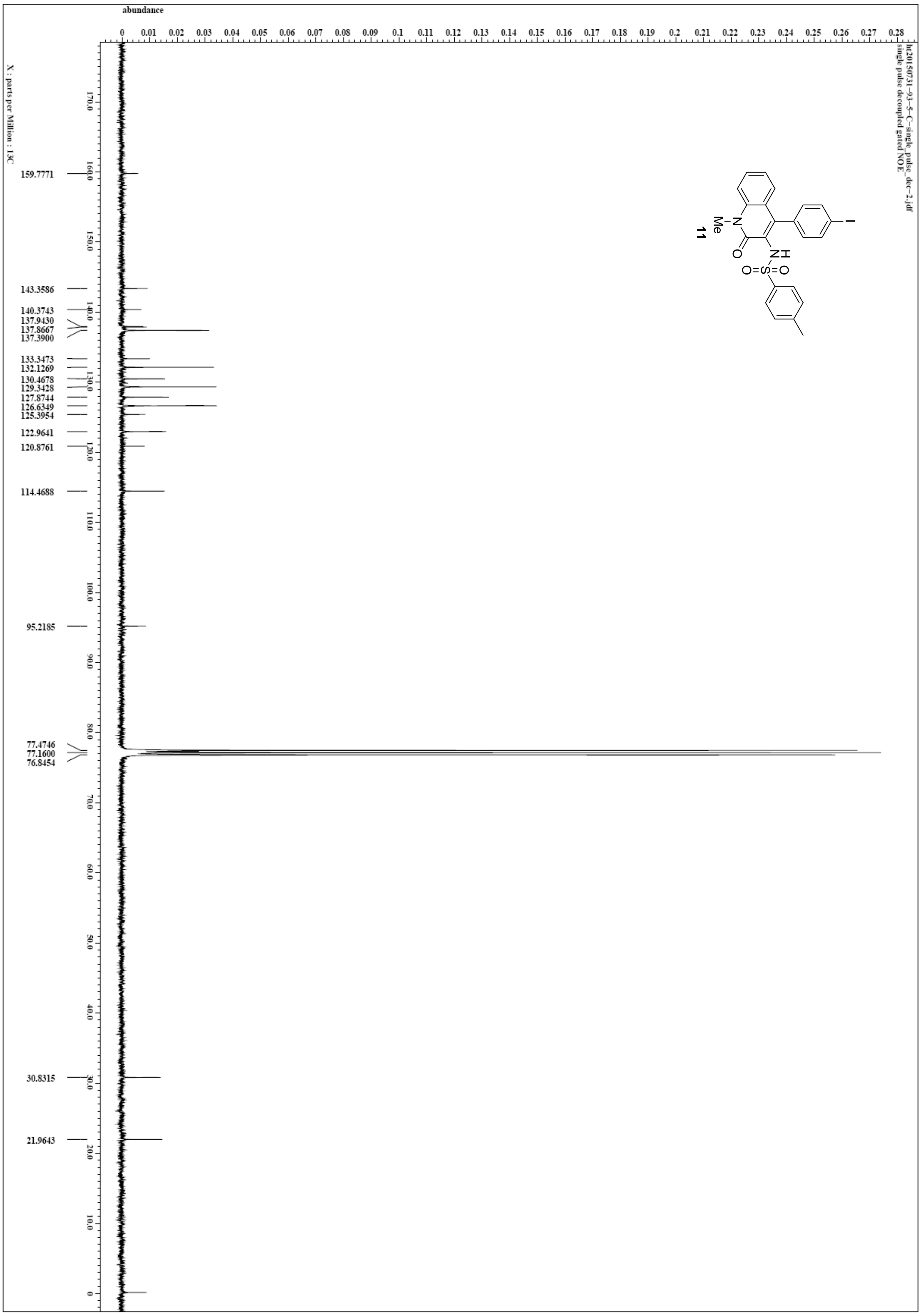
abundance
X : parts per Million : 13C
159.7771
143.3586
140.3743
137.9430
137.8667
137.3900
133.3473
132.1269
130.4678
129.3428
127.8744
126.6349
125.3954
122.9641
120.8761
114.4688
95.2185
77.4746
77.1600
76.8454
30.8315
21.9643
11

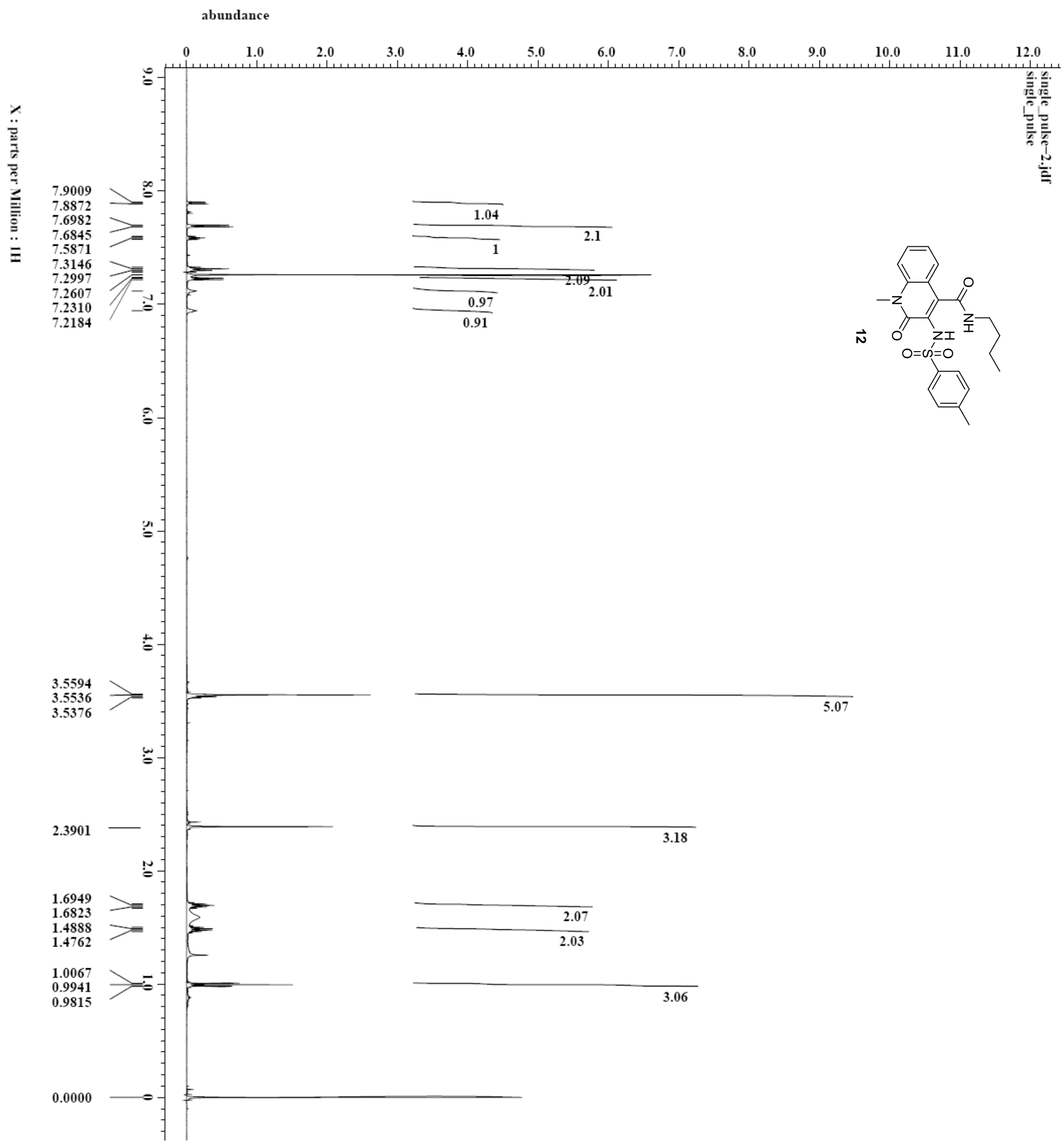
abundance
X : parts per Million : 1H
single_pulse-2.jdf
single_pulse
7.9009
7.8872
7.6982
7.6845
7.5871
7.3146
7.2997
7.2607
7.2310
7.2184
3.5594
3.5536
3.5376
2.3901
1.6949
1.6823
1.4888
1.4762
1.0067
0.9941
0.9815
0.0000
1.04
2.1
1
2.09
2.01
0.97
0.91
5.07
3.18
2.07
2.03
3.06
12

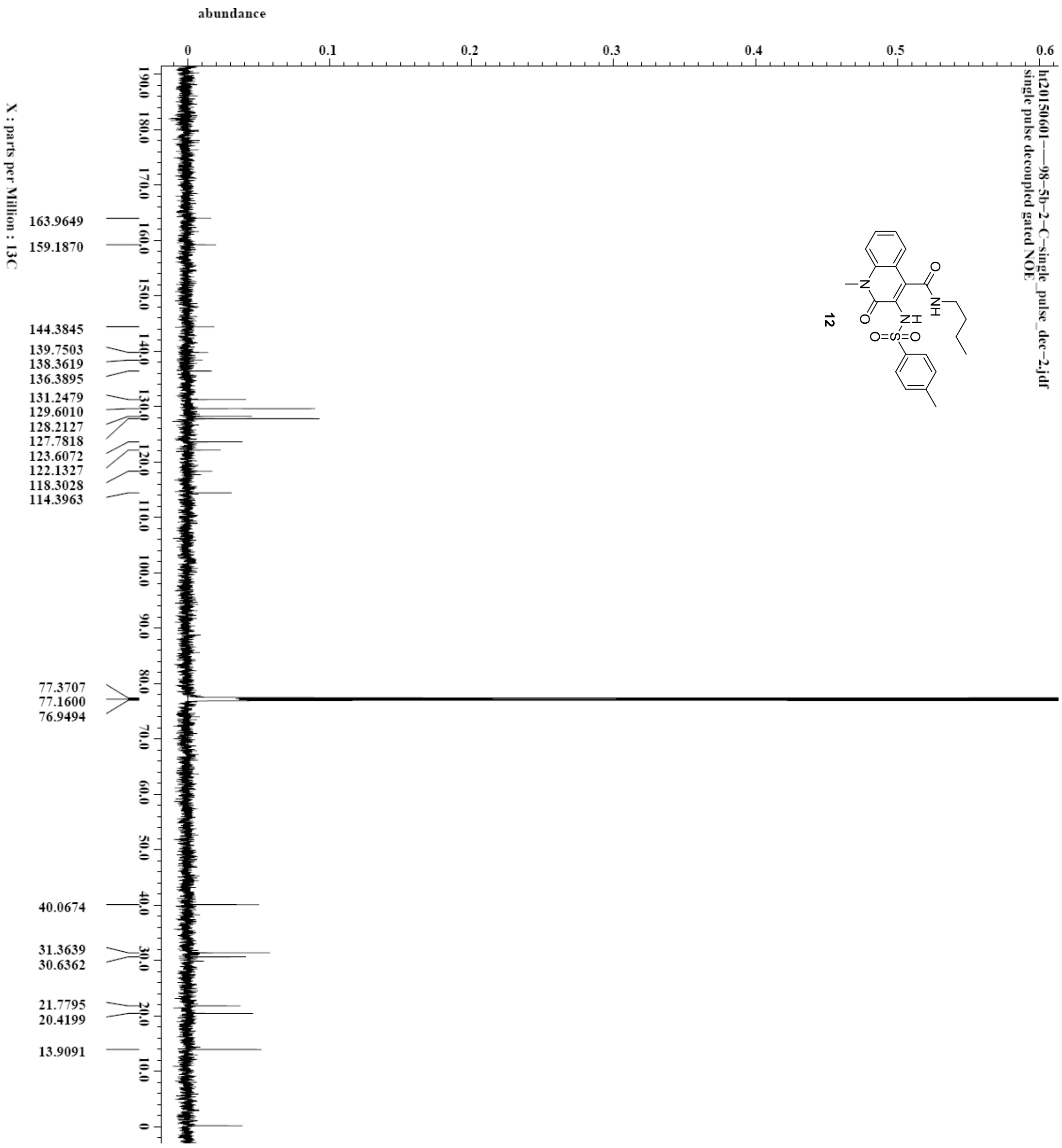

abundance
h20150601—98-5b-2-C-single_pulse_dec-2.jdf
single pulse decoupled gated NOE
12
X : parts per Million : 13C
163.9649
159.1870
144.3845
139.7503
138.3619
136.3895
131.2479
129.6010
128.2127
127.7818
123.6072
122.1327
118.3028
114.3963
77.3707
77.1600
76.9494
40.0674
31.3639
30.6362
21.7795
20.4199
13.9091
